# BEEHAVE: a systems model of honeybee colony dynamics and foraging to explore multifactorial causes of colony failure

**DOI:** 10.1111/1365-2664.12222

**Published:** 2014-03-04

**Authors:** Matthias A. Becher, Volker Grimm, Pernille Thorbek, Juliane Horn, Peter J. Kennedy, Juliet L. Osborne

**Affiliations:** ^1^ Environment & Sustainability Institute University of Exeter Penryn Campus Penryn Cornwall TR10 9FE UK; ^2^ Rothamsted Research West Common Harpenden Hertfordshire AL5 2JQ UK; ^3^ UFZ, Helmholtz Centre for Environmental Research – UFZ Permoserstr. 15 04318 Leipzig Germany; ^4^ Institute for Biochemistry and Biology University of Potsdam Maulbeerallee 2 14469 Potsdam Germany; ^5^ German Centre for Integrative Biodiversity Research (iDiv) Halle‐Jena‐Leipzig Deutscher Platz 5e 04103 Leipzig Germany; ^6^ Environmental Safety Syngenta Jealott's Hill International Research Centre Bracknell Berkshire RG42 6EY UK

**Keywords:** *Apis mellifera*, colony decline, cross‐level interactions, feedbacks, foraging, modelling, multiple stressors, multi‐agent simulation, predictive systems ecology, *Varroa destructor*

## Abstract

1. A notable increase in failure of managed European honeybee *Apis mellifera* L. colonies has been reported in various regions in recent years. Although the underlying causes remain unclear, it is likely that a combination of stressors act together, particularly varroa mites and other pathogens, forage availability and potentially pesticides. It is experimentally challenging to address causality at the colony scale when multiple factors interact. *In silico* experiments offer a fast and cost‐effective way to begin to address these challenges and inform experiments. However, none of the published bee models combine colony dynamics with foraging patterns and varroa dynamics.
2. We have developed a honeybee model, BEEHAVE, which integrates colony dynamics, population dynamics of the varroa mite, epidemiology of varroa‐transmitted viruses and allows foragers in an agent‐based foraging model to collect food from a representation of a spatially explicit landscape.
3. We describe the model, which is freely available online (www.beehave-model.net). Extensive sensitivity analyses and tests illustrate the model's robustness and realism. Simulation experiments with various combinations of stressors demonstrate, in simplified landscape settings, the model's potential: predicting colony dynamics and potential losses with and without varroa mites under different foraging conditions and under pesticide application. We also show how mitigation measures can be tested.
4. *Synthesis and applications*. BEEHAVE offers a valuable tool for researchers to design and focus field experiments, for regulators to explore the relative importance of stressors to devise management and policy advice and for beekeepers to understand and predict varroa dynamics and effects of management interventions. We expect that scientists and stakeholders will find a variety of applications for BEEHAVE, stimulating further model development and the possible inclusion of other stressors of potential importance to honeybee colony dynamics.

## Abstract 2

BEEHAVE offers a valuable tool for researchers to design and focus field experiments, for regulators to explore the relative importance of stressors to devise management and policy advice and for beekeepers to understand and predict varroa dynamics and effects of management interventions. We expect that scientists and stakeholders will find a variety of applications for BEEHAVE, stimulating further model development and the possible inclusion of other stressors of potential importance to honeybee colony dynamics.

## Introduction

A notable increase in failure of managed European honeybee *Apis mellifera* L. colonies has been reported in the US, Europe and other areas of the Northern Hemisphere in recent years (Moritz *et al*. 2010; Potts *et al*. 2010), but the underlying causes remain unclear and may vary according to region (Neumann & Carreck 2010). It is assumed that a combination of several stressors is acting together to cause colony failure (vanEngelsdorp *et al*. 2009; Potts *et al*. 2010). The potential stressors most frequently cited as affecting honeybee colony strength are varroa mites *Varroa destructor* (Rosenkranz, Aumeier & Ziegelmann 2010), viruses transmitted by these mites (Chen *et al*. 2006; Cox‐Foster *et al*. 2007), *Nosema ceranae* infections (Higes *et al*. 2009; Higes, Martín‐Hernández & Meana 2010), pesticides (Thompson 2003; Blacquière *et al*. 2012; Cresswell, Desneux & vanEngelsdorp 2012) and forage quantity and quality (Naug 2009).

Honeybee research at the colony level can be expensive and time‐consuming, often resulting in restricted sample sizes. This problem is exacerbated when the interactions between multiple factors affecting the colony are addressed, and as there are several feedback mechanisms that may dampen or exacerbate effects, the results can be difficult to interpret (Becher *et al*. 2013). While empirical research is essential to create new knowledge, *in silico* experiments can help to provide understanding of the findings and highlight critical knowledge gaps, as they allow us to test and analyse the effects of a variety of factors and interactions between them in a fast and cost‐effective way (Grimm & Railsback 2005). Empirical studies may also benefit from using a model to test hypotheses in advance to refine the experimental setup (Grimm *et al*. 1999). Finally, an established and freely available model will provide a useful tool for beekeepers, landscape managers and policy makers to help in decision‐making with respect to bee, pollinator and land management; for example exploring the benefits or costs of different varroa mite management strategies, or agri‐environment schemes.

Most published honeybee models focus on separate aspects of bee biology, for example, colony dynamics, foraging behaviour or impact of parasites (Martin 1998, 2001; Sumpter & Pratt 2003; Schmickl & Crailsheim 2007; reviewed in Becher *et al*. 2013). A recently published model (Khoury, Barron & Myerscough 2013) represents honeybee colony dynamics and food stores in a simple way, without incorporating disease or foraging dynamics and does not aim to be ‘realistic’ (Khoury, Barron & Myerscough 2013). An integrated model, combining all the different stressors within and outside the hive, and the interactions between them, is not yet available (Becher *et al*. 2013). For this reason, we have developed a model, BEEHAVE, that integrates honeybee colony dynamics, population dynamics of the varroa mite, epidemiology of varroa‐transmitted viruses and allows foragers in an agent‐based foraging model to collect food from a representation of a spatially explicit landscape, as proposed by Becher *et al*. (2013). The model is presented here, together with sensitivity analyses, testing against empirical data and examples of its use. We assume that the here published version of BEEHAVE is a starting point and future versions may follow with improved parameterizations, more realistic processes (e.g. taking differences in pollen quality into account) or additional modules (e.g. for pesticides or Nosema infection).

## Materials and methods

### The model

Here, we provide a summary description of the BEEHAVE model. A full description is included in Appendix S1 (Supporting information), following the ODD protocol (Overview, Design concepts, Details), a standard format for describing individual‐based models (Grimm *et al*. 2006, 2010). Additionally, hyperlinks allow the reader to move between the ODD description and corresponding program code. BEEHAVE is available to download at www.beehave-model.net and is implemented in the freely available software platform netlogo (Wilensky 1999). The program code and a user manual are included in Appendices S2 and S3 (Supporting information). Unless explicitly stated otherwise, we always address the modelled colony when using biological terms like ‘colony’, ‘foraging’, etc. in the following description.

**Figure 1:**
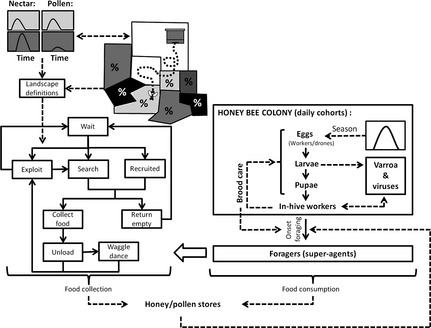
Overview of the BEEHAVE model structure: Based on the egg‐laying rate and interacting with the varroa and foraging modules, the structure of a single honeybee colony is modelled. A separate landscape module allows the determination of detection probabilities (%) of flower patches by scouting bees and definition of their nectar and pollen flows over the season. This information is then taken into account when foragers collect food in an agent‐based foraging module. Note that the various mortalities implemented in the model are not shown in this figure.

The purpose of BEEHAVE is to explore how various stressors, including varroa mites, virus infections, impaired foraging behaviour, changes in landscape structure and dynamics, and pesticides affect, in isolation and combination, the performance and possible decline and failure of single managed colonies of honeybees. The model consists of three integrated modules: the colony, the mite and the foraging model (Fig. 1). An additional, external landscape module (M.A. Becher, V. Grimm, P. Thorbek, P.J. Kennedy & J.L. Osborne, unpublished data) can be used to create input files (Table S2, Supporting information) for the foraging model. We did not add a Nosema module at this point, as the mechanisms of transmission are still not well understood (Higes, Martín‐Hernández & Meana 2010), but we provide some suggestions of how Nosema infections can be addressed by changing some parameters. The colony model describes in‐hive processes, using difference equations to generate the colony structure and dynamics for the brood, in‐hive worker and drone population. The mite model describes the dynamics of a varroa mite population within the honeybee colony. As vectors for viruses, mites affect the mortality of bee pupae and adult bees. Viruses are not implemented as entities but via infection rates of mites and bees (BEEHAVE considers one type of virus at a time). The colony and mite model proceed in daily time steps.

The foraging model is an agent‐based model, which represents foraging at flower patches located in the landscape around the hive. Space is represented implicitly: properties of these flower patches, such as probability of being detected by scouting bees, distance to the hive, or nectar and pollen availability, are either set by the modeller when exploring hypothetical landscapes or extracted from real crop maps using the external landscape module. Landscape dynamics, including changes in location and availability of crop fields of different types, can be taken into account by updating the imported landscape data at every time step of the colony model. The foraging model, which is executed once per day, includes a varying number of foraging trips, depending on the quality of nectar and pollen sources in the landscape, the weather conditions and the size, stores, and demand of the colony. The foraging processes represented thus operate on the time‐scale of minutes.

The structure of the model is a compromise between structural realism (i.e. the ability to represent heterogeneity where it is likely to matter) and computational efficiency and parsimony regarding parameterization and model analysis. Hence, the in‐hive bee population is represented via age cohorts, foraging bees via ‘super‐individuals’ (Rose, Christensen & DeAngelis 1993; Scheffer *et al*. 1995; Grimm & Railsback 2005), mites via the total number of virus‐free and virus‐carrying mites and viruses via transmission rates between mites and bees. We also considered it essential to explicitly represent environmental factors driving colony dynamics and to link within‐hive dynamics and foraging by representing the seasonally dynamic storage, consumption, demand and collection of nectar and pollen. Including this level of complexity ensures that links and feedback mechanisms between reproduction, brood, food stores and foragers can be successfully captured.

### Colony model

The entities comprising the colony are (1) age classes (cohorts) of eggs, larvae, pupae and adults, both for in‐hive worker bees and drones, (2) the hive and (3) optionally, the queen. Bees are distinguished by cohort identity number, age, sex, exposure to varroa mites and virus infection, plus auxiliary variables that keep track of mortality and infections. The hive is characterized by the honey and pollen stores, with maxima set for honey stores and brood space. The queen is defined by her egg‐laying rate over the season, which can optionally be influenced by the relative amount of brood and the queen's age. A commented list of the model's entities and their variables can be found in Table S1 (Supporting information) (worksheet ‘Entities state variables’).

### Mite model

The mite model describes the dynamics of a varroa mite population within the honeybee colony and is based on an established varroa/virus model (Martin 1998, 2001). As vectors for viruses (either deformed wing virus, DWV, or acute paralysis virus, APV), mites can affect the mortality of bee pupae and adult bees. The entity of the mite model is the population of phoretic mites, that is, mites attached to adult bees, and is characterized by its size and the proportion of virus‐free phoretic mites.

To reproduce, phoretic mites enter drone or worker larvae cells shortly before the cells are capped. Such larval cells are thus invaded by 0–8 mites for worker cells and up to 16 mites for drone cells, respectively. Following Boot *et al*. (1995), we randomly distribute the mites over suitable cells, which affects mite reproduction and virus transmission rates from mites to bee pupae and from infected bee pupae to mites.

### Foraging model

The foraging model comprises two entities: foragers and flower patches. Foragers are ‘super‐individuals’ representing a given number of identical and identically behaving foragers; super‐individuals are referred to as foragers henceforth. The number of foragers represented by one super‐individual is determined via the parameter *Squadron_Size*, which is set to 100 in all analyses presented below. Foragers are created from those cohorts of adult in‐hive workers that reach the age of first foraging (AFF). Foragers are characterized by the state variables listed in Table 1 of Appendix S1 (Supporting information). Flower patches in the landscape provide seasonally variable nectar and pollen resources, for example crop fields. As the foraging model is spatially implicit, flower patches do not have a spatial extent or shape, but are characterized by the state variables listed in Table 3 of Appendix S1 (Supporting information).

### Process overview and scheduling

Figure 1 provides a conceptual overview of the processes represented in the model. In the colony model, eggs laid by the queen according to her variable egg‐laying rate develop into larvae, then pupae, and in‐hive worker bees or drones. Worker bees turn into foragers at AFF, which varies with the brood to in‐hive bee ratio, total honey and pollen stores, protein content of jelly and the in‐hive bee to forager ratio. In addition to background mortalities, survival of the brood is affected by the number of in‐hive bees available for nursing, the protein content of jelly fed by nurse bees, and by virus infections. Honey and pollen stores are decreased by the consumption rates of adult bees and larvae and increased by successful foraging.

The mite model is called upon once during each time step. Data on all mites that invade brood cells on this day are stored. The number of newly infected mites and worker bees is determined, and mortality of the mites applied. Moreover, the submodel *MiteReleaseProc* will be called upon whenever bees emerge from brood cells or when pupal brood dies.

The foraging model is also called upon once each time step when the variables characterizing the flower patches and various aspects of foraging are updated (i.e. an individual beginning and finishing foraging; searching for flower patches; collecting nectar or pollen; dancing for recruitment of other foragers; or unloading their crop). The foraging decisions of the bees are either guided by the energetic efficiency of a food source (for nectar collection) or by the total trip duration (for pollen collection). Additionally, energy gains and losses of each foraging trip are calculated, and foraging mortality, based on trip duration, takes place. Foragers are assumed to die at latest when they have flown 800 km (Neukirch 1982) or reach a maximum age.

### Default settings

Unless otherwise stated, simulations were run using the following default settings [Table S1, Supporting information (worksheet ‘Simulations’)]: Simulations start on 1st January with 10 000 worker bees. Food is offered by two generic flower patches: one 500 m from the colony flowering March–November and one 1500 m away flowering January–September. Flowering of the patches follows a bell‐shaped curve with a maximum production of 20 L nectar (concentration: 1·5 mol L^−1^) and 1 kg pollen per day. Weather defines the daily foraging period and is based on real weather data (‘Rothamsted 2009’; available at: http://www.era.rothamsted.ac.uk). Plots visualizing all environmental drivers used in this default setting are in Table S1 (Supporting information) (worksheet ‘Drivers’). No varroa mites are present, and no beekeeping practices take place in this scenario.

For some of the tests and applications, modified scenarios for the environmental drivers, for example, weather or nectar and pollen availability, were chosen. For ease of interpretation, these deviations from the default are noted in the Results section. Parameterization for all simulations can be found in Table (Supporting information) (worksheet ‘Simulations’); moreover, for each figure in the results section, the corresponding netlogo program used (containing the definition of the simulation experiment) is included in Appendices S3 or S5–S8 (Supporting information).

### Model testing

#### Verification of the code

The correctness of the code was thoroughly checked during model development. Visual testing was performed using a wide range of plots and symbols that allow monitoring of the model behaviour (Table S1, Supporting information (worksheet ‘Plots’) lists all output options of the BEEHAVE interface). ‘Assertions’ are placed at various locations in the code and stop the program if state variables assume values beyond a defined range (Table S1, Supporting information (worksheet ‘Assertions’) provides a list of all assertions included in the program). Finally, the complete code was scrutinized separately by two co‐authors who were not involved in writing the program.

#### Testing the model

We graphically compared model output of all three modules with data from literature (experiments and other models). No statistical analyses are presented as the data on environmental drivers underlying empirical findings were usually not available in the literature, and it is therefore only appropriate to describe overall trends of the data. Ten replicates of each scenario were run unless stated otherwise. For the colony model, we compared the model outputs of colony dynamics, AFF and life span over 1 year with empirical data. The AFF and life span of workers were not calibrated but are emerging properties of the model and are the result of a complex set of factors and feedback mechanisms and are therefore an excellent pattern for validation (Grimm *et al*. 2005). The foraging model was tested by simulating a feeder experiment by Seeley, Camazine & Sneyd (1991).

#### Sensitivity analysis

Sensitivity analyses were carried out for the default setting when varroa mites were added (10 virus‐free and 10 virus‐carrying mites on day 0 of the simulation). Sixty‐one parameters were tested individually, as testing the number of parameter combinations necessary for a full global sensitivity analysis is not possible within a realistic time‐scale. Each parameter was multiplied by a factor ranging from 0·1 to 4 (Table 1), except when the default value was 0 or an integer value was required (details of the sensitivity analyses are given Appendix S4, Supporting information). *Squadron_Size* varied from 1 to 1000. Colony size after 3 years was used as output, averaged over 10 replicate simulations.

**Table 1:** Sensitivity analyses

Parameter (no varroa)	Effect (Δ bees)	Parameter (with varroa)	Effect (Δ bees)
MORTALITY_FOR_PER_SEC	−16800	MITE_FALL_WORKERCELL	12208
MAX_BROOD_NURSE_RATIO	12005	MORTALITY_FOR_PER_SEC	−11728
CROPVOLUME	11463	TIME_NECTAR_GATHERING	−7690
TIME_NECTAR_GATHERING	−10982	MORTALITY_INHIVE	−7173
LIFESPAN	10754	MAX_EGG_LAYING	7148
CONC_G	10311	CONC_G	6972
MAX_EGG_LAYING	9604	MORTALITY_INHIVE_INFECTED_AS_PUPA	−6815
MORTALITY_INHIVE	−9569	MITE_MORTALITY_BROODPERIOD	6613
MAX_PROPORTION_POLLEN_FORAGERS	6609	MAX_BROOD_NURSE_RATIO	6388
POLLENLOAD	5950	CROPVOLUME	6187

Listed are the 10 most important parameters when either varroa is present or absent. ‘Effect (Δ bees)’ describes the difference in colony size after 3 years, when comparing the sensitivity factors 2 and 0·5. For example, if the default value of MORTALITY_FOR_PER_SEC is doubled, the colony size is 16 800 bees smaller than when the forager mortality is halved. Hence, negative values indicate a negative correlation between the parameter and the colony size. The complete sensitivity analyses for 61 parameters are provided in Appendix S4, Supporting Information. [Description of parameters and their default value: CONC_G, sucrose concentration in nectar of the closer food source (1·5 mol L^−1^); CROPVOLUME, volume of a forager's crop, is completely filled at flower patch (50 μL); LIFESPAN, maximum life span of a worker bee (290 days); MAX_BROOD_NURSE_RATIO, maximum amount of brood, nurse bees can care for (3 pre‐adults/nurse); MAX_EGG_LAYING, maximum egg‐laying rate per day (1600 eggs/days); MAX_PROPORTION_POLLEN_FORAGERS, maximum proportion of pollen foragers (0·8); MITE_FALL_WORKERCELL, probability that a mite emerging from a worker cell will fall from the comb and die (0·3); MITE_MORTALITY_BROODPERIOD, mite mortality rate per day during brood period (0·006); MORTALITY_FOR_PER_SEC, mortality rate of foragers per second of foraging (0·00001); MORTALITY_INHIVE, daily mortality rate of healthy in‐hive bees and foragers (0·004); MORTALITY_INHIVE_INFECTED_AS_PUPA, daily mortality rate of in‐hive bees and foragers, infected as pupae with deformed wing virus (DWV) (0·012); POLLENLOAD, amount of pollen collected during a single, successful pollen foraging trip, equals two pollen pellets (0·015 g); TIME_NECTAR_GATHERING, time to fill crop with nectar if nectar quantity in the food patch is not yet reduced (1200 s)].

### Model applications

We chose three scenarios to demonstrate the application of the model to practical honeybee and land management issues: (1) simulations of colony, varroa and virus dynamics, and the response to acaricide treatment over 5 years, (2) comparison of colony growth and survival with and without varroa mites, under different foraging conditions to explore the interactions between parasitism and food limitation, and (3) simulations of the effect of doubled forager mortality at different times of the year on colony survival, as explored by Henry *et al*. (2012) and Cresswell, Desneux & vanEngelsdorp (2012) in relation to pesticide exposure using a highly simplified honeybee model by Khoury, Myerscough & Barron (2011).

To systematically explore the chosen stressors, all applications used highly stylized landscapes and excluded beekeeping practices (except for varroa treatment in the varroa scenario).

## Results

### Model testing

#### Sensitivity analysis

Sensitivity of most parameters was low, indicating that our comparisons with empirical data and our demonstration examples are robust to small changes in these parameters (Appendix S4, Supporting information). The overall low sensitivity might be explained by the number of feedback mechanisms in the model, which allow the colony to compensate for changes in one of the submodels. For example, a reduced efficiency in brood care could partly be mitigated by a delay in the AFF. The parameters with the strongest impact on colony size were related to mortality [of foragers, in‐hive bees or mites (if present)], energy influx (crop volume, handling time for nectar, size of pollen load) and colony growth (maximal egg laying, efficiency of brood care) (Table 1).

#### Colony dynamics

##### Setting

Three different settings were used to simulate foraging conditions (*n* = 10 replicates for each): (1) ‘BEEHAVE default setting’ using real weather data (Rothamsted, 2009), (2) ‘BEEHAVE artificial weather’ simulating more favourable conditions: daily foraging period follows a bell‐shaped curve over the year (maximum 12 h per day), (3) ‘BEEHAVE ideal’: honey and pollen stores set to remain full so that no foraging takes place, to demonstrate the maximum potential growth rate that can be achieved by the model.

##### Output

**Figure 2:**
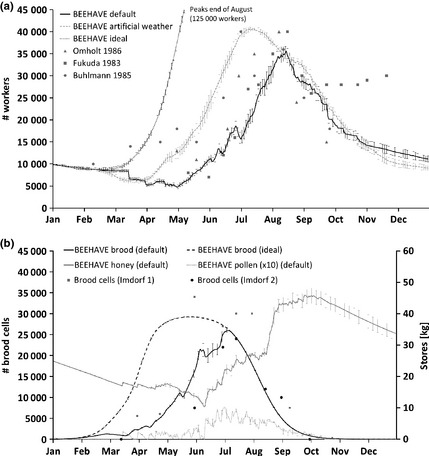
(a) Colony dynamics of BEEHAVE under three sets of conditions: the default setting (continuous line), a setting with favourable, artificial weather data (dashed line) and a setting with ideal food supply that requires no foraging (dotted line) (mean ± SD; *n* = 10) in comparison with data from literature (data redrawn from Schmickl & Crailsheim 2007). Under ideal food supply, the model colonies peak at the end of August (125 000 workers) and contain about 80 000 bees at the end of the year (*y*‐axis truncated for clarity). Error bars are shown for every second day. (b) Numbers of worker brood cells, and honey and pollen stores under the BEEHAVE default setting, and numbers of brood cells under ‘ideal’ conditions (mean ± SD; *n* = 10). Note that pollen stores are shown as increased by a factor of 10 for clarity in the figure. Empirical brood data redrawn from Imdorf, Ruoff and Fluri (2008) (squares: fig. 2 (‘control’), *n* = 8; circles: fig. 14 (‘carnica’), *n* = 54). Error bars are shown for every fifth day.

The colony dynamics were within the range of those reported in the literature (Fig. 2a). The three simulated scenarios suggest daily foraging conditions are important for colony dynamics (Fig. 2a), although weather and forage conditions are not reported in the empirical studies so exact comparison was not feasible. Under the default setting, the start of colony growth was delayed due to a loss of foragers at the onset of foraging (higher mortality than in‐hive bees) and the colony peaked in mid‐August (similar to Fukuda 1983 and Omholt 1986) with ca. 36 000 bees. Real colony sizes can also have peak abundances lower than 40 000 bees; Imdorf, Ruoff and Fluri (2008) monitored colony dynamics at six different locations, where maximal colony sizes ranged between ca. 16 000 and 26 000 bees. Under favourable, artificial weather conditions, the colony grew more quickly, peaking in mid‐July at ca. 40 000 workers; matching data from Bühlmann (1985). Under ‘ideal’ conditions where no foraging is required, colony growth was much faster and stronger.

In spring and early summer, under default conditions, the slow increase in the abundance of brood in the colonies (Fig. 2b) was caused by insufficient pollen stores and bees available to care for queen and brood, which via feedback mechanisms reduced the egg‐laying rate and increased brood mortality. This resembled the pattern shown in Imdorf, Ruoff & Fluri (2008). These factors were no longer limiting in late summer and autumn, and the number of brood cells was then directly related to the seasonally changing egg‐laying rate. Under ‘ideal’ food conditions, the bell‐shaped curve of the number of brood cells (Fig. 2b) reflects the egg‐laying rate (when not constrained by lack of pollen or bees caring for brood), and brood died only because of the daily, stage‐specific mortality.

#### Onset of foraging and life span

##### Setting

Default setting with additional recording of output for the onset of foraging and the life span of individual foragers.

##### Output

**Figure 3:**
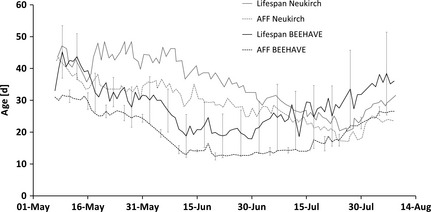
Modelled average age of first foraging (AFF) of workers and average life span (mean ± SD; *n* = 10 simulations, under default setting) depending on their hatching date, in comparison with empirical data (redrawn from Neukirch 1982, fig. 1). Error bars are shown for every fifth day.

Under the default setting, predicted average AFF and average life span (Fig. 3) were of similar range to those reported by Neukirch (1982), but showed different predicted patterns over time. Neukirch (1982) did not report the weather and foraging conditions under which the experiment was conducted so we could not replicate her experimental setup directly. Nonetheless, the main trend of a decline of AFF and life span during May is reproduced by the model, as well at the relative size of that decrease. Likewise, the correlation between AFF and average life span (driven mainly by forager mortality) is captured. BEEHAVE and the empirical data differ in that BEEHAVE predicts turning points at the end of June, while the Neukirch (1982) data show a later transition.

#### Simulating Seeley's feeder experiment

##### Setting

Default setting with a modification to simulate the setup of Seeley, Camazine and Sneyd's (1991) feeder experiment. On the day of the experiment, flower patches in the landscape were replaced by two feeders at a distance of 400 m, offering nectar but no pollen. Time to gather a nectar load was set to 79 s (Seeley 1994); nectar concentration was set to 0·75 mol L^−1^ (‘North’) for one feeder and to 2·5 mol L^−1^ (‘South’) for the other feeder. Nectar concentrations were swapped after 4 h. Forager traffic at each feeder in each foraging round was recorded.

##### Output

**Figure 4:**
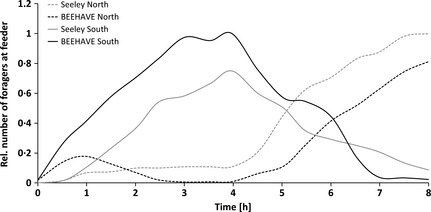
Simulation of a feeder experiment by Seeley, Camazine and Sneyd (1991) on 19th June: Two feeders are set up 400 m north and south of the colony with sugar concentrations of 0·75 and 2·5 mol L^−1^, respectively. After 4 h, the two feeders are switched. The number of visits at each feeder relative to the maximum number of visits over time is shown for BEEHAVE simulations compared with the redrawn empirical data. Simulations are based on 10 replicates with the number of visits being averaged for each 30 min time slot.

Seeley, Camazine and Sneyd's (1991) experiment showed that foragers switched from the South to the North feeder when the energetic rewards were changed and BEEHAVE captured this switch in feeder preference by the foragers very well (Fig. 4). The slope of the relative number of visits over time, as well as the timing of the switch, was in agreement with Seeley, Camazine & Sneyd (1991).

### Model applications

#### Varroa mites, virus infection and acaricide treatment

##### Setting

Three scenarios were compared, running the model for 5 years: (1) default setting (no mites); (2) default setting with the addition of mites and DWV virus infection (adding 10 virus‐free mites and 10 virus‐carrying mites on day 0); (3) as setting 2 but with simulated use of a common acaricide (assuming no resistance), implemented by adding an additional 11·5% daily mortality of mites for 40 days (Fries, Camazine & Sneyd 1994) starting on 27th September each year.

##### Output

**Figure 5:**
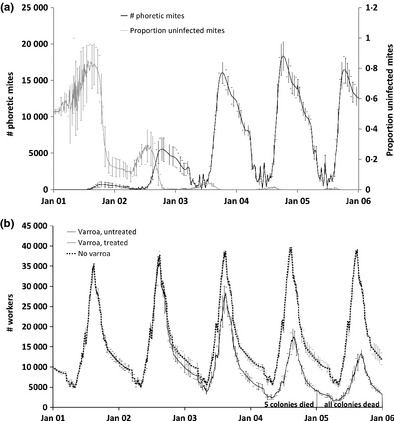
(a) Modelled dynamics of the number of varroa mites transmitting deformed wing virus in a colony and the proportion of mites that are uninfected (mean ± SD; *n* = 10) when 10 virus‐free and 10 virus‐carrying mites were introduced to the colony at the beginning of each simulation (scenario 2). Only colonies that remained alive were included in the calculation of the mean. Error bars are shown for every tenth day. (b) Honeybee colony dynamics in the presence of virus‐carrying varroa mites (with and without acaricide treatment) and without mites (mean (±SD for varroa and untreated); *n* = 10). The dotted and grey lines overlap because of acaricide‐treated colonies have similar dynamics to those without varroa. Error bars are shown for every 10th day.

With respect to scenario 2, as mites reproduced in the capped brood cells, the mite population peaked in late summer and declined during the broodless winter of each year (Fig. 5a). In this scenario, 50% of the mites were initially carrying DWV. As virus‐carrying mites had a reduced reproductive success due to increased mortality of infected bee pupae, the proportion of virus‐free mites increased during the first 6 months (Fig. 5a). However, when the mite population was growing and the brood nest was shrinking at the end of the summer, multiple mite infestations of single brood cells were more widespread, and the virus spread faster in the mite population.

There is little difference between the colonies with and without varroa, or acaricide, in year 1 and 2 (Fig. 5b). However, as the mite population built up (Fig. 5a) and virus spread in the varroa and bee population under scenario 2 (varroa, untreated), colony sizes began to decline compared with colonies without varroa (scenario 1), leading to the death of five colonies in the fourth winter and the other five in the fifth winter (Fig. 5b).

If the colonies were treated against varroa with an efficacious acaricide every autumn, the number of worker bees was almost unaffected by the varroa mites so that scenario 1 (no varroa) and scenario 3 (varroa; treated) had very similar colony dynamics, and none of the colonies in these scenarios died.

#### Interaction between varroa and forage availability

##### Setting

Default setting either without varroa mites (control) or with mites (as above). Scenarios were run with the main (i.e. closer) forage patch set at three different distances: *Distance_G*: 250, 500 or 1000 m. (Total 6 scenarios; 10 replicates of each). All scenarios were run for 5 years, reporting colony sizes and losses at the end of each year (a colony was presumed dead if there are less than 4000 bees on 31 December).

##### Output

In settings without varroa mites, none of the colonies died irrespective of distance to the forage patch. The presence of varroa caused colony deaths with all three forage patch settings, but losses occurred more quickly when patches were further away. For the scenarios with mites: (i) with forage at 1000 m, two colonies died in year 3 and the rest died in year 4, (ii) with forage at 500 m, five colonies died in year 4 and the rest in year 5, (iii ) with forage at 250 m, two colonies died in year 4 and another five colonies died in year 5. The increasing prevalence of the virus in the bee population caused the colonies to decline over the years (as in Fig. 5b), having a stronger effect than forage patch distance. There was, however, a combined effect of mite‐driven mortality with increased forager mortality resulting from the longer distances flown to the further patches, which tipped many colonies over a threshold to failure.

#### Forager mortality after pesticide treatment

##### Setting

Default setting with (i) modified forage availability (high or low forage flow) in combination with (ii) doubled probability of forager mortality. (i) Forage availability: In all scenarios, the landscape consisted of a single forage patch placed 1 km from the hive, with constant nectar (1·5 M) and pollen flow throughout the year. For ‘high forage flow’ conditions, the nectar and pollen flow were high enough to pose no limitation on colony growth (10 L nectar and 1 kg of pollen per day). For low forage flow conditions, the pollen and nectar flow were deliberately set to keep the colonies at the threshold of survival (3 L nectar and 0·5 kg of pollen per day). (ii) Forager mortality: Henry *et al*. (2012) studied the impact of a 30‐day period of doubled forager loss on colony growth, which was predicted to result from pesticide exposure (but see Cresswell & Thompson 2012). We simulated similar conditions by doubling the chance of mortality for each forager visit to the food patch over a 30‐day period, resulting in all successful foragers being exposed as there was no alternative forage. To further study the effect of the timing of exposure, we ran scenarios (at both high and low forage flow) with the doubled mortality implemented separately for each month of the year. We also ran control scenarios without increased forager mortality. Total scenarios = (2 × forage availability) × (12 × mortality each month + control) = 26; 20 replicates of each. All scenarios were run for 5 years, reporting colony sizes and losses at the end of each year.

##### Output

**Figure 6:**
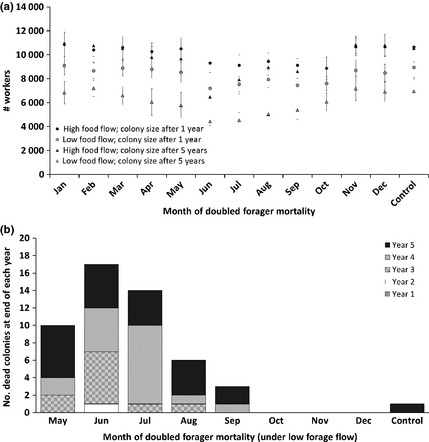
(a) The consequences of doubling forager mortality at a forage patch in different months for a 30 days period (*x*‐axis) were explored via simulation and compared with an untreated control. Impact of increased forager mortality on modelled colony size is shown (mean ± SD; *n* = 20) after one or 5 years, if a single food patch was present. The patch provided food constantly throughout the year, as either a relatively high food flow or a relatively low food flow. The mean number of workers is based on surviving colonies only (i.e. excluding dead colonies). (b) Number of colonies dying during each year when limited by forage availability (low food flow scenario) and when doubled forager mortality is imposed for 1 month of each year (timing on *x*‐axis). Results are shown for 20 replicates, so colonies were lost when exposed in May (50%), June (85%), July (70%), August (30%) and September (15%) and in the control (5%). No colony losses occurred from January to April. No losses occurred under the high food flow scenario, regardless of exposure date.

Low food flow led to smaller colonies than high food flow and the difference increased over the 5 years of simulation (Fig. 6a). One control colony was lost of 20 at low food flow (Fig. 6b). With high food flow, doubling forager mortality for 30 days had some impact on colony size (Fig. 6a), but no colonies were lost after 5 years (Fig. 6b), regardless of the timing of exposure. However, at low food flow, the increased forager losses led to much larger reduction in colony sizes (Fig. 6a). The effects of forager losses accumulated over time, resulting in colony losses after two to 5 years, when the 30‐day treatment period was between May and September (Fig. 6b).

By the end of year 5, 85% of the colonies were dead under low food flow conditions when the double forager mortality occurred during June. The life span of adult workers (i.e. foragers and in‐hive bees) between 15th and 30th June was 18·3 ± 3 days for the control and 13·0 ± 3 days if treatment was in June (low food flow scenario, first year).

## Discussion

We have presented BEEHAVE, the first honeybee model that integrates processes within the hive and in the landscape and thereby allows representation of various stressors and their interactions in a more realistic way than previous models. There is also the option of including the effects of other stressors in the future, either by changing parameter values accordingly, or coding further modules. This model, if it is well tested and captures the most important processes in real honeybee colonies, will be an invaluable tool for exploring the relative importance of stressors to devise management and policy advice designed to reduce losses of honeybee colonies.

### Indicators of structural realism

The colony dynamics of the BEEHAVE model are within the range of experimental data and indicate how BEEHAVE is driven by the availability of nectar and pollen within the hive and in the landscape. The level of pollen and nectar stores in the colony affects the age at which workers are sent out to forage, and this can have knock on effects on colony size as mortality of foragers is higher than for in‐hive bees. BEEHAVE captures the internal relationships between season, colony size, and honey and nectar stores well.

We used AFF and the average life span of worker bees as indicators to demonstrate that the most important feedback mechanisms within a colony have been captured sufficiently well, because they are affected by a large number of factors which interact in a complex way (see Materials and methods). Survival of the brood is affected by the number of in‐hive bees available for nursing and the protein content of jelly fed by nurse bees, and survival of foragers is mainly determined by their activity level (total foraging time). Because of these complex relationships, it would have been impossible to directly impose AFF and average life span by choosing the correct phenologies of environmental drivers. They emerge from the model and, while the magnitudes and turning points of average AFF and life span over time do not match the empirical data well (possibly because environmental conditions differ between simulations and the Neukirch 1982 experiment), the overall trends are similar and the correlation between these indicators is well captured. Thus, a central feedback mechanism of real honeybee colonies, the change of AFF in response to the colony's state and demands, is realistically represented in the model. Although it would be possible, via calibration, to obtain better fits between these data and the model output, this would defeat the purpose of the model to mechanistically represent the internal mechanisms of honeybee colonies and how they respond to changes in the environment (rather than mimic a single environmental setting).

The foraging model was tested against the results of Seeley, Camazine and Sneyd (1991). This feeder experiment has become a standard test for individual‐based foraging models of honeybees (e.g. De Vries & Biesmeijer 1998, 2002; Johnson & Nieh 2010; Schmickl, Thenius & Crailsheim 2012) because the colony's behaviour observed in the experiment is the summary outcome of a complex network of processes, including searching, recruitment of foragers and accounting for the energetic efficiency of foraging. Hence, reproducing the results of the feeder experiment is generally taken as a strong indicator that a foraging model is structurally realistic. BEEHAVE captured the switch of foragers between feeders very well. BEEHAVE is the first integrated colony model that can reproduce these patterns, even under the default parameterization which was not optimized to closely fit the empirical data.

When varroa mites were included, the model output compared well with predictions of the models developed by Martin (1998, 2001). If DWV‐carrying mites are present in BEEHAVE, the mites will start affecting the colony in the third year and severely damage it in the fourth year, resulting in the death of the colony in the fourth or fifth winter. In contrast, colonies with DWV‐carrying mites survive in Martin's (2001) model for only two summers and die during their second winter or the following spring. This seems to be surprising, as we developed our varroa model on the basis of Martin's (2001) model. However, Martin uses the critical colony size of 4000 bees (threshold for colony failure) throughout the winter, whereas in our simulations, this same critical colony size is implemented on the last day of the year. If we apply the same criterion for colony survival as Martin (2001), then most varroa‐infested colonies actually die in the spring of the third year (data not shown). Without varroa, nine of 10 colonies survive for at least 5 years under this tightened survival criterion. Empirical survival experiments by Fries, Imdorf and Rosenkranz (2006) with non‐treated, varroa‐infested colonies show that most colonies died during their third and fourth winter after varroa infestation. From our results, we conclude that the varroa model is sufficiently realistic. The criterion used for assuming when a colony essentially fails can have a strong influence on predicted extinction dynamics. Predictions regarding colony losses over the course of time should be considered as relative, not absolute predictions.

BEEHAVE is thus shown to give realistic and robust predictions of colony dynamics, under different conditions. However, there are processes which are not yet included in the model, such as dynamic task allocation and temperature regulation within the hive, and the effects of bacterial and microsporidian pathogens. The model is also not able to predict synergistic effects between stressors on an individual. Elucidation of such interactions should be carried out experimentally or using toxicokinetic/toxicodynamic models or similar; once quantified they can later be included in BEEHAVE if desired. In its current form, BEEHAVE also allows us to address some problems, which are not explicitly implemented. Nosema infections for example could be simulated by increasing the background mortality (Higes, Martín‐Hernández & Meana 2010) and the energy consumption rates (Naug & Gibbs 2009) of bees, by modifying AFF towards an earlier onset of foraging (Mayack & Naug 2009; Dussaubat *et al*. 2013) and by increasing the foraging mortality (Kralj & Fuchs 2010). With respect to future development of the model, some factors can be taken into account via parameter variation (as above), some might be considered in future modules, but some would require a fully individual‐based design of the model, with internal states and decision‐making of bees explicitly represented. The development of all of these is likely to require further data.

### Sensitivity analysis

In our sensitivity analysis, we varied one parameter at a time, but over large ranges. This goes far beyond local sensitivity analysis, the most common type of sensitivity analysis in ecological modelling, where parameters are changed by only 5–10%. We thus performed 61 ‘sensitivity experiments’ (Railsback & Grimm 2012), which give a quite comprehensive overview of how single parameters affect model behaviour. Our analysis did not, however, cover interaction between parameters, which would have required a global sensitivity analysis based on some systematic sampling of parameter space (Saltelli *et al*. 2008; Saltelli & Annoni 2010). Although such an analysis might be desirable, it requires running the model for a very large number of parameter combinations, with significant run time and computing power implications.

### Initial applications of BEEHAVE

Here, we provide evidence that BEEHAVE is realistic enough for exploring the impact of various stressors on honeybee colonies and provides a sample of applications to demonstrate how BEEHAVE can be used to address practical questions.

BEEHAVE output demonstrated that a single annual treatment with an effective hypothetical acaricide can protect varroa‐infested colonies from failure over 5 years. This indicates how beekeeping interventions can be implemented within BEEHAVE to explore the relative effects of different mite management options on colony, mite and virus dynamics. If required, resistance of varroa to an acaricide could be addressed by reducing its efficacy.

We also found increasing distance to forage decreased survival time of varroa‐infested colonies. The increased distance led to higher foraging costs in terms of energy expenditure and forager mortality, which hugely reduced honey stores. Certainly, the default landscape setting used is simplified, and further scenarios representative of realistic nectar and pollen landscapes will be needed to better understand the impact of landscape structure and dynamics on the colony. However, it does indicate that with multiple stressors, the increase in one (distance) can lead to greater sensitivity to another (varroa).

The final scenario simulated doubled forager mortality with an exposure period of 30 days as was simulated by Henry *et al*. (2012) who used the simple Khoury, Myerscough & Barron (2011) model. Again, adding another stressor (food availability) reduced the resilience to the existing stressor (pesticide). Thus, with poor forage conditions, average colony size was markedly reduced and colonies were lost if exposure leading to doubled forager mortality was repeated year‐on‐year during the most sensitive months. The results of these simulations do not lead to colony losses as rapidly as the simulations by Henry *et al*. (2012), most likely because BEEHAVE captures more of the processes and feedbacks within the colony, so that resilience of the colony emerges in a more biologically realistic fashion. Cresswell and Thompson (2012) find less severe colony effects than Henry *et al*. (2012) while Guez (2013) questions the calculation of the homing failure in Henry *et al*. (2012).

Overall, our results indicate that the effect of stressors such as forager losses, varroa and poor forage can build up over several years, particularly as in these simulations, beekeeping interventions were lacking. While it is immensely challenging to test such interacting stressors in controlled multiyear experiments, the study of interacting stressors is feasible with BEEHAVE. Moreover, our results indicate that the timing of a stressor may be as important as the magnitude of the stressor and that release from one stressor may mitigate the effects of other stressors. Lastly, our results indicate the importance of looking at possible effects affecting the colony repeatedly over several years, which are historically not captured in pesticide risk assessments.

### Synthesis and recommendations

We conclude that BEEHAVE is ready to be used to tackle basic and applied questions regarding honeybees, their functioning and their decline. BEEHAVE was designed to be used by all those who are willing to invest time in understanding the model and the netlogo program and could be a valuable tool for scientists, pesticide regulators, land‐based industries and beekeepers. We chose netlogo because it is freely available, easy to learn and comes with powerful and flexible tools for visualizing model output (including a link to GIS data and R statistical package). The model, its underlying assumptions and its biological basis are fully described using the ODD protocol (Grimm *et al*. 2006, 2010), which is a standard format that can be read by anybody, not just modellers. Moreover, the Supporting Information includes a User Manual and a Guided Tour which should enable non‐modellers to understand how the program works and how it can be used. We are also maintaining a website (http://beehave-model.net/) supporting the use of BEEHAVE. To make sure that all users are working with the same version of BEEHAVE, we also provide means for version control and this will require that publications based on BEEHAVE include evidence that the correct version has been used. This is critical to ensure that results obtained with BEEHAVE can consistently be related to each other. Beehave (2013)©, the implementation of the model BEEHAVE is copyrighted to Matthias Becher and licensed under the GNU General Public License.

There is an urgent practical need for a model to provide biologically realistic predictions of honeybee colony dynamics, growth and survival in complex and changing environmental conditions, so that we can understand and manage the effects of emergent diseases, parasite pressure, changing landscapes and multiple pesticide exposures (Osborne 2012; Becher *et al*. 2013; 2013a, 2013b, 2013c; Vanbergen and the Insect Pollinators Initiative 2013). The tests and applications illustrated here demonstrate that BEEHAVE provides a robust platform, with sufficient complexity to simulate realism, to be developed and used to explore a range of practical management questions, of relevance to beekeepers (e.g. Application 1: acaricide), land managers (e.g. Application 2: forage and varroa) and risk assessors (e.g. Application 3: pesticide exposure and forage). Two further examples of such applications are (i) how colonies respond to different proportions and locations of planted forage mixtures that are used within agri‐environment schemes and (ii) contributing to higher tier risk assessments of agrochemicals (2013a, 2013b, 2013c) using realistic projections of time and space. Such simulation experiments could save substantial time and resources, allowing scientists to focus field experiments on those factors and interactions which seem to be having the strongest effects in the simulations. We therefore recommend that BEEHAVE is used to explore the complex and urgent problems underlying honeybee colony failure and also to find and test alternative management techniques for the landscape and for the colonies themselves to improve their health and survival.

## Supporting information


**Appendix S1.** (JPEbecherSA1_ODD.pdf): Detailed model description, following the ODD (Overview, Design concepts, Details) protocol (Grimm et al. 2006, 2010).

**Appendix S2.** (JPEbecherSA2_Manual.pdf): User Manual and Guided Tour of the BEEHAVE implementation.

**Appendix S3.** (JPEbecherSA3_Beehave2013.nlogo): Beehave (2013)^©^, the implementation of the BEEHAVE model developed in NetLogo (http://ccl.northwestern.edu/netlogo/).

**Appendix S4.** (JPEbecherSA4_Sensitivity.pdf): Complete sensitivity analyses.

**Appendix S5.** (JPEbecherSA7_modBhave‐Pesticide.nlogo): modified version of Beehave, used for the pesticide scenario.

**Appendix S6.** (JPEbecherSA8_modBhave‐SeeleyTest.nlogo): modified version of Beehave, used to simulate Seeley's feeder experiments.

**Appendix S7.** (JPEbecherSA9_modBhave‐Sensitivity.nlogo): modified version of Beehave, used for the sensitivity analyses

**Appendix S8.** (JPEbecherSA10_modBhave‐AFF.nlogo): modified version of Beehave, used to determine life span and age of first foraging.

**Appendix S9.** (JPEbecherSA11_Copyright&Licence.pdf): Beehave (2013)^©^, the implementation of the model BEEHAVE is copyrighted to Matthias Becher and licensed under the GNU General Public License.

**Table S1.** (JPEbecherST5_Variables.xlsx): Scheduling of all processes, lists of global and local variables and further information.

**Table S2.** (JPEbecherST6_Inputfiles.xlsx): Input files for Beehave.
