# Supplementary material for: BEEHAVE: a systems model of honeybee colony dynamics and foraging to explore multifactorial causes of colony failure
Source: J Appl Ecol. 2014 Mar 4;51(2):470–82. doi: 10.1111/1365-2664.12222 (PMC4283046; doi:10.1111/1365-2664.12222)
Supplement: Supplementary file 1 — Appendix S1. (JPEbecherSA1_ODD.pdf): Detailed model description, following the ODD (Overview, Design concepts, Details) protocol (Grimm et al. 2006, 2010). [file JPE-51-470-s001.pdf]

BEEHAVE – Model Description

The model description follows the ODD (Overview, Design concepts, Details) protocol, a standard format for describing individual-based models (Grimm et al. 2006, 2010). Additionally, hyperlinks allow going forth and back between the verbal ODD description and corresponding program code of the model's elements (Adobe Reader shows the previous view with the shortcut "Alt + Left Arrow"). The model was implemented in NetLogo (Wilensky, 1999), version 4.1.3. and transferred to version 5.0.1. The program and a user manual are available at http://beehave-model.net/.

1. PURPOSE

The purpose of the model BEEHAVE is to explore how various stressors, including varroa mites, virus infections, impaired foraging behaviour, changes in landscape structure and dynamics, and pesticides affect, in isolation and combination, the performance and possible decline and collapse of single managed colonies of honey bees.

2. ENTITIES, STATE VARIABLES, AND SCALES

The model consists of three linked modules: a colony, a mite, and a foraging model (Fig. 1). Optionally, an additional, external landscape module can be used to create input files for the foraging module. The colony module (hereafter colony model) describes in-hive processes. It is based on difference equations which generate the colony structure and colony dynamics for the brood, in-hive worker and drone population. The mite module (hereafter mite model) describes the dynamics of a *Varroa* mite population within the honey bee colony. As vectors for viruses, mites affect the mortality of bee pupae and adult bees. Viruses are not implemented as entities but via infection rates of mites and bees.

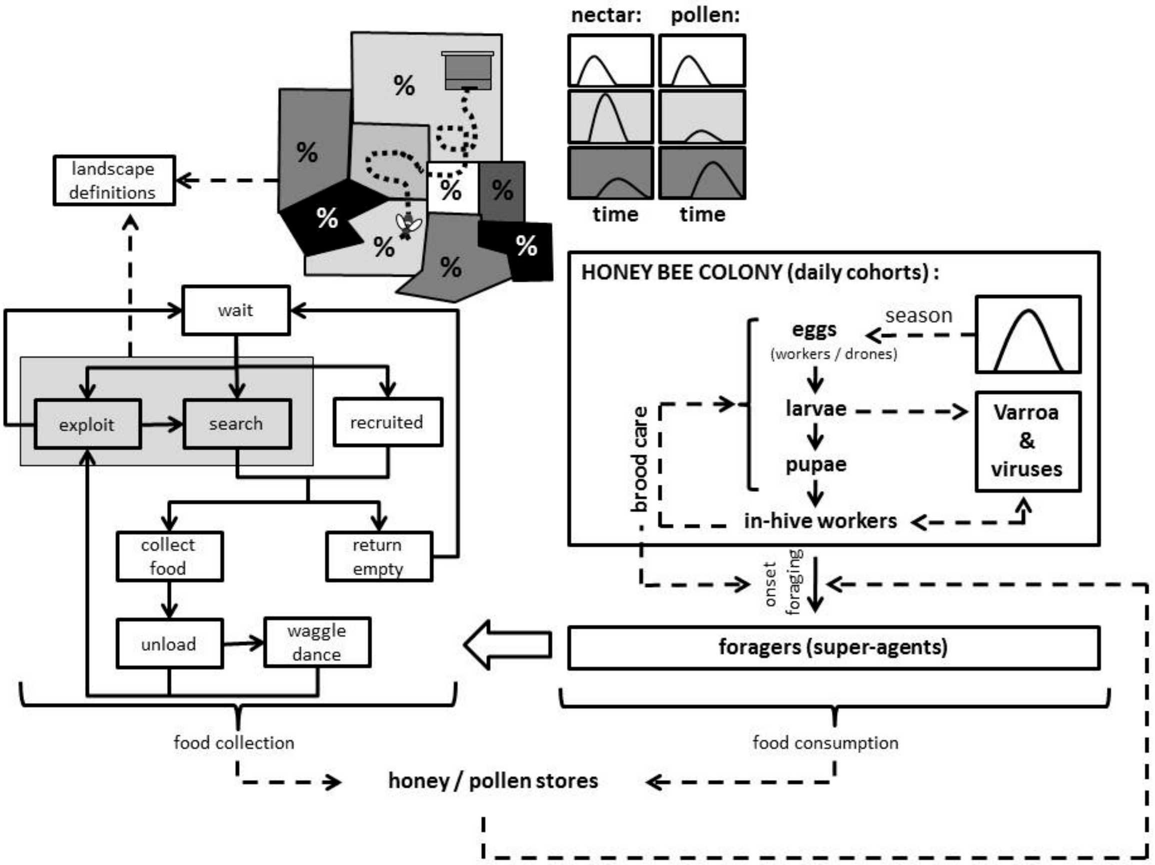

Fig. 1: Simplified overview of the BEEHAVE model structure: Based on the egg laying rate and interacting with the varroa and foraging modules, the structure of a single honey bee colony is modeled. A separate landscape module allows to determine detection probabilities of flower patches and to define their nectar and pollen flows over the season. This information is then taken into account, when foragers collect food in an agent-based foraging module. Note that the various mortalities implemented in the model are not shown in this graph.

The foraging module (hereafter foraging model) is a spatially implicit, individual-based model which represents foraging from flower patches located in the landscape around the hive. Properties of these flower patches, such as probability of being detected by scouting bees, distance to the hive, or nectar and pollen availability, are either set by the modeler when exploring hypothetical landscapes, or extracted from real crop maps using an external landscape module (Fig. 1 and "Input data" below). Landscape dynamics, including dynamic location and availability of crop fields of different types, can be taken into account by updating the imported landscape data every time step of the colony model. The colony and mite model proceed in daily time steps. The foraging model, which is called from the colony model once per day, includes a flexible number of time steps of varying length, which are in the order of magnitude of minutes.

The structure of the model is a compromise between structural realism on the one hand, i.e. the ability to represent heterogeneity where it is likely to matter, and computational efficiency and parsimony regarding parameterisation and model analysis on the other hand. Hence, the in-hive bee population is represented via age cohorts, foraging bees via collectives of individuals, mites via the total number of healthy and virus-infected phoretic mites, and viruses via transmission rates between mites and bees; the model can only consider one virus species at a time.

Conceptually, the model is very simple and includes many simplifying assumptions. Still, in terms of the number of entities, state variables, parameters, and processes, the model is quite complex. It is therefore especially important for understanding the model to get familiar with and fully understand the following Overview part of the ODD model description. Hyperlinks to the corresponding NetLogo code should be used to overcome possible remaining ambiguities of the verbal model description. Hyperlinks from variables and parameters link to an external spreadsheet file that provides an overview of all global and local variables used in the model and NetLogo program.

Colony model

The entities comprising the colony are (1) age classes, or cohorts, of eggs, larvae, pupae and adults, both for in-hive worker bees and drones, (2) pollen and honey stores in the hive, (3) optionally the queen, if queen aging is considered. Bees are distinguished by cohort identity number, age, sex, exposure to varroa mites, and virus infection, plus auxiliary variables that keep track of mortality and infections. Hence, each cohort is characterised by its unique ID number *who*, *age* [days], *number*, which is the number of individuals in the cohort, *ploidy*, which describes the sex of the bee entities (1: haploid drones, 2: diploid workers), *numberDied*, which represents the number of individuals that died within a time step due to cohort-specific mortality, and *invadedByMiteOrganiserID* [id], which refers to the invasion of mites of this bee cohort shortly before their brood cells were capped; likewise, bee cohort IDs (*who*) are used to link bee and mite population dynamics (see mite model). Cohorts of worker and drone pupae and adult drones are additionally characterised by *number_infectedAsPupa* and *number_healthy*, representing the numbers of healthy bees and bees infected as pupae. In-hive bees can also become infected as adults (*number_infectedAsAdult*). Foragers are also involved in the colony model as they contribute to the brood care (defined by the global variable *Forager_Nursing_Contribution*). This is necessary to start raising of brood in spring and reflects the activity of winter bees.

The hive is characterised by the honey and pollen stores, with maximum set for honey stores (*Max_Honey_Store_kg*) and brood space (*Max_Broodcells*). The queen is defined by her egg-laying rate, which can optionally be influenced by the queen's age and by the number of bees available for nursing.

Mite model

The mite model describes the dynamics of a varroa mite population within the honey bee colony. As vectors for viruses (either deformed wing virus, DWV, or acute paralysis virus, APV), mites can affect the mortality of bee pupae and adult bees. The entity of the mite model is the total population of phoretic mites, i.e. mites attached to adult bees, characterised by both the total number of phoretic mites, *PhoreticMites*, and the proportion of healthy phoretic mites, *PhoreticMitesHealthyRate*.

To reproduce, phoretic mites enter drone or worker larvae cells shortly before the cells are capped. Such larvae cells are thus invaded by none or up to eight mites for worker cells and up to 16 mites for drone cells (*Max_Invaded_Mites_Workercells*/*Dronecells*). Knowing how many larvae cells are invaded, on a certain day, by a certain number of mites is important because the number of mites in a pupa cell determines virus transmission from mites to bee pupae and from infected bee pupae to mites, and mite reproduction.

However, in the colony model, bees of the same age and sex are not distinguished individually. Therefore, for mite invasion, suitable larvae cells are temporarily represented individually and it is checked for every individual phoretic mite whether it invades a randomly chosen temporary cell. Then, the auxiliary entity *miteOrganiser* is used to keep

track of all mites that invaded larvae cells in a time step. Thus, a *miteOrganiser*'s state variables are: a list for both worker and drone cells which specifies how many cells were invaded by none or up to [Max_Invaded_Mites_Workercells/Dronecells] mites (*workerCellListCondensed* and *droneCellListCondensed*); the proportion of healthy mites on the day of invasion, *invadedMitesHealthyRate*, which is required to calculate the virus transmission from mites to pupae and back to healthy mites; and the identity of the corresponding drone and worker brood cohort, which are invaded by the miteOrganisers mites (*invadedDroneCohortID* [id] and *invadedWorkerCohortID* [id]), to allow for an interaction of mites and brood.

Foraging model

The foraging model comprises two types of entities, foragers and flower patches. Foragers are in fact "squadrons", or "super-individuals" (Rose et al., 1993; Scheffer et al., 1995; Grimm and Railsback, 2005) representing a given number of identical and identically behaving foragers; for brevity, in the following we refer to these squadrons as foragers. Super-individuals are used to save computation time. The number of individual foragers represented by one super-individual is determined via the parameter Squadron_Size, which is set to 100 in all analyses presented below. Foragers are created from those cohorts of adult in-hive workers that reach the age of first foraging (AFF). Foragers are characterised by the state variables listed in Tab. 1; the variable *activity* describes the current activity of a forager, which is one of seven different activities (Tab. 2).

Flower patches represent flowering patches in the landscape that provide nectar and pollen, for example crop fields or orchards. Since the foraging model is spatially implicit, flower patches do not have a spatial extent or shape, but are characterised by the the state variables listed in Tab. 3. The variables *xcorMap*, *ycorMap* and *size_sqm* are only used to visualise the landscape in the 'foraging map' plot.

Tab. 1. State variables of foragers.

**State variable**	**Definition [unit/possible values]**	**Comment**
age	Age [days]	
activity	The current activity (Tab. 2)	
activityList	Record of all activities performed during a day	used for verification of code
knownNectarPatch	[patch id]	
knownPollenPatch	[patch id]	
pollenForager	[*true*/*false*]	if false, forage nectar
cropEnergyLoad	Energy load of nectar in the honey stomach [kJ]	
collectedPollen	[g]	
infectionState	[*healthy*/ *infectedAsPupa*/*infectedAsAdult*]	
mileometer	Total flight distance [km]	used to determine mortality of foragers
km_today	Summed flight distance during a day [km]	forager stops foraging if km_today > *Max_km_per_day*

Tab. 2: Activities of forager squadrons in the foraging submodel.
Forager activities during a day are stored in the forager's state variable "activityList" (Tab. 1).

**Activity**	**Definition**
lazy	Lazy bees will not forage on that day and cannot be recruited
resting	Resting bees, can be recruited to an advertised flower patch by dancers or may start foraging spontaneously in a following foraging round

expForaging	Experienced foragers, engaged in nectar or pollen collection at a certain patch
searching	Scouts, searching a new flower patch for nectar or pollen
bringingNectar	Successful nectar foragers, bringing back nectar
bringingPollen	Successful pollen foragers, bringing back pollen
recForaging	Foragers, recruited to an advertised flower patch by a dancer. They will search for this patch in the next foraging round

Tab. 3: State variables of flower patches.
Variables in parentheses are auxiliary variables, which can be derived from the state variables but were, for convenience, implemented as state variables.

**Variable**	**Definition**
patchType	Habitat type of the flower patch, e.g. "oilseed rape"
distanceToColony	Distance from the patch to the colony [m]
xcorMap, ycorMap	Coordinates of patch centre (used for visualisation in 'foraging map' plot)
size_sqm	Size of the patch [m^2] (used for visualisation in 'foraging map' plot
quantityMyl	Quantity of available nectar [µl]
nectarConcFlowerPatch	Sugar concentration of provided nectar [mol/l]
amountPollen_g	Quantity of available pollen [g]
detectionProbability	Probability that a scout finds the flower patch.
handlingTimeNectar handlingTimePollen	Handling times based on depletion of nectar/pollen at patch
(flightCostsNectar, flightCostsPollen)	(Energetic costs calculated from trip length and handling time)
(eef)	(Energetic efficiency calculated from nectar concentration and costs)
(danceCircuits)	(Calculated from EEF, following Seeley 1994)
(danceFollowersNectar)	(Number of possible recruits for nectar patches, calculated from danceCircuits)
(summedVisitors)	(Number of visits by forager squadrons)
(nectarVisitsToday, pollenVisitsToday)	(Number of nectar/pollen visits on current day)
(tripDurationNectar)	(Duration of trip [s] calculated from distance and handlingTimeNectar)
(tripDurationPollen)	(Duration of trip [s] calculated from distance and handlingTimePollen)
(mortalityRisk)	(Risk to die during nectar foraging trip, calculated from trip length and handlingTimeNectar)
(mortalityRiskPollen)	(Risk of dying during pollen foraging trip, calculated from trip length and handlingTimePollen)

3. PROCESS OVERVIEW AND SCHEDULING

Here we describe all processes represented in the model and by which entity or procedure (i.e. a subroutine defined in the source code) they are called within the program. Some processes are called at various places in the program; the appendix S5 (worksheet "Scheduling") provides an overview of all procedures, including which other procedure they are calling.

Colony model scheduling

Every time step, i.e. every day, the colony model’s processes are simulated in the order given in Tab. 4. Variables are updated immediately. The sequence by which entities are processed is, if not specified otherwise, randomised each time a set of entities runs a certain submodel.

Development follows the stages eggs, larvae, pupae, adults (both for workers or drones). Newly emerged workers become in-hive bees (IH-bees), which develop into foragers at the age of first foraging (*AFF*), which depends on the ratio of the numbers of brood and in-hive bees, total honey and pollen stores, protein content of jelly and the in-hive bees to foragers ratio.

Mortalities are based on Poisson-distributed probabilities, specified for each developmental stage and depending on potential virus infection. Additionally, mortality of brood depends on the number of in-hive bees available for nursing and the protein content of jelly fed by nurse bees. Drones and foragers have a maximum age, and foragers are also assumed to have a high mortality risk during their foraging activities and they will die when their total flight distance exceeds 800 km.

Simulations stop, (i.e. colony "dies") if honey stores are exhausted, if colony size at the end of a year is below a critical threshold (*Critical_Colony_Size_Winter*), or if no more bees are alive.

Table 4: Scheduling of the colony model's processes.
The submodels, or procedures, implementing these processes are described in detail in Section 7 ("Details"). The scheduling of the mite and foraging models are described below. For each submodel, the entities performing the corresponding task are specified (note that the "observer" refers to the calling program, or programmer). Submodels in which entities are processed in a certain order instead of randomly are marked with an asterisk. Submodels which are optional, i.e. can be deactivated by the user, are marked with "+/-". (See also NetLogo code and the overview of NetLogo procedures provided in appendix S5 (worksheet "Procedures")).

**Process**	**Description [entity/entities running the submodel]**
DailyUpdateProc	Resetting of parameters once every time step; needed because some model parameters change during simulations [observer].
SeasonProc_HoPoMo	Day of year determines a seasonal factor (*HoPoMo_seasont*) that influences egg laying rate [observer].
WorkerEggsDevProc	Ageing by one day, then reduction due to mortality; oldest cohort is removed when it develops into worker larvae [worker egg cohorts].
DroneEggsDevProc	Ageing by one day, then reduction due to mortality; oldest cohort is removed when it develops into drone larvae [drone egg cohorts].
NewEggsProc	Determines number of newly laid worker and drone eggs [observer].
SwarmingProc +/-	Calculates day of swarming, removal of parts of the adult bee population, phoretic mites and stores [observer].
WorkerEggLayingProc	New worker egg cohorts are created.
DroneEggLayingProc	New drone egg cohorts are created.
WorkerLarvaeDevProc	Ageing by one day, then reduction due to mortality; oldest cohort is removed when it develops into worker pupae [worker larvae cohorts].
DroneLarvaeDevProc	Ageing by one day, then reduction due to mortality; oldest cohort is removed when it develops into drone pupae [drone larvae cohorts].
NewWorkerLarvaeProc	Worker eggs developed into larvae are now created as larvae [observer].
NewDroneLarvaeProc	Drone eggs developed into larvae are now created as larvae [observer].
WorkerPupaeDevProc	Ageing by one day, then reduction due to mortality; oldest cohort is removed when larvae emerge and develop into in-hive bees [worker pupae cohorts].
DronePupaeDevProc	Ageing by one day, then reduction due to mortality; oldest cohort is removed when larvae emerge and develop into adult drones [drone pupae cohorts].
NewWorkerPupaeProc	Worker larvae developed into pupae are now created as pupae [observer].
NewDronePupaeProc	Drone larvae developed into pupae are now created as pupae [observer].
WorkerIHbeesDevProc*	Ageing by one day, then reduction due to mortality; oldest cohort is removed when in-hive bees develop into foragers. Entities are processed in order of their age for a correct transition of in-hive bees to foragers [in-hive bee cohorts].
DronesDevProc	Ageing by one day, then reduction due to mortality; oldest cohort dies when reaching *Drone_Lifespan* [drone cohorts].

BroodCareProc*	Brood may starve/freeze, if ratio of brood to nursing bees is too high. Entities are processed in reversed order of their age as death of brood affects more likely younger than older brood [observer].
NewIHbeesProc	Pupae developed into in-hive bees are created as in-hive bees, age is reset to 0 (thus, for adults age refers to their actual lifetime as adults) [observer].
NewDronesProc	Pupae developed into adult drones are created as adult drones, age is reset to 0 (thus, for adults age refers to their actual lifetime as adults) [observer].
MiteProc +/-	Run *mite* model (if mites exist) [observer].
BeekeepingProc +/-	Beekeeping activities: honey harvest, varroa treatment, queen replacement [observer].
DrawIHcohortsProc	Number of bees in IH cohorts (workers & drones, brood & adults) is visualised via coloured bars in NetLogo's "View" [all worker and drone cohort of all stages].
Start_IBM_ForagingProc	Run *foraging* model (if foragers exist) [observer].
CountingProc (also called by BroodCareProc, MiteProc)	Count all entities (separately) [observer].
PollenConsumptionProc	Calculate pollen requirements on basis of number of individuals in the different cohorts times their pollen consumption rates [observer].
HoneyConsumptionProc	Calculate need of honey on basis of number individuals in the different cohorts times their honey consumption rates [observer].
DoPlotsProc	Update all plots [observer].

Mite model scheduling

The mite model is called once each time step (Tab. 4), but its submodel MiteReleaseProc might be called, whenever bees emerge from brood cells or larval or pupal brood dies, i.e. in all submodels of the colony model including "Dev" for development in their name, and in the submodel BroodCareProc (for details, see Section 7, "Details"; for an overview of submodels and in which parts of the model they are called, see appendix S5 (worksheet "Scheduling")). The scheduling of the mite model is listed in Tab. 5.

Tab. 5: Scheduling of the mite model.
(All processes are called by the Netlogo "observer".)

Process	Description
CreateMiteOrganisersProc	Create every, time step, an entity called "miteOrganiser", which keeps track of all mites that invaded larvae cells in this time step
CountingProc	Count all honey bee entities of the colony model.
MitesInvasionProc	Determine the number of mites invading drone and worker larvae cells in this time step.
MitePhoreticPhaseProc	Calculate number of newly infected phoretic mites and newly infected in-hive workers.
MiteDailyMortalityProc	Determine mortality of the phoretic mites.
MiteOrganisersUpdateProc	Count mites, actualise miteOrganisers (age, xy-position in visualisation).

Foraging model scheduling

The foraging model is called once each time step by Start_IBM_ForagingProc (Tab. 4). Its submodels are listed in Tab. 6 and processed in the given order. Procedures with other than randomised processing are marked with an asterisk (*).

Tab. 6. Scheduling of the processes of the foraging model.

Process	Description
ForagersDevelopmentProc	Ageing of forager (squadrons) by one day.
NewForagersProc	In-hive bees that were determined to develop into foragers are created as forager squadrons.
ForagingRoundProc	Runs the submodels representing the actual foraging activity ("foraging rounds"; Table 7). This submodel is only called during the foraging season, defined by *Season_Start* and *Season_Stop*, and if *honeyEnergyStore* < 0.95 * *Max_Honey_Energy_Store*, i.e. the honey stores are not almost filled.
WriteToFileProc*	Results can be written in an output file. Entities are processed in order of their ID for ease of presentation.
ForagersLifespanProc	Foragers may die due to age, total flight distance, or background mortality.

Foraging is based on repeated 'foraging rounds'. The submodels of a single foraging round are processed in the order given in Tab. 7; state variables are updated immediately; foragers are, in each submodel, processed in a randomised sequence.

Tab. 7. Scheduling of the processes run by ForagingRoundProc.

Process	Description
FlowerPatchesUpdateProc	Updates handling times, energetic efficiency, flight costs, trip duration, foraging mortality rate and dance circuits
Foraging_start-stopProc	Foragers become pollen foragers with a probability of ProbPollenCollection, else they stay nectar foragers. Active foragers quit foraging with a probability of *Foraging_Stop_Prob*; foragers abandon their current patch with a probability dependent on the energy efficiency (*Eef*) or of the duration of a foraging trip; resting foragers start foraging with a probability of *ForagingSpontaneousProb*; active foragers that are not experienced or have abandoned their patch search for a patch.
Foraging_searchingProc	Searching foragers may find a flower patch.
Foraging_collectNectarPollenProc	Foragers arriving at a patch fill their crop with nectar or collect pollen.
Foraging_flightCosts_flightTimeProc	Calculate energy and time spent on the foraging trip.
Foraging_mortalityProc	Active foragers may die, risk depends on trip duration.
Foraging_dancingProc	Successful foragers may dance, depending on the energetic efficiency of the flower patch.
Foraging_unloadingProc	Successful foragers unload their crop and increase the colony's honey store.

4. DESIGN CONCEPTS

Basic principles: The colony dynamic is based on an age cohort model with fixed periods for each developmental stage and specific mortalities. Evaluation of flower patches in the foraging model is based on the energetic efficiency for foraging at this patch, following Seeley (1994). Resilience mechanisms are included via numerous feedbacks, i.e. the change of state variables is affected by the values of other state variables. Some key processes are driven by first principles, i.e. the collection and consumption of energy (nectar) and pollen.

Emergence: Many processes are imposed, for example development times and many mortality rates, but others emerge from lower-level processes, for example egg laying rate, which is partly imposed but also driven by a feedback from the number of nursing bees. The use of

flower patches by the colony emerges from the foraging behaviour of its foragers, i.e. their interaction with flower patches and with each other.

Adaption: The colony and mite models do not include any adaptive decision making, because they are not individual-based. In the foraging model, foragers prefer flower patches with higher energetic efficiency (*Eef*, i.e. (energy gain - energy costs) / energy costs). High *Eef* is also necessary to elicit dancing in successful foragers.

Sensing: Entities do not possess any senses per se, however, sensing is implicitly involved in some processes and decision making: Workers sense the colonies food stores and the numbers of nurse bees and brood, which affects their age of first foraging. Successful foragers sense the energetic efficiency of a flower patch. If they dance, the *Eef* they sensed can be sensed, in turn, by dance following bees.

Interaction: Foragers interact with food patches by exploiting them. Dancing bees communicate the quality and location of a food patch to dance followers. Furthermore, all members of the colony interact indirectly with each other, as they contribute to or consume from common food stores. Additionally, in-hive bees take care for the brood. Also *Varroa* mites and bees interact by transferring viruses to each other.

Stochasticity: The colony submodel contains random mortality, based on a Poisson distribution. Probabilities are also used in the foraging submodel to determine the spontaneous foraging activity, success of finding a flower patch and the foraging mortality. Distribution of varroa mites on brood cells, mortality of phoretic mites and transmission of viruses among entities is also based on random assignments. In all these cases, stochasticity is used to represent variability which exists in the real system. To avoid artificial effects from always processing certain sets of entities in the same sequence, the processing sequence of entities is randomised, if not stated otherwise (Tables 3-6).

Collectives: The entire colony, comprising brood, in-hive bees and foragers, is a collective ensuring and supporting the single reproductive unit of the colony, the queen. For run-time reasons, foragers are grouped into collectives, “squadrons”, which behave as one unit, representing, in the default settings, one hundred foraging bees which are identical and do exactly the same; to test for artefacts of this super-individual approach, squadron size was varied, including each squadron representing only one individual, i.e. a truly individual-based setting.

Observation: The model is very rich in structure and thus produces, in each time step, vast amounts of data, which therefore need to be aggregated into summary statistics, for example colony size (adult bees), egg-laying rate, or age at first foraging (*Aff*). These data are recorded at the end of each time step. The output is shown in diagrams, representing the colony structure (# individuals in each developmental stage and in each age cohort for workers and drones), amount of stored honey, mean duration of foraging trip and nectar availability in the flower patches, travelled distance [km], age of first foraging & daily lifespan, # dead bees, # varroa mites, including infection states of mites and bees. However, for testing purposes a larger number of options exist and have been used; see also the description of the BEEHAVE user interface in the User Manual (appendix S2) and appendix S5 (worksheet "Plots") for a complete list. The NetLogo code also includes a large number of test code checking whether certain state variables are within their reasonable ranges; if not, message will pop up and inform the user that something went

wrong with a certain state variable. This test code is permanently active; list of checks included in the code is provided in the appendix S5 (worksheet "Assertions").

5. INITIALISATION

Simulations start on the 1st January with a colony size of *N_Initial_Bees*, all of them foragers with an average age of 130 days. We did not specifically implement the long living "winter bees" of real colonies, but longevity of foragers in the model is a consequence of reduced foraging activity in autumn and winter. To allow the production of a first in-hive bee generation however, foragers contribute to the brood care (*Forager_Nursing_Contribution*). When using the term "worker" we refer to all foragers and in-hive bees. "Adult" bees comprise foragers, in-hive bees and adult drones. "Nurse" bees are workers that contribute to brood care, i.e. in-hive bees and foragers times *Forager_Nursing_Contribution*.
Some of the parameters can be chosen on the user interface, like distance of two flower patches to the colony, their detection probabilities, nectar concentrations, nectar quantities, and the amount of available pollen but also beekeeping activities or varroa infestation and virus infection. We also provide a default parameter setting as well as some adaptions to simulate for example scenarios with the varroa mite, in more realistic landscapes or with only a feeder station providing nectar and pollen. The parameterisations for all these scenarios as well as the initial values of all local and global variables are listed in the appendix S5 (worksheets "Initialisation buttons", "Global variables" and "Local variables") .

The procedures involved in the initialisation process are:

- Setup
- ReadFileProc
- ParameterizationProc
- CreateFlowerPatchesProc
- Create_Read-in_FlowerPatchesProc
- CreateImagesProc

In the Setup procedure, called by the "Setup"-button, the model world is reset, i.e. all bee-related entities are deleted and all other global variables are set to zero, except those defined on the Interface ("clear-all"). If an experimental setup is defined via the Netlogo "chooser" *Experiment*, the initial numbers of bees and mites etc. are defined, according to the experimental conditions defined in the procedure GoTreatmentProc.

If *ReadInfile* = *true*, then the ReadFileProc is called and data of the *Input_File* are read and saved in the list *AllDaysAllPatchesList*.

In the ParameterizationProc, all globals variables which are not defined via an input field on the user interface are set to their initial value (if other than zero).

Then the flower patches are defined, either in the procedure CreateFlowerPatchesProc (if *ReadInfile* = *false*) or in the procedure Create_Read-in_FlowerPatchesProc (if *ReadInfile* = *true*), in both cases followed by FlowerPatchesUpdateProc.

CreateFlowerPatchesProc and Create_Read-in_FlowerPatchesProc both define those state variables of the flower patches, which will not change during a simulation run, like *distanceToColony* or *nectarConcFlowerPatch*.

Finally, CreateImagesProc is called, which creates a range of symbols like "sun", "cloud", "varroa mite" etc. which are used on the interface to inform the user about weather, colony state, beekeeping activities etc.

6. INPUT DATA

As an alternative to input the flower patch parameters via the user interface, it is also possible to read in a text file. Optionally, the input file can be created by an external landscape module. It provides information about the number of available patches, their distance to the colony, the detection probability, the amount of nectar and pollen they offer on each day of the year and the handling times of collecting a nectar or pollen load.
The file consists of a headline and 365 x (# flower patches) lines, defining the food availability of each flower patch on each day of one year.
The 15 data columns are: 1. day-of-year 2. ID of the flower patch 3. old ID of the flower patch (as it was determined in the landscape module) 4. type of the flower patch 5. distance of the flower patch to the colony 6,7: x- and y coordinates of the patch 8. size of the patch [m^2] 9. amount of pollen [g] available on this day 10. sugar concentration of the nectar [mol/l] 11. amount of nectar [l] available on this day 12. the calculated detection probability of the patch. 13. the modelled detection probability of the patch 14. the time to gather a nectar load and 15. the time to gather a pollen load.
In the default parameterisation, "old ID" is identical to "ID". However, there is an option in the external landscape module, which allows neglection of flower patches further away from the colony than a threshold distance. If this option is chosen, the "old ID" may differ from "ID". "Old ID" is not used by the program but is only stored as additional information for the user.
Also the x-, and y-coordinates as well as the size of the patch are only for the visual representation of the patch in the "foraging map" plot.
The detection probabilities relate to the likelihood of a scout (searching for a new food patch) finding this particular patch. They are determined in the external landscape module, either by an spatially explicit, individual based model ("modelled detection probability"), or calculated on the basis of the size of a flower patch and its distance from the colony ("calculated detection probability"). Per default, the calculated detection probability is used by the model.

7. SUBMODELS

The three modules of BEEHAVE include a large number of submodels, and some of them are calling subprograms or other submodels. To facilitate understanding of the submodel descriptions while keeping an overview of the entire model, we structured the descriptions as follows:

- Each description of a complex submodel starts with a description of its rationale and biological background.
- This is followed by a verbal description of the model, augmented by lines of program code that implement equations or certain logical conditions; hyperlinks lead to the corresponding line of the code or, for global variables (italic, with capital first letter), to a table listing all global variables, their default or initial value and additional information; there are no “back” links for these hyperlinks (but a Back button can be

inserted in the Word Ribbon as explain on page 1 or use the shortcut "Alt + Left Arrow" in the Adobe Reader). The verbal description is not always complete, because, for example, listing each single variable that is updated in a certain submodel, would be cumbersome. Thus, for each submodel we include hyperlinks to the submodels' corresponding NetLogo code, which has a simple syntax and uses self-explaining variable names, so it can also be understood also by non-programmers. The program code also includes numerous comments, which provide additional information and explanation; thus, readers should switch forth and back between this ODD model description and the code.

When variables are described for the first time, they are linked to a list of all global variables, where their initial value, units and comments are given.

I - THE COLONY MODEL

7.1. Time step

Procedures involved:

- StartProc
- Go
- GoTreatmentProc

The StartProc is called by the various run buttons, either a single time ("1 Day"), repeatedly ("1 Month", "1 Year", "run X days") or continually ("RUN"). It stops the program, if the debugger variable *BugAlarm* is *true* or otherwise calls the *Go* procedure and optionally calls the WriteToFileProc procedure (section GUI & Output).

The Go procedure is called by the *StartProc*. It increments the time step, representing one day, and calls all procedures that describe the models schedule, i.e. all processes of a day.

7.2. Updates

Procedures involved:

- CountingProc
- DailyUpdateProc

The CountingProc procedure counts the number of individuals for all developmental stages in the worker and drone caste by summing up the number of bees in the relevant cohorts. For example, the total number of in-hive bees (*TotalIHbees*) is calculated as:

```
set TotalIHbees 0
ask IHbeeCohorts [ set TotalIHbees (TotalIHbees + number)]
```

The number of phoretic mites is determined as well. If any inconsistencies are detected in these numbers, for example negative numbers within any cohort, the debugger variable *BugAlarm* is set *true* and the program stops.

The DailyUpdateProc procedure updates those state variables that can change every day. Variables that depend on the current date, for example *DailyForagingPeriod*, are calculated. The description of Foraging_PeriodREP, where *DailyForagingPeriod* is calculated, is given in the section "Foraging model". Variables counting events on that day (like

NectarFlightsToday or *DailyMiteFall*) are set to their default value (usually 0). Flower patches are replenished and drawings of the hive and the flower patches in the Netlogo "world" are refreshed. The age of the queen (*QueenAge*) is increased by one day.

At the end of each year (Day = 365), it is checked if the colony survives the winter and a message in the output field informs the user about the colony size and the honey stores.

7.3. Egg laying

Rationale: Egg laying

Egg laying is a crucial process as it determines in large part the colony dynamics. The egg laying rate of the queen is not only influenced by seasonal factors, but also by the activities of worker bees, who are responsible for feeding the queen and preparing the brood cells (Allen 1960; Free & Williams 1972; Harbo 1986).

DeGrandi-Hoffman et al. (1989) provide an egg laying procedure in their Beepop model, which is also used by Martin (2001), where the daily number of eggs laid depends on the age of the queen, the degree days, day length and worker population. However, a justification of their model is not given. Schmickl & Crailsheim (2007) propose a bell-shaped curve reflecting the seasonal egg laying pattern and add random variability as well as a gradual reduction of the egg laying rate if brood space is a limiting factor. The shape of their curve can be adjusted to account for regional differences in the egg laying.

Egg laying in the BEEHAVE model is based on Schmickl & Crailsheim's (2007) bell shaped curve, but does not add random variation. The maximum size of the brood nest can be defined, but as long as there are empty cells available, the egg laying rate is unaffected. To avoid loss of brood due to a lack of in-hive bees (see 7.5. Brood care), the egg laying rate can optionally be limited by the number of bees available for brood care.

Procedures involved:

- NewEggsProc
- WorkerEggLayingProc
- DroneEggLayingProc

We mainly follow Schmickl & Crailsheim (2007) using their factor "season(t)" to create a bell-shaped egg laying curve, however without adding random variability (NewEggsProc):

```
let ELRt_HoPoMo (MAX_EGG_LAYING * (1 - HoPoMo_seasont))
```

with *HoPoMo_seasont* either being

```
seas1 (1 - (1 / (1 + x1 * e ^ (-2 * today / x2))))
```

or

```
seas2 (1 / (1 + x3 * e ^ (-2 * (today - x4) / x5)))
```

whichever is larger.

If *EggLaying_IH* is *true*, then the egg laying rate can also be limited by an insufficient number of nurse bees, which may reflect a reduced number of prepared brood cells, less frequent feeding of the queen or removal of eggs at a very early stage:

```
let ELRt_IH (TotalIHbees + TotalForagers * FORAGER_NURSING_CONTRIBUTION) * MAX_BROOD_NURSE_RATIO / EMERGING_AGE
```

The *ELRt_IH* reflects the total amount of brood that can be cared for on a particular day divided by the total duration of the brood period. A reduced egg laying rate can help avoid severe losses of older brood stages, which may occur as a consequence of a lack of nurse bees relative to the amount of brood. The actual number of eggs laid equals either *ELRt_HoPoMo* or *ELRt_IH*, whichever is smaller and can never be larger than *Max_Egg_Laying*. Egg laying is interrupted, if no more empty brood cells are available. However, this will not happen in the default setting, where *Max_Broodcells* is set to an extremely high value. More details on raising brood are given in the section "Brood care".

The number of drone eggs is calculated as a proportion of the number of worker eggs:

```
set NewDroneEggs floor(NewWorkerEggs * DRONE_EGGS_PROPORTION)
```

Drones are only produced during a certain period in summer, defined by *Drone_Egglaying_Start* and *Drone_Egglaying_Stop*.

If ageing of the queen is taken into account (*QueenAgeing* = *true*), the number of worker eggs declines with the queen's age (*QueenAge*) following the equation of DeGrandi_Hoffman et al. 1989. However, queen age does not affect the number of drone eggs so the proportion of drone brood increases in older queens.

Very young queens (<= 10 days) do not lay eggs, irrespective of whether *QueenAgeing* is *true* or *false*. There is no queen mortality and queen replacement only takes place as part of swarming or beekeeping processes.

7.4. Brood development

Rationale: Brood development

Worker and drone larvae hatch ca. 3 days after the egg was laid. Worker larvae cells are sealed after ca. 9 days and drone cells after ca. 10 days. The total developmental time for workers is ca. 21 days and for drones 24 days (reviewed by Jay 1963, Winston 1987).

Schmickl and Crailsheim (2007) refer to Fukuda and Sakagami (1968) to model the duration of their immature worker stages with 3 days for eggs, 5 days for larvae and another 12 days for pupae.

In the BEEHAVE model, worker larvae hatch after 3 days, cap their cells after 9 days and emerge after 21 days. Drone larvae hatch after 3 days, cap their cells after 10 days and emerge after 24 days.

Procedures involved:

- DroneEggsDevProc
- DronePupaeDevProc
- NewDroneLarvaeProc
- NewDronePupaeProc
- NewWorkerLarvaeProc
- NewWorkerPupaeProc

- WorkerEggsDevProc
- WorkerPupaeDevProc

The brood development procedures are similar to each other and describe the ageing of the bee cohorts by one day, the reduction of the numbers of individuals due to the daily, cohort-specific mortality and the development of the last cohort of a developmental stage into the first cohort of the next stage. The procedures are scheduled so the existing cohorts age by one day first and only then are the new cohorts created. Otherwise, freshly created cohorts would age by two days. The egg laying is described in the section "Egg laying", whereas the development of in-hive bees into foragers is described in the section "Transition to foragers". The principle of these procedures is shown in the example of the worker larvae:

In the WorkerLarvaeDevProc, all worker larvae cohorts are handled. The cohorts age by one day. The graphic representation of in the Netlogo "world" moves one step down. Then the number of larvae which died during the last day is calculated:

```
set numberDied random-poisson (number * MORTALITY_LARVAE)
```

The Netlogo reporter "random-poisson" reports a Poisson-distributed random integer, where a mean value is used as input variable. This mean number of deaths is calculated by the cohort size *number* and the mortality rate *Mortality_Larvae*. The result of this process is the actual number of deaths on that day within the cohort. The cohort size is then reduced by the number of deaths.

If larvae have died, the procedure MitesReleaseProc is called to determine the number of varroa mites that are released from the dead larval cells (for details, see varroa model). This process is only conducted if varroa mites are present in the colony and for those cohorts, which might be invaded by varroa mites.

Finally, if the oldest larvae cohort reaches the age *Pupation_Age*, then the global variable *NewWorkerPupae* saves the number of individuals in this cohort, *SaveWhoWorkerLarvaeToPupae* saves the ID ("who") of the worker larvae cohort and *SaveInvadedMOWorkerLarvaeToPupae* saves the ID of the "mite organiser" (*invadedByMiteOrganiserID*) which is related to this specific bee cohort (for details, see varroa model). Then the Netlogo "turtle" representing this cohort dies, i.e. the cohort is deleted.

It is necessary to save these values as global variables, as a new Netlogo "turtle" *pupaeCohort* is later created with an automatically set ID ("who"). This new cohort will then get *NewWorkerPupae* as its cohort size and it has to be reconnected to the corresponding "mite organiser" using *SaveInvadedMOWorkerLarvaeToPupae* (see below in this section).

After the worker larvae have aged by one day, the youngest worker larvae cohort is created, which represents those larvae that hatched from worker eggs during the last day (procedure NewWorkerLarvaeProc). A single cohort is created with *NewWorkerLarvae* as its cohort size. The age of the larvae is the *Hatching_Age*, the *ploidy* is two (diploid) and, for visualisation, the shape of the corresponding Netlogo turtle, its colour and position in the Netlogo "world" are set.

Based on the values of oldest larvae cohort, the youngest pupae cohort is created in the NewWorkerPupaeProc. This procedure is largely equivalent to NewWorkerLarvaeProc,

except that pupae cohorts have to be connected to their corresponding "mite organiser", which is an entity that keeps track of the number and the distribution of mites that invaded brood cells. Hence, the pupae cohort variable *invadedByMiteOrganiserID* is set to *SaveInvadedMOWorkerLarvaeToPupae* to record, which "mite organiser" contains the distribution of mites in the current cohort. And for this very "mite organiser" *invadedWorkerCohortID* is set to *saveWho*, where *saveWho* represents the ID of the pupae cohort, invaded by the mites of this "mite organiser". Thus, both entities (the "mite organiser" and the pupae cohort) are unambiguously linked to each other.

For example, mites of the *miteOrganiser* #1 invade cells of the *larvaeCohort* #2, so the miteOrganiser's *invadedWorkerCohortID* is 2 and the *invadedByMiteOrganiserID* of the *larvaeCohort* is 1. The next day, these larvae develop into pupae. Due to the way this process is implemented, the larvaeCohort #2 is deleted and a new, pupaeCohort has to be created, e.g. as #3. *InvadedWorkerCohortID* of the *miteOrganiser* #1 is then changed to 3 (using *SaveWhoWorkerLarvaeToPupae* and *saveWho*) and *invadedByMiteOrganiserID* of the *pupaeCohort* #3 is set to 1 (using *SaveInvadedMOWorkerLarvaeToPupae*).
In principle, the same processes take place in the drone brood procedures.

7.5. Brood care

Rationale: brood care

Brood care mainly involves two processes, feeding of larvae and thermoregulation of the brood nest. Larvae are primarily fed with protein-rich jelly produced by nurse bees or winter bees (Winston 1987, Deseyn & Billen 2005, Münch & Amdam 2010). If nursing bees themselves lack proteinaceous food, they can continue to successfully feed larvae for only about one week (Crailsheim 1990). Lack of pollen results in removal and cannibalisation of younger larvae by workers (Schmickl & Crailsheim 2001).
Not only larvae but also eggs and pupae require brood care as they rely on developmental temperatures of about 35°C, otherwise brood dies or they will suffer from morphological deficits, once they developed into adults (Himmer 1927, Groh et al. 2004).

Only few models explicitly take brood care into account. Schmickl & Crailsheim (2007) differentiate the need of nursing for various brood stages and also include pollen consumption of larvae and cannibalism, if there is a lack of nurse bees or pollen. Becher et al. (2010) model the thermoregulation of the brood nest, where brood can die, if temperatures are too low.

In the BEEHAVE model, in-hive bees (but to a small degree also foragers) are responsible for brood care. If not enough adult bees for brood the brood care are available, brood dies, starting with the youngest cohorts. Lack of pollen results in the death of eggs and larvae, which represents cannibalisation.

Procedures involved:

- BroodCareProc

In addition to the daily, stage specific background mortality, brood can also die, if an insufficient number of adult workers are present to maintain the brood nest temperature or if larvae lack proteinaceous food.

ExcessBrood represents the total amount of brood lost during a day and is calculated as

```
set ExcessBrood ceiling (TotalWorkerAndDroneBrood - (TotalIHbees + TotalForagers *
FORAGER_NURSING_CONTRIBUTION) *  MAX_BROOD_NURSE_RATIO
```

where *TotalWorkerAndDroneBrood* comprises all egg, larval and pupal stages of worker and drone cohorts. All in-hive bees are assumed to be capable to care for brood and the *Forager_Nursing_Contribution* (set to 0.2) allows also foragers to take part in brood rearing. Otherwise, brood could not be produced in spring, when no in-hive bees are present. *Max_Brood_Nurse_Ratio* is set to 3, and also used in other parts of the model, whenever the ratio of brood to nursing potential of the colony matters, i.e. in the procedures NewEggsProc, AffProc, PollenConsumptionProc, HoneyConsumptionProc and GenericPlottingProc. The NetLogo command “ceiling” rounds floating point numbers to the next largest integer.

For example, if 300 in-hive bees and 100 foragers are present in the colony, then the brood nest could have up to 3 * (300 + 100 * 0.2) = 960 cells. If 1000 brood cells were present of which 30 were one day old drone eggs and another 30 two days old drone eggs, then all one day old drone eggs and ten of the two days old drone eggs would die.

If the colony lacks pollen, then brood will also die:

```
let starvedBrood ceiling ((TotalDroneLarvae + TotalLarvae) * (1 - ProteinFactorNurses))
```

The *ProteinFactorNurses* ranges from 1 to 0 and reflects the relative protein content of the brood food (more details in the "Consumption" section). If the lack of pollen has a stronger impact on brood mortality than the lack of nurse bees, then *ExcessBrood* is set to *starvedBrood*. The according number of brood is then removed from the brood cohorts in the following order: 1. drone eggs 2. drone larvae 3. worker eggs 4. worker larvae 5. drone pupae 6. worker pupae, with the youngest cohorts always dying first. If lack of pollen is the reason for brood removal, then only larvae die, starting with young drone larvae.

7.6. Development adults

Rationale: Development adults

Fukuda and Sakagami (1968) recorded the survival of honey bee worker brood. They found the following survival/stage function: of 100.0 eggs 94.2 developed into unsealed brood of which 86.4 became sealed brood, which finally resulted in 85.1 adults.
Fukuda and Ohtani (1977) present survival curves for adult drones and drone brood. The survival/stage function was: 100 eggs, 82 unsealed brood, 60 sealed brood and 56 adults.

Schmickl and Crailsheim (2007) refer to Fukuda and Sakagami (1968) to calculate their daily mortality rates of immature worker stages: 0.03 for eggs, 0.01 for larvae and 0.001 for pupae. For adult bees they use values between 0.005 and 0.035, depending on the bees' activity.
Martin (2001) also uses the data of Fukuda and Sakagami (1968) to calculate brood survival, but additionally takes the colony size into account (referring to Harbo 1986). Mortality rates of healthy and infected adult workers are directly taken from survivorship curves, based on literature and Martin's own data.

We use the same mortality rates as Schmickl and Crailsheim (2007) for the worker brood and calculated the mortality rates for drones from Fukuda and Ohtani (1977). Our mortality rates for healthy and infected workers were derived from Martin's (2001) survivorship curves for winter, as these are not affected by foraging. We have chosen our values in such a way that 50% of an initial population is dead after the same period as in Martin's curves, which results in mortality rates of 0.004 for in-hive mortality of healthy workers and 0.012 for in-hive mortality of DWV-infected workers.

Procedures involved:

- DronesDevProc
- NewDronesProc
- NewIHbeesProc
- WorkerIHbeesDevProc

Similar to the development of the brood, also the adult drones and in-hive workers are implemented in cohorts that age on a daily basis and that are exposed to a stage specific mortality (*Mortality_Drones*, *Mortality_Drones_Infected_As_Pupae*, *Mortality_Inhive*, *Mortality_Inhive_Infected_as_Pupa*, *Mortality_Inhive_Infected_As_Adult*), which can depend on whether and when a bee was infected by a virus transferred by varroa mites. Virus infection may happen during pupation (when it is determined on the day of emergence of young adult bees in the procedure MitesReleaseProc) or virus infection may be caused by phoretic mites on adult bees. Hence, regarding mortality, we distinguish amongst three sub-cohorts of adult worker bees (healthy, infected as adult, and infected as pupa) and two for drones (healthy and infected as pupa - adult drones are not infested by phoretic mites in the model). Adult in-hive workers will develop into foragers, when they reach the age *Aff*. This process is described in following section "Transition to foragers".

7.7. Transition to foragers

Rationale: transition to foragers

Honey bees show a temporal polyethism and perform tasks inside the hive (e.g. brood care) at earlier ages than tasks outside the hive (e.g. foraging) (Rösch 1925, reviewed in Robinson 1992, Robinson 2002). The age of first foraging can be delayed if enough foragers are present or accelerated if there is additional demand (Rösch 1930, Huang and Robinson 1996, Robinson and Huang 1998). It is assumed that foragers inhibit the behavioural development of younger bees, and that lost foragers are equally replaced by new foragers (Huang and Robinson 1992, Huang and Robinson 1996, Leoncini et al. 2004). Precocious foraging can also be induced by food shortage of the colony (Schulz et al. 1998, Toth et al. 2005), whereas high concentrations of brood pheromone result in a later onset of foraging (Le Conte et al. 2001). Among other factors, the age of first foraging is also affected by the genotypes of the bees (Guzman-Novoa et al. 1994), their infection state (Woyciechowski and Moron 2009) and developmental temperatures (Becher et al. 2009). Studies reviewed by Winston (1987) show a huge variation in the onset of foraging with the minimum age between 3 and 20 days, a maximum between 27 and 65 days and an average between 18 and 38 days.

While the BeePop model assumes a constant age of first foraging (21 days), HoPoMo does not differentiate between in-hive bees and foragers, but allows all workers to perform in-hive and outside tasks. In a model by Becher et al. (2010) the onset of foraging varies with the developmental temperature around a default value of 15 days. There are some models specifically addressing the transition from in-hive workers to foragers, like the model of Beshers et al. (2001) or Khoury et al. (2011, 2013), which calculate the age of first foraging on the basis of social inhibition. However, many of their parameters lack biological meaning. Amdam and Omholt (2003) provide a model that simulates in great detail the physiological processes within a worker bee to predict the age of first foraging.

To calculate a cohorts' age to develop into foragers (*Aff*), the BEEHAVE model takes social interactions into account, via the ratio of in-hive bees to foragers, as well as the effect of brood via the ratio of brood to nurse bees. Moreover, reduced honey or pollen stores or a decrease in the protein content of the brood food results in an earlier onset of foraging. Strong deviations of the current *Aff* from an age of first foraging base value (*Aff_Base*, 21 days) affect the onset of foraging in the opposite direction, i.e. there is a tendency to increase *Aff* if it is small and to decrease it, if it is large.

Procedures involved:

- AffProc
- WorkerIHbeesDevProc

In-hive bees, reaching the age of first foraging (*Aff*) become foragers. The calculation of the age of first foraging takes place in the procedure AffProc, which is called at the beginning of *WorkerIHbeesDevProc*. The factors that are used to determine the age of first foraging are: pollen and honey stores, amount of brood, protein content of nurse bees and the forager to in-hive worker ratio and the deviation of the current *Aff* from the base age of first foraging (*Aff_Base*).

For each of these factors, a stimulus is defined (e.g. actual stores relative to maximal stores) and a threshold (indicated by "TH" at the end of the variable name). If the stimulus exceeds the threshold, *Aff* changes by one or by two days. If several thresholds are exceeded, *Aff* can change within this procedure several times in either direction. However, the final value for *Aff* cannot deviate by more than one day from the *Aff* value of the day before and it cannot be

larger than *Max_Aff* nor smaller than *Min_Aff*. As reliable, empirical data are missing, the definition of these stimulus-threshold relations are based on heuristic tests of the model to create a reasonable colony response to a variety of environmental and intra-colonial conditions.
At the beginning of the procedure AffProc, the current (= yesterday's) Aff is saved in the local variable *affYesterDay* and the thresholds (*pollenTH*, *proteinTH*, *honeyTH*, *broodTH*, *foragerToWorkerTH*) are defined.

The impact of pollen stores is calculated as:

```
if PollenStore_g / IdealPollenStore_g < pollenTH [ set Aff Aff - 1 ]
```

where *PollenStore_g* represent the actual amount of stored pollen [g] and the *IdealPollenStore_g* reflects a theoretical pollen store that would last for about seven days (see PollenConsumptionProc).

The impact of the protein content in the nurses' brood food is calculated as:

```
if ProteinFactorNurses < proteinTH [ set Aff Aff - 1 ]
```

The *ProteinFactorNurses* ranges from 1 to 0 and reflects the relative protein content of the brood food (more details in the "Consumption" section).

If the honey store is too small, the preliminary value of *Aff* is reduced by *two* days:

```
if HoneyEnergyStore < honeyTH [ set Aff Aff - 2 ]
```

where *honeyTH* reflects a theoretical honey store that would last for about 35 days. As the colony immediately dies in the model if it runs out of honey, this factor changes the preliminary value of *Aff* by *two* days.

Also the impact of social interaction between in-hive bees and foragers on the onset of foraging are considered, so that the presence of older bees affects the behavioural development of the younger bees:

```
if (TotalIHbees > 0) and (TotalForagers / TotalIHbees < foragerToWorkerTH)
  [ set Aff Aff - 1 ]
```

All factors listed so far have *decreased* the onset of foraging. The main factor that *increases* *Aff* is the amount of brood relative to the available nurse bees:

```
if (...)TotalWorkerAndDroneBrood / ((TotalIHbees + TotalForagers * FORAGER_NURSING_CONTRIBUTION) * MAX_BROOD_NURSE_RATIO) > broodTH
 [ set Aff Aff + 2 ]
```

To increase the relative weight of the brood factor, a lack of nurse bees increases the preliminary value of *Aff* by *two* days.

To avoid extreme values, the preliminary *Aff* is changed towards *Aff_Base* by one day, if *affYesterDay* deviates from *Aff_Base* by more than seven days:

```
if affYesterDay < AFF_BASE - 7 [ set Aff Aff + 1 ]
if affYesterDay > AFF_BASE + 7 [ set Aff Aff - 1 ]
```

At last, it is assured that the final, new value for *Aff* deviates from the old value by no more than one day and is within the range defined by *Max_Aff* and *Min_Aff*.

While the first part of WorkerIHbeesDevProc deals with ageing and mortality of the in-hive bee cohorts, the second part of the procedure addresses the onset of foraging. After today's *Aff* is determined in AffProc, the number of new foragers can be calculated. This has to be done for all three sub-cohorts (i.e. "healthy", "infected as adults" and "infected as pupae") separately.

Due to possible changes in *Aff*, there might be two cohorts developing into foragers at the same time step, hence *number_healthy* has to be summed up, using the variable *NewForagerSquadronsHealthy*, for all healthy in-hive bee cohorts that reach or exceed the age of first foraging:

```
if age >= Aff
[ set NewForagerSquadronsHealthy
          floor (number_healthy / SQUADRON_SIZE) + NewForagerSquadronsHealthy
  ...
]
```

(with "floor" meaning to round a floating point number to the next lower integer).

As foragers are implemented as super-individuals with a group size of Squadron_Size (100 in all model runs), there might always be some bees “lost” due to the rounding. To avoid this loss, these bees are addressed as "over-aged" and added to the one day *younger* cohort:

```
set overagedIHbees number_healthy mod SQUADRON_SIZE
ask IHbeeCohorts with [ age = Aff - 1 ]
 [ set number number + overagedIHbees
   set number_healthy number_healthy + overagedIHbees ]
```

For example, 260 healthy in-hive bees with age = *Aff* would result in two new *foragerSquadrons* and 60 bees joining the cohort with age = *Aff* - 1.

These processes are then repeated in a similar way for the two remaining sub-cohorts ("infected as adults" and "infected as pupae").

In contrast to other "development" procedures, the cohorts are handled in WorkerIHbeesDevProc in an ordered way, starting with the youngest. This is to avoid a possible loss of over-aged cohorts.

7.8. Consumption

Rationale: Honey and pollen consumption

Worker bees rely on honey or nectar as source for carbohydrates. Young adults (8-10 days) and nurse bees also require pollen which provides proteins and lipids (Hrassnigg and Crailsheim 1998, Brodschneider and Crailsheim 2010). While eggs and pupae do not consume anything, larvae need to be fed with plenty of proteinaceous brood food produced by nurse bees and also some pollen at later stages (reviewed in Winston 1987, Crailsheim 1990).

Schmickl and Crailsheim (2007) suggest that a bee larva consumes 142mg pollen and 163mg nectar to reach adulthood. For adult workers, they assume task specific nectar and pollen consumption rates, but the authors do not provide the actual parameterisation.

For worker brood we use honey and pollen consumption rates based on Schmickl and Crailsheim (2007), but do not differentiate this dependent on the age of the larvae. Energy consumption by resting and nursing workers is derived from Rortais et al. (2005) ("winter bees" and "brood attending bees" (i.e. bees heating the brood nest)). We assume that the difference in honey consumptions between resting bees and "brood attending" bees represents the energy spend for thermoregulation of the brood nest.

Nectar consumption rates for drone larvae are also taken from Rortais et al. (2005), for adult drones it is estimated from Winston (1987) and references therein. The energy used during foraging flights is calculated in the foraging module. Although pollen consumption by adult workers decreases with age, we assume it to be constant using the average consumption rates from Pernal and Currie (2000) for 14 days old workers. Pollen consumed by nurses to produce brood food is taken into account via the pollen consumption by larvae.

Procedures involved:

- HoneyConsumptionProc
- PollenConsumptionProc

These procedures calculate the daily consumption of honey and pollen and the resulting reduction of honey and pollen stores.

In the procedure HoneyConsumptionProc, the daily honey consumption for adults and larvae of workers and drones are defined. To calculate the honey consumption, these values are then multiplied with the number of individuals in the corresponding group. Eggs and pupae do not consume any food. For adult workers we use two values, defined as local variables: *Daily_Honey_Need_Adult_Resting* (11 mg honey/day), which reflects the honey consumption of resting bees, and *Daily_Honey_Need_Nurses* (53.42 mg honey/day), which reflects the honey consumption of bees attending and heating the brood nest. Dividing the difference of these two honey consumption rates by the number of brood cells a single bee can attend should reflect the additional cost of a single brood cell to be maintained at the right temperature:

```
let THERMOREGULATION_BROOD (DAILY_HONEY_NEED_NURSES
    - DAILY_HONEY_NEED_ADULT_RESTING)
    / MAX_BROOD_NURSE_RATIO
```

Then, multiplying *Thermoregulation_Brood* with the total numbers of drone and worker brood (*TotalWorkerAndDroneBrood*) reflects the total energetic costs of brood care.

Honey consumption of adults includes in-hive bees, foragers and drones:

```
let needHoneyAdult
      ((TotalIHbees + TotalForagers) * DAILY_HONEY_NEED_ADULT_RESTING
      + TotalDrones * DAILY_HONEY_NEED_ADULT_DRONE)
```

Note that the additional energy consumption of those foragers actually involved in foraging activities is calculated in the foraging model.

The total honey consumption of the colony (in mg) is then

```
set DailyHoneyConsumption
     needHoneyAdult + needHoneyLarvae
     + TotalWorkerAndDroneBrood * THERMOREGULATION_BROOD
```

and is removed from the colony's honey store:

```
set HoneyEnergyStore
      HoneyEnergyStore - (DailyHoneyConsumption / 1000) * ENERGY_HONEY_per_g
```

For model analysis, the Boolean variable *HoneyIdeal* can be set to *true*, which means the honey store is set to its maximal value at the end of the procedure, i.e. there is no feedback between the state of the colony, its corresponding consumption, and the honey stores, which are constant and optimal.

Similar to the honey consumption, the procedure PollenConsumptionProc first defines the daily pollen consumption by adults and larvae of workers and drones. These values are then multiplied with the number of individuals in each group.

The total pollen consumption (in g) is then:

```
set DailyPollenConsumption_g (needPollenAdult + needPollenLarvae) / 1000
```

which is removed from the colony's pollen store (*PollenStore_g*); here, we use units of gram instead of milligram, hence the factor 1000.

Additionally, an "ideal" pollen store (*IdealPollenStore_g*) is calculated. It represents the amount of pollen a colony tries to keep (depending on its current pollen consumption) so that it would provide the colony with sufficient pollen for about seven days (*pollenStoreLasting_d*), even if no new pollen would be collected by the foragers:

```
set IdealPollenStore_g DailyPollenConsumption_g * pollenStoreLasting_d
```

The minimum of *IdealPollenStore_g* is *Min_Ideal_Pollen_Store*, which guarantees that foragers try to collect pollen for raising new brood in near future, even if the current pollen consumption is rather low (e.g. in spring). If the Boolean variable *PollenIdeal* is *true*, the pollen store is set to its "ideal" value.

If the actual pollen store is depleted, the protein content of the brood food (*ProteinFactorNurses*), produced by the nurse bees is reduced. We do not calculate the absolute protein concentration but assume a relative value, ranging from 0 (no proteins) to 1 (maximal protein concentration) (see section 7.5 "Brood care"). In a first step, the current workload of the nurse bees is calculated:

```
set workloadNurses
      TotalWorkerAndDroneBrood /
        ((TotalIHbees + TotalForagers * FORAGER_NURSING_CONTRIBUTION)
        * MAX_BROOD_NURSE_RATIO)
```

and then the *ProteinFactorNurses* is decreased (with a lower limit of 0):

```
set ProteinFactorNurses ProteinFactorNurses -
    (workloadNurses / PROTEIN_STORE_NURSES_d)
```

We thus assume that the protein in the brood food decreases proportionally to the amount of brood the nurses have to care for, with Protein_Store_Nurses_d being 7 days.

As soon as pollen is available again, the *ProteinFactorNurses* increases (to a maximum of 1):

```
set ProteinFactorNurses ProteinFactorNurses + (1 / PROTEIN_STORE_NURSES_d)
```

7.9. Swarming

Rationale: Swarming

A honey bee colony reproduces by swarming, where the old queen leaves the hive, together with roughly half of all workers and drones. In preparation for swarming, new developing queens have to be produced and the old queen reduces her egg laying about one week before swarming. After the swarm has left, the young queen emerges, conducts her mating flight and then starts to lay eggs (reviewed in Winston 1987).

Schmickl and Crailsheim (2007) suggest a procedure to model swarming with HoPoMo. However, the date of swarming has to be set by the user. Fefferman and Starks (2006) test several triggers of swarming in their model, e.g. colony size, amount of brood.

If swarming is allowed in BEEHAVE, it is triggered by the total amount of brood in the colony, with a threshold set to 17000 (as suggested by Fefferman and Starks 2006). The simulation either follows the parental colony or the (prime) swarm.

Procedures involved:

- SwarmingProc

In this procedure it is determined if and when swarming takes place. The colony size and stores are then reduced to simulate either a departing swarm or the parental colony. BEEHAVE is designed to primarily focus on a single colony; the option to take swarming into account is mainly included to be able to observe the colony performance after a sudden, extreme but nevertheless realistic change in colony size, colony composition and stores. In the default setting, swarming is deactivated.

Swarming is triggered if the total amount of brood is larger than *broodSwarmingTH* (17000) and if date is lower than *lastSwarmingDate* (199 = 18 July):

```
if TotalWorkerAndDroneBrood > broodSwarmingTH and SwarmingDate = 0
and day <= (lastSwarmingDate - PRE_SWARMING_PERIOD)
     [ set SwarmingDate (day + PRE_SWARMING_PERIOD) ]
```

with *SwarmingDate* being set to 0 at the beginning of a simulation and after swarming has taken place.

The actual swarming takes place *Pre_Swarming_Period* days after this swarming event has been triggered. During this period of swarm preparation, no eggs are laid.

Immediately after swarming has taken place, *SwarmingDate* is reset to 0 to allow further swarms to emerge.

Parental colony
If the option is chosen to follow the fate of the parental colony, the simulation continues with that part of the colony that stays in the hive, with a new queen soon to be emerging. On the day of swarming, a fraction of *fractionSwarm* (0.6) bees leaves the colony, together with the old queen and the corresponding number of phoretic varroa mites. The leaving bees fill their crops with honey and hence reduce the honey stores in the hive by 36 mg per bee (Winston 1987). The age of the new queen is set to -7 days (reflecting a queen that is still in her cell) and she will not start egg laying until she is 10 days old (see "Egg laying" section).

All cohorts (healthy and infected) of adult workers and drones are then reduced by the factor (1 - *fractionSwarm*). The number of healthy in-hive bees for example is:

```
ask IHbeeCohorts
        [ set number_Healthy round (number_Healthy * (1 - fractionSwarm)) ]
```

Also the number of foragers is reduced accordingly, by randomly removing foragers (i.e. by letting them die) according to the probability fractionSwarm:

```
 ask foragerSquadrons [ if random-float 1 < fractionSwarm [ die ] ]
```

Finally, also the number of phoretic mites is reduced:

```
 set PhoreticMites round (PhoreticMites * (1 - fractionSwarm))
```

The number of mites in the brood cells as well as the brood itself does not change. However, the day after swarming brood may die due to the sudden reduction in the number of bees available for brood care (see section 7.5. "Brood care").

Prime swarm
The same processes take place if the simulation continues with the prime swarm, in which case the swarm leaving the hive together with the old queen is the part followed by the model while the parental colony, including its brood, its mites, and the food stores are removed. Hence, all *number* for all brood cohorts are set to zero, e.g.:

```
 ask (turtle-set eggCohorts larvaeCohorts droneEggCohorts droneLarvaeCohorts)
         [ set number 0 ]
```

The number of adults is reduced to those who join the swarm, e.g. for healthy in-hive bees:

```
 ask IHbeeCohorts
     [set number_Healthy round (number_Healthy * fractionSwarm) ]
```

The number of foragers is reduced by those who stay in the old hive:

```
 ask foragerSquadrons
      [ if random-float 1 < (1 - fractionSwarm) [ die ] ]
```

Only a fraction of phoretic mite remains in the colony, whereas all mites in brood cells are removed:

```
ask miteOrganisers [ die ]
set PhoreticMites round (PhoreticMites * fractionSwarm)
```

The pollen stores are set to zero and the honey stores reduced to the 36 mg per worker bee, which are kept in the crops of the bees.

It is assumed that a swarm finds a suitable new home after *Post_Swarming_Period* days. During this post swarming period, the pollen store remains at zero, no eggs can be laid and the honey store cannot exceed 36mg per bee.

7.10. Beekeeping

- BeekeepingProc

In the procedure BeekeepingProc, bee keeping practices like varroa treatment, honey harvest and feeding of the colony are performed. All bee keeping processes are optional and can be chosen on the interface by the user; they are not included in the default settings of BEEHAVE.

If *FeedBees* is *true*, honey stores can be stocked up if they are below a certain threshold. The threshold *minWinterStore_kg* (16 kg) describes the minimal honey store a colony should have, before the winter pause starts. During the winter pause, which is defined by the local variables *winterPauseStart* (day 320 = 16 November) and *winterPauseStop* (day 45 = 14 February), no bee keeping practices are allowed. If the honey stores on the day *winterPauseStart* are too low, they are stocked up to *minWinterStore_kg*. Outside of the winter pause, the lower honey store threshold *minSummerStore_kg* (3 kg) applies. If it is undercut, a relatively small amount *addedFondant_kg* (1 kg) of fondant (i.e. sugar paste) is added to the honey store.

If *AddPollen* is *true*, the amount of *addedPollen_kg* (0.5 kg) pollen is added to the pollen store in spring (day 90 = 31 March), to boost the colony growth.

The colony size of a weak colony can be increased, if *MergeWeakColonies* is *true* and the colony size is below *MergeColoniesTH* on the day *winterPauseStart*. In this case, *MergeColoniesTH* foragers, aged between 60 and 100 days, are added.

If *HoneyHarvesting* is *true*, then honey will be harvested from the day *HarvestingDay* onwards for *HarvestingPeriod* days, whenever the honey stores exceed *HarvestingTH*. They are then reduced to *RemainingHoney_kg*.
If *QueenAgeing* is *true* (see section "Egg laying") so that the egg laying rate decreases with he age of the queen, the queen will be replaced when she is 375 days old by a young queen, 10 days of age.

Finally, also varroa treatment can take place if *VarroaTreatment* is *true*. During a period, defined by *treatmentDay* (270 = 27 September) and *treatmentDuration* (40 d), all phoretic mites are exposed to an additional daily mortality:

```
set PhoreticMites round(PhoreticMites * (1 - treatmentEfficiency))
```

This applies to phoretic mites only, not to mites in brood cells.

7.11. Graphic user interface & output

More details on the graphic user interface are given in a separate user manual.

Procedures involved:

- DrawIHcohortsProc
- DoPlotsProc
- DrawForagingMapProc
- GenericPlottingProc
- GenericPlotClearProc
- WriteToFileProc
- CreateOutputFileProc

DrawIHcohortsProc draws the representations of all in-hive cohorts as histogram in the Netlogo "world". The in-hive cohorts shown are: eggs, larvae, pupae for workers and drones, as well as adult in-hive workers and adult drones. The infection states (healthy, infected as pupa, infected as adult) are also represented.

In DoPlotsProc, *Signs* for beekeeping activity and for changes in the honey and pollen stores can be shown or hidden. If *ShowAllPlots* is true, then DrawForagingMapProc is called, where the "foraging map" plot is defined. The "foraging map" plot can show all available patches, all patches that currently offer nectar or pollen or it shows the nectar and pollen foraging activities of the bees at the current time step on a 2-dimensional representation of the landscape. Finally, GenericPlottingProc is called repeatedly, once for each of the "generic plots". The variables to be displayed in such plots can be selected from a Netlogo drop-down "chooser". Plots showing the detailed foraging activities during a foraging round (i.e. "foragers today [%]" and "active foragers today [%]") need to be cleared once a day, which is done in GenericPlotClearProc.

To run simulations on a large scale, we suggest using the Netlogo "BehaviorSpace" tool, which makes repeated runs of different scenarios with tailored outputs easy. However, BEEHAVE in principle offers the possibility to generate an output file defined in WriteToFileProc, though the current code in this procedure only serves as an example and per default, the write-to-file function is switched off. In CreateOutputFileProc, called by the "write file" button, the output file is created and named.

II - THE FORAGING MODEL

Rationale: Foraging

Foragers collect mainly nectar and pollen for nutrition, but also water for thermoregulation and brood rearing as well as propolis for nest construction (reviewed in Winston 1987 and Seeley 1995). A forager has not only to decide if she leaves the colony to search or collect food, but also what kind of food she wants to collect, where to collect it, and finally, in case she returns successfully, if she should dance to recruit other bees.

Usually, foragers are stimulated by dancing bees to become active (Lindauer 1952). However, 5 - 35% of the foragers leave the hive as scouts to search for new food sources (Seeley 1983).

The overall forager traffic is strongly influenced by the weather and requires temperatures of at least 12 - 14°C and only little wind or rain (reviewed in Ribbands 1953). Furthermore, intra-colonial conditions affect the foraging efforts, where small food stores and empty comb space enhance the foraging efforts and increase the acceptance for less efficient nectar sources (Rinderer and Baxter 1978, Seeley 1989).

The proportion of pollen foragers mainly depends on the demand of pollen in the colony and can vary hugely, from very few to over 80% (Lindauer 1952). Low pollen stores or a large amount of brood results in an increasing pollen intake (Fewell and Winston 1992, Eckert et al. 1994, Pankiw et al. 1998).

It is unclear, whether the colony's pollen stores are directly assessed by pollen foragers (Dreller et al. 1999, Dreller and Tarpy 2000, Calderone and Johnson 2002, Vaughan and Calderone 2002), or indirectly, when the foragers receive trophallactically jelly from nurse bees with varying protein content (Crailsheim 1991, Camazine 1993, Weidenmüller and Tautz 2002, but see also Sagili and Pankiw 2007). However, there is no doubt that a colony adapts its pollen foraging efforts according to its pollen needs, so that their pollen stores last for about one week (Seeley 1995, Schmickl and Crailsheim 2004).

As colonies require large honey stores for successful overwintering, foragers generally try to acquire as much nectar as possible (Seeley 1995). Feeder experiments, offering sugar water at various distances and concentrations, suggest that there is a linear relationship between the energetic efficiency of a nectar patch and the number of waggle runs a returning forager performs to advertise this patch, which then affects the number of dance followers (Seeley and Towne 1992, Seeley 1994). During a waggle dance, not only the location of a food source in distance and direction is communicated, but also floral odours are perceived by the dance follower (von Frisch 1967, Dyer 2002, Riley et al. 2005). Information on the profitability of a food source may also be encoded in the thorax temperature of a dancer (Stabentheiner and Hagmüller 1991, Stabentheiner 2001). Additionally, foragers, dance followers and nectar receiving bees are well informed about the quality of incoming nectar, which they share via trophallaxis (Seeley 1992, Farina 1996, Crailsheim 1998, Farina 2000). The time to find a receiver bee for unloading their nectar will inform a forager about the current value of her load and may influence her future foraging decisions (Seeley 1986, 1989, 1992, Kirchner and Lindauer 1994). Though foragers also dance for pollen sources, little is known about which factors affect the motivation of a pollen forager to dance

The mortality risk during foraging is quite high. Visscher and Dukas (1997) calculated the lifespan of a forager as 7 days (median) and the probability to die as 0.036 per hour foraging.
Estimates for energy consumption during flights are about 0.006 J/m (Schmid-Hempel et al. 1985, Goller and Esch 1990). The average foraging range is about 1.5 km, but depends on the forage availability and is higher for pollen foragers than for nectar foragers. Maximum foraging ranges are about 10 km (Visscher and Seeley 1982, Steffan-Dewenter and Kuhn 2003).

So far, no honey bee colony model has combined an explicit foraging model with a model of colony dynamics. Schmickl and Crailsheim's (2007) HoPoMo calculates the influx of nectar and pollen simply on the basis of a few parameters, representing weather conditions, foraging success, forage load, stochastics, and number of active foragers

Existing foraging models on the other hand are not combined with a colony model and they focus on nectar foraging but ignore pollen collection (e.g. Camazine and Sneyd 1991, de Vries and Biesmeijer 2002, Dornhaus et al. 2006, Schmickl et al. 2012).

The BEEHAVE foraging module basically follows to concept of Sumper and Pratt 2003, concerning the behavioural states of the foraging process (shown in their Fig. 1). However, we add foraging mortality and represent the choice between nectar and pollen collection. We chose energetic efficiency as the currency for nectar foraging and the duration of a foraging trip as criterion to assess the quality of a pollen patch. Patches are defined by the amount of nectar and pollen they offer on each day, the sugar concentration of the nectar, their distance to the colony and the probability to be detected by a searching scout bee. These detection probabilities are either modelled (individual-based) or simply calculated in an external landscape module. Weather conditions affect the daily foraging period. Foraging probabilities and forage choice depend on the need of the colony. We did not include foraging for water or propolis, as only a small number of bees performs these tasks.

7.II.1 Foraging

Procedures involved:

- Start_IBM_ForagingProc
- Foraging_ProbabilityREP
- Foraging_PeriodREP

Start_IBM_ForagingProc is the main procedure of the foraging model and is only called if there are foragers present in the colony (see procedure *Go*). It calls the procedures for forager creation and development, calculates the number of foraging rounds, determines the probability to start foraging spontaneously, and the foraging motivation of older foragers ("laziness").

Foraging activities involve a high mortality risk. If bees pursue too many foraging trips, especially during winter, when no young bees are produced, the colony size may become too small to survive the winter. To avoid such forager losses, a degree of laziness can optionally be defined which applies to foragers older than a certain age. Lazy bees will not forage and cannot be recruited on that day. Before the first foraging trip of the day takes place, all foragers with an age of at least *ageLaziness* (100 d) may become lazy. This is the case if a random number is smaller than *ProbLazinessWinterbees* and another random number is smaller than *HoneyEnergyStore* / *DecentHoneyEnergyStore* (*DecentHoneyEnergyStore* is explained in the section "Foraging_round"). These two factors increase the longevity of foragers, especially in autumn and winter, but without increasing the risk of starvation of the colony due to laziness. Per default, *ProbLazinessWinterbees* is set to zero so that there are no lazy bees.

The probability that a forager "resting" inside the hive starts foraging spontaneously (*ForagingSpontaneousProb*) is calculated in Foraging_ProbabilityREP:

The initial foraging probability *foragingProbability* is set to 0.01; this value will be used for intermediate pollen and nectar stores. If the stores for pollen or honey are low, the foraging probability is increased to *highForProb* (0.05). If the honey stores are very low, it is increased to *emergencyProb* (0.2). In contrast, if there is sufficient pollen stored and enough honey to survive the winter, foraging probability is set to 0.

Foraging is only allowed during the season, which is defined by *Season_Start* and *Season_Stop*. As these are per default set to Julian day 1 and 365 respectively, the season lasts for the whole year and hence does not restrict foraging. Additionally, foraging only takes place, if either the honey stores are filled to less than 95% or if the pollen store is less than the "ideal" pollen store (*IdealPollenStore_g*). Furthermore, foraging requires adequate weather conditions. The total duration of adequate weather conditions on the current day is represented by *DailyForagingPeriod* and calculated in the Foraging_PeriodREP (called in DailyUpdateProc):

Depending on the setting of *Weather* (a Netlogo "chooser" on the interface tab) a specific weather scenario is chosen. Options like "Rothamsted (2009)" or "Berlin 2000 - 2006" define *DailyForagingPeriod* for each day of one or several years and are based on real weather data. *DailyForagingPeriod* describes the amount of time bees are allowed to spend on foraging per day. We used the hours of sunshine on days with a maximum temperature above 15°C from those weather data as an approximation to the hours of suitable weather for foraging. "HoPoMo_Season" provides theoretical data, based on the same bell shaped curve as we use for egg-laying. "HoPoMo_Season_Random" is similar but with some random variation added. "Constant" finally results in a constant foraging period of eight hours a day.

If all conditions for foraging are fulfilled, the procedure ForagingRoundProc (described in the section "Foraging round") is repeatedly called until the time allowed for foraging on that day is over and hence *continueForaging* is set *false*. At the end of each foraging round, the duration of this foraging round is calculated (= *meanTripDuration*) and summed up in the variable *summedTripDuration*. If *summedTripDuration* exceeds *DailyForagingPeriod*, foraging stops for that day.

MeanTripDuration is calculated as the average trip duration of all foragers active during this foraging round:

```
ifelse ColonyTripForagersSum > 0
     [ set meanTripDuration ColonyTripDurationSum / ColonyTripForagersSum ]
     [ set meanTripDuration HANGING_AROUND ]
```

where *ColonyTripDurationSum* and *ColonyTripForagersSum* are calculated in Foraging_flightCosts_flightTimeProc by addressing unsuccessful scouts, and successful pollen and nectar foragers. A code example for unsuccessful scouts:

```
ask foragerSquadrons with [ activity = "searching" ]
  [ ..set ColonyTripDurationSum ColonyTripDurationSum
          + (SEARCH_LENGTH_M / FLIGHT_VELOCITY )
      set ColonyTripForagersSum ColonyTripForagersSum + 1.. ]
```

In case that no foraging at all takes place during this foraging round, a duration of this round is set to *Hanging_Around*, which is defined as *Search_Length_m* / *Flight_Velocity* (= 17 minutes).

For example, at the beginning of a days' foraging period, all foragers might stay in the hive, and the duration of the first foraging round would be 17 minutes (=*Hanging_Around*). If in the second foraging round half of the active forager force would spend 10 minutes for their foraging trips to a nearby food source and the other half of the foragers would spend 20 minutes foraging at a patch further away, the duration of this second foraging round would be 15 minutes (= *meanTripDuration*) and all foragers would return to the hive at the same time. *SummedTripDuration* would now be 32 minutes. If all foragers went to the nearest patch in all following foraging rounds, the durations of these foraging rounds would be 10 minutes and the *summedTripDuration* after the third foraging round would hence be 42 minutes. Assuming the weather conditions on that day allow foraging for 50 minutes (=*DailyForagingPeriod*), then foraging would cease after the fourth foraging round, as *summedTripDuration* (52 minutes) then exceeds *DailyForagingPeriod*. This approach, although not realistic, allows to conveniently use foraging rounds but with a variable duration.

At the end of Start_IBM_ForagingProc the procedure ForagersLifespanProc is called to determine the survival of the foragers (see next section) and finally, the *activity* of all foragers is set to "resting" again.

7.II.2. Forager development

Procedures involved:

- ForagersDevelopmentProc
- NewForagersProc
- ForagersLifespanProc

The number of new foragers is determined in the procedure WorkerIHbeesDevProc (see section "Transition to foragers"). The new foragers are then created in the procedure NewForagersProc.

The initial bees, created in the first time step of a simulation, get a randomly chosen age between 100 and 160 days and their total lifetime flight distance (*mileometer*) is randomly set between 0 and (*Max_Total_km* / 4). All other foragers created in later time steps get an age of *Aff* (age of first foraging) and their *mileometer* is set to 0. The *infectionState* results from the number of foragers created from the in-hive bee sub-cohorts "healthy", "infectedAsPupae" or "infectedAsAdult".

Foragers age by one day every day and their bee-shaped representation on the interface moves further to the right to visualise their age (*ForagersDevelomentProc*). In the procedure ForagersLifespanProc, called at the end of a foraging day, mortality of the foragers is determined (additional to the mortality during a foraging trip).

Foragers die at the end of a day if their *age* exceeds *Lifespan* or if their *mileometer* (the total length of all trips during their life summed up) exceeds *Max_Total_km*. Additionally, they die with the probability *dailyRiskToDie*, which is set to *Mortality_Inhive*, *Mortality_Inhive_Infected_as_Pupa*, or *Mortality_Inhive_Infected_As_Adult*, depending on their *infectionState*.

The total number of deaths is recorded (*DeathsAdultWorkers_t*) and is an optional output.

7.II.3. Foraging round

Procedures involved:

- ForagingRoundProc
- FlowerPatchesUpdateProc
- Foraging_start-stopProc
- Foraging_searchingProc
- Foraging_collectNectarPollenProc
- Foraging_flightCosts_flightTimeProc
- Foraging_mortalityProc
- Foraging_dancingProc
- Foraging_unloadingProc

In the procedure ForagingRoundProc, the *DecentHoneyEnergyStore* is calculated:

```
set DecentHoneyEnergyStore (TotalIHbees + TotalForagers) * 1.5
                            * ENERGY_HONEY_per_g
```

which reflects the energy a colony should store to survive the winter, based on the assumption that each bee consumes ca. 1.5g honey during winter.

Next, the probability of a forager to collect pollen instead of nectar is calculated:

```
set ProbPollenCollection (1 - PollenStore_g / IdealPollenStore_g)
    * MAX_PROPORTION_POLLEN_FORAGERS
```

where *PollenStore_g* represents the actual amount of stored pollen [g] and *IdealPollenStore_g* reflects a theoretical pollen store that would last for about seven days (see PollenConsumptionProc).

To avoid starvation of the colony if honey stores are low, the probability to collect pollen is then related to the relative amount of stored honey:

```
if HoneyEnergyStore / DecentHoneyEnergyStore < 0.5
  [
    set ProbPollenCollection ProbPollenCollection
    * (HoneyEnergyStore / DecentHoneyEnergyStore)
  ]
```

Subsequently, several procedures are called, dealing with the details of the foraging processes and at last, several output variables (e.g. *currentNectarForagers*, *currentPollenForagers*) are defined and foraging related plots are updated on the interface.

FlowerPatchesUpdateProc

In this procedure handling times, energetic efficiency, flight costs, trip duration, foraging mortality rate and dance circuits are updated, which are all described by state variables of the flower patches.

Handling times:

The handling time is the time a forager needs to collect a nectar (*handlingTimeNectar*) or pollen load (*handlingTimePollen*) at the patch. Handling times are set to *Time_Nectar_Gathering* and *Time_Pollen_Gathering* or, if *ReadInfile* is *true*, they are read

in once a day from a list (*TodaysSinglePatchList*) which is based on an input file. If *ConstantHandlingTime* is *true* the handling times (*handlingTimeNectar*, *handlingTimePollen*) are constant during a day. If *ConstantHandlingTime* is *false* (default setting) the handling times increase during a day with the degree of food depletion at the flower patch (flower patches are completely replenished on the next day). This reflects that bees have to search longer, the more flowers are depleted. To determine the handling times in this case the maximal availability of nectar and pollen on the current day is required. This maximal availability is either based on input variables on the interface (*Quantity_R/G_l*, *Pollen_R/G_kg*) and then caclulated in *FlowerPatchesMaxFoodAvailableTodayREP* (if *ReadInfile* is *false*) or the maximal availability read in from an input file, (if *ReadInfile* is *true*).

FlowerPatchesMaxFoodAvailableTodayREP is a reporter procedure which uses *patchID* and *foodType* as input variables. It reports the maximal amount of either nectar or pollen (depending on *foodType*) available at the flower patch *patchID* for the current *day*. If the global variable *SeasonalFoodFlow* is *true* the maximal amount of nectar and pollen at a patch depends on the season, represented by the seasonal factor from the HoPoMo model. For example, the amount of nectar [µl] offered at the "red" patch (*patchID* = 0) is then:

```
let patchDayR day + SHIFT_R
report (1 - Season_HoPoMoREP patchDayR []) * QUANTITY_R_l * 1000 * 1000
```

Shift_R allows for earlier or later onset of flowering in the patch, *Season_HoPoMoREP* (0..1) calculates the seasonal factor and *Quantity_R_l* is the overall maximal amount of nectar [l]. For pollen availability is calculated in a similar way.
If *SeasonalFoodFlow* is *false* the maximal amount of nectar and pollen at all patches is constant and independent of the season (i.e. *Quantity_R/G_l*, *Pollen_R/G_kg*, but transferred to the units [µl] and [g]). If *ReadInfile* is *true* *FlowerPatchesMaxFoodAvailableTodayREP* is not called and hence *SeasonalFoodFlow* has no effect.

The degree of nectar or pollen depletion at the patch is subsequently calculated by dividing the maximal amount of food that could be available at the patch by the actually available amount of food. The handling time is then increased accordingly and, if *ReadInfile* is *false*, calculated for nectar as:

```
set handlingTimeNectar TIME_NECTAR_GATHERING *
              ((FlowerPatchesMaxFoodAvailableTodayREP who "Nectar") / quantityMyl)
```

and similarly for pollen:

```
set handlingTimePollen TIME_POLLEN_GATHERING *
           ((FlowerPatchesMaxFoodAvailableTodayREP who "Pollen") / amountPollen_g)
```

If *ReadInfile* is *true*, the handling time is calculated in a similar way, however the maximum amount of food available today as well as the base value of the handling times, are then taken from an input file. For example the handling time for nectar is calculated as:

```
set handlingTimeNectar (item 13 TodaysSinglePatchList) *
              ((item 10 TodaysSinglePatchList) * 1000 * 1000) / quantityMyl
```

where "item 13" refers to the base value of handling time for nectar and "item 10" to the (maximal) quantity of nectar [l] available at the patch today. *TodaysSinglePatchList* is a short

excerpt of the input file, but contains only data of the currently processed flower patch and only for the current day.

Trip duration:
Using these handling times, the duration of a foraging trip [s] can then be calculated for nectar:

```
set tripDuration 2 * distanceToColony * (1 / FLIGHT_VELOCITY )
                 + handlingTimeNectar
```

and for pollen:

```
set tripDurationPollen 2 * distanceToColony * (1 / FLIGHT_VELOCITY )
                 + handlingTimePollen
```

Mortality rate:
The mortality risk during a foraging trip depends on the time spent outside the hive. For nectar foragers (and similar for pollen foragers) it is:

```
set mortalityRisk 1 - ((1 - MORTALITY_FOR_PER_SEC) ^ tripDuration)
```

Flight costs:
The flight costs for a nectar patch are based on two components, the energetic costs for flying to and returning from the patch and the energy spend at the patch while collecting nectar [kJ]:

```
let energyFactor_onFlower 0.2
set flightCostsNectar  (2 * distanceToColony * FLIGHTCOSTS_PER_m)
       + (FLIGHTCOSTS_PER_m * handlingTimeNectar
       * FLIGHT_VELOCITY * energyFactor_onFlower)
```

The local variable *energyFactor_onFlower* (0.2) takes into account that a bee saves energy while sitting on a flower to collect the nectar or pollen.

Energetic efficiency:
Based on the flight costs and the energy content of the nectar, the energetic efficiency (*Eef*) of a patch can be calculated, which is the ratio of energetic gain to energetic costs:

```
set EEF ((nectarConcFlowerPatch * CROPVOLUME
            * ENERGY_SUCROSE) - flightCostsNectar) / flightCostsNectar
```

Dance circuits:
The number of dance circuits performed by a returning nectar forager is then:

```
set danceCircuits DANCE_SLOPE * Eef + DANCE_INTERCEPT
```

with a maximal number of *Max_Dance_Circuits*. If *SimpleDancing* is *true* (default: false) then *danceCircuits* is set to 40 if the energetic efficiency is above 20 or to 0 if it is below. If *AlwaysDance* is *true* (default: false), then *danceCircuits* is set to 40, independent of the energetic efficiency. In this case is *SimpleDancing* irrelevant.

The mean number of dance followers of a nectar patch is calculated as:

```
set danceFollowersNectar danceCircuits * 0.05
```

based on the assumption that 0.05 foragers can be recruited per dance circuit (Seeley et al. 2005). *DanceFollowersNectar* is patch specific and updated every foraging round, as nectar collection by the foragers may affect the handling time and hence the energetic efficiency.

Foraging_start-stopProc

Here, the foragers decide if they take up, continue, or stop foraging and their preferences for nectar or pollen foraging are determined.

The local variable *forage_autocorr* (0) defines the degree of autocorrelation in the preference for nectar or pollen. With a high value, a forager switches only rarely from one food type to another, whereas with the default value of zero, every new food type choice is independent of earlier decisions. If a forager reconsiders its forage type, the preference for nectar or pollen foraging is based on the probability for pollen collection, *ProbPollenCollection*, calculated in ForagingRoundProc.
Those foragers that are currently active and were not recruited in the last foraging round, may stop foraging and set their activity to "resting", which is decided according to the constant probability *Foraging_Stop_Prob*.

Experienced foragers (i.e. those who currently know a pollen patch (*knownPollenPatch* >= 0), if their forage type is pollen or a nectar patch (*knownNectarPatch* >= 0), if their forage type is nectar) may abandon and forget their patch. The probability to abandon a pollen patch depends on the constant *Abandon_Pollen_Patch_Probability_Per_s* and on the trip duration. This is based on the assumption that a pollen patch is less attractive, and hence more likely to be abandoned, the lower the rate of collected pollen per unit of time is from that patch. If a pollen patch is abandoned, *knownPollenPatch* is then set to -1. If the forager was active, it becomes a scout (activity is set to "searching"). Each change in the activity is recorded in the foragers activity list. This process of abandoning a pollen patch is implemented as follows:

```
ask foragerSquadrons with [ knownPollenPatch >= 0 and pollenForager = true ]
  [ if random-float 1 < 1 - (1 -
      ABANDON_POLLEN_PATCH_PROB_PER_S) ^ [ tripDurationPollen ]
      of flowerPatch knownPollenPatch
    [set knownPollenPatch -1   ; Pollen foragers forget their known pollen patch
     ifelse ( activity != "resting" and activity != "lazy")
        [
          set activity "searching"
          set activityList lput "ApSp" activityList
        ] ; active foragers that abandoned their patch will search a new one
        [
          set activityList lput "Ap" activityList
        ]  ; no change in activity for resting or lazy foragers
    ]
  ]
```

This process is similarly repeated for nectar foragers, but their probability to abandon their nectar patch depends on the energetic efficiency of the patch and on the honey stores:

```
if random-float 1 < 1 / [ EEF ] of flowerPatch knownNectarPatch
      and random-float 1 < (HoneyEnergyStore / DecentHoneyEnergyStore)
```

Foragers with activity "resting" may then spontaneously start foraging with the probability *ForagingSpontaneousProb* (see section "Foraging Model", Start_IBM_ForagingProc):

```
ask foragerSquadrons with [ activity = "resting" ]
  [  if random-float 1 < ForagingSpontaneousProb...]
```

If they already know a flower patch of their current forage type preference, they are considered to be experienced foragers otherwise they become scouts:

```
if pollenForager = false  ; if they are nectar foragers..
      [
        ifelse knownNectarPatch >= 0 ; ..and if they know a nectar patch
          [
            set activity "expForaging" ; ..then they are experienced foragers
            set activityList lput "Xn" activityList
          ]
          [ ;if a nectar forager does not know a nectar patch, it becomes a scout:
            set activity "searching"
            set activityList lput "Sn" activityList
          ]
      ]
```

And similar for pollen foragers.

If the total distance a forager already travelled on that day is above *Max_Km_Per_Day*, then this forager will start resting.

All foraging activities in this and the following foraging procedures are recorded in the *activityList* of the forager, which can be used as output to exactly reconstruct what each forager has done in a foraging round. This was important for testing and debugging the program.

Foraging_searchingProc

In the procedure Foraging_searchingProc it is determined, if and, if yes, which flower patch is found by a searching scout.

In a first step, the probability is calculated that a scout will not find any patch at all:

```
ask flowerPatches with
     [ quantityMyl >= CROPVOLUME * SQUADRON_SIZE
       or amountPollen_g >= POLLENLOAD * SQUADRON_SIZE ]
[
  set probSum probSum + detectionProbability
  set cumulative_NON-detectionProb
      cumulative_NON-detectionProb * (1 - detectionProbability)
  set nowAvailablePatchesList fput who nowAvailablePatchesList
]
```

Only flower patches which have enough nectar or pollen left for at least one forage load are considered. The ID's ("who") of these patches are then recorded in the list *nowAvailablePatchesList*. *ProbSum* sums up the detection probabilities of all these patches, and is later required to determine, which patch will be found. The probability that any patch is found is then:

```
set TotalFPdetectionProb (1 - cumulative_NON-detectionProb)
```

If a scout is successful, it is randomly determined which of the available patches is found. For this purpose, all patches listed in *nowAvailablePatchesList* are addressed and their dectection probabilities are summed up in the variable *patchCounter*, until *patchCounter* exceeds *p* (a random variable between 0 and *probSum*). This very patch is then the one found by the scout. The ID ("who") of this patch is then used to set either *knownNectarPatch* or

knownPollenPatch - depending on the forage choice ("pollenForager" *true* or *false*) - of the scout:

```
let p random-float probSum
set patchCounter 0
set chosenPatch -1
foreach nowAvailablePatchesList
        [ ask flowerPatch ?
          [ set patchCounter patchCounter + detectionProbability
            if (patchCounter >= p) and (chosenPatch = -1)
               [ set chosenPatch who ]
          ]
        ]
```

An example: three flower patches are available, their detection probabilities are 0.1, 0.2 and 0.5. Hence, the probabilities that these patches are *not* detected are 0.9, 0.8 and 0.5 respectively and the probability that *none* of these patches is detected is 0.9 * 0.8 * 0.5 = 0.36. Assuming the scout finds a flower patch during its trip (probability 1 - 0.36 = 0.64), it is now determined which of these three patches is found: The random variable *p* gets a value between 0 and 0.8 (= *probSum*: 0.1 + 0.2 + 0.5), let's say 0.219. The detection probabilities are then added, until their sum (*patchCounter*) exceeds *p*: The detection probability of the first patch (0.1) is smaller than *p* (0.219), so detection probability of the second patch (0.2) is added. Now, *patchCounter* is 0.3, i.e. larger than *p* (0.219) and hence the second patch is the one found by the scout (i.e. *chosenPatch* is set to the ID (*who*) of the second patch).

If the food source a scout is searching for, is available, then the scout starts to exploit the patch, so either loads its crop with nectar or collect pollen and its activity is set to "bringingNectar" or " bringingPollen".

If the wanted food source is not available, because a nectar searching scout found a pollen-only patch or a pollen searching scout found a nectar-only patch, the bee remains a scout, its activity remains "searching" and the patch is forgotten (i.e. *knownNectarPatch* or *knownPollenPatch* is set to -1 again). If a scout has not found a patch at all, it also remains a scout with activity = "searching".

Not only scouts can find patches, but also recruits (i.e. foragers with *activity* = "recForaging"). However, recruits can only find the specific patch they are searching for (with a constant probability of *Find_Danced_Patch_Prob* and will not find a different patch. If they do not find this specific patch or it does not offer the wanted food source anymore, they forget this patch (*knownNectarPatch* or *knownPollenPatch* is set to -1) and their *activity* is set to "searching", so they will become scouts in the next foraging round.

If a scout or recruit has found a patch with the wanted food source, it starts to exploit it in the same way as experienced foragers do. This is described in the following section.

Foraging_collectNectarPollenProc

In this procedure, experienced foragers collect nectar or pollen from a flower patch. A forager is considered to be experienced (*activity* = "expForaging") after successfully returning from a nectar or pollen patch and it remains experienced until it forgets the known pollen patch (as a pollen forager) or the known nectar patch (as a nectar forager).

An experienced forager will exploit its patch if enough pollen or nectar is still available at the patch and its *activity* is set to "bringingNectar" or "bringingPollen" as soon as it has collected

its food load at the patch. The amount of pollen or nectar available at the patch is then reduced accordingly:

```
set quantityMyl (quantityMyl - ( CROPVOLUME * SQUADRON_SIZE))
```

for nectar foraging, and

```
set amountPollen_g (amountPollen_g - (POLLENLOAD * SQUADRON_SIZE))
```

for pollen foraging.

Successful foraging trips are counted (*NectarFlightsToday*, *PollenFlightsToday*) and again all activities are recorded in the foragers' activity list. If not enough nectar or pollen is left at the patch, the forager forgets the patch and becomes a scout, but it will not switch to the potential other food source. This is to ensure that bees choose their forage type according to the requirements of the colony and not according to its availability in the landscape. So a nectar forager, for example, arriving at an emptied food patch, would become a scout, searching for nectar. It would not collect pollen, even if pollen would be available at this patch.

If a nectar forager is successful, the energy content of its crop load calculated:

```
set cropEnergyLoad ([ nectarConcFlowerPatch ] of
       flowerPatch knownNectarPatch * CROPVOLUME * ENERGY_SUCROSE)
```

which depends on the constants *Cropvolume* and *Energy_Sucrose* and on the patch-specific sugar concentration of the nectar.

Successful pollen foragers get their pollen loads, which are set to the constant *Pollenload*. Subsequently both the travelled distances of the bees for today (*km_today*) and in total (*mileometer*) are updated and the colours of the foragers are updated to represent the patch they are foraging at. It can happen that a forager has the activity "expForaging" although it does not know a flower patch of its currently preferred forage type. This is the case if, for example, an experienced nectar forager, knowing a nectar patch but not a pollen patch, has switched at the beginning of this foraging round (in Foraging_collectNectarPollenProc) to pollen foraging. Then its activity is still "expForaging", *pollenForager* is set true for this forager but its *knownPollenPatch* is -1 (meaning unknown), so it cannot exploit a pollen patch. The *activity* of this forager is then set to "resting", its *km_today* and its *mileometer* are not changed and it is not exposed to the foraging mortality in this foraging round.

Foraging_flightCosts_flightTimeProc

This procedure sums up the travelled distances for unsuccessful scouts and calculates the cost of foraging flights for successful foragers and unsuccessful scouts. (Travelled distances for successful foragers are calculated in Foraging_collectNectarPollenProc, see previous section). The travelled distance of an unsuccessful scout is defined by the constant *Search_Length_m*, which is added to the bees' *mileometer* and *km_today*. *Search_Length_m* is also the basis to calculate the energy consumption of unsuccessful scouts, which is summed up in the local variable *energyConsumption*. Furthermore *Search_Length_m* is used to calculate the duration of a foraging trip, which is summed up in the global variable *ColonyTripDurationSum*.

To determine the energy consumption of successful nectar foragers, the foraging costs (*flightCostsNectar*) of the flower patch visited by the bees is used and added to

energyConsumption. Then the total duration of all foraging trips (*ColonyTripDurationSum*) is calculated by summing up the individual trip durations, i.e. for nectar foragers:

```
set ColonyTripDurationSum ColonyTripDurationSum + tripDuration + TIME_UNLOADING
```

and similar for pollen foragers.

Additionally, the total number of foraging trips during this foraging round is recorded (*ColonyTripForagersSum*) and later used to calculate the duration the foraging round.

Foraging_mortalityProc

This procedure determines the mortality of foragers during their foraging trip, counts the number of dying foragers, and calculates their lifespan.

The mortality for an unsuccessful scout is calculated as:

```
if random-float 1 < 1 - ((1 - MORTALITY_FOR_PER_SEC) ^ emptyTripDuration)
```

with *emptyTripDuration* [s] = *Search_Length_m* / *Flight_Velocity*

The mortality of successful nectar and pollen foragers is determined similarly, but the mortality risk in theses cases is a patch-specific variable (*mortalityRisk* or *mortalityRiskPollen*) which depends on the duration of a foraging trip, i.e. on the distance of the patch and the handling time (see next section).
Every death of a forager is counted (*DeathsForagingToday*) and the age of the dead bees summed up (*SumLifeSpanAdultWorkers_t*) to later calculate the average life span of the bees which died today.

Foraging_dancingProc

Successful nectar foragers will dance for a valuable patch to recruit up to five foragers to a nectar patch. Successful pollen foragers will always dance and recruit two foragers (*Pollen_Dance_Followers*) to a pollen patch.

Dancing is not associated with any costs in time or energy and principally all successful foragers dance, although not all of them will have dance followers. In the case of nectar foragers, the average number of dance followers is a flower patch specific variable (*danceFollowersNectar*). The number of dance followers is calculated in FlowerPatchesUpdateProc and depends on the energetic costs and gain of a foraging trip, and hence on sugar concentration, distance, and handling time of the patch (for details see above).

DanceFollowersNectar, a state variable of the flower patch that was visited by the forager, is used as a mean value to determine the actual number of dance followers (*danceFollowersNectarNow*) with the "random-poisson" function:

```
let danceFollowersNectarNow
        random-poisson [ danceFollowersNectar ] of flowerPatch knownNectarPatch
```

So even if *DanceFollowersNectar* (float variable) has a value < 1 the resulting *danceFollowersNectarNow* can be >= 1 (and will be an integer value).

However, dancing only takes place if at least *danceFollowersNectarNow* foragers with *activity* = "resting" are still available in the colony to follow a dance. Of these resting bees, *danceFollowersNectarNow* ("n-of") are addressed to follow the dance:

```
ask n-of  ([danceFollowersNectarNow] of flowerPatch knownNectarPatch)
               foragerSquadrons with [activity = "resting"]
```

They will be recruited to this patch in the next foraging round, if they do not know a nectar patch at all:

```
ifelse knownNectarPatch = -1
            [set knownNectarPatch patchNumberDanced
             set activity "recForaging"
             set pollenForager false]
```

or if the advertised nectar patch has a higher energetic efficiency than the nectar patch they know. Otherwise, i.e. if the dance following bee knows a more attractive nectar patch, this bee is not recruited to the advertised patch but will instead exploit its previously known patch in the next foraging round. Hence, its *activity* is set to "expForaging".

In contrast to dancing for nectar patches, pollen dances always attract a constant number of dance followers (*Pollen_Dance_Followers*), as we do not have data about how pollen patch properties, dance performance, and number of recruits are related. These pollen dance followers will be recruited to the advertised pollen patch, if they either do not know a pollen patch yet or if the total trip duration to the advertised patch is shorter than of the pollen patch they know.

Foraging_unloadingProc

The final step in each foraging round is the unloading of successful foragers resulting in the increase of the colony's food stores.

Successful nectar foragers increase the *HoneyEnergyStore* by their *cropEnergyLoad*, although it cannot exceed *Max_Honey_Energy_Store*. The *cropEnergyLoad* of these foragers is then set to zero and they are now considered to be experienced (*activity* is changed to "expForaging").

Similarly, successful pollen foragers increase *PollenStore_g* by *collectedPollen*, which is then set to zero and they are also experienced foragers now.

Again, all activities are recorded in the bees' *activityList*.

III - THE VARROA MODEL

Rationale: Varroa

Varroa destructor is an ectoparasitic mite, which switched from its original host, the Eastern honeybee (*Apis cerana*), to the Western honeybee (*A. mellifera*) (Anderson and Trueman 2000, reviewed in Rosenkranz et al. 2010). To reproduce, female mites enter the cells of drone or worker larvae, 2 days or 1 day before capping respectively (Boot et al. 1992). They prefer drone cells over worker cells for invasion (Boot et al. 1995). Once the cell is capped, the mite sucks hemolymph from the bee larva and starts its oogenesis. The first, unfertilized, egg will develop into a haploid male, all other eggs are fertilized and develop into females (Martin 1994, Martin 1995). Mite nymphs also feed on the bee's hemolymph, using small holes punctured by the mother (Donze and Guerin 1994, Kanbar and Engels 2003). Finally, the male mates with the females in the cell (Donze et al. 1996). The mites are released with the emerging adult bee, or if a dead bee pupa is removed from its cell, and become phoretic (Oldroyd 1999). The phoretic mites usually switch their host and prefer young worker bees (Kuenen and Calderone 1997). On the day of emergence, mites have a high risk of falling from the comb (Martin and Kemp 1997).
The main problem caused by Varroa are not the mites themselves but the viruses they transmit, especially the deformed wing virus (DWV) (Carreck et al. 2010). Viruses can spread from an infected mite to a bee pupa or adult bee and then to uninfected mites (Bowen-Walker et al. 1999, Martin 2001, Martin et al. 2010).

Early *Varroa* models ignored the effect of mites on the bee colony dynamics (e.g. Fries et al. 1994). Martin (2001) developed a combined the honeybee colony and *Varroa* model, where the mites act as vector for either the deformed wing virus or for the acute paralysis virus.

The BEEHAVE *Varroa* module follows, with a few exceptions, the mite model of Martin (2001). We use the survivorship curves from Martin (2001) to derive the daily mortality rates of healthy and infected bees (see also section 7.7. "Development adults").

The varroa model simulates all processes related to varroa mites and varroa transmitted viruses. Based on an initial number of phoretic mites, the invasion into brood cells, reproduction of mites, and mite mortality are calculated as well as spreading of viruses from mites to bees and vice versa. The main procedure of this module is MiteProc, which calls all other mite-related procedures. We distinguish between phoretic mites, represented by the global variable *PhoreticMites*, and mites that entered honeybee brood cells for reproduction. These mites in brood cells are represented in the auxiliary entity *miteOrganiser* (see section 2, "Entities, state variables, and scales").

7.III.1. CreateMiteOrganisersProc

This procedure initialises a new *miteOrganiser*. A *miteOrganiser* keeps track of one worker and one drone brood cohort (both of the same age), and records how many of their brood cells are infested with 0, 1, 2 or more mites. A *miteOrganiser* is displayed on the interface in the shape of a varroa mite between the corresponding drone and worker brood cohort.

7.III.2. MitesInvasionProc

To reproduce, phoretic mites have to enter worker or drone larval cells of a specific age (*Invading_Workers_Cells_Age*, *Invading_Drone_Cells_Age*). We follow Boot et al. (1995) to calculate the invasion rates for worker and drone cells (rW, rD), i.e. the probabilities of a phoretic mite entering a worker or drone brood cell. Detailed justification of this approach can also be found in Martin (1998, 2001) and Calis et al. (1999). The invasion rates depend on the

number of brood cells suitable for invasion (i.e. 1 or 2 days before capping) and the number of adult worker bees:

```
ask larvaeCohorts with [ age = INVADING_WORKER_CELLS_AGE ]
  [ set suitableWorkerCells number ]
ask droneLarvaeCohorts with [ age = INVADING_DRONE_CELLS_AGE ]
  [ set suitableDroneCells number ]
```

The cell type specific invasion rates are then:

```
let adultsWeight_g (TotalIHbees + TotalForagers) * WEIGHT_WORKER_g
set rW factorWorkers * (suitableWorkerCells / adultsWeight_g)
set rD factorDrones * (suitableDroneCells / adultsWeight_g)
```

with the local variables *factorWorkers* = 0.56 and *factorDrones* = 6.49. The overall probability of invading any brood cell is:

```
set invadingBroodCellProb (1 - (exp (-(rW + rD))))
```

InvadingBroodCellProb is then used to randomly determine for each phoretic mite if it will enter a brood cell:

```
repeat PhoreticMites [ if random-float 1 < invadingBroodCellProb [..] ]
```

If a brood cell was entered, the cell type is determined using the probability *invadingWorkerCellProb* for worker cell invasion:

```
set invadingWorkerCellProb (rW / (rW + rD))
```

Otherwise, a drone cell is invaded. The total number of mites invading brood cells are then summed up (*InvadingMitesWorkerCellsTheo*, *InvadingMitesDroneCellsTheo*). The number of mites in cells may later be reduced to avoid crowding (see below).

To allow representing multiple infestations of a brood cell, we temporarily create individual brood cells. *WorkerCellListTemporary* and *droneCellListTemporary* record the number of invading mites (example for worker cells, but similar for drone cells):

```
let workerCellListTemporary n-values suitableWorkerCells [ 0 ]
```

If, for example, 87 suitable worker cells exist, the list contains 87 items (numbered 0…86), each item counts the number of mites invading the corresponding cell. If a phoretic mite invades a worker brood cell, then the invaded *cell* is randomly chosen from the *workerCellListTemporary* list and the number of mites in this cell is increased by one:

```
set cell random suitableWorkerCells
set WorkerCellListTemporary replace-item cell WorkerCellListTemporary
            (item cell WorkerCellListTemporary + 1)
```

This procedure might occasionally lead to unrealistically high numbers of mites in a cell, therefore the number of mites in each brood cell is reduced to its maximum *Max_Invaded_Mites_Workercell* and the removed mites (*exitingMites*) become phoretic again. The remaining mites invading worker brood cells are then *InvadingMitesWorkerCellsReal* and the number PhoreticMites is updated accordingly:

```
set PhoreticMites PhoreticMites - InvadingMitesWorkerCellsTheo
        - InvadingMitesDroneCellsTheo + exitingMites
```

These processes are repeated in the same way for drone cells.

The list *workerCellListTemporary* contains information about individual brood cells, but what matters for the dynamics of the model populations of mites and bees is only how many brood cells were invaded by 0, 1, or more mites, because these numbers determine both the reproduction of mites and the bee's risk of becoming infected by mite transmitted viruses. Thus, for the auxiliary entity *miteOrganiser*, the number of worker cells invaded by zero, one, two etc. mites is extracted from *WorkerCellListTemporary* into *workerCellListCondensed* (similar for drone cells):

```
ask miteOrganisers with [age = INVADING_WORKER_CELLS_AGE]
  [ foreach workerCellListTemporary
    [ set workerCellListCondensed replace-item ? workerCellListCondensed
        ((item ? workerCellListCondensed) + 1)] ... ]
```

The age of a *miteOrganiser* is set at its creation in CreateMiteOrganisersProc to either *Invading_Workers_Cells_Age* or to *Invading_Drone_Cells_Age* if the latter is smaller; it increases every day by 1. The resulting *workerCellListCondensed* may then look, for example, like [316 32 4 0 0], which refers to 316 cells without mites, 32 cells invaded by a single mite and four cells invaded by two mites, so altogether 40 mites would be present in this worker larvae cohort.

The ID (*who*) of the invaded larvae cohort is recorded in the miteOrganisers' *invadedWorkerCohortID* and the ID of miteOrganiser is recorded in the invaded larvae cohort as *invadedByMiteOrganiserID*. So both entities are unambiguously linked with each other.

7.III.3. MitePhoreticPhaseProc

In this procedure the distribution of new, emerged phoretic mites among adult worker bees is calculated as well as the transmission of viruses between mites and bees. If an in-hive bee dies or develops into a forager, then her mites jump to another in-hive bee.

In a first step, the numbers of "healthy" (i.e. not infected with a virus) and virus-infected mites are calculated:

```
let healthyPhoreticMites round (PhoreticMites * PhoreticMitesHealthyRate)
let infectedPhoreticMites PhoreticMites - healthyPhoreticMites
```

Then, the average number of mites on in-hive bees is determined, using numbers of mites and bees, *before* the new mites were released (in MitesReleaseProc, see below) or in-hive bees died or developed into foragers:

```
set phoreticMitesPerIHbee
       ( PhoreticMites - NewReleasedMitesToday)
         / (TotalIHbees + InhivebeesDiedToday
            + SQUADRON_SIZE *
               ( NewForagerSquadronsHealthy
                 + NewForagerSquadronsInfectedAsPupae
                 + NewForagerSquadronsInfectedAsAdults
               )
           )
```

Next, if in-hive bees die or develop into foragers, the phoretic mites sitting on them are "released" from their current host bee:

```
let mitesReleasedFromInhivebees
    phoreticMitesPerIHbee
         * ( InhivebeesDiedToday
             + SQUADRON_SIZE
              * ( NewForagerSquadronsHealthy
```

```
                    + NewForagerSquadronsInfectedAsPupae
                    + NewForagerSquadronsInfectedAsAdults
                  )
              )
```

Then they have to switch to another in-hive bee. Additionally, also the mites newly released from brood cells have to find a new host bee. We distinguish between healthy and infected mites (calculations for both cases are similar) and the total number of healthy mites that will jump on an in-hive bee is then:

```
let healthyPhoreticMitesSwitchingHosts
    round( mitesReleasedFromInhivebees * PhoreticMitesHealthyRate
           + PhoreticMites * PropNewToAllPhorMites * PhoreticMitesHealthyRate)
```

where *PropNewToAllPhorMites* describes the proportion of newly emerged phoretic mites to all phoretic mites.

To determine the number of phoretic mites that are newly infected with the virus because they switched to an infected in-hive bee, we count the number of healthy in-hive bees and the number of infected (as pupae or as adults) in-hive bees (*totalInfectedWorkers*, *totalHealthyWorkers*). The probability of a healthy mite becoming newly infected equals the proportion of infected in-hive workers (assuming an always successful transmission of the virus from the bee to the mite):

```
repeat healthyPhoreticMitesSwitchingHosts
  [
   if random-float 1 <  totalInfectedWorkers /
                          (totalInfectedWorkers + totalHealthyWorkers)
        [
          set newlyInfectedMites newlyInfectedMites + 1
        ]
      ]
```

However, also healthy in-hive bees can be infected when they are newly infested by infected mites. These infected mites are either freshly released from brood cells or have left a bee that died in the colony or developed into a forager:

```
let allInfectedMitesSwitchingHosts
    round (PhoreticMites * PropNewToAllPhorMites * (1 - PhoreticMitesHealthyRate)
           + mitesReleasedFromInhivebees * (1 - PhoreticMitesHealthyRate))
```

These infected mites are then distributed over the in-hive worker cohorts:

```
let infectedMitesSwitchingHostsInThisCohort
        (allInfectedMitesSwitchingHosts / TotalIHbees) *  number
```

where *number* refers to the cohort size.

The number of healthy bees that become newly infected in this cohort (assuming an always successful transmission of the virus from the mite to the bee) is then determined by:

```
let newlyInfectedIHbeesInThisCohort 0
repeat number_healthy
  [ if random-float 1 > (1 - (1 / number))
         ^ infectedMitesSwitchingHostsInThisCohort
      [ set newlyInfectedIHbeesInThisCohort newlyInfectedIHbeesInThisCohort + 1 ]
        set infectedMitesSwitchingHostsInThisCohort
                    infectedMitesSwitchingHostsInThisCohort - 1
        if infectedMitesSwitchingHostsInThisCohort < 0
             [ set infectedMitesSwitchingHostsInThisCohort 0 ]
```

```
  ]
```

where *number* is the total number of bees in this in-hive cohort and hence 1 / *number* reflects the probability that a bee will be infested by a mite. As this process is based on probabilities, it is possible that no bees becomes infected, even if *infectedMitesSwitchingHostsInThisCohort* is larger than 0.

Finally, the number of healthy and infected bees and mites and the *PhoreticMitesHealthyRate* are updated.

7.III.4. MiteDailyMortalityProc

In this procedure, the daily mortality of phoretic mites is calculated. If brood is present, then the mortality risk for a mite is *Mite_Mortality_Broodperiod* and the number of surviving phoretic mites is calculated as:

```
set PhoreticMites (PhoreticMites - random-poisson (PhoreticMites *
                                                   MITE_MORTALITY_BROODPERIOD))
```

If no brood is present, then the mortality risk for a mite is *Mite_Mortality_Winter* and the number of surviving phoretic mites is calculated in a similar way. Note that mortality of mites caused by varroa treatment is addressed in BeekeepingProc.

7.III.5. MiteOrganisersUpdateProc

Here the *MiteOrganisers* age by one day and their representation on the interface moves on one step. The number of all mites in brood cells is counted and *TotalMites* is updated. If the worker and drone brood cohort connected to the *miteOrganiser* has emerged, the miteOrganiser is deleted ('dies').

7.III.6. MitesReleaseProc

This is a key submodel for the interaction between mites and honeybees and the corresponding virus infection. This procedure is called whenever mites are released from drone or worker brood cells, either because brood dies or adult bees emerge. This is the case in the following procedures (one or several times): WorkerLarvaeDevProc (dying brood), DroneLarvaeDevProc (dying brood), WorkerPupaeDevProc (2x, for dying & emerging brood), DronePupaeDevProc (2x, for dying & emerging brood), and BroodCareProc (4x, for dying worker larvae and pupae and dying drone larvae and pupae). At the end of the procedure, the number of phoretic mites, the proportion of healthy phoretic mites and the number of brood cells are updated.

If mites are released because adult bees emerge, then mites can produce offspring. If mites are released because brood has died, no mite reproduction takes place. Infection of pupae with mite-transmitted viruses and subsequent survival of the bees is also determined in this procedure. A key feature of this submodel is, as with MitesInvasionProc, that in BEEHAVE brood cells are not represented individually. However, just multiplying, e.g., average numbers of invading and released mites would be too coarse, in particular since a single virus-infected mite can make a difference. Therefore, in MitesInvasionProc, brood cells are temporarily represented individually, and in turn here, when mites are released, processes like mite reproduction or virus transmission depend on the number of mites that invaded a cell and on their virus infection.

MitesReleaseProc has the following variables as input: *miteOrganiserID* (the ID (*who*) of the *miteOrganiser* to be addressed), *ploidyMiteOrg* (the ploidy of the brood i.e. 1 for drone brood or 2 for worker brood), *diedBrood* (number of died brood cells) and *releaseCausedBy* (cause

of the mite release: "emergingBrood" or "dyingBrood"). The local variable *cellListCondensed* contains the information on the mite distribution over the brood cells of the relevant cohort, i.e. it is a copy of *workerCellListCondensed*, if *ploidyMiteOrg* is one or of *droneCellListCondensed*, if *ploidyMiteOrg* is two.

After counting the total number of brood cells (*totalCells*) in the relevant brood cohort, the number of cells to be uncapped (*uncappedCells*) is determined. This is either *totalCells* (if *releaseCausedBy* is "emergingBrood") or it is *diedBrood* (if *releaseCausedBy* is "dyingBrood").

Now the number of mites released from each of these uncapped cells is determined. We temporarily individualise the brood cells and randomly chose one of them (*randomCell*) as the next one to be uncapped:

```
repeat uncappedCells
  let randomCell (random totalCells) + 1
  let cellCounter 0
  let allMitesInSingleCell -1
  [ while [ cellCounter < randomCell ]
     [ set allMitesInSingleCell allMitesInSingleCell + 1
       set cellCounter cellCounter + (item allMitesInSingleCell cellListCondensed)
     ]
  ...
  set totalCells totalCells - 1
  ]
```

This process is based on the idea that the cells of the current cohort are ordered according to the number of mites they are invaded by. This list of ordered cells is represented by *cellListCondensed*, (with the first item being the number of cells invaded by 0 mites, the second item are the cells invaded by 1 mite, etc.). A random number (0…total number of cells in this cohort) determines a cell, which will now be uncapped and the information we need to get is, by how many mites this cell is invaded. So, the while-loop sums up the items of *cellListCondensed* until the sum is larger, or equal to, the randomly chosen cell and the rank of the item in *cellListCondensed* reflects then the number of mites that invaded this particular cell.
For example, *cellListCondensed* is [100 10 5 1 0], so 100 cells are without mites, 10 cells are invaded by a single mite, 5 cells invaded by 2 mites etc. and *totalCells* is 116. A random cell (1..116) is then chosen, e.g. 112. In the first repetition of the while-loop *allMitesInSingleCells* is set from -1 to 0, i.e. the number of cells without mites is added to *cellCounter*, which is changed from 0 to 100. As 100 is smaller than 112, there is another repetition of the while-loop, adding the number of cells invaded by a single mite (10), so *cellCounter* is now 110. In the third loop finally, *cellCounter* is set to 115, which exceeds 112, and the number of invaded mites is 2.

Now, the number of mites released from the currently uncapped cell is known (=*allMitesInSingleCell*). However, these mites only represent the mother mites in the cells. The mother mites may have reproduced, if *releaseCausedBy* is "emergingBrood" and if the infested pupa has not died due to a virus infection caused by an infected mite. So in the next step it is determined for each of these mother mites in the cell, if they are infected by a virus (*infectedMitesInSingleCell*):

```
repeat allMitesInSingleCell
    [ if random-float 1 > healthyRateMiteOrg
      [ set infectedMitesInSingleCell infectedMitesInSingleCell + 1]
    ]
```

where *healthyRateMiteOrg* represents the proportion of healthy phoretic mites on the day the brood cohort was invaded by the mites. The remaining mites in the brood cell are *healthyMitesInSingleCell*.

It is now determined if the pupa was infected by invaded mites, which depends on the *Virus_Transmission_Rate_Mite_To_Pupa* and on the number of infected mites in the cell (*infectedMitesInSingleCell*):

```
if random-float 1 >
   (1 - VIRUS_TRANSMISSION_RATE_MITE_TO_PUPA) ^ infectedMitesInSingleCell
   [ set pupaInfected true ]
```

If the pupa was infected, it dies with the probability *Virus_Kills_Pupa_Prob*:

```
if pupaInfected = true
    [ if random-float 1 < VIRUS_KILLS_PUPA_PROB
      [ set pupaAlive 0 ] ]
```

The pupa is also dead (i.e. *pupaAlive* = 0) if *releaseCausedBy* is "dyingBrood". If the pupa has died due to virus infection, the number of bees (healthy and total) in the cohort is reduced by one.

If the pupa was infected but survives, the number of healthy bees (*number_healthy*) is reduced by one, whereas *number_infectedAsPupa* is increased by one.

Then the average number of offspring per mother mite in the cell is determined, using the reporter procedures MiteOffspringREP (with *ploidyMiteOrg* as input) and MiteDensityFactorREP (with *ploidyMiteOrg* and *mitesIndex* as input):

```
let averageOffspring
   random-poisson (MiteOffspringREP ploidyMiteOrg
                * MiteDensityFactorREP ploidyMiteOrg mitesIndex)
```

In MiteOffspringREP the reproduction rate of the varroa mites is determined, where *ploidyMiteOrg* is an input variable that reflects if the invaded cell is a worker or drone brood cell. The actual reproduction rates depend on the study the data is based on e.g. "Fuchs&Langenbach" or "Martin" (*MiteReproductionModel*).

The mite reproduction rate is reduced, if a brood cell is infested by more than one mite. This effect is considere in MiteDensityFactorREP. Based on the *MiteReproductionModel*, the brood cell type (*ploidyMiteOrg*), and the number of invaded mites (*mitesIndex*), the actual reproduction rate is determined. E.g. the density factors for *MiteReproductionModel* = "Martin" are 0, 1, 0.91, 0.86, and 0.60 for 0, 1, ..4 mites respectively.

The total number of mites in the brood cell is then the healthy mother mites plus the infected mother mites plus the (healthy) offspring of all mother mites:

```
set healthyMitesInSingleCell
         healthyMitesInSingleCell +
         allMitesInSingleCell * averageOffspring * pupaAlive
set healthyMitesInSingleCell round healthyMitesInSingleCell
set allMitesInSingleCell healthyMitesInSingleCell + infectedMitesInSingleCell
```

All mite offspring are healthy at first, but they can become infected the same way as healthy mother mites by an infected pupa with a probability of *Virus_Transmission_Rate_Pupa_To_Mites*:

```
if pupaAlive = 1 and pupaInfected = true
    [ repeat healthyMitesInSingleCell
      [ if random-float 1 < VIRUS_TRANSMISSION_RATE_PUPA_TO_MITES
        [ set healthyMitesInSingleCell healthyMitesInSingleCell - 1
          set infectedMitesInSingleCell infectedMitesInSingleCell + 1
        ] ] ]
```

Newly emerged mites may fall from the comb and will be considered dead with a probability of *Mite_Fall_Dronecell* or *Mite_Fall_Workercell*, depending on the brood cell type they emerge from. This is done for healthy and infected mites separately; their numbers are updated and total mite fall is recorded in *DailyMiteFall*.

PhoreticMites as well as some local variables keeping track of emerging mites are updated and finally, the cell is removed from the *cellListCondensed*, depending on the number of mites that had invaded the cell (*mitesIndex*):

```
set cellListCondensed replace-item mitesIndex cellListCondensed
      (item mitesIndex cellListCondensed - 1)
set totalCells totalCells - 1
```

These processes (i.e. determination of the number of mother mites in the cell, infection states of mites and pupa, survival of pupa and reproduction of mites) are then repeated for all brood cells, which have to be uncapped (*uncappedCells*).

At the end of the procedure, the proportion of healthy phoretic mites (*PhoreticMitesHealthyRate*) is recalculated.

8. NETLOGO CODE

```
; breeds, globals & owns
breed [ hives hive ]
breed [ eggCohorts eggCohort]
breed [ larvaeCohorts larvaeCohort]
breed [ pupaeCohorts pupaeCohort]
breed [ IHbeeCohorts IHbeeCohort]  ; in-hive bee
breed [ droneEggCohorts droneEggCohort]
breed [ droneLarvaeCohorts droneLarvaeCohort]
breed [ dronePupaeCohorts dronePupaeCohort]
breed [ droneCohorts droneCohort]
breed [ foragerSquadrons foragerSquadron ]
  ; small group of foragers, groupsize: SQUADRON_SIZE
breed [ miteOrganisers miteOrganiser ]
  ; keep track of mites in brood cells
breed [ flowerPatches flowerPatch]
breed [ Signs Sign ]
  ; signs to inform the user


globals
 [
  ABANDON_POLLEN_PATCH_PROB_PER_S
  AFF
  AFF_BASE
  AllDaysAllPatchesList
  BugAlarm
  ColonyDied
```

ColonyTripDurationSum
ColonyTripForagersSum
CROPVOLUME
CumulativeHoneyConsumption
DailyForagingPeriod
DailyHoneyConsumption
DailyMiteFall
DailyPollenConsumption_g
Day
DeathsAdultWorkers_t
DeathsForagingToday
DecentHoneyEnergyStore
DRONE_EGGLAYING_START
DRONE_EGGLAYING_STOP
DRONE_EMERGING_AGE
DRONE_HATCHING_AGE
DRONE_LIFESPAN
DRONE_PUPATION_AGE
DRONE_EGGS_PROPORTION
EMERGING_AGE
EmptyFlightsToday
ENERGY_HONEY_per_g
ENERGY_SUCROSE
ExcessBrood
FIND_DANCED_PATCH_PROB
FLIGHT_VELOCITY
FLIGHTCOSTS_PER_m
FORAGER_NURSING_CONTRIBUTION
FORAGING_STOP_PROB
ForagingRounds
ForagingSpontaneousProb
HarvestedHoney_kg

HATCHING_AGE
HONEY_STORE_INIT
HoneyEnergyStore
HoneyEnergyStoreYesterday
HoPoMo_seasont
IdealPollenStore_g
InhivebeesDiedToday
INVADING_DRONE_CELLS_AGE
INVADING_WORKER_CELLS_AGE
InvadingMitesDroneCellsReal
  ; actual number of mites invading the cells, might be
  ; lower than theor. number, if brood cells are crowded with mites
InvadingMitesDroneCellsTheo
  ; theoretical number of mites invading the cells
InvadingMitesWorkerCellsReal
InvadingMitesWorkerCellsTheo
LIFESPAN
LostBroodToday
  ; brood that die due to lack of nursing or lack of pollen today
LostBroodTotal ; .. and summed up
MAX_AFF
MAX_BROOD_NURSE_RATIO
MAX_DANCE_CIRCUITS
MAX_EGG_LAYING
MAX_HONEY_ENERGY_STORE
MAX_INVADED_MITES_DRONECELL
MAX_INVADED_MITES_WORKERCELL
MAX_PROPORTION_POLLEN_FORAGERS
MAX_TOTAL_KM
MIN_AFF
MIN_IDEAL_POLLEN_STORE
MITE_FALL_DRONECELL

MITE_FALL_WORKERCELL
MITE_MORTALITY_BROODPERIOD
MITE_MORTALITY_WINTER
MORTALITY_DRONE_EGGS
MORTALITY_DRONE_LARVAE
MORTALITY_DRONE_PUPAE
MORTALITY_DRONES
MORTALITY_DRONES_INFECTED_AS_PUPAE
MORTALITY_EGGS
MORTALITY_FOR_PER_SEC
MORTALITY_INHIVE
MORTALITY_INHIVE_INFECTED_AS_ADULT
MORTALITY_INHIVE_INFECTED_AS_PUPA
MORTALITY_LARVAE
MORTALITY_PUPAE
N_FLOWERPATCHES
N_GENERIC_PLOTS
NectarFlightsToday
NewDroneEggs
NewDroneLarvae
NewDronePupae
NewDrones
NewDrones_healthy
NewForagerSquadronsHealthy
NewForagerSquadronsInfectedAsAdults
NewForagerSquadronsInfectedAsPupae
NewIHbees
NewIHbees_healthy
NewReleasedMitesToday
  ; all (healthy and infected) mites released from cells (mothers+offspring)
  ; on current day (calculated after MiteFall!)
NewWorkerEggs

NewWorkerLarvae
NewWorkerPupae
PATCHCOLOR
PhoreticMites ; all phoretic mites, healthy and infected
PhoreticMitesHealthyRate
POLLEN_DANCE_FOLLOWERS
POLLEN_STORE_INIT
PollenFlightsToday
POLLENLOAD
PollenStore_g
PollenStore_g_Yesterday
POST_SWARMING_PERIOD
PRE_SWARMING_PERIOD
ProbPollenCollection
PropNewToAllPhorMites
PROTEIN_STORE_NURSES_d
ProteinFactorNurses
Pupae_W&D_KilledByVirusToday
; number of drone + worker pupae that were killed by the virus today
PUPATION_AGE
Queenage
RecruitedFlightsToday
SaveInvadedMODroneLarvaeToPupae
SaveInvadedMOWorkerLarvaeToPupae
SaveWhoDroneLarvaeToPupae
SaveWhoWorkerLarvaeToPupae
SEARCH_LENGTH_M
SearchingFlightsToday
SEASON_START ; defines beginning of foraging period
SEASON_STOP ; end of foraging period & latest end of drone production
SimpleDancing
STEPWIDTH

STEPWIDTHdrones
SumLifeSpanAdultWorkers_t
SummedForagerSquadronsOverTime
SwarmingDate
TIME_UNLOADING
TIME_UNLOADING_POLLEN
TodaysAllPatchesList
TodaysSinglePatchList
TotalBeesAdded
  ; beekeeper can add bees in autumn, these are added up as long
  ; as simulation runs
TotalDroneEggs
TotalDroneLarvae
TotalDronePupae
TotalDrones
TotalEggs
TotalEventsToday ; sum of todays "xxxFlightsToday"
TotalForagers
TotalFPdetectionProb
TotalHoneyFed_kg
  ; if "beekeeper" has to feed the colony, fed honey is added up as long
  ; as simulation runs
TotalHoneyHarvested_kg
TotalIHbees
TotalLarvae
TotalMites
TotalPollenAdded
  ; beekeeper can add pollen in spring, which is added up as long
  ; as simulation runs
TotalPupae
TotalWeightBees_kg ; weight of all bees (brood, adults, drones..)
TotalWorkerAndDroneBrood

```
  VIRUS_KILLS_PUPA_PROB
  VIRUS_TRANSMISSION_RATE_MITE_TO_PUPA
    ; probability for an infected invaded mite to infect the bee pupa
  VIRUS_TRANSMISSION_RATE_PUPA_TO_MITES
    ; probability for an infected bee pupa to infect healthy invaded mites
  WEIGHT_WORKER_g
]


turtles-own
; all cohorts below have these variables too
[
  age
  ploidy
  number
  numberDied
  invadedByMiteOrganiserID
]


pupaeCohorts-own
[
  number_infectedAsPupa
  number_healthy
]


dronePupaeCohorts-own
[
  number_infectedAsPupa
  number_healthy
]
```

IHbeeCohorts-own
[
  number_infectedAsPupa
  number_infectedAsAdult
  number_healthy
  ]

droneCohorts-own
[
  number_infectedAsPupa
  number_healthy
]

foragerSquadrons-own
[
  activity
  activityList
  knownNectarPatch
  knownPollenPatch
  pollenForager
  cropEnergyLoad
  collectedPollen
  mileometer
  km_today
  infectionState
]

flowerPatches-own
[
  patchType

distanceToColony
xcorMap
ycorMap
oldPatchID
size_sqm
quantityMyl
amountPollen_g
nectarConcFlowerPatch
detectionProbability
flightCostsNectar
flightCostsPollen
EEF
danceCircuits
danceFollowersNectar
summedVisitors
nectarVisitsToday
pollenVisitsToday
tripDuration
tripDurationPollen
mortalityRisk
mortalityRiskPollen
handlingTimeNectar
handlingTimePollen
]

miteOrganisers-own
[
workerCellListCondensed
droneCellListCondensed
cohortInvadedMitesSum
invadedMitesHealthyRate
invadedDroneCohortID

```
  invadedWorkerCohortID
]


; =========== BUTTONS =========================================================
; *****************************************************************************


to Setup
; BUTTON!

  clear-all
  set N_INITIAL_BEES round N_INITIAL_BEES
  set N_INITIAL_MITES_HEALTHY round N_INITIAL_MITES_HEALTHY
  set N_INITIAL_MITES_INFECTED round N_INITIAL_MITES_INFECTED
  reset-ticks
  if ReadInfile = true [ ReadFileProc ]
  ParameterizationProc
  ifelse ReadInfile = false
    [ CreateFlowerPatchesProc ]
       ; IF: flower patches are defined by input fields in GUI
    [ Create_Read-in_FlowerPatchesProc ]
       ; ELSE: or read in from a text file

  CreateImagesProc
  if (Experiment = "Experiment A") or (Experiment = "Experiment B")
    [
      user-message "Please make sure experimental colony conditions are defined in Setup and GoTreatmentProc"
      ;(INSERT INITIAL CONDITIONS FOR EXPERIMENTAL COLONIES HERE)
      GoTreatmentProc
    ]
```

```
end

; ************************************************************************************************************


to CreateOutputFileProc
  ; BUTTON!  writes data in file, copied from:
  ; Netlogo: Library: Code Examples: Output_Example.nlogo

  set WriteFile true
  let filename "Output.txt"
  if is-string? filename  ; to make sure filename is a string
  [
    if file-exists? filename   ; if the file already exists, it is deleted
    [
      file-delete filename
    ]
    file-open filename
    WriteToFileProc  ; record the initial turtle data
  ]
end

; ************************************************************************************************************
; ************************************************************************************************************


to StartProc
  ; called by Day/Month/Year/x days and RUN Button
```

```
  if BugAlarm = true
  [
    ask patches
    [
      set pcolor red
    ]
    user-message ("BUG ALARM!! (Start Proc)")  stop
      ; programm is stopped, if an "assertion" is violated, background becomes red
  ]

  if (stopDead = true) and (ColonyDied = true) [ stop ]
    ; programm is stopped, if colony is dead and stopDead switch is "On"

  Go ; <<<<<<<<<<<<<<<<<<<<<<<<<<<<

  if WriteFile = true [ WriteToFileProc ]
    ; results are recorded in Output
    ; file after each timestep

end ; StartProc

;===========================================================================================
; *******************************************************************************************


to ParameterizationProc

; BROOD CARE
  set FORAGER_NURSING_CONTRIBUTION  0.2
  set MAX_BROOD_NURSE_RATIO  3
    ; 3  (3: Free & Racey 1968) (Becher et al. 2010: 2.65)
    ; # brood that can be raised by a single "nurse" bee ("nurse": IH-bee and
```

```
  ; to some degree also foragers!, see FORAGER_NURSING_CONTRIBUTION)

; COLONY
 set ColonyDied  FALSE
 set DRONE_EGGS_PROPORTION  0.04
  ; 0.04  Wilkinson&Smith 2002 (from Allen 1963, 1965)

 set MIN_IDEAL_POLLEN_STORE  250
  ; 250  [g] min. amount of pollen that a colony tries to store

 set POLLEN_STORE_INIT  100
  ; 100 [g] pollen present on 1st day of simulation

 set PRE_SWARMING_PERIOD  3
  ;  HoPoMo: 3d, see also Winston p. 184

 set PROTEIN_STORE_NURSES_d  7
  ;  7 [d] Crailsheim 1990

 set ProteinFactorNurses  1
  ; 0..1, is daily calculated in PollenConsumptionProc, reflects protein
  ; content of brood food

 set Queenage  230        ;  queen emerged mid of May

 set WEIGHT_WORKER_g  0.1
  ; 0.125  0.1 or 0.11 or 0.125
  ; (0.1: HoPoMo 0.11: ; Martin 1998: 1kg adults = 9000 bees)
  ; (0.125: Calis et al. 1999) higher weight => less mites!
```

```
; DEVELOPMENT
  set AFF_BASE  21       ; like BEEPOP
  set MIN_AFF 7  ; Robinson 1992: 7d; see also: Winston 1987, p. 92/93
    ; models: Amdam & Omholt 2003, Beshers et al 2001: 7d
  set MAX_AFF 50
    ; within range given in Winston 1987, p. 92/93
  set DRONE_EGGLAYING_START  115
    ;  115: 25.April (Allen 1963: late April ..late August)
  set DRONE_EGGLAYING_STOP  240
    ; 240  240: 28.August (Allen 1963: late April ..late August)
  set DRONE_HATCHING_AGE  3     ; Jay 1963, Hrassnig, Crailsheim 2005
  set DRONE_PUPATION_AGE  10    ; i.e. capping of the cell; Fukuda, Ohtani 1977
  set DRONE_EMERGING_AGE  24
  set HATCHING_AGE  3           ;  Winston p. 50
  set PUPATION_AGE  9    ; i.e. capping of the cell
  set EMERGING_AGE  21
  set MAX_EGG_LAYING 1600        ; 1600  max. # eggs laid per day


; ENVIRONMENT
  set SEASON_START  1       ; season: 1st January - 31st December, i.e.
  set SEASON_STOP  365      ; foraging potentially possible throughout the year (weather depending)
  set ABANDON_POLLEN_PATCH_PROB_PER_S  0.00002


; FORAGING
  set CROPVOLUME  50
    ; 50   [microlitres] (~50mg nectar) Winston (1987), NuÃ±ez (1966, 1970), Schmid-Hempel et al. (1985)
  set FIND_DANCED_PATCH_PROB  0.5; (0.5 = ca. average of reported values):
    ; Seeley 1983: recruits required 4.8 dance-guided search trips to find target patch = 0.21
    ; Judd 1995: of 63 dance followers, 16 were successful, 16/63 = 0.25
    ; Biesmeijer, deVries 2001: review: 0.95 (Oettingen-Spielberg 1949), 0.73 (Lindauer 1952)
```

```
set FLIGHT_VELOCITY  6.5
  ; 6.57084    [m/s] derived from Seeley 1994, mean velocity
  ; during foraging flight see also Ribbands p127: 12.5-14.9mph (*1.609=20.1-24.0 km/h =
  ; 5.58-6.66m/s)

set FLIGHTCOSTS_PER_m 0.000006   ;
  ; [kJ/m] Flightcosts per m (Goller, Esch 1990: 0.000006531 kJ/m,  (assuming speed of 6.5m/s:
  ; flight costs: 0.0424W - compare with Schmid-Hempel et al. 1985: 0.0334W => 0.000005138 )

set FORAGING_STOP_PROB  0.3

set MAX_DANCE_CIRCUITS  117                  ;  (117) (Seeley, Towne 1992)
set MAX_PROPORTION_POLLEN_FORAGERS  0.8       ;  (0.8: Lindauer 1952)
set POLLEN_DANCE_FOLLOWERS  2     ; 2: number of bees, following a pollen dancer
set POLLENLOAD  0.015
  ; [g] 0.015g average weight of 2 pollen pellets, HoPoMo: 15 mg: "On average,
  ; one pollen foraging flight results in 15mg of collected pollen (Seeley, 1995)"

set ProbPollenCollection  0
  ; probability to collect pollen instead of nectar  calculated in ForagingRoundProc

set SEARCH_LENGTH_M 17 * 60 * FLIGHT_VELOCITY  ; 17*60*6.5 = 6630m
  ; [m] distance (= 17 min!), a unsuccesful forager flies on average
  ; Seeley 1983: search trip: 17min (+-11)

set SimpleDancing FALSE
  ; (false) if true: fixed nectar dancing TH and fixed number of dance followers

set TIME_UNLOADING  116
  ; (116) [s] time, a nectar forager needs to become unloaded  derived from Seeley 1994

set TIME_UNLOADING_POLLEN  210
```

```
  ; (210s = 3.5 min) [s]  Ribbands p.131: 3.5 minutes (Park 1922,1928b)

 set TotalFPdetectionProb  -1
  ; correct value is set in "Foraging_searchingProc" but only when searching takes places


; MORTALITY
 set DRONE_LIFESPAN  37
 ; Fukuda Ohtani 1977; life span drones:  summer: 14d, autumn: 32-42d
 set LIFESPAN  290
  ; [d] 290d (max. life span of worker; Sakagami, Fukuda 1968)

 set MAX_TOTAL_KM  800
  ; [ km ]  800, as mortality acts only at end of time step! 838km: max. flight
  ; performance in a foragers life (Neukirch 1982)

 set MORTALITY_DRONE_EGGS 0.064     ;  Fukuda Ohati 1977:
 set MORTALITY_DRONE_LARVAE 0.044   ;  100 eggs, 82 unsealed brood, 60 sealed brood and 56 adults
 set MORTALITY_DRONE_PUPAE  0.005
 set MORTALITY_DRONES  0.05       ; Fukuda Ohati 1977: "summer", av. lifespan: 14d
 set MORTALITY_EGGS  0.03          ;  HoPoMo p. 230: 0.03
 set MORTALITY_LARVAE  0.01       ;  HoPoMo p. 230: 0.01
 set MORTALITY_PUPAE  0.001       ;  HoPoMo p. 230: 0.001
 set MORTALITY_FOR_PER_SEC  0.00001
  ; derived from Visscher&Dukas 1997 (Mort 0.036 per hour foraging)

 set MORTALITY_INHIVE  0.004;
  ; 0.0038: derived from Martin 2001 (healthy winter
  ; based on 50% mortality) (HoPoMo: MORTALITYbase: 0.01) p. 230
```

```
; PHYSICS
  set ENERGY_HONEY_per_g  12.78
    ; [kJ/g] (= [J/mg])    Wikipedia: http://www.nal.usda.gov/fnic/foodcomp/search/

  set ENERGY_SUCROSE  0.00582       ; 0.00582 [kJ/micromol]   342.3 g/mol


; PROGRAM
  set STEPWIDTH  50       ;  for graphic
  set STEPWIDTHdrones  5       ;  for graphic
  set BugAlarm  FALSE       ;
  set N_GENERIC_PLOTS 8


; VARROA
  set MITE_FALL_DRONECELL  0.2
    ;  0.2 (20%) Martin 1998  proportion of those mites emerging from
    ; worker cells, which fall from the comb and are hence considered to die.

  set MITE_FALL_WORKERCELL  0.3
    ;  0.3 (30%) Martin 1998  proportion of those mites emerging from drone
    ; cells, which fall from the comb and are hence considered to die.

  set MITE_MORTALITY_BROODPERIOD  0.006
    ; Martin 1998: 0.006;  (0.006: Fries et al 1994, Tab. 6) daily mortality of phoretic
    ; mites during brood period

  set MITE_MORTALITY_WINTER  0.002
    ; Martin 1998: 0.002;   Fries et al 1994: 0.004 (Tab. 6)
  set NewReleasedMitesToday  0
    ;  all (healthy and infected) mites released from cells (mothers+offspring)
    ; on current day (calculated after MiteFall!)
```

```
; AUXILIARY VARIABLES
  set DecentHoneyEnergyStore  N_INITIAL_BEES * 1.5 * ENERGY_HONEY_per_g
    ; re-set in every foraging round (ForagingRoundProc )

  set HONEY_STORE_INIT  0.5 * MAX_HONEY_STORE_kg * 1000
    ;  [g]   (1g Honey = 124.80kJ)

  set HoneyEnergyStore  (HONEY_STORE_INIT * ENERGY_HONEY_per_g)       ;  [kJ]
  set IdealPollenStore_g  POLLEN_STORE_INIT
    ; [g] is calculated daily in PollenConsumptionProc

  set MAX_HONEY_ENERGY_STORE  MAX_HONEY_STORE_kg * ENERGY_HONEY_per_g * 1000 ; [kJ]
  set PollenStore_g  POLLEN_STORE_INIT       ;  [g]
  set NewForagerSquadronsHealthy (N_INITIAL_BEES / SQUADRON_SIZE)
    ; foragers in time step 1 are all healthy

  set TotalForagers  NewForagerSquadronsHealthy * SQUADRON_SIZE
    ;  has to be set here to calculate egg laying on the 1st time step

  set Aff  AFF_BASE
  set INVADING_DRONE_CELLS_AGE  DRONE_PUPATION_AGE - 2
    ; 2d before capping, Boot et al. 1992 (Exp. & Appl. Acarol. 16:295-301)

  set INVADING_WORKER_CELLS_AGE  PUPATION_AGE - 1
    ; 1d before capping, Boot et al. 1992 (Exp. & Appl. Acarol. 16:295-301)

  set PhoreticMites  N_INITIAL_MITES_HEALTHY + N_INITIAL_MITES_INFECTED
  set TotalMites  PhoreticMites
  set PATCHCOLOR 38 ; colour of the background
  ask patches [ set pcolor PATCHCOLOR ]
  if (N_INITIAL_MITES_HEALTHY + N_INITIAL_MITES_INFECTED) > 0
  [
```

```
  set PhoreticMitesHealthyRate N_INITIAL_MITES_HEALTHY
      / (N_INITIAL_MITES_HEALTHY + N_INITIAL_MITES_INFECTED)
 ]
 if RAND_SEED != 0 [ random-seed RAND_SEED ]
  ; if RAND_SEED set to 0, random numbers will differ in every run


; MITE REPRODUCTION MODELS
 if MiteReproductionModel = "Fuchs&Langenbach"
 [
  set MAX_INVADED_MITES_DRONECELL 16
    ; 16 (Fuchs&Langenbach 1989) defines length of workercell, dronecell list
    ; of MiteOrganisers

  set MAX_INVADED_MITES_WORKERCELL 8
    ; (Fuchs&Langenbach 1989)
    ; defines length of workercell, dronecell list of MiteOrganisers
 ]

 if MiteReproductionModel = "Martin"
 [
  set MAX_INVADED_MITES_DRONECELL 4
    ; defines length of workercell, dronecell list of MiteOrganisers
  set MAX_INVADED_MITES_WORKERCELL 4
    ; defines length of workercell, dronecell list of MiteOrganisers
 ]

 if MiteReproductionModel = "Test"
 [
  set MAX_INVADED_MITES_DRONECELL 5
  set MAX_INVADED_MITES_WORKERCELL 5
 ]
```

```
  if MiteReproductionModel = "Martin+0"
  [
   set MAX_INVADED_MITES_DRONECELL 5
   set MAX_INVADED_MITES_WORKERCELL 5
  ]

  if MiteReproductionModel = "No Mite Reproduction"
  [
   set MAX_INVADED_MITES_DRONECELL 5
   set MAX_INVADED_MITES_WORKERCELL 5
  ]


; VIRUS TYPES
  if Virus = "DWV"
  [
   set VIRUS_TRANSMISSION_RATE_MITE_TO_PUPA 0.89  ; 0.89
   set VIRUS_TRANSMISSION_RATE_PUPA_TO_MITES 1   ; 1: Martin 2001
   set VIRUS_KILLS_PUPA_PROB 0.2   ; DWV: 0.2 (Martin 2001)
   set MORTALITY_INHIVE_INFECTED_AS_PUPA 0.012; (0.0119)
     ; if pupa was infected but survived
     ; based on Martin 2001 Survivorship curve (infected, winter)
     ; calculated at: 50% mortality(=58d);

   set MORTALITY_INHIVE_INFECTED_AS_ADULT MORTALITY_INHIVE
     ;  Martin 2001: DWV infected adults become carriers with unaffected survivorship

   set MORTALITY_DRONES_INFECTED_AS_PUPAE MORTALITY_INHIVE_INFECTED_AS_PUPA * (MORTALITY_DRONES /
MORTALITY_INHIVE)
     ; NO data on drone mortality! Use same increase in mortality as for workers
  ]

  if Virus = "APV"
```

```
 [
  set VIRUS_TRANSMISSION_RATE_MITE_TO_PUPA 1
  set VIRUS_TRANSMISSION_RATE_PUPA_TO_MITES 0
    ; 0: Martin 2001 (0, as the pupae dies! - so this value doesn't matter at all!)

  set VIRUS_KILLS_PUPA_PROB 1   ; APV: 1 (Martin 2001)
  set MORTALITY_INHIVE_INFECTED_AS_PUPA 1
    ; doesn't matter, as APV infected pupae die before emergence!

  set MORTALITY_INHIVE_INFECTED_AS_ADULT 0.2
   ; (0.2: Sumpter & Martin 2004)

  set MORTALITY_DRONES_INFECTED_AS_PUPAE MORTALITY_INHIVE_INFECTED_AS_PUPA * (MORTALITY_DRONES /
MORTALITY_INHIVE)
    ; NO data on drone mortality! Use same increase in mortality as for workers
 ]

 if Virus = "benignDWV" ; like DWV but does not harm the infected bees
 [
  set VIRUS_TRANSMISSION_RATE_MITE_TO_PUPA 0.89     ; 1
  set VIRUS_TRANSMISSION_RATE_PUPA_TO_MITES 1
    ; 0: Martin 2001 (0, as the pupae dies!)
  set VIRUS_KILLS_PUPA_PROB 0  ; (benign!)
  set MORTALITY_INHIVE_INFECTED_AS_PUPA MORTALITY_INHIVE ; (benign!)
  set MORTALITY_INHIVE_INFECTED_AS_ADULT MORTALITY_INHIVE
  set MORTALITY_DRONES_INFECTED_AS_PUPAE MORTALITY_INHIVE_INFECTED_AS_PUPA
    ; NO data on drone mortality! Use worker mortality!
 ]

 if Virus = "modifiedAPV"
 [
  set VIRUS_TRANSMISSION_RATE_MITE_TO_PUPA 1     ; 1
```

```
  set VIRUS_TRANSMISSION_RATE_PUPA_TO_MITES 1   ;
  set VIRUS_KILLS_PUPA_PROB 1  ; APV: 1 (Martin 2001)
  set MORTALITY_INHIVE_INFECTED_AS_PUPA 1
    ; doesn't matter, as APV infected pupae die before emergence!

  set MORTALITY_INHIVE_INFECTED_AS_ADULT 0.2
    ; (0.2: Sumpter & Martin 2004)

  set MORTALITY_DRONES_INFECTED_AS_PUPAE MORTALITY_INHIVE_INFECTED_AS_PUPA
  ; NO data on drone mortality! Use worker mortality!
 ]

 if Virus = "TestVirus"
 [
  set VIRUS_TRANSMISSION_RATE_MITE_TO_PUPA 1  ; 0.89
  set VIRUS_TRANSMISSION_RATE_PUPA_TO_MITES 1   ; 1: Martin 2001
  set VIRUS_KILLS_PUPA_PROB 0   ; DWV: 0.2 (Martin 2001)
  set MORTALITY_INHIVE_INFECTED_AS_PUPA 0.012; (0.0119)
    ; if pupae was infected but survived; based on Martin 2001 Survivorship
    ; curve (infected, winter) calculated at 50% mortality = 58d age

  set MORTALITY_INHIVE_INFECTED_AS_ADULT MORTALITY_INHIVE
    ;  Martin 2001: DWV infected adults become carriers with unaffected survivorship

  set MORTALITY_DRONES_INFECTED_AS_PUPAE MORTALITY_INHIVE_INFECTED_AS_PUPA
    ; NO data on drone mortality! Use worker mortality!
 ]

end;

; ****************************************************************************************************************************
```

```
to CreateImagesProc
  ; "signs" are symbols in the NetLogo "World" which are used to visualize structure
  ; and dynamics of the colony/varroa model

  create-hives 1
  [
    ifelse ReadInfile = true ;
      ; true: hive placed on the left side, else: in the centre
      [ setxy -1 4.5 ]
      [ setxy 16 4.5 ]
    set size 7 set shape "beehiveDeepHive" set color brown
  ]

  create-Signs 1
  [
    setxy 16 -15
    set shape "skull"
    set size 15
    set color black
    hide-turtle
  ] ;

  create-Signs 1
  [
    setxy 40 3
    set shape "sun"
    set size 7
    set color yellow
    hide-turtle
  ] ;

  create-Signs 1
```

```
[
 setxy 37 2
 set shape "cloud"
 set size 7
 set color grey
 hide-turtle
]

create-Signs 1
[
 setxy 38 -10
 set shape "beelarva_x2"
 set size 8
 set color white
 facexy xcor + 1 ycor + 1 ; (turned by 45deg)
 hide-turtle
]

create-Signs 1
[
 setxy 31 3
 set shape "arrow"
 set size 4
 set color green
 facexy xcor + 1 ycor
 set label (HoneyEnergyStore - HoneyEnergyStoreYesterday)
       / ( ENERGY_HONEY_per_g * 1000 )
]

create-Signs 1
[
 setxy 26 3
```

```
  set shape "arrowpollen"
  set size 4
  set color green
  facexy xcor - 1 ycor
  set label (PollenStore_g - PollenStore_g_Yesterday)
]

create-Signs 1
  ; sign for suppressed foraging i.e. if foraging prob. is set
  ; to 0 although weather is suitable for foraging
[
  setxy 36 -4
  set shape "exclamation"
  set size 3
  set color orange
  hide-turtle
]

create-Signs 1
[
  setxy 38 -18
  set shape "pete"
  set size 6
  set color white
  set label-color black
  hide-turtle
]

create-Signs 1
[
  setxy 38 -25
  set shape "honeyjar"
```

```
  set size 6
  set color white
  hide-turtle
]

create-Signs 1
[
  setxy 38 -25
  set shape "ambrosia"
  set size 6
  set color white
  hide-turtle
]
create-Signs 1
[
  setxy 42.5 -25
  set shape "pollengrain"
  set size 7
  set color yellow
  hide-turtle
]
create-Signs 1
[
  setxy 38 -31
  set shape "varroamite03"
  set size 6
  set color 33
  set heading 0
  hide-turtle
]
create-Signs 1
[
```

```
    setxy 38 -31.2
    set shape "x"
    set size 6
    set color red
    hide-turtle
  ]
  create-Signs 1
  [
    setxy 38 -33
    set shape "colonies_merged"
    set size 6
    set color brown
    set heading 45
    hide-turtle
  ]
  create-Signs 1
  [
    setxy 38 -40
    set shape "queen"
    set size 8
    set color 33
    set heading 0
    hide-turtle
    ]
end

; ****************************************************************************************************************************

to Go

  tick
  DailyUpdateProc
```

```
SeasonProc_HoPoMo

; Egg laying & development:
WorkerEggsDevProc
DroneEggsDevProc
NewEggsProc
if Swarming != "No swarming" [ SwarmingProc ]
WorkerEggLayingProc
DroneEggLayingProc
WorkerLarvaeDevProc
DroneLarvaeDevProc
NewWorkerLarvaeProc
NewDroneLarvaeProc
WorkerPupaeDevProc
DronePupaeDevProc
NewWorkerPupaeProc
NewDronePupaeProc
WorkerIHbeesDevProc
DronesDevProc
BroodCareProc
NewIHbeesProc
NewDronesProc

; Varroa mite module:
if (TotalMites > 0) [ MiteProc ]
BeekeepingProc
DrawIHcohortsProc

; Foraging module:
GenericPlotClearProc
if ( TotalForagers
    + NewForagerSquadronsHealthy * SQUADRON_SIZE
    + NewForagerSquadronsInfectedAsPupae * SQUADRON_SIZE
```

```
      + NewForagerSquadronsInfectedAsAdults * SQUADRON_SIZE ) > 0
  [
    Start_IBM_ForagingProc
  ]

 ask turtles
 [
  set label-color black
  ifelse ploidy = 2
  [
    set label number
  ]
  [
    if ploidy = 1
    [
      set label number
    ]
  ]
 ]
 CountingProc
 PollenConsumptionProc
 HoneyConsumptionProc
 DoPlotsProc
end

; ****************************************************************************************************


to GoTreatmentProc
 ; similar to "Go", but used if colonies don't start on 1st January
 ; (e.g. to mimic empirical colony treatments), called only once by "Setup"
 ; but contains a "repeat"-loop
```

```
;;  repeat (INSERT START DAY)
;;  [
;;    Go
;;    set HoneyEnergyStore (MAX_HONEY_ENERGY_STORE / 5)
;;    set PollenStore_g 0.5 * IdealPollenStore_g
;;    ; guarantees survival of colonies before experiment
;;  ]
;;
;;  ask (turtle-set droneEggCohorts droneLarvaeCohorts) [ set number (INSERT NUMBER) ]
;;
;;  ask (turtle-set dronePupaeCohorts droneCohorts)
;;  [
;;    set number (INSERT NUMBER)
;;    set number_healthy (INSERT NUMBER)
;;    set number_infectedAsPupa (INSERT NUMBER)
;;  ]
;;  ask eggCohorts [ set number (INSERT NUMBER) ]
;;  ask larvaeCohorts [ set number (INSERT NUMBER) ]
;;  ask pupaeCohorts
;;  [
;;    set number (INSERT NUMBER)
;;    set number_Healthy (INSERT NUMBER)
;;    set number_infectedAsPupa (INSERT NUMBER)
;;  ]
;;
;;  ask IHbeeCohorts
;;  [
;;    set number_healthy (INSERT NUMBER)
;;    set number_infectedAsPupa (INSERT NUMBER)
;;    set number_infectedAsAdult (INSERT NUMBER)
```

```
;;  ]
;;
;;  set HoneyEnergyStore ENERGY_HONEY_per_g * (INSERT NUMBER OF CELLS WITH HONEY)
;;    ; 1 comb ca. 2*3268 cells (PJK), 1 cell full of honey = 500mg
;;    ; (Schmickl, Crailsheim, HoPoMo)
;;
;;  if Experiment = "INSERT NAME EXPERIMENT A"
;;  [
;;    (INSERT INITIAL CONDITIONS FOR EXERIMENT A)
;;  ]
;;
;;  if Experiment = "INSERT NAME EXPERIMENT B"
;;  [
;;    (INSERT INITIAL CONDITIONS FOR EXERIMENT B)
;;  ]
;;
;;
;;  ask miteOrganisers
;;  [
;;    set droneCellListCondensed n-values (MAX_INVADED_MITES_DRONECELL + 1) [ (INSERT NUMBER) ]
;;  ]    ; +1 as also the number of mite free cells is stored in this list
;;
;;  StartProc
end

; ****************************************************************************************************************

to-report FlowerPatchesMaxFoodAvailableTodayREP [ patchID foodType ]
  ; foodType: "Nectar" or "Pollen"
  ; determines the max amount of nectar and pollen available at the patch today
  ; this reporter is ONLY called if ReadInfile = FALSE!!
```

```
; called by: CreateFlowerPatchesProc (i.e. 1x per run), DailyUpdateProc (i.e. 1x per day),
; and        FlowerPatchesUpdateProc (i.e. 1x per foraging round)

ifelse SeasonalFoodFlow = true
[
  ; SEASONAL variation of nectar ond pollen availability at RED and
  ; GREEN patch (if SeasonalFoodFlow = ON):
  let patchDayR day + SHIFT_R
  if day + SHIFT_R > 365 [ set patchDayR patchDayR - 365 ]
    ; to shift the seasonal food offer to earlier (+) or later (-) in the year

  let patchDayG day + SHIFT_G
  if day + SHIFT_G > 365 [ set patchDayG patchDayG - 365 ]

  if foodType != "Nectar" and foodType != "Pollen"
  [
    set BugAlarm true
    show "BUG ALARM in FlowerPatchesFoodAvailableTodayREP - Wrong 'foodType' of flower patch!"
  ]
  if patchID != 0 and patchID != 1
  [
    set BugAlarm true
    show "BUG ALARM in FlowerPatchesFoodAvailableTodayREP - Wrong 'who' of flower patch!"
  ]

  if ReadInfile = true
  [
    set BugAlarm true
    show "BUG ALARM in FlowerPatchesFoodAvailableTodayREP - called although ReadInfile = true!"
  ]

  if patchID = 0 ; "RED" patch
```

```
  [
    if foodType = "Nectar"
    [
      report (1 - Season_HoPoMoREP patchDayR []) * QUANTITY_R_l * 1000 * 1000
    ]
    if foodType = "Pollen"
    [
      report (1 - Season_HoPoMoREP patchDayR []) * POLLEN_R_kg * 1000
    ]
  ]

  if patchID = 1 ; "GREEN" patch
  [
    if foodType = "Nectar"
    [
      report (1 - Season_HoPoMoREP patchDayG []) * QUANTITY_G_l * 1000 * 1000
    ]
    if foodType = "Pollen"
    [
      report (1 - Season_HoPoMoREP patchDayG []) * POLLEN_G_kg * 1000
    ]
  ]
]
[
  ; ELSE (i.e. if SeasonalFoodFlow = FALSE):
  if foodType = "Nectar"
  [
    if patchID = 0 [ report QUANTITY_R_l * 1000 * 1000 ] ; "red" patch
    if patchID = 1 [ report QUANTITY_G_l * 1000 * 1000 ] ; "green" patch
  ]

  if foodType = "Pollen"
```

```
    [
      if patchID = 0 [ report POLLEN_R_kg * 1000 ] ; "red" patch
      if patchID = 1 [ report POLLEN_G_kg * 1000 ] ; "green" patch
    ]
  ]
end



; *****************************************************************************************************************************************


to DailyUpdateProc
  set Day round (ticks mod 365.00001)
  set DeathsAdultWorkers_t 0
  set SumLifeSpanAdultWorkers_t 0
  set DailyMiteFall 0
  set Pupae_W&D_KilledByVirusToday 0
  set NewReleasedMitesToday 0
    ; all (healthy and infected) mites released from cells (mothers+offspring)
    ; on current day (calculated after MiteFall!)

  ask foragerSquadrons [ set km_today 0 ]
  if N_INITIAL_MITES_INFECTED = 0
  [
    if ( count foragerSquadrons with [ infectionState = "infectedAsPupa"]
       + count foragerSquadrons with [ infectionState = "infectedAsAdult"] ) > 0
         or
       ( count IHbeeCohorts with [ number_infectedAsPupa > 0]
       + count IHbeeCohorts with [ number_infectedAsAdult > 0] ) > 0
    [
      set BugAlarm true
```

```
    show "BUG ALARM! Infected bees from out of the blue!"
  ]
]

ask flowerpatches
[
  ifelse ( quantityMyl < CROPVOLUME * SQUADRON_SIZE
           and
           amountPollen_g < POLLENLOAD * SQUADRON_SIZE )
    [ set shape "fadedFlower" ] ; IF
    [ set shape "Flower" ] ; ELSE = not empty
]

set DailyForagingPeriod Foraging_PeriodREP
set HoneyEnergyStoreYesterday HoneyEnergyStore
set PollenStore_g_Yesterday PollenStore_g
set LostBroodToday 0
set Queenage Queenage + 1

ask patch 0 -27 [ set plabel  5] ask patch 0 -32 [ set plabel 10]
ask patch 0 -37 [ set plabel 15] ask patch 0 -42 [ set plabel 20]
ask patch 0 -47 [ set plabel 25] ask patch 0 -52 [ set plabel 30]
ask patch 0 -57 [ set plabel 35] ask patch 1 -58 [ set plabel "age  "]

set SearchingFlightsToday 0
set RecruitedFlightsToday 0
set NectarFlightsToday 0
set PollenFlightsToday 0
set EmptyFlightsToday 0
set DeathsForagingToday 0

if ReadInfile = false
```

```
 [
  ask flowerPatches
  [ ; flower patches are set to the max. amount of nectar and pollen possible today:
    set quantityMyl FlowerPatchesMaxFoodAvailableTodayREP who "Nectar"
    set amountPollen_g FlowerPatchesMaxFoodAvailableTodayREP who "Pollen"
  ]
 ]

ask flowerPatches
[
  set nectarVisitsToday 0 set pollenVisitsToday 0
  if detectionProbability < -1
  [
    set BugAlarm true
    user-message "Wrong detection probability! Set 'ModelledInsteadCalcDetectProb' 'false' and re-start run!"
  ]
]

if ReadInfile = true
[
  set TodaysSinglePatchList []
    ; short list, contains data of current patch and only for today
  set TodaysAllPatchesList []
    ; shorter list, contains data of all patches, but only for today
  let counter (Day - 1)
  repeat N_FLOWERPATCHES
  [
    ; todays data for ALL N_FLOWERPATCHES flower patches are saved in a new,
    ; shorter list (= todaysAllPatchesList)

    set TodaysSinglePatchList (item counter AllDaysAllPatchesList)
      ; this new, shorter list (= todaysAllPatchesList) is comprised of very
```

```
  ; short lists (=todaysSinglePatchList) that contain only the data of the
  ; current patch and only for today

set TodaysAllPatchesList fput TodaysSinglePatchList TodaysAllPatchesList
  ; fput: faster as lput (NetLogo version 4)! however: list is in reversed order!

set counter counter + 365
let id item 1 TodaysSinglePatchList ; patch number

ask flowerpatch id
[
  set amountPollen_g item 8 TodaysSinglePatchList ; [g]
  if amountPollen_g < 0 [ set amountPollen_g 0 ]
  set quantityMyl (item 10 TodaysSinglePatchList) * 1000 * 1000
    ; [microlitres]  new nectar value from infile (emptied flowers
    ; replenish nectar completely (or are replace by new flowers))

  if quantityMyl < 0 [ set quantityMyl 0 ]
  if id != who [  user-message "Error in id / who!" set BugAlarm true  ]

  if shape != "fadedflower"
  [
    ifelse amountPollen_g > 250
    [ set shape "flowerorange" ]
    [ set shape "flower" ]
  ]
    ; if a "reasonable" amount of pollen available, patch is shown
    ; as 'pollen patch'

  ifelse quantityMyl < CROPVOLUME * SQUADRON_SIZE [ set color grey ]
  [
    set color scale-color red eef 0 50
```

```
          ; colour: reddish, dependent on eef, if eff >= 50: white
        ]
      ]
    ] ; ask flowerpatch ID

    set todaysAllPatchesList reverse todaysAllPatchesList
      ; to correct the reversed order, caused by the fput command
  ] ; repeat

  ask patches [ set pcolor PATCHCOLOR ]

  ask hives
  [
    set shape "beehiveDeepHive"
      ; # of supers on drawn colony depends on honey store

    if HoneyEnergyStore / ENERGY_HONEY_per_g > 15000 [ set shape "beehive1super" ]
    if HoneyEnergyStore / ENERGY_HONEY_per_g > 30000 [ set shape "beehive2super" ]
    if HoneyEnergyStore / ENERGY_HONEY_per_g > 45000 [ set shape "beehive3super" ]
    if HoneyEnergyStore / ENERGY_HONEY_per_g > 60000 [ set shape "beehive4super" ]
    if HoneyEnergyStore / ENERGY_HONEY_per_g > 75000 [ set shape "beehive5super" ]
    if HoneyEnergyStore / ENERGY_HONEY_per_g > 90000 [ set shape "beehive6super" ]
    if HoneyEnergyStore / ENERGY_HONEY_per_g > 105000 [ set shape "beehive7super" ]
    if HoneyEnergyStore < 0
    [
      if ColonyDied = false
      [
        output-print word "Starvation! Colony died on Day " ticks
      ]
      set ColonyDied true
    ]
  ] ; ask hives
```

```
if (ticks > 1) and (TotalWorkerAndDroneBrood + TotalIHbees + TotalForagers = 0)
[
  if ColonyDied = false
  [
    output-print word "No bees left! Colony died on Day " ticks
  ]
  set ColonyDied true
]

if (Day = 365)
[
  output-type word "31.12.: COLONY SIZE: " (TotalIHbees + TotalForagers)
  output-type "   HONEY STORE [kg]: "
  output-print precision (HoneyEnergyStore / (1000 * ENERGY_HONEY_per_g)) 1
]

if (Day = 365) and (TotalIHbees + TotalForagers < CRITICAL_COLONY_SIZE_WINTER)
[
  if ColonyDied = false
  [
    output-print word "Winter mortality! Colony died on Day " ticks
  ]
  set ColonyDied true
]

if ColonyDied = true
[
  ask hives [ set color grey ]
    ; grey colony: died! (even if it "recovers" later, it remains grey)

  if stopDead = true
```

```
[
  ask Signs with [shape = "skull"]
  [
    show-turtle
  ]
]
ask patches [ set pcolor black ]
if stopDead = true
[
  ask eggCohorts [ set number 0]
  ask larvaeCohorts [ set number 0]
  ask pupaeCohorts
  [
    set number 0
    set number_Healthy 0
    set number_infectedAsPupa 0
  ]
  ask IHbeeCohorts
  [
    set number 0
    set number_Healthy 0
    set number_infectedAsPupa 0
    set number_infectedAsAdult 0
  ]
  ask foragerSquadrons [ die ]
  ask droneEggCohorts [ set number 0]
  ask droneLarvaeCohorts [ set number 0]
  ask dronePupaeCohorts
  [
    set number 0
    set number_Healthy 0
    set number_infectedAsPupa 0
```

```
    ]
    ask droneCohorts [ set number 0  ]
  ]
 ]

end

; *****************************************************************************************************************************************



to-report Season_HoPoMoREP [ today parameterList ]
  ; see Schmickl&Crailsheim2007: p.221 and p.230
  ; Values HoPoMo: x1 385; x2 30; x3 36; x4 155; x5 30

  let x1 385  ;385
  let x2 25 ; (earlier increase in egg laying rate than in HoPoMo)
  let x3 36    ; 36
  let x4 155   ;155  ; Day of max. egg laying
  let x5 30    ;30
  if empty? parameterList = false
  [
    set x1 item 0 parameterList
    set x2 item 1 parameterList
    set x3 item 2 parameterList
    set x4 item 3 parameterList
    set x5 item 4 parameterList
  ]
  let seas1 (1 - (1 / (1 + x1 * e ^ (-2 * today / x2))))
  let seas2 (1 / (1 + x3 * e ^ (-2 * (today - x4) / x5)))
  ifelse seas1 > seas2
    [ report seas1 ]
```

```
  [ report seas2 ]
end

; ****************************************************************************************************************************************


to SeasonProc_HoPoMo
  ; see Schmickl&Crailsheim2007: p.221 and p.230

  set HoPoMo_seasont Season_HoPoMoREP day []
    ; calls to-report SeasonProc_HoPoMoREP to calculate the HoPoMo seasonal
    ; factor on basis of "day" and of a parameter list ("[]"), which is empty in
    ; this case but could contain 5 values: x1..x5
end

; ****************************************************************************************************************************************


to NewEggsProc
  ; CALLED BY WorkerEggLayingProc   see: HoPoMo p.222 & p.230, ignoring ELRstoch
  let ELRt_HoPoMo (MAX_EGG_LAYING * (1 - HoPoMo_seasont))
  if EMERGING_AGE <= 0 [ set BugAlarm true show "EMERGING_AGE <= 0" ]
  let ELRt_IH (TotalIHbees
      + TotalForagers * FORAGER_NURSING_CONTRIBUTION)
      * MAX_BROOD_NURSE_RATIO / EMERGING_AGE
        ; EMERGING_AGE = 21: total developmental time of worker brood

  let ELRt ELRt_HoPoMo
    ; egg laying rate follows a seasonal pattern as described in
    ; HoPoMo (Schmickl & Crailsheim 2007)

  if EggLaying_IH = true and ELRt_IH < ELRt_HoPoMo
```

```
  ; if EggLaying_IH SWITCH is on and not enough nurse bees are available,
  ; the egg laying rate is reduced to ELRt_IH
 [
  set ELRt ELRt_IH
 ]

 if ELRt > MAX_EGG_LAYING
 [
  set ELRt MAX_EGG_LAYING
 ]


 ; limited brood nest
 if TotalWorkerAndDroneBrood + ELRt > MAX_BROODCELLS
 [
  set ELRt MAX_BROODCELLS - TotalWorkerAndDroneBrood
 ]

 set NewWorkerEggs round ELRt  ; ROUND! in contrast to HoPoMo


 ; calculation of drone eggs
 set NewDroneEggs floor(NewWorkerEggs * DRONE_EGGS_PROPORTION)
 if Day >= SEASON_STOP
   - ( DRONE_HATCHING_AGE
   -   DRONE_PUPATION_AGE
   -   DRONE_EMERGING_AGE )
 [
  set NewDroneEggs 0
 ] ; no more drone brood at end of season (however: Season set to day 1 - 365)
; drone egg production in DroneEggLayingProc restricted to period between Drone_Egglaying_Start & ..Stop!
```

```
  ; ageing of queen
  ;  based on deGrandi-Hofmann, BEEPOP:
  if QueenAgeing = true ; GUI: "switch"
  [
    let potentialEggs (MAX_EGG_LAYING
        + (-0.0027 * Queenage ^ 2)
        + (0.395 * Queenage))
          ; Beepops potential egglaying Pt
    set NewWorkerEggs round (NewWorkerEggs * (potentialEggs / MAX_EGG_LAYING) )
  ]

  ; no egg-laying of young queen (also if QUEEN_AGEING = false!):
  if Queenage <= 10
  [
    set NewWorkerEggs 0
      ; Winston p. 203: 5-6d until sexually mature, 2-4d for orientation and mating flight, mating
      ; can be postponed for 4 weeks if weather is bad

    set NewDroneEggs 0
  ]
  if NewWorkerEggs < 0 [ set NewWorkerEggs 0 ]
  if NewDroneEggs < 0 [ set NewDroneEggs 0 ]
end

; ***************************************************************************************************************


to SwarmingProc

  ; # total brood triggers swarming
  ; PRE_SWARMING_PERIOD: 3d of preparation before swarming
  ; SwarmingDate: set to 0 in Param.Proc and in SwarmingProc (after swarming and on day 365)
```

```
let fractionSwarm 0.6  ; 0.6 ; Winston p. 187
let broodSwarmingTH 17000 ; Fefferman & Starks 2006 (model)
let lastSwarmingDate 199; Winston 1980: prime: 14.05.(134) after swarm: 18.07.(199)
 ; McLellan, Rowland 1986: 162 (modelled),
if TotalWorkerAndDroneBrood > broodSwarmingTH and SwarmingDate = 0 and day <= (lastSwarmingDate - PRE_SWARMING_PERIOD)
[
  set SwarmingDate (day + PRE_SWARMING_PERIOD)
]

if day = SwarmingDate
   and Swarming = "Swarm control"
[
  output-type "Swarming (prevented) on day: " output-print day
]

if day >= SwarmingDate - PRE_SWARMING_PERIOD
   and day <= SwarmingDate
[
  if Swarming = "Swarming (parental colony)"
  [

; Swarm PREPARATION of PARENTAL colony:
    set NewDroneEggs 0
    set NewWorkerEggs 0
    if  day = SwarmingDate
    [ ; SWARMING of PARENTAL colony:
      set Queenage -7
        ; a new queen is left in the hive, still in a capped cell, ca. 7d
        ; before she emerges (Winston p. 187)

      ; Winston p. 185: 36mg honey is taken by a swarming bee:
      set HoneyEnergyStore HoneyEnergyStore
```

```
    - (( TotalForagers + TotalIHbees) * 0.036 * ENERGY_HONEY_per_g)
    * fractionSwarm

  ; (1-fractionSwarm) of all healthy & infected in-hive bees stay in the hive:
  ask IHbeeCohorts
  [
   set number_Healthy round (number_Healthy * (1 - fractionSwarm))
   set number_infectedAsPupa round (number_infectedAsPupa * (1 - fractionSwarm))
   set number_infectedAsAdult round (number_infectedAsAdult * (1 - fractionSwarm))
   set number number_Healthy + number_infectedAsPupa + number_infectedAsAdult
  ]

  ; (1-fractionSwarm) of all healthy & infected drones stay in the hive:
  ask droneCohorts
  [
   set number_Healthy round (number_Healthy * (1 - fractionSwarm))
   set number_infectedAsPupa round (number_infectedAsPupa * (1 - fractionSwarm))
   set number number_Healthy + number_infectedAsPupa
  ]

  ; fractionSwarm foragers leave the colony and are considered to be dead in the model:
  ask foragerSquadrons
  [
   if random-float 1 < fractionSwarm [ die ]
  ] ; LEAVING foragers are treated as being dead

  ; the phoretic mite population in the hive is reduced:
  set PhoreticMites round (PhoreticMites * (1 - fractionSwarm))
  output-type "Swarming on day: " output-print day
  set SwarmingDate 0  ; allows production of after swarms
 ]
]
```

```
    if Swarming = "Swarming (prime swarm)"
    [

; Swarm PREPARATION of PRIME SWARM:
      set NewDroneEggs 0
      set NewWorkerEggs 0
      if  day = SwarmingDate
      [ ; Swarming of PRIME SWARM:
        ask (turtle-set eggCohorts larvaeCohorts droneEggCohorts droneLarvaeCohorts)
        [ ; all brod is left behind and hence removed from the smulation:
          set number 0
        ]
        ask (turtle-set pupaeCohorts dronePupaeCohorts)
        [
          set number 0
          set number_infectedAsPupa 0
          set number_healthy 0
        ]
        set NewWorkerLarvae 0
        set NewDroneLarvae 0
        set NewWorkerPupae 0
        set NewDronePupae 0
        ask IHbeeCohorts
        [ ; fractionSwarm of all healthy & infected in-hive bees join the swarm
          set number_Healthy round (number_Healthy * fractionSwarm)
          set number_infectedAsPupa round (number_infectedAsPupa * fractionSwarm)
          set number_infectedAsAdult round (number_infectedAsAdult * fractionSwarm)
          set number number_Healthy + number_infectedAsPupa + number_infectedAsAdult
        ]

        ask droneCohorts
```

```
      [ ; fractionSwarm of all healthy & infected drones join the swarm
        set number_Healthy round (number_Healthy * fractionSwarm)
        set number_infectedAsPupa round (number_infectedAsPupa * fractionSwarm)
        set number number_Healthy + number_infectedAsPupa
      ]

      ask foragerSquadrons
      [ ; (1 - fractionSwarm) foragers do not join the swarm and hence die (in the model):
        if random-float 1 < (1 - fractionSwarm) [ die ]
      ]

      ask miteOrganisers [ die ]
        ; mites in brood cells are left behind in the old colony

      ; the phoretic mite population in the swarm is reduced:
      set PhoreticMites round (PhoreticMites * fractionSwarm)
      set PollenStore_g 0
      set HoneyEnergyStore
           ((TotalForagers + TotalIHbees)
             * 36 * ENERGY_HONEY_per_g) / 1000
        ; Winston p. 185: 36mg honey per bee during swarming
      output-type "Swarming on day: "
      output-print day
      set SwarmingDate 0  ; allows production of after swarms
    ] ; if  day = SwarmingDate ..
  ] ; if Swarming = "Swarming (prime swarm)"   ,,
] ; if SwarmingDate > 0 and ..

if Swarming = "Swarm (daughter colony)"
   and day > SwarmingDate
   and day <= SwarmingDate + POST_SWARMING_PERIOD   ; DAUGHTER COLONY AFTER SWARMING (0d period)
[ ; no eggs can be laid, no food stored, as long as they have no new home..
```

```
    set NewDroneEggs 0
    set NewWorkerEggs 0
    set PollenStore_g 0
    set Aff MAX_AFF
    if HoneyEnergyStore >
      (((TotalForagers + TotalIHbees) * CROPVOLUME) / 1000)
         * 1.36 * ENERGY_HONEY_per_g
    [
      set HoneyEnergyStore (((TotalForagers + TotalIHbees) *
        CROPVOLUME) / 1000) * 1.36 * ENERGY_HONEY_per_g
    ]
  ]
  ; resetting SwarmingDate to zero at the end of a year:
  if day = 365 [ set SwarmingDate 0 ]
end

; ********************************************************************************************************

to WorkerEggLayingProc

; creation of worker eggs
  create-eggCohorts 1 ;
  [
    set shape "circle"
    set number NewWorkerEggs
    set age 0
    setxy 3 0
    set color blue
    set ploidy 2
  ]
end
```

```
; ***************************************************************************************************************

to DroneEggLayingProc
; creation of drone eggs
  create-DroneEggCohorts 1 ;
  [
    set shape "circle"
    set number NewDroneEggs
    if Day < DRONE_EGGLAYING_START or Day > DRONE_EGGLAYING_STOP [ set number 0 ]
    set age 0
    setxy -5 0
    set color blue
    set ploidy 1
  ]
end

; ***************************************************************************************************************

to WorkerEggsDevProc
; ageing, deletion of oldest cohort
  ask eggCohorts
  [
    set age age + 1
    fd 1   ; turtle moves one step (display)
    set number (number - random-poisson (number * MORTALITY_EGGS))
    if number < 0 [ set number 0 ]
      ; random mortality, based on Poisson distribution

    if age = HATCHING_AGE [ set NewWorkerLarvae number ]
    if age >= HATCHING_AGE [ die ]
```

```
  ]
end

; ******************************************************************************************************************

to DroneEggsDevProc
; ageing, deletion of oldest cohort
  ask droneEggCohorts
  [
    set age age + 1
    set number (number - random-poisson (number * MORTALITY_DRONE_EGGS))
    if number < 0 [ set number 0 ]  ; random mortality, based on Poisson distribution
    if age = DRONE_HATCHING_AGE [ set NewDroneLarvae number ]
    if age >= DRONE_HATCHING_AGE [ die ]
    fd 1    ; turtle moves one step (display)
  ]
end

; ******************************************************************************************************************

to NewWorkerLarvaeProc
; creation of worker larvae
   create-larvaeCohorts 1
   [
     set number NewWorkerLarvae   ; the cohort size
     set age HATCHING_AGE
     set shape "circle"   ; shape
     set color yellow
     setxy 3 (- age)
     set ploidy 2     ; worker larvae are diploid
```

```
  ]
end

; ****************************************************************************************************************

to NewDroneLarvaeProc
; creation of drone larvae
  create-droneLarvaeCohorts 1
  [
    set shape "circle"
    set number NewDroneLarvae   ; the cohort size
    set age DRONE_HATCHING_AGE
    set color yellow
    setxy -5 (- age)
    set ploidy 1    ; drone larvae are haploid
  ]
end

; ****************************************************************************************************************

to WorkerLarvaeDevProc
; ageing of cohort
 ask larvaeCohorts
 [
   set age age + 1   ; cohorts age by one day
   fd 1    ; turtle moves one step (display)
   set numberDied 0
   set numberDied random-poisson (number * MORTALITY_LARVAE)
   if numberDied > number [ set numberDied number ]
     ; random mortality, based on Poisson distribution
```

```
    set number number - numberDied
    if (numberDied > 0)
      and ( age > INVADING_WORKER_CELLS_AGE )
      and (TotalMites > 0)
    [
     MitesReleaseProc invadedByMiteOrganiserID ploidy numberDied "dyingBrood"
    ]

    if age = PUPATION_AGE
    [
     set NewWorkerPupae number
     set SaveWhoWorkerLarvaeToPupae who   ; "Who" is stored as a global variable
     set SaveInvadedMOWorkerLarvaeToPupae invadedByMiteOrganiserID
    ]
    if age >= PUPATION_AGE [ die ]
  ]
end

; ******************************************************************************************************

to DroneLarvaeDevProc
 ; ageing of cohort
  ask droneLarvaeCohorts
  [
    set age age + 1
    set numberDied 0
    set numberDied random-poisson (number * MORTALITY_DRONE_LARVAE)
    if numberDied > number [ set numberDied number ]
     ; random mortality, based on Poisson distribution
    set number number - numberDied
```

```
    if (numberDied > 0)
      and ( age > INVADING_DRONE_CELLS_AGE )
      and (TotalMites > 0)
    [
      MitesReleaseProc invadedByMiteOrganiserID ploidy numberDied "dyingBrood"
    ] ; variables correspond to [ miteOrganiserID ploidyMO diedBrood ]

    fd 1
    if age = DRONE_PUPATION_AGE
    [
      set NewDronePupae number
      set SaveWhoDroneLarvaeToPupae who   ; "Who" is stored as a global variable
      set saveInvadedMODRONELarvaeToPupae invadedByMiteOrganiserID
    ]
    if age >= DRONE_PUPATION_AGE [ die ]
  ]
end

; ****************************************************************************************************


to NewWorkerPupaeProc

  create-pupaeCohorts 1
  [
    set shape "circle"   ; shape of the turtle as shown in the GUI
    set number NewWorkerPupae  ; cohort size
    set number_healthy number ; all newly created pupae are healthy
    set age PUPATION_AGE ; age of the cohort
    setxy 3 (- age)   ; xy position of the turtle in the Netlogo world
    set color brown    ; color of the turtle
    set ploidy 2    ; worker pupae are diploid
```

```
    set invadedByMiteOrganiserID SaveInvadedMOWorkerLarvaeToPupae
      ; saves "invadedByMiteOrganiserID" of the old larvaeCohort that has now developed
      ; into a pupaeCohort
    let saveWho who
      ; saves "who" for the following command (transition of larvae to pupae results in the
      ; death of larvae turtles, hence: ensuing pupae turtles have a different "who")
    ask miteOrganisers with [ invadedWorkerCohortID = SaveWhoWorkerLarvaeToPupae ]
    [
      set invadedWorkerCohortID saveWho
    ] ; miteOrganiser updates its value for the invadedWorkerCohortID
  ]
end

; ****************************************************************************************************************

to NewDronePupaeProc

   create-dronePupaeCohorts 1
   [
     set shape "circle"
     set number NewDronePupae
     set number_healthy number ; all newly created pupae are healthy
     set age DRONE_PUPATION_AGE
     setxy -5 (- age)
     set color brown
     set ploidy 1
     set invadedByMiteOrganiserID SaveInvadedMODroneLarvaeToPupae
       ; saves "invadedByMiteOrganiserID" of the old larvaeCohort that has
       ; now developed into a pupaeCohort

     let saveWho who
       ; saves "who" for the next line (transition of larvae to pupae results
       ; in the death of larvae turtles, hence: ensuing pupae turtles
```

```
      ; have a different "who")

     ask miteOrganisers with [ invadedDroneCohortID = SaveWhoDroneLarvaeToPupae ]
     [
       set invadedDroneCohortID saveWho
     ] ; miteOrganiser updates its value for the invadedDroneCohortID
   ]
end

; ****************************************************************************************************************

to WorkerPupaeDevProc

  ; ageing of cohort, oldest cohort may emerge and release mites
  ask pupaeCohorts
  [
    set age age + 1
    fd 1
    set numberDied 0
    set numberDied random-poisson (number * MORTALITY_PUPAE)
    if numberDied > number [ set numberDied number ]
      ; random mortality, based on Poisson distribution
    set number number - numberDied
    set number_healthy number_healthy - numberDied
      ; all pupae are healthy as infection takes place (in the model)
      ; at emergence - and if not..

    if number_infectedAsPupa > 0
    [
      set BugAlarm true
      show "BUG ALARM!!! number_infectedAsPupa > 0 in WorkerPupaeDevProcs!"
    ] ;  .. raise a bug alarm!
```

```
    if (numberDied > 0) and (TotalMites > 0)
    [
      MitesReleaseProc invadedByMiteOrganiserID ploidy numberDied "dyingBrood"
    ] ; variables correspond to [ miteOrganiserID ploidyMO diedBrood ]

    if age = EMERGING_AGE
    [
      if (number > 0) and (TotalMites > 0)
      [
        MitesReleaseProc invadedByMiteOrganiserID 2 0 "emergingBrood"
      ]  ; invadedByMiteOrganiserID ploidy = 2 numberDied = 0

      set NewIHbees number
      set NewIHbees_healthy number_healthy
    ]

    if age >= EMERGING_AGE [ die ]
  ]
end

; ****************************************************************************************************************


to DronePupaeDevProc

  ; ageing of cohort, oldest cohort may emerge and release mites
  ask dronePupaeCohorts
  [
    set age age + 1
    fd 1  ; turtle moves one step (display)
    set numberDied 0
    set numberDied random-poisson (number * MORTALITY_DRONE_PUPAE)
```

```
    if numberDied > number [ set numberDied number ]
    set number number - numberDied
    set number_healthy number_healthy - numberDied
      ; all pupae are healthy as infection takes place (in the model) at
      ; emergence - and if not..

    if number_infectedAsPupa > 0
    [
      set BugAlarm true
      show "BUG ALARM!!! number_infectedAsPupa > 0 in DronePupaeDevProcs!"
    ] ;  .. raise a bug alarm!
    if (numberDied > 0) and (TotalMites > 0)
    [
      MitesReleaseProc invadedByMiteOrganiserID ploidy numberDied "dyingBrood"
    ] ; variables correspond to [ miteOrganiserID ploidyMO diedBrood ]
    if age = DRONE_EMERGING_AGE
    [
      if (number > 0) and (TotalMites > 0)
      [
        MitesReleaseProc invadedByMiteOrganiserID 1 0 "emergingBrood"
      ]   ; invadedByMiteOrganiserID ploidy = 1 numberDied = 0
      set NewDrones number
      set NewDrones_healthy number_healthy ]
    if age >= DRONE_EMERGING_AGE [ die ]
  ]
end

; ***************************************************************************************************************************

to NewIHbeesProc

   create-IHbeeCohorts 1
```

```
  [
    set shape "circle"
    set number NewIHbees ; all new IH bees
    set number_healthy NewIHbees_healthy ; new, healthy IH bees
    set number_infectedAsPupa number - number_healthy
      ; the others were infected during pupal phase

    set number_infectedAsAdult 0
      ; adult workers hadn't had any chance to become infected so far..

    set age 0
    set color orange
    setxy 3 (- age - EMERGING_AGE - 1)
    set ploidy 2
  ]
end

; *****************************************************************************************************************


to NewDronesProc

  create-DroneCohorts 1
  [
   set shape "circle"
   set number NewDrones ; all new drones
   set number_healthy NewDrones_healthy ; new, healthy drones
   set number_infectedAsPupa number - number_healthy ; the others are infected
   set age 0
   set color grey
   setxy -5 (- age - DRONE_EMERGING_AGE - 1)
   set ploidy 1
  ]
```

```
end

; ***************************************************************************************************************

to AffProc
  ; calculates the actual age of first foraging on basis of nectar stores and
  ; brood/nurse ratio - called by WorkerIHbeesDevProc

  let affYesterDay Aff  ; the current (= yesterday's) Aff is saved
  let pollenTH  0.5
  let proteinTH 1
  let honeyTH 35 * (DailyHoneyConsumption / 1000) * ENERGY_HONEY_per_g
    ; min. desired honey store lasts for 35 days (arbitrarily chosen)
  let broodTH 0.1
  let foragerToWorkerTH 0.3  ; like in Beshers et al. 2001

  ; POLLEN criterion:
  if PollenStore_g / IdealPollenStore_g < pollenTH [ set Aff Aff - 1 ]

  ; PROTEIN criterion:
  if proteinFactorNurses < proteinTH [ set Aff Aff - 1 ]

  ; HONEY criterion:
  if HoneyEnergyStore < honeyTH [ set Aff Aff - 2 ]

  ; FORAGER TO WORKER criterion
  if (TotalIHbees > 0)
     and (TotalForagers / TotalIHbees < foragerToWorkerTH)
  [
    set Aff Aff - 1
  ]
```

```
  ; BROOD TO NURSES criterion:
  if ((TotalIHbees
      + TotalForagers * FORAGER_NURSING_CONTRIBUTION) * MAX_BROOD_NURSE_RATIO)
      > 0
    and
      TotalWorkerAndDroneBrood / ((TotalIHbees
      + TotalForagers * FORAGER_NURSING_CONTRIBUTION) * MAX_BROOD_NURSE_RATIO)
      > broodTH
  [
    set Aff Aff + 2
  ]

  ; to reduce strong deviations from the base Aff:
  if affYesterDay < AFF_BASE - 7 [ set Aff Aff + 1 ]
  if affYesterDay > AFF_BASE + 7 [ set Aff Aff - 1 ]

  ; Aff can be changed only by +-1 per day:
  if Aff < affYesterDay [ set Aff affYesterDay - 1 ]
  if Aff > affYesterDay [ set Aff affYesterDay + 1 ]

  ; MIN and MAX values for Aff:
  if Aff < MIN_AFF [ set Aff MIN_AFF ]
  if Aff > MAX_AFF [ set Aff MAX_AFF ]

end

; ****************************************************************************************************
```

```
to WorkerIHbeesDevProc
 ; ageing of IH bees, mortality for healthy and infected IH-workers,
 ; calls CalculateAffProc, calculation of # new foragerSquadrons

 let overagedIHbees 0
   ; bees with age > Aff but have to remain in the last IH cohort, as number < SQUADRON_SIZE

 AffProc
   ; in the AffProc today's age of first foraging (Aff) is calculated
 foreach reverse sort IHbeeCohorts
   ; cohorts have to be asked in order of their age (i.e. in reverse order of
   ; their "who") otherwise over-aged bees vanish with a 50% chance
 [
   ask ?
   [
     let deathsCounter 0
       ; # of bees dying in this cohort at current time step

     set age age + 1
     fd 1 ; turtle moves one step (display)

     ; MORTALITY
     ; healthy bees:
     set deathsCounter random-poisson (number_healthy * MORTALITY_INHIVE)
     if deathsCounter > number_healthy [ set deathsCounter number_healthy ]
       ; random mortality, based on Poisson distribution

     set number_healthy number_healthy - deathsCounter
       ; deathCounter: dead HEALTHY bees

     ; infectedAsPupa:
     set deathsCounter
```

```
  random-poisson (number_infectedAsPupa * MORTALITY_INHIVE_INFECTED_AS_PUPA)
if deathsCounter > number_infectedAsPupa
[
  set deathsCounter number_infectedAsPupa
]  ; random mortality, based on Poisson distribution

set number_infectedAsPupa number_infectedAsPupa - deathsCounter
  ; deathCounter now: dead INFECTED bees

; infectedAsAdults:
set deathsCounter
  random-poisson (number_infectedAsAdult * MORTALITY_INHIVE_INFECTED_AS_ADULT)
if deathsCounter > number_infectedAsAdult
[
  set deathsCounter number_infectedAsAdult
]  ; random mortality, based on Poisson distribution
set number_infectedAsAdult number_infectedAsAdult - deathsCounter
  ; deathCounter now: dead INFECTED bees

set deathsCounter number - number_healthy
  - number_infectedAsPupa - number_infectedAsAdult
  ; deathCounter is now set to the TOTAL number of dead bees

set number number - deathsCounter
  ; # of bees in this cohort is reduced by # of dead bees

set DeathsAdultWorkers_t DeathsAdultWorkers_t
  + deathsCounter
  ; sums up # of adult workers dying in current timestep to calculate
  ; mean lifespan of adult bees

set SumLifeSpanAdultWorkers_t SumLifeSpanAdultWorkers_t
```

```
  + (deathsCounter * age)
  ; sums up lifespan of adult workers dying in current timestep

 set InhivebeesDiedToday DeathsAdultWorkers_t


; onset of foraging
 if age >= Aff
 [
  ; new healthy foragerSquadrons:
  set NewForagerSquadronsHealthy
    floor (number_healthy / SQUADRON_SIZE) + NewForagerSquadronsHealthy
  set overagedIHbees number_healthy mod SQUADRON_SIZE
  ask IHbeeCohorts with [ age = Aff - 1 ]
  [
    set number number + overagedIHbees
    set number_healthy number_healthy + overagedIHbees
  ]
    ; overaged bees would vanish here without "reverse sort", as there
    ; might be no IHbeeCohort with age = Aff - 1! (50% chance)

  ; new foragerSquadrons, which were infected as pupae:
  set NewForagerSquadronsInfectedAsPupae
    floor (number_infectedAsPupa / SQUADRON_SIZE)
         + NewForagerSquadronsInfectedAsPupae

  set overagedIHbees number_infectedAsPupa mod SQUADRON_SIZE
  ask IHbeeCohorts with [ age = Aff - 1 ]
  [
    set number number + overagedIHbees
    set number_infectedAsPupa number_infectedAsPupa + overagedIHbees
  ]
    ; overaged bees would vanish here without "reverse sort", as there might
```

```
          ; be no IHbeeCohort with age = Aff - 1! (50% chance)

        ; new infectedAsAdults foragerSquadrons:
        set NewForagerSquadronsInfectedAsAdults
          floor (number_infectedAsAdult / SQUADRON_SIZE)
               + NewForagerSquadronsInfectedAsAdults

        set overagedIHbees number_infectedAsAdult mod SQUADRON_SIZE
        ask IHbeeCohorts with [ age = Aff - 1 ]
        [
          set number number + overagedIHbees
          set number_infectedAsAdult number_infectedAsAdult + overagedIHbees
        ]
          ; overaged bees would vanish here without "reverse sort", as there might
          ; be no IHbeeCohort with age = Aff - 1! (50% chance)
                    ]
      if age >= Aff
      [
        set plabel ""
        die
      ]
    ] ; ask ?
  ] ; foreach reverse sort IHbeeCohorts
end

; ******************************************************************************************************************

to DronesDevProc

  ; ageing of cohort, mortality for healthy and infected drones
  ask DroneCohorts [
    fd 1
```

```
    set age age + 1

    ; MORTALITY:
    set number_healthy (number_healthy -
      random-poisson (number_healthy * MORTALITY_DRONES))
    if number_healthy < 0 [ set number_healthy 0 ]

    set number_infectedAsPupa
     ( number_infectedAsPupa
      - random-poisson (number_infectedAsPupa * MORTALITY_DRONES_INFECTED_AS_PUPAE) )

    if number_infectedAsPupa < 0 [ set number_infectedAsPupa 0 ]
    set number number_healthy + number_infectedAsPupa
     ; total number of drones = healthy + infected drones
    if age >= DRONE_LIFESPAN [ die ]
  ]
end

; ****************************************************************************************************************

to BroodCareProc
  ; checks if enough nurses are present and, if not, kills excess of drone and
  ; worker brood; order of dying: 1. droneEggCohorts 2. droneLarvaeCohorts
  ; 3. eggCohorts 4. larvaeCohorts 5. dronePupaeCohorts 6. pupaeCohorts

  let lackNurses false
    ; all kind of brood might die due to lack of nurse bees..
  let lackProtein false
    ; .. or (drone&worker) LARVAE may die due to lack of protein in brood food

  if ticks > 1 [ CountingProc ]
```

```
  ; current # of IH-bees and brood, cannot be called in time step 1, as
  ; counting foragerSquadrons results wrongly in 0

set ExcessBrood
  ceiling ( TotalWorkerAndDroneBrood
  - (TotalIHbees + TotalForagers * FORAGER_NURSING_CONTRIBUTION)
  *  MAX_BROOD_NURSE_RATIO )
    ; rounded up! totalWorkerDroneBrood: all brood stages of drones & workers;
    ; Nursing: also foragers are assumed to contribute (partly) to brood care

ifelse ExcessBrood > 0
[
  set lackNurses true
  ask signs with [shape = "beelarva_x2"]
  [
    show-turtle
    set label ExcessBrood
  ]
]
[
  ask signs with [shape = "beelarva_x2"]
  [
    hide-turtle
  ]
]

let starvedBrood ceiling ((TotalDroneLarvae + TotalLarvae) * (1 - ProteinFactorNurses))
  ; larvae require protein and may die if jelly contains not enough proteins

if starvedBrood > 0 [ set lackProtein true ]
if starvedBrood > ExcessBrood [ set ExcessBrood starvedBrood ]
  ; excess of brood is either determined by lack of nurses or lack of protein
```

```
set LostBroodToday LostBroodToday + ExcessBrood
set LostBroodTotal LostBroodTotal + ExcessBrood
let stillToKill ExcessBrood
  ; keeps track of the amount of brood that is still to be killed

if ExcessBrood > 0
[ ; whenever a brood cell dies, the corresponding miteOrganiser is updated in the
  ; releaseMitesProc! (only for pupae and oldest larvae as eggs and young larvae are
  ; not invaded by mites

  if lackNurses = true
  [
    foreach reverse sort DroneEggCohorts
    [
      ask ?    ; young drone eggs die first if not enough nurses are available
      [ while [ (stillToKill * number) > 0 ]
        [
          set number number - 1
          set stillToKill stillToKill - 1
        ]
      ]
    ]
  ]

  if lackNurses = true or lackProtein = true
  [
    foreach reverse sort DroneLarvaeCohorts
    [
      ask ?
      [
        while [ (stillToKill * number) > 0 ]
```

```
      [ set number number - 1 set stillToKill stillToKill - 1
        if age > INVADING_DRONE_CELLS_AGE and (TotalMites > 0)
        [
          MitesReleaseProc invadedByMiteOrganiserID ploidy 1 "dyingBrood"
        ]
          ; Died brood: always 1! calls releaseMitesProc and transfers variables
          ; (correspond to [ miteOrganiserID ploidyMO diedBrood ])
      ]
    ]
  ]
] ; if lackNurses = true or lackProtein = true

if lackNurses = true
[
  foreach reverse sort EggCohorts
  [
    ask ?
    [
      while [ (stillToKill * number) > 0 ]
      [
        set number number - 1
        set stillToKill stillToKill - 1
      ]
    ]
  ]
] ;if lackNurses = true
; (stillToKill * number): BOTH, number AND stillToKill have to be > 0 to continue "while"

if lackNurses = true or lackProtein = true
[
  foreach reverse sort larvaeCohorts
  [
```

```
    ask ?
    [
      while [ (stillToKill * number) > 0 ]
      [
        set number number - 1 set stillToKill stillToKill - 1
        if age > INVADING_WORKER_CELLS_AGE and (TotalMites > 0)
        [
          MitesReleaseProc invadedByMiteOrganiserID ploidy 1 "dyingBrood"
        ]
          ; calls releaseMitesProc and transfers variables (correspond
          ; to [ miteOrganiserID ploidyMO diedBrood ])
      ]
    ]
  ]
] ; if lackNurses = true or lackProtein = true

if lackNurses = true
[
  foreach reverse sort DronePupaeCohorts
  [
    ask ?
    [
      while [ (stillToKill * number) > 0 ]
      [
        ifelse random number <= number_healthy ; choose a random pupal cell
          [ set number_healthy number_healthy - 1 set number number - 1  ]
            ; IF pupa is healthy, then number_healthy and (total) number are decreased by one
          [ set number_infectedAsPupa number_infectedAsPupa - 1 set number number - 1 ]
            ; ELSE number_infectedAsPupa and (total) number are decreased by one
        set stillToKill stillToKill - 1
        if (TotalMites > 0)
        [
```

```
          MitesReleaseProc invadedByMiteOrganiserID ploidy 1 "dyingBrood"
        ]
      ]
    ]
  ]
] ;    if lackNurses = true

if lackNurses = true
[
  foreach reverse sort pupaeCohorts
  [
    ask ?
    [
      while [ (stillToKill * number) > 0 ]
      [
        ifelse random number <= number_healthy  ; choose a random pupal cell
          [ set number_healthy number_healthy - 1 set number number - 1  ]
            ; IF pupa is healthy, then number_healthy and (total) number are decreased by one
          [ set number_infectedAsPupa number_infectedAsPupa - 1 set number number - 1 ]
            ; ELSE number_infectedAsPupa and (total) number are decreased by one
        set stillToKill stillToKill - 1
        if (TotalMites > 0)
        [
          MitesReleaseProc invadedByMiteOrganiserID ploidy 1 "dyingBrood"
        ]
      ]
    ]
  ]
] ; if lackNurses = true

if stillToKill > 0
[
```

```
      set BugAlarm true
      output-show (word ticks " BUG ALARM! stillToKill > 0")
    ]
  ] ; end IF ExcessBrood > 0

end

; ******************************************************************************************************************

to DrawIHcohortsProc
  ; # bees in IH cohorts (workers & drones, brood & adults) are drawn as coloured bars

  ask (turtle-set eggCohorts larvaeCohorts pupaeCohorts)
  [                                                    ;   WORKERS
    set heading 90
    fd 1
    repeat ceiling( 10 * number / STEPWIDTH)
    [
      fd 0.1
      set pcolor color
    ]
    set heading 180 setxy 3 (- age)
  ]

  ask IHbeeCohorts
  [
    set heading 90
    fd 1
    repeat ceiling( 10 * number_healthy / STEPWIDTH)
    [
      fd 0.1
```

```
    set pcolor color
  ]
  repeat ceiling( 10 * number_infectedAsAdult / STEPWIDTH)
  [
    fd 0.1
    set pcolor (color - 1)
  ]
  repeat ceiling( 10 * number_infectedAsPupa / STEPWIDTH)
  [
    fd 0.1
    set pcolor (color - 2)
    ]
  set heading 180
  setxy 3 (- age - EMERGING_AGE - 1)
] ; ask IHbeeCohorts

ask (turtle-set droneEggCohorts droneLarvaeCohorts dronePupaeCohorts)  ;   DRONES
[
  set heading 270
  repeat ceiling( number / STEPWIDTHdrones)
  [
    fd 1
    set pcolor color
  ]
  set heading 180
  setxy -5 (- age)
]

ask DroneCohorts
[
  set heading 270 repeat ceiling( number_healthy / STEPWIDTHdrones)
  [
```

```
    fd 1
    set pcolor color
    ]
  repeat ceiling( number_infectedAsPupa / STEPWIDTHdrones)
  [
    fd 1
    set pcolor (color - 2)
  ]
  set heading 180
  setxy -5 (- age - DRONE_EMERGING_AGE - 1)
 ]
end



; ==================================================================

; IBM FORAGING SUBMODEL =====

; ==================================================================

; ***************************************************************************************************************


to Start_IBM_ForagingProc
 ; controls the number of foraging trips per day, calls ForagingRoundProc

 let continueForaging true
   ; foraging is continued until it is stopped
 let meanTripDuration 0
 let summedTripDuration 0
 let HANGING_AROUND SEARCH_LENGTH_M / FLIGHT_VELOCITY
   ; [s] duration of a foraging round if all foragers are resting
```

```
  ; (= time for unsuccessful search flight)
let ageLaziness 100
; [d] min. age to allow foragers being lazy
ForagersDevelopmentProc
  ; called before creation of new foragers to avoid ageing by 2d at creation

NewForagersProc
ask foragerSquadrons
[ ; Laziness: lazy bees won't forage and can't be recruited on that day.
  ; applies only to older bees and if the honey store is not too small
  if age >= ageLaziness and
    random-float 1 < ProbLazinessWinterbees and ; ProbLazinessWinterbees: default: 0!
    random-float 1 < (HoneyEnergyStore / DecentHoneyEnergyStore)
   [
     set activity "lazy"
   ]
]

set ForagingSpontaneousProb Foraging_ProbabilityREP
  ; the probability for a resting forager to start spontaneously foraging in a single foraging
  ; round today is calculated in "to-report Foraging_ProbabilityREP "

set ForagingRounds 0
  ; counter of the foraging rounds
ask foragerSquadrons
[
  set activityList [ ]
  ; activityList records all activities of a forager during the day
]

; always "season" as SEASON_START = 1 & SEASON_STOP = 365
if ( Day >= SEASON_START )
```

```
    and ( Day <= SEASON_STOP )
     ; foraging takes only place during season and while honey store not
     ; (almost) full (0.95: to avoid foraging, when honey cannot be stored)..
    and
     ( HoneyEnergyStore < 0.95 * MAX_HONEY_ENERGY_STORE
       or PollenStore_g < IdealPollenStore_g )
     ;  ..or when pollen is needed
    and DailyForagingPeriod > 0

[
  while [ continueForaging = true ]
    ; .. and only for a certain time (=DailyForagingPeriod), which is checked
    ; via "continueForaging"
  [
    ask foragerSquadrons
    [
      set activityList lput ForagingRounds activityList
      ; the ForagingRounds is added to a foragers activityList
    ]
    ForagingRoundProc
      ; call ForagingRoundProc, which calls all procedures involved in foraging

    set ForagingRounds ForagingRounds + 1
      ; # foraging rounds is increased

    ifelse ColonyTripForagersSum > 0
      [ set meanTripDuration ColonyTripDurationSum / ColonyTripForagersSum ]
        ; IF > 0 (i.e. if at least 1 foraging trip has taken place):  calculate the average time
        ; a forager needed for its trip in this round
      [ set meanTripDuration HANGING_AROUND ]
        ; ELSE: if no one goes foraging: foraging round lasts "HANGING_AROUND" seconds
```

```
      set summedTripDuration ( summedTripDuration + meanTripDuration )
        ; mean trip durations are summed up

      ; if the duration of all foraging rounds summed up is larger than DailyForagingPeriod
      ; then foraging ends for today
      if summedTripDuration >= DailyForagingPeriod
      [
        set continueForaging false
      ]  ; until the total time >= DailyForagingPeriod

      if ((Details = true) and (continueForaging = true))
      [
        if WriteFile = true [ WriteToFileProc ]
      ]
        ; if Details & WriteFile true: results are recorded in Output file after each foraging round (trip)
    ]
  ]

  ForagersLifespanProc
    ; mortality of foragers due to max. lifespan, max. km or in-hive mortality risk

  ask foragerSquadrons
  [
    set activity "resting"
    set activityList lput "End" activityList
  ]     ; after foraging is completed for today, all foragers do rest
end;

;****************************************************************************
;  *************** PARAMETERIZATION FLOWER PATCH  ***********************
;****************************************************************************
```

```
to CreateFlowerPatchesProc
 ; creates 2  flower patches ("red" & "green"),

 set N_FLOWERPATCHES  2 ; 2
 if readInfile = true
 [
   set bugAlarm true
   show "BugAlarm in CreateFlowerPatchesProc! Check read-in!"
 ]
 create-flowerPatches N_FLOWERPATCHES
 [
   set patchType "GreenField"
   set distanceToColony DISTANCE_G ;1500  ; [m]
   set xcorMap distanceToColony
   set size_sqm 100000
   set quantityMyl  QUANTITY_G_l * 1000 * 1000; [microlitres]
   set amountPollen_g POLLEN_G_kg * 1000 ;10000 ; 10kg = 10000g
     ; total amount of pollen available at this patch

   if SeasonalFoodFlow = true
   [
     set quantityMyl FlowerPatchesMaxFoodAvailableTodayREP who "Nectar"
     set amountPollen_g FlowerPatchesMaxFoodAvailableTodayREP who "Pollen"
   ]

   set nectarConcFlowerPatch CONC_G
     ;  mean nectar concentration returned to colony ca. 1.4 (assessed from Seeley (1986), Fig 2)

   set detectionProbability DETECT_PROB_G
   set shape "fadedFlower"
   set color green
   set size 4
```

```
    ifelse distanceToColony <= 5500
      [ setxy (15.1 + (distanceToColony / 250) )  3 ]  ; IF (distance)
      [ setxy 39.5 3 ]                                ; ELSE (distance)
  ] ; create-flowerPatches N_FLOWERPATCHES

  ask flowerPatch 0
  [
    set patchType "RedField"
    set distanceToColony DISTANCE_R  ; [m]   ; RED PATCH
    set xcorMap -1 * distanceToColony
    set quantityMyl QUANTITY_R_l * 1000 * 1000 ; [microlitres]
    set amountPollen_g POLLEN_R_kg * 1000 ; [g]

    if SeasonalFoodFlow = true
    [
      set quantityMyl FlowerPatchesMaxFoodAvailableTodayREP who "Nectar"
      set amountPollen_g FlowerPatchesMaxFoodAvailableTodayREP who "Pollen"
    ]

    set nectarConcFlowerPatch CONC_R
    set detectionProbability DETECT_PROB_R
    set color red

    ifelse distanceToColony <= 5500
     [ setxy (14.9 - (distanceToColony / 250) )  3 ]
     [ setxy -7.5 3 ]
  ]

  FlowerPatchesUpdateProc
end;

;*****************************************************************************
```

```
;  ************** PARAMETERIZATION FLOWER PATCHES FROM FILES  ********
;**************************************************************************


to Create_Read-in_FlowerPatchesProc
  ; copy of CreateFlowerPatchesProc but data are read from input file
  ; calculates derived values (e.g. EEF, flight costs etc)

  let counter 0
  set TodaysAllPatchesList []
    ; shorter list, contains data of all patches, but only for today

  set TodaysSinglePatchList []
    ; short list, contains data of a single patch for today

  set counter Day
    ; counter: to chose only the values for today from the complete
    ; (all days, all patches) list

  repeat N_FLOWERPATCHES
  [
    ; todays data for ALL N_FLOWERPATCHES flower patches are saved in a
    ; new, shorter list (= todaysAllPatchesList)

    set TodaysSinglePatchList (item counter AllDaysAllPatchesList)
      ; this new, shorter list (= todaysAllPatchesList) is comprised of very
      ; short lists (=todaysSinglePatchList) that contain only the data of the
      ; current patch and only for today

    set todaysAllPatchesList fput TodaysSinglePatchList todaysAllPatchesList
      ; fput: faster as lput! (Netlogo 4) however: list is in reversed order!
```

```
set counter counter + 365
create-flowerPatches 1
[
 set oldPatchID item 2 TodaysSinglePatchList
   ; refers to patch number of crop maps from a landscape module,
   ; an optional external tool to read in and analyse maps of food patches

 set patchType item 3 TodaysSinglePatchList ; e.g. Oilseed rape
 set distanceToColony item 4 TodaysSinglePatchList  ; [m]
 set xcorMap item 5 TodaysSinglePatchList ; x coordinate
 set ycorMap item 6 TodaysSinglePatchList ; y coordinate
 set size_sqm item 7 TodaysSinglePatchList  ; patch area [m^2]
 set amountPollen_g item 8 TodaysSinglePatchList ; [g]
 set nectarConcFlowerPatch item 9 todaysSinglePatchList  ; [mol/l]
 set quantityMyl  (item 10 TodaysSinglePatchList) * 1000 * 1000 ; [microlitres]

 let calcDetectProb item 11 TodaysSinglePatchList
 ; calculated in "2_BEEHAVE_FoodFlow"-Tool on basis of distance
 ; (if this input file is created by "BEEHAVE_FoodFlow")

 let modelledDetectProb item 12 TodaysSinglePatchList
   ; modelleded in "3_BEEHAVE_LANDSCAPE" with individual scouts
   ; exploring a 2-dim landscape

 ifelse ModelledInsteadCalcDetectProb = true
   [ set detectionProbability modelledDetectProb ]
   [ set detectionProbability calcDetectProb ]

 set shape "flower"
 set size 1 + (sqrt size_sqm) /  1000
 setxy (distanceToColony / 300) 3
]
```

```
  ] ; END of "repeat N_FLOWERPATCHES"
  FlowerPatchesUpdateProc
  set TodaysAllPatchesList reverse TodaysAllPatchesList
    ; to correct the reversed order, caused by the fput command

end;

; *****************************************************************************************************************************

to FlowerPatchesUpdateProc

  let energyFactor_onFlower 0.2 ; (0.2)
    ;  reflects reduced energy consumption while bee is sitting on the flower
    ; to collect nectar or pollen;
    ; Kacelnik et al 1986 (BES:19): 0.3 (rough estimation, based on Nunez 1982)


  ; handling time:
  ask flowerPatches
  [
    if ReadInfile = false
    [
      ifelse ConstantHandlingTime = true
        [
          set handlingTimeNectar TIME_NECTAR_GATHERING  ; IF: handling time constant
          set handlingTimePollen TIME_POLLEN_GATHERING
        ]
        [
          if quantityMyl > 0
          [
            set handlingTimeNectar
              TIME_NECTAR_GATHERING *
```

```
        ((FlowerPatchesMaxFoodAvailableTodayREP who "Nectar") / quantityMyl)
      ] ; ELSE: handling time dependent on proportion of nectar or pollen left

      if amountPollen_g > 0
      [
        set handlingTimePollen TIME_POLLEN_GATHERING
        * ((FlowerPatchesMaxFoodAvailableTodayREP who "Pollen") / amountPollen_g)
      ]
    ]
  ] ;     if ReadInfile = false

  if ReadInfile = true
  [
    set TodaysSinglePatchList item who TodaysAllPatchesList
    ifelse ConstantHandlingTime = true
      [ ; IF CONSTANT handling time:
        set handlingTimeNectar item 13 TodaysSinglePatchList
         ;  item 13: handling time nectar
        set handlingTimePollen item 14 TodaysSinglePatchList
      ] ;   item 14: handling time pollen
      [
        ; ELSE: if handling time is NOT constant:
        if quantityMyl > 0   ; nectar handling time
        [
          set handlingTimeNectar (
            item 13 TodaysSinglePatchList) *
            ((item 10 TodaysSinglePatchList) * 1000 * 1000) / quantityMyl
        ] ; item 13: NectarGathering_s, item 10: quantityNectar_l

        if amountPollen_g > 0   ; pollen handling time
        [
          set handlingTimePollen
```

```
        (item 14 TodaysSinglePatchList)
        * ((item 8 TodaysSinglePatchList) / amountPollen_g)
      ]  ; item 14: PollenGathering_s; item 8: quantityPollen_g
    ]
  ] ; if ReadInfile = true


; flight costs & EFF
  set flightCostsNectar
    ( 2 * distanceToColony * FLIGHTCOSTS_PER_m)
      + ( FLIGHTCOSTS_PER_m * handlingTimeNectar
      * FLIGHT_VELOCITY * energyFactor_onFlower ) ; [kJ] = [m*kJ/m + kJ/m * s * m/s]

  set flightCostsPollen
    ( 2 * distanceToColony * FLIGHTCOSTS_PER_m)
      + ( FLIGHTCOSTS_PER_m * handlingTimePollen
      * FLIGHT_VELOCITY * energyFactor_onFlower )

  set EEF ((nectarConcFlowerPatch * CROPVOLUME
        * ENERGY_SUCROSE) - flightCostsNectar) / flightCostsNectar
    ;  Energetic Efficiency of the flowerPatch


; trip duration:
  set tripDuration 2 * distanceToColony * (1 / FLIGHT_VELOCITY )
    + handlingTimeNectar
    ; duration of nectar foraging trip depends on speed, 2*distance + time to
    ; collect nectar from the flowers

  set tripDurationPollen 2 * distanceToColony
    * (1 / FLIGHT_VELOCITY ) + handlingTimePollen
    ; duration of pollen foraging  trip depends on speed, 2*distance + time to
    ; collect pollen from the flowers
```

```
 ; mortality:
   set mortalityRisk 1 - ((1 - MORTALITY_FOR_PER_SEC) ^ tripDuration)   ; nectar foragers
   set mortalityRiskPollen 1 - ((1 - MORTALITY_FOR_PER_SEC) ^ tripDurationPollen) ; pollen foragers
        ;                                                  DANCING:
   set danceCircuits DANCE_SLOPE * EEF + DANCE_INTERCEPT  ; derived from Seeley 1994

   if danceCircuits < 0 [ set danceCircuits 0 ]
   if danceCircuits > MAX_DANCE_CIRCUITS [ set danceCircuits MAX_DANCE_CIRCUITS ]
     ; MAX_DANCE_CIRCUITS: ca. 100 (Seeley, Towne 1992)

   if SimpleDancing = true
   [
     ifelse EEF > 20
       [ set danceCircuits 40 ] ; IF
       [ set danceCircuits 0 ]  ; ELSE
   ]

   if AlwaysDance = true [ set danceCircuits 40 ]
     ; in this case, foragers always dance for their patch,
     ; irrespective of its quality

   set danceFollowersNectar danceCircuits * 0.05
     ; Seeley, Reich, Tautz (2005): "0.05 recruits per waggle run (see Fig. 3)"
  ] ; ask flowerPatches
end

; ************************************************************************************************************************

to ForagingRoundProc

  ;  CALLED BY Start_IBM_ForagingProc calls Procedures involved in each foraging trip
  ; and does foraging related plots
```

```
set ColonyTripDurationSum 0
set ColonyTripForagersSum 0
 ; used to calculated duration of this foraging round
set DecentHoneyEnergyStore (TotalIHbees + TotalForagers ) * 1.5 * ENERGY_HONEY_per_g
 ; DecentHoneyEnergyStore reflects the amount of energy a colony should store
 ; to survive the winter, based on the assumption that a bee consumes ca. 1.5g honey during winter
if DecentHoneyEnergyStore = 0
[
  set DecentHoneyEnergyStore 1.5 * ENERGY_HONEY_per_g
] ; to avoid division by 0

; Proportion of pollen foragers:
set ProbPollenCollection (1 - PollenStore_g / IdealPollenStore_g)
  * MAX_PROPORTION_POLLEN_FORAGERS
  ; (Pollen foragers: ~ 0-90% of all foragers: Lindauer 1952)

if HoneyEnergyStore / DecentHoneyEnergyStore < 0.5
[
  set ProbPollenCollection ProbPollenCollection
  * (HoneyEnergyStore / DecentHoneyEnergyStore)
]

FlowerPatchesUpdateProc
Foraging_start-stopProc  ; some foragers might spontaneously start foraging
Foraging_searchingProc    ; unexperienced foragers search new flower patch
Foraging_collectNectarPollenProc  ; succesful scouts and experienced Foragers gather nectar
Foraging_flightCosts_flightTimeProc  ; energy costs for flights and trip duration
Foraging_mortalityProc  ; foragers might die on their way back to the colony
Foraging_dancingProc    ; successful foragers might dance..
Foraging_unloadingProc  ; ..and unload their crop & increase colony's honey store
```

```
let foragersAlive SQUADRON_SIZE * count foragerSquadrons

let currentNectarForagers
  SQUADRON_SIZE * count foragerSquadrons with
    [activity = "expForaging" and pollenForager = false]

let currentPollenForagers
  SQUADRON_SIZE * count foragerSquadrons with
    [activity = "expForaging" and pollenForager = true]

let currentResters
  SQUADRON_SIZE * count foragerSquadrons with
    [activity = "resting"]

let currentScouts
  SQUADRON_SIZE * count foragerSquadrons with
    [activity = "searching"]

let currentRecruits
  SQUADRON_SIZE * count foragerSquadrons
    with [activity = "recForaging"]

let currentLazy
  SQUADRON_SIZE * count foragerSquadrons
    with [activity = "lazy"]

if sqrt ((foragersAlive - currentNectarForagers ; to avoid bugAlarm cuased by numeric inaccuracy!
  - currentPollenForagers - currentResters
  - currentScouts - currentRecruits - currentLazy) ^ 2) > 0.0000000001
[
  set BugAlarm true show "BUG ALARM in ForagingRoundProc: wrong number of forager activities!"
]
```

```
if ShowAllPlots = true
[
 let i 1
 while [ i <= N_GENERIC_PLOTS ]
 [
   let plotname (word "Generic plot " i)
     ; e.g. "Generic plot 1"

   set-current-plot plotname
   if (i = 1 and GenericPlot1 = "active foragers today [%]")
   or (i = 2 and GenericPlot2 = "active foragers today [%]")
   or (i = 3 and GenericPlot3 = "active foragers today [%]")
   or (i = 4 and GenericPlot4 = "active foragers today [%]")
   or (i = 5 and GenericPlot5 = "active foragers today [%]")
   or (i = 6 and GenericPlot6 = "active foragers today [%]")
   or (i = 7 and GenericPlot7 = "active foragers today [%]")
   or (i = 8 and GenericPlot8 = "active foragers today [%]")
   [
     create-temporary-plot-pen "active%"
     set-plot-y-range  0 110
     set-plot-pen-mode 0 ; 0: lines
     ifelse foragersAlive > 0
       [
         plot (100 * SQUADRON_SIZE ; % active foragers of all foragers CURRENTLY alive
                    * (count foragersquadrons
                             with [ activity != "resting" and activity != "lazy"] )) / foragersAlive
       ]   ; i.e. with activities = "searching", "recForaging" or "expForaging"
       [
         plot 0
       ]
     create-temporary-plot-pen "deaths%"
```

```
  set-plot-pen-color red
  plot 100 * DeathsForagingToday ; cumulative deaths as % of todays' INITIAL foraging force
          / (foragersAlive + DeathsForagingToday)
 ]

 if (i = 1 and GenericPlot1 = "foragers today [%]")
 or (i = 2 and GenericPlot2 = "foragers today [%]")
 or (i = 3 and GenericPlot3 = "foragers today [%]")
 or (i = 4 and GenericPlot4 = "foragers today [%]")
 or (i = 5 and GenericPlot5 = "foragers today [%]")
 or (i = 6 and GenericPlot6 = "foragers today [%]")
 or (i = 7 and GenericPlot7 = "foragers today [%]")
 or (i = 8 and GenericPlot8 = "foragers today [%]")
[
  create-temporary-plot-pen "nectar"
  set-plot-pen-color yellow
  set-plot-pen-mode 0 ; 0: lines
  set-plot-y-range 0 100
  ifelse foragersAlive > 0
    [   plotxy ForagingRounds (100 * currentNectarForagers) / foragersAlive
      create-temporary-plot-pen "pollen"
        set-plot-pen-color orange
        plotxy ForagingRounds (100 * currentPollenForagers) / foragersAlive
      create-temporary-plot-pen "scouts"
        set-plot-pen-color green
        plotxy ForagingRounds (100 * currentScouts) / foragersAlive
      create-temporary-plot-pen "resters"
        set-plot-pen-color brown
        plotxy ForagingRounds (100 * currentResters) / foragersAlive
      create-temporary-plot-pen "lazy"
        plotxy ForagingRounds (100 * currentLazy) / foragersAlive
      create-temporary-plot-pen "recruits"
```

```
          set-plot-pen-color blue
          plotxy ForagingRounds (100 * currentRecruits) / foragersAlive
        ]
        [
          plotxy ForagingRounds 0
         create-temporary-plot-pen "pollen"
          set-plot-pen-color orange
          plotxy ForagingRounds 0
         create-temporary-plot-pen "scouts"
          set-plot-pen-color green
          plotxy ForagingRounds 0
         create-temporary-plot-pen "resters"
          set-plot-pen-color brown
          plotxy ForagingRounds 0
         create-temporary-plot-pen "lazy"
          plotxy ForagingRounds 0
         create-temporary-plot-pen "recruits"
          set-plot-pen-color blue
          plotxy ForagingRounds 0
        ]
    ]  ; END:  if plotChoice = "foragers today [%]"

   set i i + 1
  ]

  ] ; if ShowAllPlots = true
end

; ****************************************************************************************************************
```

```
to ForagersDevelopmentProc
  ; foragers age by 1 day, forager turtles move forward
  ask foragerSquadrons
  [
    set age age + 1
    fd 1.8   ; movement on GUI
  ]
end

; ********************************************************************************************************

to NewForagersProc
  ; creates foragerSquadrons as turtles, based on # in-hive bees developing into foragers

  let foragerSquadronsToBeCreated
     NewForagerSquadronsHealthy
     + NewForagerSquadronsInfectedAsPupae
     + NewForagerSquadronsInfectedAsAdults
  let newCreatedBees 0

  create-foragerSquadrons foragerSquadronsToBeCreated
  [
    set newCreatedBees newCreatedBees + 1
    ifelse ticks = 1
      [
        set age 100 + random 60 ; age of initial foragers: 100d + 0..59d
        setxy 40 9
        set color grey
        set size 2
        set heading 90
        set shape "bee_mb_1"
```

```
    set mileometer random (MAX_TOTAL_KM / 4)
  ]  ; IF 1st time step: (=initial bees): travelled distace equally distributed,
     ; (winterbees have done only little foraging in autumn!)

  [ ; ELSE: all other foragers
    set age Aff
    setxy (-20 + age) 9
    set color grey
    set size 2
    set heading 90
    set shape "bee_mb_1"
  ]

set activity "resting"
set activityList [ ]
set cropEnergyLoad 0 ; [kJ] no nectar in the crop yet
set collectedPollen 0 ; [g] no pollen pellets
set knownNectarPatch -1  ; -1 = no nectar Flower patch known
set knownPollenPatch -1 ; -1 = no pollen Flower patch known
set pollenForager false
  ; foragers are nectar foragers except they decide to collect pollen

; creation of HEALTHY and INFECTED foragers:
set infectionState "healthy"
  ; possible infection states are: "healthy" "infectedAsPupa" "infectedAsAdult"

if newCreatedBees > NewForagerSquadronsHealthy
[
  set infectionState "infectedAsPupa"
  set ycor ycor - 3
]  ; foragers infected as pupa are created
```

```
    if newCreatedBees > (NewForagerSquadronsHealthy + NewForagerSquadronsInfectedAsPupae)
    [
      set infectionState "infectedAsAdult"
      set ycor ycor + 1.5
    ] ; foragers infected as adults are created
  ] ; create-foragerSquadrons

  ; the toal number of ever produced foragers is recorded and can be used as output:
  set SummedForagerSquadronsOverTime
     SummedForagerSquadronsOverTime
     + NewForagerSquadronsHealthy
     + NewForagerSquadronsInfectedAsPupae
     + NewForagerSquadronsInfectedAsAdults

  ; no more new foragers have to be created in this time step:
  set NewForagerSquadronsHealthy 0
  set NewForagerSquadronsInfectedAsPupae 0
  set NewForagerSquadronsInfectedAsAdults 0
end;

; *********************************************************************************************************************************


to-report Foraging_PeriodREP
  let foragingPeriod_s -1
  let foragingHoursList [ ]
  ; "foragingPeriod" = HOURS SUNSHINE ON DAYS WITH Tmax > 15degC

  ; 2000: from weather data Berlin, Germany (DWD), (1.1.-31.12.2000);
  let foragingHoursListBerlin2000
    [ 0 0 0 0 0 0 0 0 0 0 0 0 0 0 0 0 0 0 0 0 0 0 0 0 0 0 0 0 0 0 0
      0 0 0 0 0 0 0 0 0 0 0 0 0 0 0 0 0 0 0 0 0 0 0 0 0 0 0 7.2
```

```
   0 0 0 0 0 0 0 0 0 0 0 0 0 0 0 0 0 0 0 0 0 0 0 0 0 0 0 2.5 0 0 0 0
   0 0 0 10.7 0 0 0 0 0 0 0 0 0 0 0 7 0 7.9 6.8 4.7 10.8 11.2 11.8
   11.2 9.9 0 10.7 10.4 4.2 10.6    8.7 5.7 13.3 13.2 12 14 14.1 13.9
   13.1 10.7 7.1 13.7 14.6 15 15.1 15 13.5 10.3 2.6 5.9 0 6 0 8.4 2.4
   0.7 12.1 5.8 6.8 8.7 6 10 8.7 14.2 12.3 7.4 3.4 0.2 7.2 13.2 15.8
   13.9 9.5 11 15.3 4.1 2.1 6 12.7 10.4 15.4 15.1 11.4 8.5 8 1.5 1.5
   2.4 2.6 1.1 0.1 0 9.5 4.5 2.4 3.9 1.3 2.2 8.3 1.1 3.4 2.8 5.1 0.2
   6.4 0.5 3.4 5.2 5.4 0.1 0 1.5 0 0.5 7.9 9.8 4.4 1.6 3.8 2.1 0.6 1
   1.5 10.7 3.8 8.3 7.1 9.3 12.7 6.9 3.6 10.3 3.3 0.2 5.7 11.7 13.4
   7.8 5.2 9.5 5 4.2 5.4 2 7.3 8.5 9 4.7 13.1 10.5 0 7.5 8.6 4.3 8
   2.5 0 2.2 1.2 8.1 2.8 0 0.4 5.1 1.2 6.2 2.1 0.1 5.1 0.3 0 11.7 0
   0 10.4 6.5 11.1 11.3 8.5 1.2 8.8 5.6 10.6 10.3 8.1 3.7 9.4 2.2 0.2
   0 0 0 0 0 2.2 2.9 2.7 6.9 0 6 3.3 0 0 0 7.4 9.1 8.9 1.7 0 0 0 0 4.1
   0 0 0 0 0 0 0 0 0 0 0 0 0 0 0 0 0 0 0 0 0 0 0 0 0 0 0 0 0 0 0 0 0
   0 0 0 0 0 0 0 0 0 0 0 0 0 0 0 0 0 0 0 0 0 0 0 0 0 0 0 0 0 0 ]

; 2001:
let foragingHoursListBerlin2001
  [ 0 0 0 0 0 0 0 0 0 0 0 0 0 0 0 0 0 0 0 0 0 0 0 0 0 0 0 0 0 0 0 0 0
   0 0 0 0 0 0 0 0 0 0 0 0 0 0 0 0 0 0 0 0 0 0 0 0 0 0 0 0 0 0 0 0 0
   0 0 0 0 0 0 0 0 0 0 0 0 0 0 0 0 0 0 0 0 0 0 0 0 0 2.3 10.3 6.2 5.5
   0 0 0 0 0 0 0 0 0 0 0 0 0 0 0 0 0 0 0 13 8.1 3.9 6.6 0 3 10.9 13
   13.2 13.6 4.9 0 0 0 9 14.2 14.2 14.7 13.7 12.2 12.6 2.1 8.3 2.9 5.3
   10.1 13.1 8.3 7.5 15.3 15.1 14.9 11.6 6.5 0 6.2 3.5 1 2 0 0 0.7 1.2
   3.1 3.1 1.4 8.9 0 6.9 0 11.3 4.6 6.8 4 8.5 3.2 5.7 14.3 3.3 3.3 2.5
   6 13.6 13.3 14.3 1.7 10.6 12.8 5.6 0.9 12.6 12.4 11.2 13.1 6.6 0.4
   0 5.5 5.4 11.1 6.5 2.5 3 0 0.6 8.5 11.9 11.2 5.9 11.1 7.9 11 10.4
   10.9 14.9 14.5 6.3 12.2 2.7 5.8 12.6 3.9 2.8 5.2 6.5 5.3 5.9 8.5 7.3
   7.4 1.1 0 5.6 13.3 12.8 6.2 0 2.9 6.6 0 9.3 11.8 8.3 10.3 11 3.8 4
   4.3 10.9 2.9 3.9 2.5 0.3 1.2 8.1 2.9 1.6 6.2 0 0.2 0 2.1 0.2 1.5 4.2
   3.8 3.5 0 9.9 0.5 2.6 1.1 9 0 0 0 0 0.8 4.3 0 0 0 2.2 4.5 3.8 9.5 1.1
   7.9 3.9 7.6 0 7.7 7.5 6.3 1.2 5.5 0 0 1.9 6.9 0 0 0 0 0 5.7 0 0 0 3.1
```

```
   2.2 0 0 0 0 0 0 0 0 0 0 0 0 0 0 0 0 0 0 0 0 0 0 0 0 0 0 0 0 0 0 0 0 0
   0 0 0 0 0 0 0 0 0 0 0 0 0 0 0 0 0 0 0 0 0 0 0 0 0 0 0 0 ]

; 2002:
let foragingHoursListBerlin2002
[ 0 0 0 0 0 0 0 0 0 0 0 0 0 0 0 0 0 0 0 0 0 0 0 0 0 0 0 0 0 0 0
  0 5.9 8.3 0 0 0 0 0 0 0 0 0 0 0 0 0 0 0 0 0 0 0 0 0 0 0 0 0 0 0 0 0 0 0 0 0 0 0 9.7
  0 0 0 0 0 0 7.2 0 0 0 0 0 0 0 0 0 0 11.2 9.1 2.8 11.2 11 0 0 0 0 0 0 0 0
  0 0 0 0 0 0 0 0 5 0 6.7 13.3 3.3 0 0.2 3.2 0 0 0 0 2.7 1 5.8 0 0 4.5 0 8.1
  12.7 11.7 5.2 5.6 7.9 6.7 4.3 10.4 13.7 14.7 0 8.6 10.9 12.9 7.7 2.4 1.4 0
  6.1 0 6.7 11.3 6.1 10.3 13.3 10.4 8.9 7.7 3.9 0 0 0.4 1.7 4.6 1.3 0.2 3
  4.8 6.2 11.1 14.4 6.4 6 4.3 9.9 6.3 9 10.3 10.1 7.4 8.3 5 1.4 0 2 1.9 0.3
  12.2 5.7 4.5 12.9 14.5 11.5 8.2 6.9 7.8 0 1.4 6.4 0.9 0.6 0 2.9 11.7 0.9
  1.6 2 2.9 0.4 8.6 14.3 11.3 11.5 7.1 7.6 0.7 13.4 8.8 0.1 7.5 4.3 2.9 3.7
  4.7 9.1 0 0 1.2 10.4 6.1 6.3 12.2 12.3 12.9 11.8 9.2 10.7 9 9.3 10.6 10.8
  10.5 8.5 8.6 6.7 7.8 11.8 10.4 10.6 6.7 10.6 4.8 10.4 10.9 9 7.2 12.1 10.2
  3.7 8.8 1.5 1.9 3.3 4.3 0.3 2.6 0 0 0 9.4 0 0 0 0.7 6.6 9.3 8.9 6.2 4.3 0
  0 0 0 0 0 0 0 0 0 0 1.2 0 0 0 0 0 0 0.9 0 0 0 0 0 0 0 0 0 0 0 0 0 0 0 0 0
  0 0 0 0 0 0 0 0 0 0 0 0 0 0 0 0 0 0 0 0 0 0 0 0 0 0 0 0 0 0 0 0 0 0 0 0 0
  0 0 0 0 0 0 0 0 0 0 0 0 0 0 0  ]

; 2003:
let foragingHoursListBerlin2003
[ 0 0 0 0 0 0 0 0 0 0 0 0 0 0 0 0 0 0 0 0 0 0 0 0 0 0 0 0 0 0 0 0 0 0 0 0 0
  0 0 0 0 0 0 0 0 0 0 0 0 0 0 0 0 0 0 0 0 0 0 0 0 0 0 0 0 0 0 0 0 0 0 0 0 0
  0 0 0 0 0 0 0 0 11.5 7.9 0 1.8 0 9.6 8.1 0 9.7 0 0 0 0 0 0 0 0 0 0 0 11.7
  12.4 12.4 12.5 12.7 0 0 11.8 11.7 12.8 12.4 8.6 8.6 0.1 7.3 4.7 2.9 4.5 7.3
  9.5 3.1 13.5 12.4 7.7 9.4 11.6 0.5 4.9 10.6 4.1 3.1 4.6 0 5.3 6.7 7.3 1.7
  5.5 5.9 8.1 1.1 13.1 14.3 5.6 10.3 9.9 15.4 15.4 7.8 14.3 14.4 12.5 13.6 10.9
  11.4 13.6 13.2 11.2 13.6 9.3 12.4 12.5 8.8 8.9 10.3 13.3 3.6 1.8 5.3 2.8 10.8
  5.7 10.9 2.7 3.8 13.9 15.2 5.2 11.6 2.3 6.1 8.1 1.3 0.4 0.1 3.6 4.5 3.1 6.2
  13.4 4.2 6.4 15.7 13.3 13.2 4 6.5 13.4 13.3 8.5 12.6 8.9 6.6 4.2 2.2 7.6 5
```

```
  7.5 12.6 4.6 10.4 5 8.1 12.8 12.8 12.1 13.9 13.8 13.9 14.2 14.4 10.5 13 4.6
  9.9 9.4 13.3 6 3.6 10.1 9.3 9.4 4.3 6.8 11.9 7.2 2.6 2.7 2.3 4.6 7.8 3.8 10.8
  2.7 0.8 11.7 11.2 5.7 9.7 2 3.5 0 1.3 3.5 6.1 10.8 8.2 6.9 10.7 11.4 11.3 11.6
  11.1 2.8 9.6 11.4 11.3 3.1 6.5 2.4 0 9.6 1.7 2.4 3.1 0 0 0 0 0 0 0 0 0 0 0
  0 0 0 0 0 0 0 0 0 0 0 0 0 0 0 0 0 0 0 0 0 0 0 0 0 0 0 0 0 0 0 0 0 0 0 0 0 0
  0 0 0 0 0 0 0 0 0 0 0 0 0 0 0 0 0 0 0 0 0 0 0 0 0 0 0 0 0 0 0 0 0 0 0 0 0 0 0 ]

; 2004:
let foragingHoursListBerlin2004
[ 0 0 0 0 0 0 0 0 0 0 0 0 0 0 0 0 0 0 0 0 0 0 0 0 0 0 0 0 0 0 0 0 0 0 0 0 0.1 0
  0 0 0 0 0 0 0 0 0 0 0 0 0 0 0 0 0 0 0 0 0 0 0 0 0 0 0 0 0 0 0 0 0 0 0 0 0 0
  0 9.9 7 1.3 0 0 0 0 0 0 0 0 0 0 0 12 10.6 0 6.5 2.5 0 0 0 0 0 0 0 0 0 13.2 13.1
  12.5 11 8.4 0.5 4.5 3.2 10.4 0 0 10.2 0 2 13.9 11.6 12.5 7.2 0 3.9 5.2 8.2 3.9
  2.3 0 0 4.4 4.7 0 0 6.6 0 9.6 0.4 8 8.4 8.1 0 0 0 0 1.6 0 0 11.6 15.2 14.8 8.7
  7.9 0 12.8 4.2 1.1 12.1 8.2 9.4 2.9 4.6 4 9.1 6.2 6.6 5.5 9.6 1 2.6 4.9 11.7
  11.6 7.7 4.9 5.2 5.4 6.3 0.2 8.6 8.1 4.5 5.8 9.3 7 7.5 6 11.4 13.7 4 3.6 3.9 9.6
  1 0.8 4.2 2.5 1.1 7.5 10.4 7 9.6 5 3.3 10.3 6.5 6.4 4.1 6.7 11.2 14.8 14.4 11.5
  9.7 8.3 8.5 12.2 11.9 13.9 12.4 12.6 12.9 13.7 7.3 11.5 4.9 5.2 12 7.5 5.1 6.3
  6.2 4.2 5.8 10.1 7.1 2.7 2.9 3 1.9 2.1 3.2 0.7 3.8 6.7 12.2 12.4 12.4 12.8 12.4
  10.7 11.6 12.6 12.5 4.5 5.1 4 5.2 7.8 8.1 11.6 11.7 4.7 2.4 1.5 3.2 0 0 3.9 0
  0.2 0.8 1 3.5 0.8 3.7 8.7 5.3 9.5 1.9 8.1 0 0 0 0 0 0 0 0 0 0 0 0 0 6.5 9.3 1.3
  5.4 3.7 0 0 0 0 0 0 0 0 0 0 0 0 0 0 0 0 0 0 0 0 0 0 0 0 0 0 0 0 0 0 0 0 0 0 0 0
  0 0 0 0 0 0 0 0 0 0 0 0 0 0 0 0 0 0 0 0 0 0 0 0 0 0 0 0 0 0 0 0 ]

; 2005:
let foragingHoursListBerlin2005
[ 0 0 0 0 0 0 0 0 0 0 0 0 0 0 0 0 0 0 0 0 0 0 0 0 0 0 0 0 0 0 0 0 0 0 0 0 0 0 0
  0 0 0 0 0 0 0 0 0 0 0 0 0 0 0 0 0 0 0 0 0 0 0 0 0 0 0 0 0 0 0 0 0 0 0 0 0.8 0.4
  0 0 0 0 0 0 4.7 7 0 0 0 0 0 0 0 12.3 10.4 11.7 0 9.4 0 0 0 0 0 6.3 2.1 6.7 7 10.2
  10 11.4 10.3 0 0 0 0 0 4.4 11.7 0 8.4 8.5 6.6 11.7 9.7 5 2.5 7.1 2.3 0 0 0 0 0
  0 11.8 1.6 0 8.4 0 0 12.7 11 5.7 4.7 0.4 5.4 9.6 12.7 13.9 15 14.2 0 4.3 0 2.8
  7.9 6.7 2.5 0 0 9.5 6.6 1.2 0 0 11.7 10.2 7.9 11.5 0.4 14.1 11.1 16 11.9 7.2 15.7
```

```
  9.8 8.7 14.8 15.7 15 13.8 10.9 0.1 3.2 9.2 12 0 1.1 2.1 0.1 3 14.3 14.8 14.9 13.7
  12 11 9.1 7.3 6.4 4.7 4.3 0 3 0.2 4.6 4 2.1 6.8 7.9 6.8 6.9 9.4 8.5 10.1 0 6.4
  5.6 3.9 5.1 11.1 0.5 0 1.3 8.4 0.6 1.2 4 10.9 6.6 13.7 12.4 8.4 11.5 11.1 0 6.5
  0.2 5.6 11.3 10 12.8 12 12.8 12.3 8.4 0.9 12.4 12.4 12.5 11.9 11.7 11.7 7.4 0 0.2
  6.6 6.9 7 0 8.1 11.7 6.8 5 0.7 11.3 11.2 10.3 10.5 3.6 7.4 0.8 0 3.4 1.7 0 0 5.4
  9.5 10 9.4 8.9 9.2 7.5 9.8 9.7 9.2 9.6 8.6 0 0 0 0 5.7 0 0.2 2.2 0 0 3.3 7.7 8.9
  8.6 8.2 0 0 0 2.7 0 0 0 0 0 0 0 0 0 0 0 0 0 0 0 0 0 0 0 0 0 0 0 0 0 0 0 0 0 0 0
  0 0 0 0 0 0 0 0 0 0 0 0 0 0 0 0 0 0 0 0 0 0 0 0 0 0 0 ]

; 2006:
let foragingHoursListBerlin2006
[ 0 0 0 0 0 0 0 0 0 0 0 0 0 0 0 0 0 0 0 0 0 0 0 0 0 0 0 0 0 0 0 0 0 0 0 0 0 0 0 0
  0 0 0 0 0 0 0 0 0 0 0 0 0 0 0 0 0 0 0 0 0 0 0 0 0 0 0 0 0 0 0 0 0 0 0 0 0 0 0 0
  0 0 0 0 1.8 1.5 5 0 0 0 3.8 8.5 0 0 0 0 0 8.2 0 0 0 0 0 0 0 0.1 0 0 6.5 9.6 4.3
  0 3.7 0 13.1 4.7 0 0 0 0 10.5 5.6 13.4 12.5 11.9 11 12.2 10.6 14.2 14.7 14.1 12.6
  6.8 4.6 10.5 8.6 1.4 0.3 3.5 6.1 1.5 7.7 5.8 9.9 0 0 1.6 6.6 0 0 2.4 0 11.5 4.4 0
  0 4.8 9 11.5 11.5 15.6 15.8 15.8 15.7 15.2 7.5 5.6 1.2 9.1 9.8 9 7.7 6.4 9.8 12.4
  13 9.7 12.3 10.4 10.2 0.7 14.2 15.8 16 16 15.7 12.9 10.6 2.5 12.3 11.7 10.8 13.3
  8.5 10.3 11.5 13.4 15.7 15.7 15.5 13.9 12 14.1 6 14 12.9 14.8 13.6 5 5 12.9 6 9.3
  8.5 6.4 3.5 0.6 0.8 9.3 4.6 5.3 2 3.9 8.4 0 9.8 2.2 6.9 8.2 3.7 11.2 7.7 4.9 7 0.9
  9.6 3.5 2.3 4.2 6.7 1.2 0.2 4.2 0.2 7.7 0 5.1 9.1 3.7 8.5 6.4 5.3 11.9 12.4 11.5
  12.1 12 11.4 6.4 4.7 9.2 1 8.9 11.3 11.5 11.4 11.3 11.1 9.5 0.1 3 10.2 7.8 3.9 1.3
  0.4 0.2 2.9 0.9 1.4 4.2 9.8 9.1 6.3 8.2 0 0 0 6.7 9.9 7.9 4.8 0 6 5.3 3.2 2.7 4.4
  6.3 7.1 0 1.1 0 0 0 0 0 0 0 0 0 0 0 0 0 0 0 0 4.7 7.7 1.3 0 0 0 0 0 0 0 0 0 0 0 0
  0 0 0 0 0 1 0 0 0 0 0 0 0 0 0 0 0 0 0 0 0 0 0 0 0 0 0 0 0 0 0 0 ]

; ROTHAMSTED WEATHER DATA 2009:
;TH: 15C:
let foragingHoursListRothamsted2009 [ 0  0  0  0  0  0  0  0  0  0  0  0  0  0  0  0  0  0  0  0  0  0  0  0  0  0  0  0  0  0  0  0  0  0  0  0  0  0  0  0  0  0  0
0  0  0  0  0  0  0  0  0  0  0  0  0  0  0  0  0  0  0  0  0  0  0  0  0  0  0  0  0  0  0  10.4  0  0  0  0  0  0  0  0  0  0  0  0  0  0  0  7.8  0  0  8.9  0  5.4  0  0  0
0  0  0  4.1  6  5.9  0  0  0  0  10.1  12.3  11  9.3  10.5  0  11.5  0  0  11.2  4.5  8  10.3  0  0  5.2  7.5  3.2  0  9.4  10.3  0  11.6  0  0.7  0  0  0  6.9  5.4
8.2  8.7  8.4  12.5  15  7.5  7.5  0.7  6.7  13  15  14.2  14.3  14.9  3.4  11.7  0  0  4.3  2.5  0  0.9  6.5  11.8  5.4  13  5.4  9.4  4.7  6  9.7  2.7  9  5  10.6
```

```
13.9 8 2.7 4.7 4.3 10.8 11.7 12.7 12.3 6.2 11.8 9 6.8 4.7 3.7 5.2 9.7 2.2 7.4 7.4 8.7 6.1 3.6 1.9 5.3 3.8 7.8 0.2 7.1 6.1 6.5 11.4 1.8
5.1 6.8 1.6 8.7 8.6 0.9 8.5 5.4 0 5.9 3.2 2.7 9.5 4.8 2.7 8.5 1.8 6.2 3.2 2.6 10.4 7.5 7.5 12.3 5.4 8.4 8.1 11.4 7.3 5.8 2.3 7.4 7.4
8.7 3.8 5.7 7.3 0.4 5.2 7.5 6.1 4.3 0.5 6.7 5.7 7 4.8 9.8 0.8 3.6 0 4.6 1.6 7.7 3.4 4.4 4.9 3.3 1.8 9.7 9.9 8 9.3 0.9 5.2 0.3 5.6 5.5
0.8 4.9 0.1 0.1 0 0 0 4 3.5 0 0 0 0 0 0 0 0 0 0 6.2 0.5 4.2 0 1.3 0.6 1.8 0 2.5 0.5 0 0 0 0 0 0 0 0 0 0 0 0 0 0 0 0 0 0 0
0.8 0 0 0 0 0 0 0 0 0 0 0 0 0 0 0 0 0 0 0 0 0 0 0 0 0 0 0 0 0 0 0 0 0 0 0 0 0 0 0 0 0 ]

  ; ROTHAMSTED WEATHER DATA 2010:
  ; TH: 15C:
   let foragingHoursListRothamsted2010 [ 0 0 0 0 0 0 0 0 0 0 0 0 0 0 0 0 0 0 0 0 0 0 0 0 0 0 0 0 0 0 0 0 0 0 0 0 0 0 0 0 0 0
0 0 0 0 0 0 0 0 0 0 0 0 0 0 0 0 0 0 0 0 0 0 0 0 0 0 0 0 0 0 0 0 0 0 0 5.3 0 0 0 0 0 5.8 0 0 0 0 0 0 0 0 0 0 0 0 9.3 0 11.4
9.1 10.6 0 0 0 0 0 0 11.8 11.4 0 0 0 0 13.1 11.2 2.4 4.4 10.4 8.1 1.7 0 3 0 0 0 0 0 0 0 0 0 0 0 0 0 8.3 0 6.4 7 8.1 5.9 12.5
14.9 15 14.7 9 5 6.2 10.7 0 10.1 1.1 0 12.8 15.4 12.9 8.5 3.7 5.7 3.1 2.8 0.9 5 4.5 5 6.5 9 12.1 13.9 1.5 0 6.9 9.1 14.6 13 10.2 9
8.9 13.7 14 6.2 7.6 7.3 3.8 10.3 10.2 7.2 7.6 1.4 6.5 12.5 10.8 7.3 4.6 0 2.2 4.1 6.8 9.6 6.3 9.3 5.8 10.3 7.6 1.7 7 2.9 0.9 1.2 2 2
4 6 1.3 3.3 7.3 0.8 5.8 4.6 3 5.8 9.3 1.1 9.6 2.9 2.8 1.4 8 7.2 2.1 6.6 4.9 1.1 1.3 6.3 2.9 8 1.6 0 2.9 7.5 4.7 6 10.2 11.3 11.1 8.5
5.6 2.4 4 5 1.6 4.2 1 3 8.6 2.3 0 5 4.6 6.3 7.3 1.1 5.2 7.5 8.7 1.3 0 0 0 0 0.3 0.1 6.6 0 3.1 1.3 0.1 0.7 5.2 6 4.1 0 6.4 8.6 0 0 0
0 0 0 0 0 0 0 0 0 0 0 0 4.7 0 0 0 0 0 0 3.3 2.2 0 0 0 0 0 0 0 0 0 0 0 0 0 0 0 0 0 0 0 0 0 0 0 0 0 0 0 0 0 0 0 0 0 0 0
0 0 0 0 0 0 0 0 0 0 0 0 0 0 0 0 0 0 0 0 0 ]

  ; ROTHAMSTED WEATHER DATA 2011:
  ; TH: 15degC
  let foragingHoursListRothamsted2011 [ 0 0 0 0 0 0 0 0 0 0 0 0 0 0 0 0 0 0 0 0 0 0 0 0 0 0 0 0 0 0 0 0 0 0 0 0 0 0 0 0 0 0 0
0 0 0 0 0 0 0 0 0 0 0 5.4 1.7 0 0 0 0 0 0 0 0 0 0 0 0 3.2 0 4.9 0 0 3.9 0 0 0 0 0 3.1 1.1 10.1 10.5 10.2 3 7.5 6.5 4.6 0.2 4.8
3.3 3.9 6.1 6.2 0 11.5 10.2 12.5 12 11.1 8.5 10.2 0 0.2 5 3.6 6.6 6.7 11.3 8.5 7.8 12.6 12.7 10.1 12.8 4.6 10.9 6.8 5.3 12.9 12.2 13
13.2 13.6 6.6 11.8 3.2 6.8 10.8 11 2.1 8 7.2 8.7 5.1 3.6 2.6 1.3 9.8 8.6 12.3 9.4 4.5 11.9 13 3.8 4.1 2.9 4.2 1.5 10.8 9.7 9.8 13.5
10.7 2.6 0.9 9.1 8.5 4.5 6.6 9.4 2 6.6 11.8 3.6 5.2 1.3 6.7 9.8 7.1 7.1 5.2 7 7.3 6.7 12 8.9 1.6 11.1 8.2 8.5 8.3 4.8 4.6 8.7 6.7 4.4
3.3 5.6 4.2 8.3 1 2.1 8.1 9.5 3.2 3.1 1.1 3.6 1.4 1.3 8 6.6 12.7 9.2 1.7 2.3 6.9 2.2 11.3 8.7 7.5 6.9 8.7 0.3 3.5 1.8 4.9 7.5 10.1 7.1
2.5 2.8 2 6.4 7.2 3.5 4.1 0.1 9.6 5.4 6.6 8.8 0 4.2 3.2 1.2 5.6 4.4 4.6 0 1 6.2 8.4 5 2 5.8 0 1.2 1.5 1.5 2.3 5.2 6.9 7.5 8.6 7 4.9
5.9 6 6.6 0.2 2.6 5.3 8.4 6.4 6.9 2.7 6.3 9.5 9.7 9.8 9.2 9.7 6.3 4.1 1.3 7 3.3 0.8 2.3 4.7 1.6 2.3 0.1 8.3 9.3 4.5 2.3 8.2 6 0 3.3 9.1
6.4 4.8 2.8 5.8 0 8.3 4.1 0 0 4.1 3.5 0 1.4 0.5 0 0 0 1.1 1.2 0 0.7 5.9 0 0 0 2.2 3 0 0 0 0 0 2 0 2.8 5.6 0 0.3 0 0 0 0 0 0 0 0
0 0 0 0 0 0 0 0 0 0 0 0 0 0 0 0 0 0 0.4 0 0 0 0 0 ]

  if Weather = "Rothamsted (2009-2011)"
```

```
[
  let inputYear 2011 + round ((ceiling (ticks / 365)) mod 3)
  if inputYear > 2011
  [
    while [inputYear > 2011] [ set inputYear inputYear - 3]
  ] ; after 3 years, 1st dataset is used again etc.

 ;if day = 1 [ type "Rothamsted weather data, year: " print inputYear ]
  if inputYear = 2009 [ set foragingHoursList foragingHoursListRothamsted2009 ]
  if inputYear = 2010 [ set foragingHoursList foragingHoursListRothamsted2010 ]
  if inputYear = 2011 [ set foragingHoursList foragingHoursListRothamsted2011 ]
]

if Weather = "Rothamsted (2009)" [ set foragingHoursList foragingHoursListRothamsted2009 ]
if Weather = "Rothamsted (2010)" [ set foragingHoursList foragingHoursListRothamsted2010 ]
if Weather = "Rothamsted (2011)" [ set foragingHoursList foragingHoursListRothamsted2011 ]

if Weather = "Berlin (2000-2006)"
[
  let inputYear 2006 + round ((ceiling (ticks / 365)) mod 7)
  if inputYear > 2006 [ while [inputYear > 2006] [ set inputYear inputYear - 7] ]
    ; after 7 years, 1st dataset is used again etc.
  if inputYear = 2000 [ set foragingHoursList foragingHoursListBerlin2000 ]
  if inputYear = 2001 [ set foragingHoursList foragingHoursListBerlin2001 ]
  if inputYear = 2002 [ set foragingHoursList foragingHoursListBerlin2002 ]
  if inputYear = 2003 [ set foragingHoursList foragingHoursListBerlin2003 ]
  if inputYear = 2004 [ set foragingHoursList foragingHoursListBerlin2004 ]
  if inputYear = 2005 [ set foragingHoursList foragingHoursListBerlin2005 ]
  if inputYear = 2006 [ set foragingHoursList foragingHoursListBerlin2006 ]
]

if Weather = "Berlin (2000)" [ set foragingHoursList foragingHoursListBerlin2000 ]
```

```
if Weather != "HoPoMo_Season"
  and Weather != "HoPoMo_Season_Random"
  and Weather != "Constant"
[
  set foragingPeriod_s (item (day - 1) foragingHoursList) * 3600
] ; [s] hours sunshine on that day, in seconds

if Weather = "HoPoMo_Season" or Weather = "HoPoMo_Season_Random"
[
  set foragingPeriod_s 12 * 3600 * (1 - Season_HoPoMoREP day [ 385 25 36 155 60 ])
  if foragingPeriod_s < 3600 [ set foragingPeriod_s 0 ]
]   ; bell shape curve of foraging period, 12 * 3600 = 12 hrs max.

if Weather = "HoPoMo_Season_Random"
[
  if random-float 1 < 0.15 [ set foragingPeriod_s random-float (4 * 3600)]
]

if Weather = "Constant" [ set foragingPeriod_s 8 * 3600 ]

ask signs with [ shape = "sun"]
[
  ifelse foragingPeriod_s > 0
    [ show-turtle set label precision (foragingPeriod_s / 3600) 1 ]
    [ hide-turtle set label " " ]
]   ; "sun" sign is shown, whenever there is an opportunity to forage

ask signs with [ shape = "cloud"]
[
  ifelse foragingPeriod_s < (4 * 3600)
    [ show-turtle ]
```

```
    [ hide-turtle ]
  ]  ; "cloud" sign is shown, whenever there is less than 4 hrs of foraging possible

  if foragingPeriod_s = -1
  [
    set BugAlarm true
    show "BugAlarm in Foraging_PeriodREP! Weather not defined!"
  ]

  report foragingPeriod_s
end

; *****************************************************************************************************************


to-report Foraging_ProbabilityREP
  ; calculates the probability that a forager start spontaneously to forage,
  ; called by Start_IBM_Proc once a day
  let foragingProbability 0.01  ; 0.01
    ; default foraging probability per "round" (round: ca. 13 min)
    ; 0.01 comparable to Dornhaus et al 2006: 0.00033/36s
  let highForProb 0.05 ; 0.02
  let emergencyProb 0.2
    ; foraging prob. is increased if pollen is needed:
  if (PollenStore_g / IdealPollenStore_g) < 0.2
  [
    set foragingProbability highForProb
  ]

  if HoneyEnergyStore / DecentHoneyEnergyStore < 0.5
  [
    set foragingProbability highForProb
```

```
]
 ; foraging prob. is increased if pollen is needed:
if HoneyEnergyStore / DecentHoneyEnergyStore < 0.2
[
  set foragingProbability emergencyProb
]

if (PollenStore_g / IdealPollenStore_g) > 0.5 and
   HoneyEnergyStore / DecentHoneyEnergyStore > 1
   [
     set foragingProbability 0
   ] ; no foraging if plenty of honey and pollen is present

let i 1
 while [ i <= N_GENERIC_PLOTS ]
 [
   let plotname (word "Generic plot " i)
     ; e.g. "Generic plot 1"
   set-current-plot plotname
   if (i = 1 and GenericPlot1 = "foraging probability")
   or (i = 2 and GenericPlot2 = "foraging probability")
   or (i = 3 and GenericPlot3 = "foraging probability")
   or (i = 4 and GenericPlot4 = "foraging probability")
   or (i = 5 and GenericPlot5 = "foraging probability")
   or (i = 6 and GenericPlot6 = "foraging probability")
   or (i = 7 and GenericPlot7 = "foraging probability")
   or (i = 8 and GenericPlot8 = "foraging probability")
   [
     create-temporary-plot-pen "ForProb"
     set-plot-pen-mode 0 ; 0: lines
     plotxy ticks  (foragingProbability)
    ]
```

```
    set i i + 1
  ]

  ask Signs with [shape = "exclamation"]
    [ ; if the foraging prob. is set to 0, an exclamation mark is shown
      ; on the interface (beside the weather sign)
      ifelse foragingProbability > 0
        [ hide-turtle ]
        [ show-turtle ]
    ]

 report foragingProbability
end

; ************************************************************************************************************

to Foraging_start-stopProc
  ; decision for pollen or nectar foraging; active foragers may quit foraging;
  ; foragers might spontaneously start or continue foraging (either exploiting known
  ; patch or search new patch)

  let FORAGE_AUTOCORR 0 ;
  ; autocorrelation of chosen forage (i.e. probability to not-reconsider chosen forage
  ; type: 1: always collect the same forage type (i.e. nectar!) if 0: no effect)

  ask foragerSquadrons with [ activity != "recForaging" ]
   ; this does not apply to bees, that followed a dance in the last foraging round
   ; and hence have already made their decision for nectar or pollen foraging
  [
    if random-float 1 > FORAGE_AUTOCORR
    ; if smaller, the bee sticks to her current food type
```

```
 [
  ifelse random-float 1 < ProbPollenCollection
   [
    set pollenForager true     ; IF -> pollen forager
    set activityList lput "PF" activityList
   ]
   [
    set pollenForager false    ; ELSE -> nectar forager
    set shape "bee_mb_1" ; ] ] ]
    set activityList lput "NF" activityList
   ]
 ]
]

ask foragerSquadrons with
  [ activity != "resting"
   and activity != "recForaging"
   and activity != "lazy" ]
  ; i.e. ask actively foraging bees
[
 if random-float 1 < FORAGING_STOP_PROB
 ; active foragers, that weren't recruited in the foraging round before, may abandon foraging
  [
   set activity "resting"
   set activityList lput "AfR" activityList
  ]
]

; recording of the activities & forage type in the activityList
ask foragerSquadrons with
  [ activity = "searching" ]
  [
```

```
    if pollenForager = true
    [
      set activityList lput "Sp" activityList
    ]
    if pollenForager = false
    [
      set activityList lput "Sn" activityList
    ]
                                                  ]
ask foragerSquadrons with [ activity = "resting" ]
[
  set activityList lput "R" activityList
]

ask foragerSquadrons with [ activity = "lazy" ]
[
  set activityList lput "L" activityList
]

ask foragerSquadrons with
  [ knownNectarPatch >= 0
    and pollenForager = false
  ]
  ; ask experienced NECTAR foragers if they abandon their nectar patch
[
  if random-float 1 < 1 / [ EEF ] of flowerPatch knownNectarPatch
    and random-float 1 < (HoneyEnergyStore / DecentHoneyEnergyStore)
      ; chance to abandon depends on 1/EEF and is reduced if colony needs nectar
  [
    set knownNectarPatch -1   ; forager doesn't know a nectar patch anymore
    ifelse (activity != "resting" and activity != "lazy")
      [
```

```
      set activity "searching"
      set activityList lput "AnSn" activityList
    ] ; active foragers that abandoned their patch have to search a new one
    [
      set activityList lput "An" activityList
    ]  ; resting foragers that abandoned their patch still rest
  ]
]

ask foragerSquadrons with
  [ knownPollenPatch >= 0
    and pollenForager = true ]
  ; ask experienced POLLEN foragers if they abandon their pollen patch
[
  if random-float 1 < 1 - (1 -
     ABANDON_POLLEN_PATCH_PROB_PER_S) ^ [ tripDurationPollen ] of flowerPatch knownPollenPatch
  [
    set knownPollenPatch -1   ; forager doesn't know a pollen patch anymore
    ifelse ( activity != "resting"
      and activity != "lazy")
    [
      set activity "searching"
      set activityList lput "ApSp" activityList
    ] ; active foragers that abandoned their patch have to search a new one
    [
      set activityList lput "Ap" activityList
    ]  ; resting foragers that abandoned their patch still rest
  ]
]

ask foragerSquadrons with [ activity = "resting" ]
[
```

```
  if random-float 1 < ForagingSpontaneousProb
   ;  resting foragers may start foraging spontaneously..
  [
    if pollenForager = false
     ; ask (resting) nectar foragers to become active
    [
      ifelse knownNectarPatch >= 0
        [
          set activity "expForaging"
          set activityList lput "Xn" activityList
        ] ; IF they already know a NECTAR patch, they become experienced nectar foragers
        [
          set activity "searching"
          set activityList lput "Sn" activityList
        ] ; ELSE: they become scouts and search a new one
    ]

    if pollenForager = true   ; ask (resting) pollen foragers to become active
    [
      ifelse knownPollenPatch >= 0
        [
          set activity "expForaging"
          set activityList lput "Xp1" activityList
        ] ; IF they already know a POLLEN patch, they become experienced pollen foragers
        [
          set activity "searching"
          set activityList lput "Sp" activityList
        ] ; ELSE: they become scouts and search a new one
    ]
  ] ; "if random-float 1 < ForagingSpontaneousProb"
]
ask foragerSquadrons  ; if bees are "exhausted" they cease foraging on that day:
```

```
  [
    if km_today >= MAX_km_PER_DAY
    [
      set activity "resting"
    ]
  ]

end

; ****************************************************************************************************************

to Foraging_searchingProc
  ; called by: ForagingRoundProc, determines if a patch (and which one) is
  ; found by a searching forager

  let patchCounter 0
  let probSum 0  ; necessary to decide, which flower patch is found
  let chosenPatch -1  ; -1: i.e. no patch chosen yet
  let cumulative_NON-detectionProb 1
  let nowAvailablePatchesList [ ]

  ask flowerPatches with
     [ quantityMyl >= CROPVOLUME * SQUADRON_SIZE
       or amountPollen_g >= POLLENLOAD * SQUADRON_SIZE ]
     ; only patches with enough nectar OR pollen left are considered
  [
    set probSum probSum + detectionProbability ; sums up the detection probabilities of patches, to decide later, which patch was actually found
    set cumulative_NON-detectionProb
      cumulative_NON-detectionProb * (1 - detectionProbability)
        ; Probability to find any patch is: 1 - Probability, to find no patch at all
    set nowAvailablePatchesList fput who nowAvailablePatchesList
```

```
]

set TotalFPdetectionProb (1 - cumulative_NON-detectionProb)
  ; Probability to find ANY (not empty!) flower patch during one search trip

ask foragerSquadrons with [ activity = "searching" ]
[
  set SearchingFlightsToday SearchingFlightsToday + SQUADRON_SIZE
    ; counts the numer of search flights on current day
  ifelse random-float 1 < TotalFPdetectionProb
    ; if any (not empty!!) flower patch found by the forager:
    [
      let p random-float probSum   ; to decide which flower patch is found
      set patchCounter 0
      set chosenPatch -1

      foreach nowAvailablePatchesList
      [
        ask flowerPatch ?   ;  "?" item of the list
        [  ; the patch is randomly chosen, according to its detection probability:
          set patchCounter patchCounter + detectionProbability
          if (patchCounter >= p) and (chosenPatch = -1) [ set chosenPatch who ]
        ]
      ]

      ifelse pollenForager = false
        [ set knownNectarPatch chosenPatch ]
          ; IF nectar forager: detected patch is memorised as nectar patch
        [ set knownPollenPatch chosenPatch ]
          ; ELSE pollen forager: detected patch is memorised as pollen patch

      if (knownNectarPatch < 0 and knownPollenPatch < 0)
```

```
[
  user-message "BUG: negative flower patches!"
  set BugAlarm true
]

ifelse ( pollenForager = false
  and [ quantityMyl ] of flowerPatch chosenPatch >= (CROPVOLUME * SQUADRON_SIZE))
    ; collection of NECTAR - only if nectar is available at the chosen patch!
    ; this is necessary as the patch may offer only pollen
  [
    set activity "bringingNectar"  ; then the scout becomes a successful nectar forager
    set activityList lput "fN" activityList

    ask flowerPatch knownNectarPatch
    [
      set quantityMyl (quantityMyl - (CROPVOLUME * SQUADRON_SIZE))
        ; quantity of nectar in patch is reduced

      set nectarVisitsToday nectarVisitsToday + SQUADRON_SIZE
      set summedVisitors summedVisitors + SQUADRON_SIZE
    ] ; and numbers of visitors increased
  ]
  [ ; ELSE: found a patch but it doesn't offer nectar: feN: "found empty nectar patch"
    if pollenForager = false
    [
      set knownNectarPatch -1
      set activityList lput "feN" activityList
    ]
  ]

ifelse ( pollenForager = true
  and [ amountPollen_g ] of flowerPatch chosenPatch >= (POLLENLOAD * SQUADRON_SIZE))
```

```
        ; collection of POLLEN - only if pollen is available at the chosen patch!
      [
        set activity "bringingPollen"  ; then the scout becomes a successful pollen forager
        set activityList lput "fP" activityList

        ask flowerPatch knownPollenPatch
        [
          set amountPollen_g (amountPollen_g - (POLLENLOAD * SQUADRON_SIZE))
            ; quantity of nectar in patch is reduced
          set pollenVisitsToday pollenVisitsToday + SQUADRON_SIZE
          set summedVisitors summedVisitors + SQUADRON_SIZE
        ] ; and numbers of visitors increased
      ]
      [
        if pollenForager = true
        [
          set knownPollenPatch -1
          set activityList lput "feP" activityList
        ]
      ] ; ELSE: found patch does not offer pollen: feP: "found empty pollen patch"
    ] ; "ifelse random-float 1 < TotalFPdetectionProb"

    ; ELSE: no patch is found; uS = unsuccessful searching
    [
      set activityList lput "uS" activityList
    ]
] ; "ask foragerSquadrons with [ activity = "searching" ]"

ask foragerSquadrons with ; ask recruited NECTAR foragers:
   [ activity = "recForaging" ; forager is recruited
     and knownNectarPatch >= 0 ; it knows a patch where it is recruited to
     and pollenForager = false ] ; and it is looking for nectar
```

```
[ ; the flights of recruited bees are counted:
  set RecruitedFlightsToday RecruitedFlightsToday + SQUADRON_SIZE
  ; IF(1) recruited Forager finds the nectar patch:
  ifelse random-float 1 < FIND_DANCED_PATCH_PROB
    [ ; and IF (2) nectar is still there:
      ifelse [ quantityMyl ] of flowerPatch knownNectarPatch >= (CROPVOLUME * SQUADRON_SIZE)
        [  ; .. then the recruit becomes a successful nectar forager
          set activity "bringingNectar"
          ; which is recorded in its activityList:
          set activityList lput "frN" activityList
          ask flowerPatch knownNectarPatch
          [  ; the nectar in the patch is then reduced:
            set quantityMyl (quantityMyl - (CROPVOLUME * SQUADRON_SIZE))
            ; the visit is counted:
            set nectarVisitsToday nectarVisitsToday + SQUADRON_SIZE
            set summedVisitors summedVisitors + SQUADRON_SIZE
          ]
        ]
        [  ; ELSE(2): if patch has not enough nectar, recruit becomes a scout again
          set activity "searching"
          set activityList lput "eSn" activityList
          ; and the patch is forgotten:
          set knownNectarPatch -1
        ]
    ]
    [ ; ELSE(1): if the recruits does not find the patch, it starts searching
      set activity "searching"
      set activityList lput "mSn" activityList
      ; and forgets "known" nectar patch
      set knownNectarPatch -1
    ]
]
```

```
; also recruited POLLEN foragers are searching a patch:
ask foragerSquadrons with
   [ activity = "recForaging"
     and knownPollenPatch >= 0
     and pollenForager = true ]
[
  set RecruitedFlightsToday RecruitedFlightsToday + SQUADRON_SIZE
  ; they find their patch with the probability of FIND_DANCED_PATCH_PROB
  ifelse random-float 1 < FIND_DANCED_PATCH_PROB
    ; IF(1) recruited Forager finds the pollen patch...
    [
      ifelse [ amountPollen_g ] of flowerPatch knownPollenPatch >= (POLLENLOAD * SQUADRON_SIZE)
        ; ..and pollen is still there..
        [ set activity "bringingPollen"
          ; .. then the recruit becomes a successful pollen forager
          set activityList lput "frP" activityList
          ask flowerPatch knownPollenPatch
          [
            set amountPollen_g (amountPollen_g - (POLLENLOAD * SQUADRON_SIZE))
              ; ..pollen in the patch is reduced
            set pollenVisitsToday pollenVisitsToday + SQUADRON_SIZE
            set summedVisitors summedVisitors + SQUADRON_SIZE
          ]  ; ..and numbers of visitors increased
        ]
        [ ; ELSE(2): if patch has not enough pollen, recruit becomes a scout again
          set activity "searching"
          set activityList lput "eSp" activityList
          set knownPollenPatch -1
        ]
    ]
    [ ; ELSE(1): if she does not find the patch, she starts searching
```

```
        ; (but can't find another patch in this foraging round)
        set activity "searching"
        set activityList lput "mSp" activityList
        ; it forgets its "known" pollen patch:
        set knownPollenPatch -1
      ]
  ] ; "ask foragerSquadrons with [ activity = "recForaging"]"

end;

; ****************************************************************************************************************

to Foraging_collectNectarPollenProc
  ; successful foragers gather nectar/pollen (if still available) and decrease
  ; nectar/pollen in flower patch

   ; ask experienced NECTAR foragers:
  ask foragerSquadrons with
     [ activity = "expForaging"
       and knownNectarPatch >= 0
       and pollenForager = false ]
  [ ; does patch still have enough nectar?:
    ifelse [ quantityMyl ] of flowerPatch knownNectarPatch >= (CROPVOLUME * SQUADRON_SIZE)
      [ ; the forager will then be bringing nectar:
        set NectarFlightsToday NectarFlightsToday + SQUADRON_SIZE
        set activity "bringingNectar"
        ; this is recorded in its activityList:
        set activityList lput "N" activityList

        ask flowerPatch knownNectarPatch
        [ ; available nectar in the patch is reduced:
```

```
      set quantityMyl (quantityMyl - ( CROPVOLUME * SQUADRON_SIZE))
      ; the visits are counted:
      set nectarVisitsToday nectarVisitsToday + SQUADRON_SIZE
      ; and numbers of visitors increased:
      set summedVisitors summedVisitors + SQUADRON_SIZE
     ]
    ]
    [ ; ELSE: not enough nectar available at the patch
     ; the forager will then become a scout:
     set activity "searching"
     set activityList lput "eSn" activityList
     ; the bee forgets this empty nectar patch
     set knownNectarPatch -1
    ]
]

; ask experienced POLLEN foragers:
ask foragerSquadrons with
   [ activity = "expForaging"
    and knownPollenPatch >= 0
    and pollenForager = true ]
[ ; does patch still have enough pollen?
  ifelse [ amountPollen_g ] of flowerPatch knownPollenPatch >= (POLLENLOAD * SQUADRON_SIZE)
   [ ; IF patch has enough pollen:
    set PollenFlightsToday PollenFlightsToday + SQUADRON_SIZE
    ; the forager will then be bringing pollen:
    set activity "bringingPollen"
    set activityList lput "P" activityList

    ask flowerPatch knownPollenPatch
    [  ; available pollen in the patch is reduced:
      set amountPollen_g (amountPollen_g - (POLLENLOAD * SQUADRON_SIZE))
```

```
      set pollenVisitsToday pollenVisitsToday + SQUADRON_SIZE
       ; and numbers of visitors increased
      set summedVisitors summedVisitors + SQUADRON_SIZE ]
  ]
  [ ; ELSE: not enough pollen available at the patch
    ; the forager will then become a scout:
    set activity "searching"
    set activityList lput "eSp" activityList
    set knownPollenPatch -1
  ]
]

; experienced pollen foragers, who know a nectar patch but no pollen patch
; or experienced nectar foragers, who know a pollen patch but no nectar patch:
; this can happen if e.g. an exp. nectar foragers switches to pollen foraging
; these bees switch to "resting" and DO NOT LEAVE THE HIVE!
; hence, their mileometer or km_today doesn't change
; and they are not considered in the Foraging_MortalityProc
ask foragerSquadrons with
  [ ( activity = "expForaging"    ; experienced (but got its experience as pollen forager!)
      and pollenForager = false   ; has now switched to nectar foraging
      and knownNectarPatch = -1   ; but doesn't know a nectar patch
      )
      or
    ( activity = "expForaging"    ; experienced (but got its experience as nectar forager!)
      and pollenForager = true    ; has now switched to pollen foraging
      and knownPollenPatch = -1   ; but doesn't know a pollen patch
      )]
[
  set activity "resting"       ; switch to resting - i.e. they haven't left the hive in this foraging round
  set activityList lput "Rx" activityList
]
```

```
; ask successful NECTAR foragers:
ask foragerSquadrons with [ activity = "bringingNectar" ]
[ ; the energy content of their cropload is calculated, which depends on the nectar concentration:
  set cropEnergyLoad ([ nectarConcFlowerPatch ] of
     flowerPatch knownNectarPatch * CROPVOLUME * ENERGY_SUCROSE)  ; [kJ]

  ; the distance they have travelled today is increased..
  set km_today km_today + ([ flightCostsNectar ] of
     flowerPatch knownNectarPatch / (FLIGHTCOSTS_PER_m * 1000))

  ; and also their total travelled distance:
  set mileometer mileometer + ([ flightCostsNectar ] of
    flowerPatch knownNectarPatch / (FLIGHTCOSTS_PER_m * 1000))  ;

  ifelse readInfile = true
    [ ; if patch data are read in, then the color of the bee
      ; reflects the ID of the flower patch:
      ; set color knownNectarPatch
      let memoColor 0
      ask flowerPatch knownNectarPatch [ set memoColor color ]
      set color memoColor
    ]
    [ ; ELSE: if there are 2 patches, defined via GUI,
      ; then the color of the bee reflects the patch it is foraging at:
      if knownNectarPatch = -1 [ set color grey ]
      if knownNectarPatch = 0 [ set color red ]
      if knownNectarPatch > 0 [ set color green ]
    ]
]

; and similar for successful POLLEN foragers:
```

```
  ask foragerSquadrons with [ activity = "bringingPollen" ]
  [ ; the pollen load is the same for all patches!
    set collectedPollen POLLENLOAD ; [g]
    set shape "bee_mb_pollen"

    ; the distance they have travelled today is increased..
    set km_today km_today + ([ flightCostsPollen ] of
      flowerPatch knownPollenPatch / (FLIGHTCOSTS_PER_m * 1000))

    ; and also their total travelled distance:
    set mileometer mileometer + ([ flightCostsPollen ] of
      flowerPatch knownPollenPatch / (FLIGHTCOSTS_PER_m * 1000))  ;

    ifelse readInfile = true
      [ ; the color of the bee is set according to its flower patch:
        ; set color knownPollenPatch
        let memoColor 0
        ask flowerPatch knownPollenPatch [ set memoColor color ]
        set color memoColor
      ]
      [
        if knownPollenPatch = -1 [ set color grey ]
        if knownPollenPatch = 0 [ set color red ]
        if knownPollenPatch > 0 [ set color green ]
      ]
  ]
end;

; ***********************************************************************************************************************
```

```
to Foraging_flightCosts_flightTimeProc
 ; sums up travelled distance for unsuccessful scouts and honey consumption due to foraging, trip duration
 ; consumption is subtracted from honey store, not from the crop, as it is empty for unsuccessful scouts
 let energyConsumption 0

 ; flight distance for successful foragers is calculated in Foraging_collectNectarPollenProc!
 ; flight distance for unsuccessful scout is calculated here:
 ask foragerSquadrons with [ activity = "searching" ]
 [ ; the search length [m] of the foraging trip is added to today's km and the lifetime km (mileometer):
   set km_today km_today + ( SEARCH_LENGTH_M / 1000 )
   set mileometer mileometer + ( SEARCH_LENGTH_M / 1000 )  ; mileometer: [km]

   ; honey store in the colony is reduced to reflect the energy consumed during the trip:
   set HoneyEnergyStore HoneyEnergyStore - ( SEARCH_LENGTH_M * FLIGHTCOSTS_PER_m * SQUADRON_SIZE )
   set ColonyTripDurationSum ColonyTripDurationSum + (SEARCH_LENGTH_M / FLIGHT_VELOCITY )  ; sums up time of a search trip

   ; sums up # foragers doing a trip & unsuccessful foraging trips:
   set ColonyTripForagersSum ColonyTripForagersSum + 1
   set EmptyFlightsToday EmptyFlightsToday + SQUADRON_SIZE
 ]

 ; energy consumption for successful foragers:
 ask foragerSquadrons with
   [ activity = "bringingNectar"
     or activity = "bringingPollen" ]
 [
; nectar foragers
   if pollenForager = false
   [
     ask flowerPatch knownNectarPatch
     [ ; flightCostsNectar is a flowerPatch variable, reflecting distance and handling time
```

```
      set energyConsumption flightCostsNectar
       ; energy is used, according to the flight costs of the patch
      set ColonyTripDurationSum ColonyTripDurationSum + tripDuration + TIME_UNLOADING
    ]  ; adds duration of this nectar trip to the sum of all trips performed during this foraging round so far
   ]

; pollen foragers
   if pollenForager = true
    [
      ask flowerPatch knownPollenPatch
      [
        set energyConsumption flightCostsPollen
          ; energy is used, according to the flight costs of the patch
        set ColonyTripDurationSum ColonyTripDurationSum + tripDurationPollen + TIME_UNLOADING_POLLEN
      ] ; adds duration of this pollen trip to the sum of all trips performed during this foraging round so far
    ]

    ; colony's honey store is decreased:
   set HoneyEnergyStore HoneyEnergyStore - ( energyConsumption * SQUADRON_SIZE)
    ; sums up # foragers doing a trip:
   set ColonyTripForagersSum ColonyTripForagersSum + 1
  ]

end


; ******************************************************************************************************************


to Foraging_mortalityProc
  ; mortality of foragers during their foraging trip, counts # dying foragers and their lifespan
  let emptyTripDuration SEARCH_LENGTH_M / FLIGHT_VELOCITY  ; [s] = 10 min
```

```
ask foragerSquadrons with [ activity = "searching" ]
[ ; mortality risk of unsuccessful scouts depends on their time spent for searching
  ; mortality risk calculated as probability to NOT survive every single second of the foraging trip:
  if random-float 1 < 1 - ((1 - MORTALITY_FOR_PER_SEC) ^ emptyTripDuration)
  [ ; deaths are counted and the lifespans summed up to later calculate a mean lifespan:
    set DeathsAdultWorkers_t DeathsAdultWorkers_t + SQUADRON_SIZE
    set DeathsForagingToday DeathsForagingToday + SQUADRON_SIZE
    set SumLifeSpanAdultWorkers_t SumLifeSpanAdultWorkers_t + (age * SQUADRON_SIZE)
    die
  ]
]
; this is similar for NECTAR foragers, but here with a patch specific mortalityRisk
ask foragerSquadrons with [ activity = "bringingNectar" ]
[
  if random-float 1 < ([ mortalityRisk ] of flowerPatch knownNectarPatch)
  [
    set DeathsAdultWorkers_t DeathsAdultWorkers_t + SQUADRON_SIZE
    set DeathsForagingToday DeathsForagingToday + SQUADRON_SIZE
    set SumLifeSpanAdultWorkers_t SumLifeSpanAdultWorkers_t + (age * SQUADRON_SIZE)
    die
  ]
]
; and again for POLLEN foragers, with a patch specific mortalityRiskPollen:
ask foragerSquadrons with [ activity = "bringingPollen" ]
[
  if random-float 1 < ([ mortalityRiskPollen ] of flowerPatch knownPollenPatch)
  [
    set DeathsAdultWorkers_t DeathsAdultWorkers_t + SQUADRON_SIZE
    set DeathsForagingToday DeathsForagingToday + SQUADRON_SIZE
    set SumLifeSpanAdultWorkers_t SumLifeSpanAdultWorkers_t + (age * SQUADRON_SIZE)
    die
```

```
    ]
  ]
end;

; ****************************************************************************************************************

to Foraging_dancingProc
  ; foragers dance for a good patch and recruit 2 pollen foragers or up to 5 nectar foragers
  ; to the advertised patch

  let EEFdancedPatch -999   ; correct number set later
    ; energetic efficiency of the flower patch danced for (set to nonsense number as control)

  let tripDurationDancedPatch -999  ; correct number set later
    ; trip duration to a pollen patch

  let patchNumberDanced -999  ; correct number set later
    ; ...and the number of that flower patch

  ask foragerSquadrons with
    [ activity = "bringingNectar"
      or activity = "bringingPollen" ]
      ; successful pollen or nectar foragers are addressed
  [

; nectar foragers
    if activity = "bringingNectar"
    [
      set EEFdancedPatch [ EEF ] of flowerPatch knownNectarPatch
      set patchNumberDanced knownNectarPatch
        ; successful foragers dance; they communicate EEF and ID of flowerPatch
```

```
let danceFollowersNectarNow
  random-poisson [ danceFollowersNectar ] of flowerPatch knownNectarPatch

if [ danceFollowersNectarNow ] of flowerPatch knownNectarPatch >= 1
[
  set activityList lput "Dn" activityList
]

if ( count foragerSquadrons with
    [ activity = "resting" ]) >=
        [ danceFollowersNectarNow ] of flowerPatch knownNectarPatch
        ; only if enough resting foragers are present, there will be dances
[
  ask n-of
        ([ danceFollowersNectarNow ] of flowerPatch knownNectarPatch)
           foragerSquadrons with [ activity = "resting" ]
              ; depending on EEF of the patch, (0-5) resting foragers will follow the dance
  [
    ifelse knownNectarPatch = -1
      [    ; unexperienced foragers will always accept the advertised patch:
        set knownNectarPatch patchNumberDanced
        set activity "recForaging"
        set pollenForager false
          ; and become a nectar forager
        set activityList lput "rFnNF" activityList
      ]
      [
        ifelse EEFdancedPatch > [ EEF ] of flowerPatch knownNectarPatch
          ; if(2) ; experienced foragers: if the advertised patch has higher EEF
          ; than the known flowerPatch,
          [
```

```
            set knownNectarPatch patchNumberDanced
              ; the dance follower will switch to new patch

            set pollenForager false
              ; and become a nectar forager

            set activity "recForaging"
            set activityList lput "rFnxNF" activityList
           ]
           [ ; ELSE 2 (i.e. experienced foragers, knowing a BETTER patch) are activated
            set activity "expForaging"
            set activityList lput "Xnr" activityList
           ]  ; else (2) they become active foragers to their own, known patch
         ]
       ]
     ]
   ]


; pollen foragers
   if activity = "bringingPollen"                                          [
    set tripDurationDancedPatch [ tripDurationPollen ] of flowerPatch knownPollenPatch
    set patchNumberDanced knownPollenPatch
    if POLLEN_DANCE_FOLLOWERS >= 1 ; pollen foragers dance ALWAYS (as POLLEN_DANCE_FOLLOWERS = 2)
    [
      set activityList lput "Dp" activityList
    ]

    if ( count foragerSquadrons with [ activity = "resting" ])
      >= POLLEN_DANCE_FOLLOWERS
        ; only if enough resting foragers are present, there will be dances
    [
      ask n-of POLLEN_DANCE_FOLLOWERS foragerSquadrons
```

```
      with [ activity = "resting" ]
      ; # pollen dance followers: constant and independent of patch distance!!
    [
      ifelse knownPollenPatch = -1
        [  ; unexperienced forager will always accept the advertised patch:
          set knownPollenPatch patchNumberDanced
          set activity "recForaging"

          ; and become a pollen forager:
          set pollenForager true
          set activityList lput "rFpPF" activityList
        ]
        [ ; if(2) ; experienced foragers: if the advertised patch offers a
          ;  shorter trip duration than the known pollen patch..
          ifelse tripDurationDancedPatch < [ tripDurationPollen ]
            of flowerPatch knownPollenPatch
            [ ; .. then the dance follower will switch to new patch
              set knownPollenPatch patchNumberDanced
              ; and become a pollen forager:
              set pollenForager true

              set activity "recForaging"
              set activityList lput "rFpxPF" activityList
            ]
            [ ; else (2) they become active foragers to their own, known patch:
              set activity "expForaging"
              set activityList lput "Xpr" activityList
            ]
        ]
    ]
  ]
]
```

```
  ]

end;

; ************************************************************************************************************************

to Foraging_unloadingProc
  ; successful foragers increase honey or pollen store of the colony and become experienced foragers

  ask foragerSquadrons with [ activity = "bringingNectar" ]
  [
    set HoneyEnergyStore HoneyEnergyStore + (cropEnergyLoad * SQUADRON_SIZE)

    if HoneyEnergyStore > MAX_HONEY_ENERGY_STORE
    [
      set HoneyEnergyStore MAX_HONEY_ENERGY_STORE
    ]  ; honey store can't be larger than maximum

    set activityList lput "bN" activityList
    set cropEnergyLoad 0
    set activity "expForaging"
    set activityList lput "Xn" activityList
  ]

  ask foragerSquadrons with [ activity = "bringingPollen" ]
  [
    set PollenStore_g PollenStore_g + (collectedPollen * SQUADRON_SIZE)
    set collectedPollen 0
    set activityList lput "bP" activityList
    set activity "expForaging"
    set activityList lput "Xp" activityList
```

```
  ]

  ask foragerSquadrons with [ activity = "searching" ]
  [
    set activityList lput "E" activityList
  ]  ; unsuccessful souts return empty

end;

; ************************************************************************************************************************

to ForagersLifespanProc
  ; foragers also die due to age, max. travelled distance or by chance inside
  ; the colony; dying foragers are counted to calculate mean lifespan

  ask foragerSquadrons
  [
    if age >= LIFESPAN
    [
      set DeathsAdultWorkers_t DeathsAdultWorkers_t + SQUADRON_SIZE
      set SumLifeSpanAdultWorkers_t SumLifeSpanAdultWorkers_t + (age * SQUADRON_SIZE)
      die
    ]

    if mileometer >= MAX_TOTAL_KM
    [
      set DeathsAdultWorkers_t DeathsAdultWorkers_t + SQUADRON_SIZE
      set DeathsForagingToday DeathsForagingToday + SQUADRON_SIZE
      set SumLifeSpanAdultWorkers_t SumLifeSpanAdultWorkers_t + (age * SQUADRON_SIZE)
      die
    ]
```

```
    let dailyRiskToDie MORTALITY_INHIVE
      ; the daily background mortality of (healthy) foragers, which is equal to MORTALITY_INHIVE of the inhive bees

    if infectionState = "infectedAsPupa"
    [
      set dailyRiskToDie MORTALITY_INHIVE_INFECTED_AS_PUPA
    ]  ; except for infected as pupa foragers, which have a higher mortality

    if infectionState = "infectedAsAdult"
    [
      set dailyRiskToDie MORTALITY_INHIVE_INFECTED_AS_ADULT
    ]  ; except for infected as adult foragers, which have a higher mortality

    if random-float 1 < dailyRiskToDie
    [
      set DeathsAdultWorkers_t DeathsAdultWorkers_t + SQUADRON_SIZE
      set SumLifeSpanAdultWorkers_t SumLifeSpanAdultWorkers_t + (age * SQUADRON_SIZE)
      die
    ]
  ] ; ask foragerSquadrons
end;

; *********************************************************************************************************************

==========================================================================
;  ================   END OF IBM FORAGING SUBMODEL ===================
==========================================================================

;
**********************************************************************************************************************
```

```
; THE VARROA MITE SUBMODEL =====

*****************************************************************************************************************

to MiteProc
; calls the Varroa related procedures
  CreateMiteOrganisersProc
  CountingProc ; updating number of brood & adults of drones & workers
  MitesInvasionProc
  MitePhoreticPhaseProc
  MiteDailyMortalityProc
  MiteOrganisersUpdateProc
end

; ****************************************************************************************************************

to CreateMiteOrganisersProc
  ; called by MiteProc, creates a single miteOrganiser turtle, that
  ; stores info on number and distribution of mites newly invaded into the brood cells

  create-miteOrganisers 1
  [
    setxy -1 -7
    set heading 0
    set size 1.3
    set color 33.5
    set shape "VarroaMite03"   ;"Virus1" ;"VarroaMite03"
    set workerCellListCondensed n-values (MAX_INVADED_MITES_WORKERCELL + 1) [ 0 ]
```

```
    ; +1 as also the number of mite free cells is stored in this list

   set droneCellListCondensed n-values (MAX_INVADED_MITES_DRONECELL + 1) [ 0 ]
    ; +1 as also the number of mite free cells is stored in this list

   set label-color white
   set cohortInvadedMitesSum 0
    ; sum of all mites that invaded a worker or drone cell on the same Day

   set invadedMitesHealthyRate PhoreticMitesHealthyRate
    ; rate of healthy mites in this cohort of invading mites equals the rate of healthy
    ; phoretic mites on this day

   set age INVADING_WORKER_CELLS_AGE
    ; "age" refers to age of invaded brood. If age for invasion differs in
    ; worker and drone brood..

   if INVADING_DRONE_CELLS_AGE < INVADING_WORKER_CELLS_AGE
   [
    set age INVADING_DRONE_CELLS_AGE
   ] ; ..then age refers to the younger of both
  ]
end

; ****************************************************************************************************************************

to MitesInvasionProc
  ; called by MiteProc  calculates the number of phoretic mites that
  ; enter worker and drone brood cells on this day based on: Calis et al. 1999, Martin 2001

  let factorDrones 6.49  ; (Boot et al. 1995, Martin 2001)
```

```
let factorWorkers 0.56 ; (Boot et al. 1995, Martin 2001)
let adultsWeight_g (TotalIHbees + TotalForagers) * WEIGHT_WORKER_g
  ; weight of all adult worker bees
let invadingBroodCellProb 0
  ; probability for a phoretic mite to enter any suitable brood cell
let invadingWorkerCellProb 0
  ; probaility to invade a worker cell (only if any cell was invaded)
let suitableWorkerCells 0
let suitableDroneCells 0
  ; number of worker and drone cells, that are suitable for mite invasion
let rD 0
let rW 0
  ; rD, rW: Rate of invasion into Drone cells and Worker cells (Boot et al. 1995)

ask larvaeCohorts with [ age = INVADING_WORKER_CELLS_AGE ]
[
  set suitableWorkerCells number
] ; (age = 8) mites enter worker larvae cells ~1d before capping (at 9d age) (Boot, Calis, Beetsma 1992)

ask droneLarvaeCohorts with [ age = INVADING_DRONE_CELLS_AGE ]
[
  set suitableDroneCells number
]  ; (age = 8) mites enter drone larvae cells ~ 2d before capping (at 10d age) (Boot, Calis, Beetsma 1992)

if adultsWeight_g > 0
[ ; invasion rates in worker and drone cells:
  set rW factorWorkers * (suitableWorkerCells / adultsWeight_g)  ; (Martin 1998, 2001; Calis et al.1999)
  set rD factorDrones * (suitableDroneCells / adultsWeight_g)
]

let exitingMites 0
  ; # mites, that theoretically should invade cells but leave it immediatly,
```

```
  ; because the cell is already invaded by the max. number of mites

let workerCellListTemporary n-values suitableWorkerCells [ 0 ]
  ; two temporary lists of all suitable worker/drone cells, to store
  ; the number of mites in each cell..

let droneCellListTemporary n-values suitableDroneCells [ 0 ]
  ; .. of which later the number of cells invaded by 0, 1, 2.. mites can be calculated

let cell -1
  ;  stores randomly chosen cell, which is invaded by a mite in the below
  ; "repeat phoreticMites.." process. -1 will be changed to a random number >= 0

set InvadingMitesWorkerCellsTheo 0
set InvadingMitesDroneCellsTheo 0
set invadingBroodCellProb (1 - (exp (-(rW + rD))))
  ; probability for a phoretic mite to enter a brood cell; similar to
  ; Martin 2001, however: we use probability instead of proportion

if rW + rD > 0 ; if invasion takes place..
[
  set invadingWorkerCellProb (rW / (rW + rD))
]

; based on the Boot/Martin/Calis rates of cell invasion, which are used as probabilities,
; it is calculated how many phoretic mites enter a brood cell, and whether it is
; a drone or a worker cell; each invading mite is then associated with a random brood
; cell number (WorkerCellsInvasionList), finally, the mites in each "brood cell" are
; counted and saved in the condensed nMitesInCellsList
repeat PhoreticMites
[
  if random-float 1 < invadingBroodCellProb
```

```
  ; mites have a chance to enter a brood cell
 [
  ifelse random-float 1 < invadingWorkerCellProb ; the brood cell might be a WORKER cell
   [
    set InvadingMitesWorkerCellsTheo InvadingMitesWorkerCellsTheo + 1
     ; mites entering worker cells are counted

    set cell random suitableWorkerCells
     ; randomly, one of the suitable WORKER cells is invaded by a mite

    set WorkerCellListTemporary replace-item cell WorkerCellListTemporary
     (item cell WorkerCellListTemporary + 1)
     ; this list contains all worker cells and the number of mites
     ; invading into each cell
   ]
   [
    ; ELSE: invasion into DRONE cell
    set InvadingMitesDroneCellsTheo InvadingMitesDroneCellsTheo + 1
    set cell random suitableDroneCells
     ; randomly, one of the suitable drone cells is invaded by a mite

    set DroneCellListTemporary replace-item cell DroneCellListTemporary
     (item cell DroneCellListTemporary + 1)
     ; this list contains all drone cells and the number of mites
     ; invading into each cell
   ]
 ]
]

; excess of invaded mites: # mites in each cells is restricted to MAX_INVADED_MITES:
let counter 0
foreach WorkerCellListTemporary
```

```
[
  ; (note: items are addressed in ordered way - NOT randomly)
  if ? > MAX_INVADED_MITES_WORKERCELL
  [
    set exitingMites exitingMites + (? - MAX_INVADED_MITES_WORKERCELL)
      ; if too many mites in cells: they leave the cell ("?": # of mites in the cell)

    set WorkerCellListTemporary replace-item
      counter WorkerCellListTemporary MAX_INVADED_MITES_WORKERCELL
        ; .. mites left in the cell = max. mites in worker cell
  ]

  set counter counter + 1
]
set InvadingMitesWorkerCellsReal InvadingMitesWorkerCellsTheo - exitingMites

; and the same for the drones..
set counter 0  ; resetting the counter

foreach DroneCellListTemporary
[
  if ? > MAX_INVADED_MITES_DRONECELL
  [
    set exitingMites exitingMites + (? - MAX_INVADED_MITES_DRONECELL)
      ; if too many mites in cells: they leave the cell ("?": # of mites in the cell)

    set DroneCellListTemporary replace-item counter
      DroneCellListTemporary MAX_INVADED_MITES_DRONECELL
      ; .. mites left in the cell = max. mites in drone cell
  ]
  set counter counter + 1
]
```

```
set InvadingMitesDroneCellsReal InvadingMitesDroneCellsTheo
  - exitingMites
  + (InvadingMitesWorkerCellsTheo - InvadingMitesWorkerCellsReal)
    ; mites invaded drone cells = mites theor. invading drone cells
    ; - mites exiting drone&worker cells
    ; + mites exiting worker cells (here: exitingMites: sum of worker&drone cell mites!)

set PhoreticMites PhoreticMites
  - InvadingMitesWorkerCellsTheo
  - InvadingMitesDroneCellsTheo
  + exitingMites
    ; # of phoretic mites left (=phor.mites - invading mites
    ; + mites immediately leaving cells and become phoretic again

if PhoreticMites < 0
[
  user-message "Error in MitesInvasionProc - negative number of phoretic Mites"
  set BugAlarm true
] ; assertion

let memory -1 ; -1: = no cohort invaded

ask miteOrganisers with [age = INVADING_WORKER_CELLS_AGE]
[
  foreach workerCellListTemporary
    ; checks the list that contains all worker brood cells for
    ; how many mites have entered..
  [
    set workerCellListCondensed replace-item ? workerCellListCondensed
      ((item ? workerCellListCondensed) + 1)
  ]  ; sums up the number of cells entered by 0, 1,2..n mites in the mitesOrganisers own list
```

```
  set cohortInvadedMitesSum cohortInvadedMitesSum + InvadingMitesWorkerCellsReal

  let whoMO who  ; stores the "who" of the current miteOrganiser
  ask larvaeCohorts with [age = INVADING_WORKER_CELLS_AGE]
  [
    set invadedByMiteOrganiserID whoMO
    set memory who
  ]
  set invadedWorkerCohortID memory
] ; "ask miteorganisers ..."

ask miteOrganisers with [age = INVADING_DRONE_CELLS_AGE]
[
  foreach droneCellListTemporary
    ; checks the list that contains all drone brood cells for
    ; how many mites have entered..
  [
    set droneCellListCondensed replace-item ? droneCellListCondensed
      ((item ? droneCellListCondensed) + 1)
  ] ; sums up the cell entered by 0, 1,2..n mites in the mitesOrganisers own list

  set cohortInvadedMitesSum cohortInvadedMitesSum + InvadingMitesDroneCellsReal
  set memory -1  ; -1: = no cohort invaded

  ask droneLarvaeCohorts with [age = INVADING_DRONE_CELLS_AGE]
  [
    set memory who
  ]
  set invadedDroneCohortID memory
  let whoMO who  ; stores the "who" of the current miteOrganiser
```

```
    ask droneLarvaeCohorts with [ age = INVADING_DRONE_CELLS_AGE ]
    [
      set invadedByMiteOrganiserID whoMO
    ]
  ] ; "ask miteOrganisers with ..."

  if (PhoreticMites + InvadingMitesWorkerCellsReal
    +  InvadingMitesDroneCellsReal) > 0 ; avoid div 0!
  [
    set PropNewToAllPhorMites NewReleasedMitesToday
      / ( PhoreticMites + InvadingMitesWorkerCellsReal + InvadingMitesDroneCellsReal)
  ] ; Proportion of new emerged phoretic mites (today) to all phoretic mites
    ; present (needed in the MitePhoreticPhaseProc to determine # of newly infected phoretic mites etc)
end

; ****************************************************************************************************************************


to-report MiteDensityFactorREP [ ploidyMiteOrg mitesIndex ]
  ; reports the (single) density factor for a certain number of invaded mites
  ; depending on ploidy of bee brood and chosen reproduction model

  let dataList []

  if MiteReproductionModel = "Martin"
  [ ifelse ploidyMiteOrg = 2
    [ set dataList [ 0 1 0.91 0.86 0.60 ] ]
      ; workers  (list length: 5) [ 0 1 0.91 0.86 0.60 ]
      ; from Martin 1998, Tab. 4; first value (0) doesn't matter, as no
      ; mother mite invaded these cells

    [ set dataList [ 0 1 0.84 0.65 0.66 ] ]
```

```
]   ; drones (list length: 5)  [ 0 1 0.84 0.65 0.66 ] from Martin 1998, Tab. 4

if MiteReproductionModel = "Fuchs&Langenbach"
[
  ifelse ploidyMiteOrg = 2
    [ set dataList [ 0 1 0.96 0.93 0.89 0.86 0.82 0.79 0 ]]
      ; workers   (list length: 9) calculated from Fuchs&Langenbach 1989 Tab.III
    [ set dataList [ 0 1 0.93 0.86 0.80 0.73 0.66 0.59 0.52 0.45 0.39 0.32 0.25 0.18 0.11 0.05 0 ] ]
]  ; (list length: 17) calculated from Fuchs&Langenbach 1989 Tab.III

if MiteReproductionModel = "No Mite Reproduction"  ; only for model testing
[
  ifelse ploidyMiteOrg = 2
    [ set dataList [ 0 1 1 1 1 1 ] ] ; workers   (list length: 6)
    [ set dataList [ 0 1 1 1 1 1 ] ]
]  ; drones  (list length: 6)

if MiteReproductionModel = "Martin+0"
  ; like Martin, but max # of mites in brood cell is increased by
  ; one with a rel. reprod. rate of 0 (= 0 at the end of the list)

[ ; Martin Test with 0
  ifelse ploidyMiteOrg = 2
    [ set dataList [ 0 1 0.91 0.86 0.60 0 ] ]
      ; workers   (list length: 6) [ 0 1 0.91 0.86 0.60 0 ]
      ; from Martin 1998, Tab. 4; first value (0) doesn't matter, as no
      ; mother mite invaded these cells
    [ set dataList [ 0 1 0.84 0.65 0.66 0 ] ]
]  ; drones (list length: 6)  [ 0 1 0.84 0.65 0.66 0 ] from Martin 1998, Tab. 4

report item mitesIndex dataList
```

```
end

; ******************************************************************************************************************

to-report  MiteOffspringREP [ ploidyMiteOrg ]
  ; reports offspring per mite depending on ploidy of bee brood and chosen reproduction model

  let result 0
  if ploidyMiteOrg != 1 and ploidyMiteOrg != 2
  [
    set BugAlarm true
    type "BUG ALARM in MiteOffspringREP! Wrong ploidyMiteOrg: "
    print ploidyMiteOrg
  ]

  if MiteReproductionModel = "Martin" or MiteReproductionModel = "Martin+0"
  [
    ifelse ploidyMiteOrg = 2
      [ set result 1.01 ]
        ; workers (1.01: Martin 1998; fertilisation already taken into account)

      [ set result 2.91 ]
  ]     ; drones (2.91: Martin 1998; fertilisation already taken into account)

 if MiteReproductionModel = "Fuchs&Langenbach"
 [
   ifelse ploidyMiteOrg = 2
     [ set result 1.4 * 0.95 ]
       ; workers (1.4: Fuchs&Langenbach 1989; of which 5% are
       ; unfertilised (Martin 1998 p.271))
     [ set result 2.21 * 0.967 ]
```

```
 ] ; drones (2.21: Fuchs&Langenbach 1989; of which 3.3% are unfertilised (Martin 1998 p.271))

 if MiteReproductionModel = "No Mite Reproduction"  ; only for model testing
 [
   ifelse ploidyMiteOrg = 2
      [ set result 0 ]   ; workers
      [ set result 0 ]
 ] ; drones

 report result
end

; ************************************************************************************************************************

; MitesReleaseProc: determines how many healthy and infected mites emerge from cells with a) dead or b) emerging bees
; CALLED BY: WorkerLarvaeDevProc (dying), DroneLarvaeDevProc (dying), WorkerPupaeDevProc (2x, for dying & emerging brood)
; DronePupaeDevProc (2x, for dying & emerging brood), BroodCareProc (4x, dying of drone & worker larvae & pupae)

; .. all these procedures are called BEFORE the mite module (MiteProc)!


to MitesReleaseProc [ miteOrganiserID ploidyMiteOrg diedBrood releaseCausedBy ]
    ; 1. rate of healthy mites in the cellList 2. the relevant worker/drone
    ; cellListCondensed 3.  # died broodCells (0..n) 4. "emergingBrood" or "dyingBrood"

  let cellListCondensed []
    ; to not double the code for worker and drones, the local variable
    ; cellListCondensed is defined which stores EITHER the workerCellListCondensed
    ; OR the droneCellListCondensed

  let mitesInfectedSumUncappedCells 0
    ; sums up the infected mites of the current cohort
```

```
let mitesHealthySumUncappedCells 0 ; sums up the healthy mites of the current cohort
let mitesHealthy&InfectedSumUncappedCells 0
  ; sums up the healthy and infected mites of the current cohort

let nPhoreticMitesBeforeEmergenceHealthy round (PhoreticMitesHealthyRate * PhoreticMites)
  ; saves the number healthy phoretic mites before the new mites emerge from their
  ; cells - necessary to calculate new PhoreticMitesHealthyRate

let nPhoreticMitesBeforeEmergenceInfected PhoreticMites - nPhoreticMitesBeforeEmergenceHealthy
  ; saves the number infected phoretic mites before the new mites emerge from
  ; their cells - necessary to calculate new PhoreticMitesHealthyRate

let healthyRateMiteOrg 0
  ; proportion of healthy mites in the current cohort (miteOrganiser)

let totalCells 0
  ; number of brood cells in the current cohort

let releasedPupaeCohortsID -1

let repetitions MAX_INVADED_MITES_WORKERCELL + 1
  ; to count the brood cells; (for worker cells); +1 as cells can also bee mite free
if ploidyMiteOrg = 1
[
  set repetitions MAX_INVADED_MITES_DRONECELL + 1
] ; ..the same for drone cells, +1 as cells can also bee mite free

; to save the required "cellListCondensed" and to determine the "who"
; of the affected (worker or drone) pupaeCohort:
ask miteOrganisers with [ who = miteOrganiserID ]
[
```

```
  ifelse ploidyMiteOrg = 1
    [
      set cellListCondensed droneCellListCondensed
        ; IF DRONES: local cellListCondensed =  droneCellListCondensed
      set releasedPupaeCohortsID invadedDroneCohortID
    ]   ; ... and affected droneCohort is the miteOrganisers "invadedDroneCohortID"
    [
      set cellListCondensed workerCellListCondensed
        ; ELSE WORKERS: local cellListCondensed = workerCellListCondensed
      set releasedPupaeCohortsID invadedWorkerCohortID
    ]  ; ... and affected workerCohort is the miteOrganisers "invadedWorkerCohortID"
  set healthyRateMiteOrg invadedMitesHealthyRate
    ; saves the rate of healthy mites invaded to the current miteOrganiser
]

let i 0
repeat repetitions
; repetitions = MAX_INVADED_MITES_WORKER/DRONE_CELL + 1
[
  ; counts the # of cells in the cellList
  set totalCells totalCells + (item i cellListCondensed)
  set i i + 1
]

let uncappedCells 0  ; number of cells that are uncapped ...
if releaseCausedBy = "dyingBrood" [ set uncappedCells diedBrood  ]
  ; .. because some pupae died..

if releaseCausedBy = "emergingBrood" [ set uncappedCells totalCells  ]
  ; .. or because all pupae emerge

if releaseCausedBy != "dyingBrood" and  releaseCausedBy != "emergingBrood"
```

```
[
  set BugAlarm true
  type "BUG ALARM in ReleaseMitesProc(1)! releaseCausedBy: "
  print releaseCausedBy
]  ; assertion

repeat uncappedCells
[
  ; uncapped brood cells are randomly chosen from all brood cells of
  ; this cohort. These cells may contain 0,1,2..invadedMitesCounter mites.
  ; These mother mites are released from the cell WITH OR WITHOUT
  ; reproduction and become phoretic

  let randomCell (random totalCells) + 1
    ; choses a random cell -> 1..totalCells (+1 as: random n = 0, 1, ..n-1)
    ; (totalCells is decreased at the end of each repetition by 1)

  let cellCounter 0
  let allMitesInSingleCell -1
    ; starting value of allMitesInSingleCell: -1 as it is increased by 1 in the
    ; following "while" loop
    ; allMitesInSingleCell: # of mites that invaded the randomly chosen cell

  while [ cellCounter < randomCell ]
    ; determines, by how many mites the "random cell"
    ; is invaded: sums up the # of cells invaded by 0 mites (1st loop)
    ; by 1 mite (2nd loop) etc. until the cellCounter >= randomCell
    ; the number of mites in random cell is then allMitesInSingleCell
  [
    set allMitesInSingleCell allMitesInSingleCell + 1
      ; in 1st loop: allMitesInSingleCell = 0! (i.e. item 0 = first item in list = 0 mites)
      ; in 2nd loop: 1 mite etc.
```

```
  set cellCounter cellCounter + (item allMitesInSingleCell cellListCondensed)
    ; cellCounter is increased by the # of cells with x mites in it
    ; (x = allMitesInSingleCell, i.e. 0,1,2..n)
]

; how many of the released mites are infected? -> 1. how many infected
; mites entered? 2. did they infect the larva? 3. how many healthy mites become
; infected by the infected larva?
let mitesIndex allMitesInSingleCell
  ; to address the correct item in the cellListCondensed after mite
  ; reproduction (i.e. when allMitesInSingleCell has changed)

let pupaInfected false ; a young larva is healthy
let infectedMitesInSingleCell 0
  ; the number of mites that were diseased on day of cell invasion

repeat allMitesInSingleCell
[
  ; invaded mites might be infected: repeat over all mites in the current brood cell
  if random-float 1 > healthyRateMiteOrg
  [
    set infectedMitesInSingleCell infectedMitesInSingleCell + 1
    ; this invaded mite was infected when invading the cell and is now counted as infected
  ]
]

let healthyMitesInSingleCell allMitesInSingleCell - infectedMitesInSingleCell
  ; healthy invaded mites are all invaded mites minus infected ones

if random-float 1 > (1 - VIRUS_TRANSMISSION_RATE_MITE_TO_PUPA) ^ infectedMitesInSingleCell
[
```

```
    set pupaInfected true
  ] ; as soon as at least 1 infected mite successfully infects the bee pupa, the bee pupa is infected


   ; pupa alive or dead?
; (either died normally, died due to lack of nursing or killed by virus
  let pupaAlive 1 ; (0 or 1) 1: = "yes", pupa is alive 0: = "no", pupa is dead
  if pupaInfected = true
  [
    if random-float 1 < VIRUS_KILLS_PUPA_PROB
    [
      set pupaAlive 0
    ]
  ] ; infected pupa might be killed by the virus. In this case:
    ; no offspring mites but still transmission of viruses to healthy mites in this cell
    ; (at least for DWV)

  if releaseCausedBy = "dyingBrood"
  [
    set pupaAlive 0
  ] ; larva/pupa is dead, if MitesReleaseProcis called, BECAUSE the brood died..

  if releaseCausedBy = "emergingBrood" and allMitesInSingleCell > 0
  [
    ; callow bees are emerging and with them the invaded mother mites and their offspring
    if pupaAlive = 0
    [
      ask turtles with [ who = releasedPupaeCohortsID ]
      [
        set number number - 1
          ; pupa died, hence the number of bees in this pupae cohort is reduced by 1
        set number_healthy number_healthy - 1
          ; pupa dies due to virus infection and has previously been healthy
```

```
    set Pupae_W&D_KilledByVirusToDay Pupae_W&D_KilledByVirusToDay + 1
  ]
 ]


; surviving but infected pupae:
 if pupaAlive = 1 and pupaInfected = true
 [
  ask turtles with [ who = releasedPupaeCohortsID ]
  [
    set number_infectedAsPupa number_infectedAsPupa + 1
    ; the bee was infected as pupa
    set number_healthy number_healthy - 1
    ; the pupa has become infected and is no longer healthy
  ]
 ]

 let averageOffspring
  random-poisson (MiteOffspringREP ploidyMiteOrg * MiteDensityFactorREP ploidyMiteOrg mitesIndex)
  ; average # offspring of a single mother mite in the single cell (depends on ploidy of bee pupa and # invaded mites)

 set healthyMitesInSingleCell allMitesInSingleCell
  * averageOffspring
     ; Offspring: all mites in cell x reprod. rate. NOTE: also infected mites
     ; may have healthy offspring! (MiteOffspringREP: reports # offspring for
     ; 1 mite in single invaded cell, for drones or workers)
  * pupaAlive
    ; pupaAlive =  1 or 0; if pupa is alive: normal mite reproduction, if dead:
    ; offspring = 0
  + healthyMitesInSingleCell            ; + mother mites

 set healthyMitesInSingleCell round healthyMitesInSingleCell
 ; this line is NOT NECESSARY as averageOffspring is integer!
```

```
  set allMitesInSingleCell healthyMitesInSingleCell + infectedMitesInSingleCell
    ; update of total mites in the cell
]  ; END of "if releaseCausedBy = 'emergingBrood' "

if pupaAlive = 1 and pupaInfected = true
[
  ; if the bee pupa was infected by an infected mite AND IS STILL ALIVE,
  ; then the healthy mites (invaded or offspring) might become infected too

  repeat healthyMitesInSingleCell
  [
    ; all healthy mites have then the risk to become infected too
    if random-float 1 < VIRUS_TRANSMISSION_RATE_PUPA_TO_MITES
    ; if random number < the transmission rate from bee pupa to mite, the healthy
    ; mite becomes infected
    [
      set healthyMitesInSingleCell healthyMitesInSingleCell - 1
        ; hence: the number of healthy released mites decreases by 1..

      set infectedMitesInSingleCell infectedMitesInSingleCell + 1
    ]  ; .. and the number of infected released mites increases by 1
  ] ; end of 'repeat sumInvadedMitesHealthy'
] ; end of 'IF pupaInfected' - now the numbers of healthy and infected (mother) mites in
; single cell is known (= healthyMitesInSingleCell and infectedMitesInSingleCell)

if healthyMitesInSingleCell + infectedMitesInSingleCell != allMitesInSingleCell
[
  set BugAlarm true
  type "BUG ALARM in ReleaseMitesProc(2)! allMitesInSingleCell: "
  type allMitesInSingleCell
  type " infectedMitesInSingleCell: "
  type infectedMitesInSingleCell
```

```
  type " healthyMitesInSingleCell: "
  print healthyMitesInSingleCell
]


; mite fall:
let miteFallProb MITE_FALL_DRONECELL
if ploidyMiteOrg = 2
[
  set miteFallProb MITE_FALL_WORKERCELL
] ; probabilities of mites to fall from comb, depending on cell type

repeat healthyMitesInSingleCell
[ ; determined for healthy and infected mites separately
  if random-float 1 < miteFallProb
  [
    set healthyMitesInSingleCell healthyMitesInSingleCell - 1
    set allMitesInSingleCell allMitesInSingleCell - 1
    set DailyMiteFall DailyMiteFall + 1
  ]
]

repeat infectedMitesInSingleCell
[
  if random-float 1 < miteFallProb
  [
    set infectedMitesInSingleCell infectedMitesInSingleCell - 1
    set allMitesInSingleCell allMitesInSingleCell - 1
    set DailyMiteFall DailyMiteFall + 1
  ]
]

set mitesHealthySumUncappedCells mitesHealthySumUncappedCells + healthyMitesInSingleCell
```

```
  ; sums up all healthy mites emerging from current cohort
  ; (set to 0 at beginning of this procedure)

set mitesInfectedSumUncappedCells mitesInfectedSumUncappedCells + infectedMitesInSingleCell
  ; same for infected mites (set to 0 at beginning of this procedure)

set PhoreticMites PhoreticMites + allMitesInSingleCell
  ; mother mites in this uncapped brood cell are released from the brood
  ; cell and become phoretic..

set mitesHealthy&InfectedSumUncappedCells
  mitesHealthy&InfectedSumUncappedCells + allMitesInSingleCell
    ; released mites from all brood cell in this cohort are totaled up

set cellListCondensed replace-item mitesIndex cellListCondensed
  (item mitesIndex cellListCondensed - 1)
    ; .. and one brood cell is removed; mitesIdex: number of mother mites that
    ; invaded the brood cell

if item mitesIndex cellListCondensed < 0
[
  set BugAlarm true
  type "BUG ALARM in ReleaseMitesProc(3)! Negative number in cellListCondensed (releaseMitesProc)! "
  show cellListCondensed
]

set totalCells totalCells - 1
  ; number of total brood cells in this cohort is reduced by 1

if totalCells < 0
[
  set BugAlarm true
```

```
    type "BUG ALARM in ReleaseMitesProc(4)! Negative number of  total cells in releaseMitesProc: "
    print totalCells
  ]
] ; END OF "REPEAT UNCAPPEDCELLS"

set NewReleasedMitesToday
  NewReleasedMitesToday + mitesHealthy&InfectedSumUncappedCells
    ; # of newly released (mother+offspring) mites (only those that survived
    ; MiteFall) is summed up (set to 0 in DailyUpdateProc)

if mitesInfectedSumUncappedCells + mitesHealthySumUncappedCells
   != mitesHealthy&InfectedSumUncappedCells
[ ; assertion
  set BugAlarm true
  type "BUG ALARM in ReleaseMitesProc(5)! mitesInfectedSumUncappedCells: "
  type mitesInfectedSumUncappedCells
  type " mitesHealthySumUncappedCells: "
  type mitesHealthySumUncappedCells
  type " mitesHealthy&InfectedSumUncappedCells: "
  print mitesHealthy&InfectedSumUncappedCells
]

if mitesInfectedSumUncappedCells < 0 or mitesHealthySumUncappedCells < 0
[ ; assertion
  set BugAlarm true
  type "BUG ALARM in ReleaseMitesProc(6)! mitesInfectedSumUncappedCells: "
  type mitesInfectedSumUncappedCells
  type " mitesHealthySumUncappedCells: "
  type mitesHealthySumUncappedCells
  type " mitesHealthy&InfectedSumUncappedCells: "
  print mitesHealthy&InfectedSumUncappedCells
]
```

```
; Updating of the actual cell lists - either for the drone or for the worker brood:
 ask miteOrganisers with [ who = miteOrganiserID ]
 [ ; assertion
   if ploidyMiteOrg = 1 [ set droneCellListCondensed cellListCondensed ]    ; IF drones
   if ploidyMiteOrg = 2 [ set workerCellListCondensed cellListCondensed ]    ; IF workers
   if (ploidyMiteOrg != 1) and (ploidyMiteOrg != 2)
   [
     set BugAlarm true
     type "BUG ALARM in releaseMitesProc(7)! Wrong ploidyMiteOrg: "
     print ploidyMiteOrg
   ]
                                                    ]
 ; UPDATE of the healthy mite rate:
 if ( nPhoreticMitesBeforeEmergenceHealthy
      + nPhoreticMitesBeforeEmergenceInfected
      + mitesHealthySumUncappedCells
      + mitesInfectedSumUncappedCells) > 0
 [
   set PhoreticMitesHealthyRate
     ( nPhoreticMitesBeforeEmergenceHealthy + mitesHealthySumUncappedCells)
      / ( nPhoreticMitesBeforeEmergenceHealthy
          + nPhoreticMitesBeforeEmergenceInfected
          + mitesHealthySumUncappedCells
          + mitesInfectedSumUncappedCells )
 ]

end

; ****************************************************************************************************************
```

```
to MiteDailyMortalityProc
  ifelse ( TotalEggs + TotalLarvae
         + TotalPupae + TotalDroneEggs
         + TotalDroneLarvae + TotalDronePupae) > 0 ; is it within brood period?
    [
      set PhoreticMites
       (PhoreticMites - random-poisson (PhoreticMites *  MITE_MORTALITY_BROODPERIOD))
    ]  ; IF brood is present
    [
      set PhoreticMites
      (PhoreticMites - random-poisson (PhoreticMites *  MITE_MORTALITY_WINTER))
    ] ; ELSE: if no brood is present
end

; ******************************************************************************************************************************************


to MitePhoreticPhaseProc
  ; infection of healthy worker bees via infected phoretic mites and of
  ; healthy phoretic mites via infected workers; Called daily by MiteProc

  let healthyPhoreticMites round (PhoreticMites * PhoreticMitesHealthyRate)
    ; # of healthy, phoretic mites is calculated from the rate of healthy phoretic mites

  let infectedPhoreticMites PhoreticMites - healthyPhoreticMites
    ; all other phoretic mites are infected

  let phoreticMitesPerIHbee 0

  if ( TotalIHbees + InhivebeesDiedToday
       + NewForagerSquadronsHealthy
       + NewForagerSquadronsInfectedAsPupae
```

```
    + NewForagerSquadronsInfectedAsAdults > 0 ) ; avoid division by 0
[
  set phoreticMitesPerIHbee
    ( PhoreticMites - NewReleasedMitesToday)
      / (TotalIHbees + InhivebeesDiedToday
         + SQUADRON_SIZE *
           ( NewForagerSquadronsHealthy
             + NewForagerSquadronsInfectedAsPupae
             + NewForagerSquadronsInfectedAsAdults
           )
        )
] ; phoretic mites are assumed to infest only inhive bees,
  ; "ih-bees" here = current ih-bees + ih-bees died today
  ;                 + ih-bees developed into foragers today!

; mites are released from inhive bees, if ih-bees die or develop into foragers:
let mitesReleasedFromInhivebees
  precision
    (
     phoreticMitesPerIHbee
      * ( InhivebeesDiedToday  ; died ih-bees
          + SQUADRON_SIZE      ; new foragers:
           * ( NewForagerSquadronsHealthy
               + NewForagerSquadronsInfectedAsPupae
               + NewForagerSquadronsInfectedAsAdults
             )
         )
    ) 5

if mitesReleasedFromInhivebees > PhoreticMites
[
  set BugAlarm true
```

```
    type "BugAlarm!!! mitesReleasedFromInhivebees > PhoreticMites! mitesReleasedFromInhivebees: "
    type mitesReleasedFromInhivebees
    type " PhoreticMites: "
    print PhoreticMites
  ]

  let healthyPhoreticMitesSwitchingHosts
    round
      (
        mitesReleasedFromInhivebees * PhoreticMitesHealthyRate
        + PhoreticMites * PropNewToAllPhorMites * PhoreticMitesHealthyRate
      )  ; # healthy phoretic mites that infest a bee. These are: newly
         ; released mites that haven't entered a brood cell (hence:
         ; "phoreticMites * PropNewToAllPhorMites") and phoretic mites, where the host
         ; bee just died; all multiplied with PhoreticMitesHealthyRate as only healthy
         ; mites are considered

  if healthyPhoreticMitesSwitchingHosts > healthyPhoreticMites
  [
    ; set BugAlarm true
    if (healthyPhoreticMitesSwitchingHosts - healthyPhoreticMites) > 1
    [
      set BugAlarm true  ; if difference > 1 it can't be explained by rounding errors..
      type "BugAlarm!!! (MitePhoreticPhaseProc)  healthyPhoreticMitesSwitchingHosts > healthyPhoreticMites!
healthyPhoreticMitesSwitchingHosts: "
      type healthyPhoreticMitesSwitchingHosts
      type " healthyPhoreticMites: "
      print healthyPhoreticMites
    ]

    set healthyPhoreticMitesSwitchingHosts healthyPhoreticMites
  ] ; to ensure that not more mites switch their hosts than actually present!
```

```
; healthy and infected IN-HIVE bees:
let totalInfectedWorkers 0
let totalHealthyWorkers 0
ask IHbeeCohorts
[
  set totalInfectedWorkers
    totalInfectedWorkers + number_infectedAsPupa + number_infectedAsAdult
      ; infected: either during pupal phase or as adults
  set totalHealthyWorkers totalHealthyWorkers + number_healthy
]

; Infection of healthy mites:
let newlyInfectedMites 0
 ; the probability of healthy mites to become infected equals the proportion of
  ; infected in-hive workers to all in-hive workers:
if (totalInfectedWorkers + totalHealthyWorkers) > 0  ; avoid division by 0!
 [
  repeat healthyPhoreticMitesSwitchingHosts
   [
     if random-float 1 <  totalInfectedWorkers / (totalInfectedWorkers + totalHealthyWorkers)
      [
        set newlyInfectedMites newlyInfectedMites + 1
      ]
    ]
 ]

; infection of healthy adult workers - ONLY IN-HIVE WORKERS!
let allInfectedMitesSwitchingHosts
  round
    ( PhoreticMites * PropNewToAllPhorMites * (1 - PhoreticMitesHealthyRate)
      + mitesReleasedFromInhivebees * (1 - PhoreticMitesHealthyRate))
```

```
    ; # infected phoretic mites that infest a new bee. These are: newly
    ; released mites, that haven't entered a brood cell (hence: "phoreticMites
    ; * PropNewToAllPhorMites") and phoretic mites, where the host bee just died;
    ; all multiplied with (1 - PhoreticMitesHealthyRate) as only infected mites are considered

ask IHbeeCohorts
[
  if TotalIHbees > 0 and number > 0  ; avoid division by 0!
  [
    let infectedMitesSwitchingHostsInThisCohort
      (allInfectedMitesSwitchingHosts / TotalIHbees) *  number
        ; # of infected mites switching their host in current bee cohort: # mites per ih-bee * number of ih-bees
        ; in this cohort (assumes an equal distribution of mites)

    let newlyInfectedIHbeesInThisCohort 0
    repeat number_healthy  ; only healthy bees can become newly infected
      [
        if random-float 1 > (1 - (1 / number)) ^ infectedMitesSwitchingHostsInThisCohort
        ; "number" (i.e. all bees in this cohort) as mites can also jump on already infected bees
          [
            set newlyInfectedIHbeesInThisCohort newlyInfectedIHbeesInThisCohort + 1
             ; # of newly infected bees is increased by 1
            set infectedMitesSwitchingHostsInThisCohort infectedMitesSwitchingHostsInThisCohort - 1
            if infectedMitesSwitchingHostsInThisCohort < 0
              [ set infectedMitesSwitchingHostsInThisCohort 0 ]
          ]
      ]


    ; Assertion to be sure there are not more newly infected bees than there were healthy bees:
    if newlyInfectedIHbeesInThisCohort > number_healthy
    [
```

```
     set BugAlarm true
     print "Bug Alarm! newlyInfectedIHbeesInThisCohort > number_healthy!"

    ]

    set number_infectedAsAdult number_infectedAsAdult + newlyInfectedIHbeesInThisCohort
    set number_healthy  number_healthy - newlyInfectedIHbeesInThisCohort

    if number_healthy < 0
    [
     set BugAlarm true
     type "BUG ALARM!!! (MitePhoreticPhaseProc) Negative number of healthy IH bees (MitePhoreticPhaseProc): "
     show number_healthy
    ]

    if number_healthy + number_infectedAsPupa + number_infectedAsAdult != number
    [
     set BugAlarm true
     type "BUG ALARM!!! (MitePhoreticPhaseProc) Wrong sum of healthy + infected bees in this cohort: "
     type number_healthy + number_infectedAsPupa + number_infectedAsAdult
     type " instead of: "
     show number
    ]
  ]  ; end "if TotalIHbees > 0 and number > 0 "
 ] ; end "ask IHbeeCohorts "

 set infectedPhoreticMites infectedPhoreticMites + newlyInfectedMites
 set healthyPhoreticMites healthyPhoreticMites - newlyInfectedMites

 if healthyPhoreticMites < 0
 [
  set BugAlarm true
```

```
   type "BUG ALARM!!! Negative number of healthy mites (MitePhoreticPhaseProc): "
   show healthyPhoreticMites
  ]

  if infectedPhoreticMites + healthyPhoreticMites > 0
  [
   set PhoreticMitesHealthyRate
     healthyPhoreticMites / (infectedPhoreticMites + healthyPhoreticMites)
  ]

end

; ****************************************************************************************************************


to MiteOrganisersUpdateProc
  set TotalMites 0
   ; all mites in the colony, irrespective if phoretic or in cells

  ask miteOrganisers
  [
   back 1 ; new position in the GUI
   set age age + 1
   set cohortInvadedMitesSum 0
   let counter 0
     ; counts total numbers of mites in brood cells for each miteOrganiser (="mite cohort")

   foreach workerCellListCondensed
   [
     set cohortInvadedMitesSum cohortInvadedMitesSum + (? * counter)
     set counter counter + 1
   ] ; sums up the mites in worker cells ( multiplication of # cells with X mites in them * X) (X = counter)
```

```
    set counter 0

    foreach droneCellListCondensed
    [
      set cohortInvadedMitesSum cohortInvadedMitesSum
          + (? * counter)
      set counter counter + 1
    ] ; sums up the mites in drone cells ( multiplication of # cells with X mites in them * X) (X = counter)

    set label cohortInvadedMitesSum
    set TotalMites TotalMites + cohortInvadedMitesSum
      ; interim result: summing up all the mites in the cells

    if (age > DRONE_EMERGING_AGE) and (age >= EMERGING_AGE)
    [
      die
    ]  ; ">" (not ">=") as they age at the beginning of this procedure
  ] ; end "ask miteOrganisers "

 set TotalMites TotalMites + PhoreticMites
   ; final result: TotalMites = all mites in the cells + phoretic mites
end

; ****************************************************************************

; ...............  END OF THE VARROA MITE SUBMODEL   ..................................................

****************************************************************************
```

```
to CountingProc
 ; counts # bees in different stages, castes CALLED BY: 1. BroodCareProc 2. Go 3. MiteProcedure

 ; WORKERS:
 set TotalEggs 0 ask eggCohorts [ set TotalEggs (TotalEggs + number)]
 set TotalLarvae 0 ask larvaeCohorts [ set TotalLarvae (TotalLarvae + number)]
 set TotalPupae 0 ask pupaeCohorts [ set TotalPupae (TotalPupae + number)]
 set TotalIHbees 0 ask IHbeeCohorts [ set TotalIHbees (TotalIHbees + number)]
 set TotalForagers (count foragerSquadrons) * SQUADRON_SIZE

 ; DRONES:
 set TotalDroneEggs 0 ask DroneEggCohorts [ set TotalDroneEggs (TotalDroneEggs + number)]
 set TotalDroneLarvae 0 ask DroneLarvaeCohorts [ set TotalDroneLarvae (TotalDroneLarvae + number)]
 set TotalDronePupae 0 ask DronePupaeCohorts [ set TotalDronePupae (TotalDronePupae + number)]
 set TotalDrones 0 ask DroneCohorts [ set TotalDrones (TotalDrones + number)]
 set TotalWorkerAndDroneBrood TotalEggs + TotalLarvae + TotalPupae + TotalDroneEggs + TotalDroneLarvae + TotalDronePupae
 if TotalEggs  < 0 OR TotalLarvae < 0 OR TotalPupae < 0 OR TotalIHbees < 0 OR TotalForagers < 0
 [
  set BugAlarm true
  output-show (word ticks " BUG ALARM! negative number in total bees")
  type "TotalEggs: "
  type TotalEggs
  type " TotalLarvae: "
  type TotalLarvae
  type " TotalPupae: "
  type TotalPupae
  type " TotalIHbees: "
  type TotalIHbees
  type " TotalForagers: "
  print TotalForagers
 ]
```

```
ask turtles
[
  if number < 0
  [
    set BugAlarm true
    type (word ticks " BUG ALARM! negative number in turtles: ")
    show number
    ]
]

if TotalMites < 0 or PhoreticMites < 0 or PhoreticMitesHealthyRate > 1  or PhoreticMitesHealthyRate < 0
[
  set BugAlarm true
  output-show (word ticks " BUG ALARM! Check number of mites and PhoreticMitesHealthyRate!")
  type "PhoreticMitesHealthyRate: "
  type PhoreticMitesHealthyRate
  type " TotalMites: "
  type TotalMites
  type " PhoreticMites: "
  type PhoreticMites
]

ask (turtle-set pupaeCohorts dronePupaeCohorts droneCohorts)
[
  if number != number_infectedAsPupa + number_healthy
  [
    set BugAlarm true
    show "BUG ALARM! (CountingProc) number <> healthy + infected"
  ]
]

ask IHbeeCohorts
```

```
  [
    if number != number_infectedAsAdult + number_infectedAsPupa + number_healthy
    [
      set BugAlarm true
      show "BUG ALARM! (CountingProc) number <> healthy + infected (IH-bees)"
    ]
  ]
end

; ****************************************************************************************************************************


to PollenConsumptionProc
  ; calculates the daily pollen consumption

  let DAILY_POLLEN_NEED_ADULT 1.5 ; 0 ;1.5 ; 1.5 ;
    ; 1.5 mg fresh pollen per Day per bee (based on
    ; Pernal, Currie 2000, value for 14d old bees, Fig. 3)

  let DAILY_POLLEN_NEED_ADULT_DRONE 2 ; just an ESTIMATION

  let DAILY_POLLEN_NEED_LARVA 142 / (PUPATION_AGE - HATCHING_AGE)
    ; (23.6 mg/d) see HoPoMo
  let DAILY_POLLEN_NEED_DRONE_LARVA 50
    ; ESTIMATION, Rortais et al. 2005: "The pollen consumption of drone larvae has never been determined."

  let pollenStoreLasting_d 7
    ; similar to "FACTORpollenstorage" of HoPoMo model, which is set to 6.
    ; Seeley 1995: pollen stores last for about 1 week;

  let needPollenAdult
    ((TotalIHbees + TotalForagers) *  DAILY_POLLEN_NEED_ADULT
```

```
    + TotalDrones * DAILY_POLLEN_NEED_ADULT_DRONE )

  let needPollenLarvae (TotalLarvae * DAILY_POLLEN_NEED_LARVA
    + TotalDroneLarvae * DAILY_POLLEN_NEED_DRONE_LARVA )

  set DailyPollenConsumption_g (needPollenAdult + needPollenLarvae) / 1000 ; [g]
  set PollenStore_g PollenStore_g - DailyPollenConsumption_g
  if PollenStore_g < 0
  [
   set PollenStore_g 0
  ]

  ; the amount of pollen a colony tries to keep (depends on its current pollen consumption):
  set IdealPollenStore_g DailyPollenConsumption_g * pollenStoreLasting_d ; [g]

  if IdealPollenStore_g < MIN_IDEAL_POLLEN_STORE
  [
   set IdealPollenStore_g MIN_IDEAL_POLLEN_STORE
  ]

  ; PollenIdeal: switch in GUI, if true: pollen stores are always "ideal":
  if PollenIdeal = true
  [
   set PollenStore_g IdealPollenStore_g
  ]

  ; if no more pollen is left, protein stores of nurse bees are reduced.
  ;Assumption: protein stores of nurses can last for 7d, if the max. amount of brood (rel. to # nurses) is present, or proportionally longer if less brood
is present:
  let workloadNurses 0
  if (TotalIHbees + TotalForagers * FORAGER_NURSING_CONTRIBUTION) * MAX_BROOD_NURSE_RATIO > 0
  [
```

```
    set workloadNurses
      TotalWorkerAndDroneBrood /
        ((TotalIHbees + TotalForagers * FORAGER_NURSING_CONTRIBUTION) * MAX_BROOD_NURSE_RATIO)
  ]

  ifelse PollenStore_g > 0
    [
      set ProteinFactorNurses ProteinFactorNurses + (1 / PROTEIN_STORE_NURSES_d)
    ] ;  IF pollen in present in colony, nurses can restore the protein stores of
      ; their bodies (within 7d)
    [
      set ProteinFactorNurses ProteinFactorNurses - (workloadNurses / PROTEIN_STORE_NURSES_d)
    ] ; ELSE protein content of brood food decreases, depending on brood to nurse ratio

  if ProteinFactorNurses > 1 [ set ProteinFactorNurses 1 ]
    ; range of ProteinFactorNurses between 1..

  if ProteinFactorNurses < 0 [ set ProteinFactorNurses 0 ]  ; .. and 0
end

; ************************************************************************************************************


to HoneyConsumptionProc

  let DAILY_HONEY_NEED_ADULT_RESTING 11 ; 15 ; (11)
    ; [mg/Day of honey] Rortais et al 2005: Winter bees: 11 mg/d (based on
    ; assumptions from Winston, 1987)

  let DAILY_HONEY_NEED_NURSES 53.42  ; (53.42) [mg/Day of honey]
    ; Rortais et al 2005: average for "brood attending" 34-50mg sugar/d => 43-64mg/d honey

  let THERMOREGULATION_BROOD (DAILY_HONEY_NEED_NURSES - DAILY_HONEY_NEED_ADULT_RESTING)
```

```
  / MAX_BROOD_NURSE_RATIO
  ;  additional cost per broodcell (e.g. Thermoregulation): difference between nursing
  ; and resting divided by # broodcells;

let DAILY_HONEY_NEED_LARVA 65.4 / (PUPATION_AGE - HATCHING_AGE) ; [mg/day]
  ; = 10.9[mg] HONEY per Day per larvae  = 163.5mg nectar in total * 0.4
  ; (0.4: Nectar to honey); HoPoMo =  65.4 mg / 6

let DAILY_HONEY_NEED_DRONE_LARVA 19.2 ;
  ; [mg/Day of honey] Rortais et al 2005: 98.2mg sugar in 6.5d
  ; sugar to honey: x1.272 i.e. 124.9mg honey in total or 19.2 mg/d

let DAILY_HONEY_NEED_ADULT_DRONE 10  ;
  ; (9.806 = 10mg honey per day): Winston p62: resting drone 1-3mg sugar/hr
  ; flying drone: 14mg/hr (Mindt 1962); assumptions: 22h resting, 2h flying (MB);
  ; 1 mg sucrose = 17J; 1kJ = 0.008013g Honig

; honey costs of all adults, in-hive bees, foragers and drones:
let needHoneyAdult
    (TotalIHbees + TotalForagers) * DAILY_HONEY_NEED_ADULT_RESTING
    + TotalDrones * DAILY_HONEY_NEED_ADULT_DRONE

let needHoneyLarvae
    TotalLarvae * DAILY_HONEY_NEED_LARVA
     + TotalDroneLarvae * DAILY_HONEY_NEED_DRONE_LARVA

set DailyHoneyConsumption
   needHoneyAdult + needHoneyLarvae + TotalWorkerAndDroneBrood
    * THERMOREGULATION_BROOD  ; [mg]

; the honey consumption is removed from the honey stores:
set HoneyEnergyStore
```

```
     HoneyEnergyStore
     - (DailyHoneyConsumption / 1000) * ENERGY_HONEY_per_g

  ; sum up the total honey consumption as potential output:
  set CumulativeHoneyConsumption
    CumulativeHoneyConsumption  + DailyHoneyConsumption  ;[mg]

  ; HoneyIdeal: switch in GUI, if true: honey stores are always full:
  if HoneyIdeal = true
  [
   set HoneyEnergyStore MAX_HONEY_ENERGY_STORE
  ]
end

; ****************************************************************************************************


to BeekeepingProc

  let winterPauseStart 320 ; 320 = mid November
  let winterPauseStop 45 ; 45 = mid February
  let minWinterStore_kg 16 ; [kg] honey
  let minSummerStore_kg 3   ; [kg]
  let addedFondant_kg 1 ; [kg]
  let addedPollen_kg 0.5  ; [kg]


  ; feeding of colony
  ask Signs with [shape = "ambrosia"] [ hide-turtle]

  if FeedBees = true
    and day < winterPauseStart
    and day > winterPauseStop
```

```
 and HoneyEnergyStore / ( ENERGY_HONEY_per_g * 1000 ) < minSummerStore_kg
   ; feeding colony in spring or summer
[
  set TotalHoneyFed_kg TotalHoneyFed_kg + addedFondant_kg
  set HoneyEnergyStore HoneyEnergyStore + (addedFondant_kg * ENERGY_HONEY_per_g * 1000)
  output-type "Feeding colony on day "
  output-type ceiling (day mod 30.4374999) ; day
  output-type "."
  output-type floor(day / (365.25 / 12)) + 1 ; month
  output-type "."
  output-type ceiling (ticks / 365)     ; year
  output-type " Fondant provided [kg]: "
  output-type precision addedFondant_kg 1
  output-type " total food added [kg]: "
  output-print precision TotalHoneyFed_kg 1
  ask Signs with [shape = "ambrosia"] [ show-turtle]
]

if FeedBees = true
  and day = winterPauseStart
  and HoneyEnergyStore / ( ENERGY_HONEY_per_g * 1000 ) < minWinterStore_kg
   ; feeding colony before winter
[
  set TotalHoneyFed_kg TotalHoneyFed_kg
    + minWinterStore_kg
    -(HoneyEnergyStore / ( ENERGY_HONEY_per_g * 1000 ))

  output-type "Feeding colony on day "
  output-type day
  output-type ". Ambrosia fed [kg]: "
  output-type precision (minWinterStore_kg - (HoneyEnergyStore / ( ENERGY_HONEY_per_g * 1000 ))) 1
  output-type " total food added [kg]: "
```

```
  output-print precision TotalHoneyFed_kg 1
  set HoneyEnergyStore minWinterStore_kg * 1000 * ENERGY_HONEY_per_g
    ; if honey store is smaller than minWinterStore it is filled up to minWinterStore

  ask Signs with [shape = "ambrosia"] [ show-turtle]
 ]


 ; add bees to weak colony
; a weak colony is "merged" with another
 ; (not modelled!) weak colony (all of them are healthy):
 ask signs with [shape = "colonies_merged"] [ hide-turtle ]
 if MergeWeakColonies = true
  and (TotalIHbees + TotalForagers) < MergeColoniesTH
  and day = winterPauseStart
 [
  set TotalBeesAdded TotalBeesAdded + MergeColoniesTH
  output-type "Merging colonies in autumn! "
  output-type " # added bees: "
  output-type MergeColoniesTH
  output-type " total bees added: "
  output-print TotalBeesAdded
  ask signs with [shape = "colonies_merged"] [ show-turtle ]

  create-foragerSquadrons (MergeColoniesTH / SQUADRON_SIZE)
  [
   set age 60 + random 40
   setxy 30 9
   set color grey
   set size 2
   set heading 90
   set shape "bee_mb_1"
   set mileometer random (MAX_TOTAL_KM / 5)
```

```
    set activity "resting"
    set activityList [ ]
    set cropEnergyLoad 0 ; [kJ] no nectar in the crop yet
    set collectedPollen 0 ; [g] no pollen pellets
    set knownNectarPatch -1  ; -1 = no nectar Flower patch known
    set knownPollenPatch -1 ; -1 = no pollen Flower patch known
    set pollenForager false ; new foragers are nectar foragers
    set infectionState "healthy"
     ; possible infection states are: "healthy" "infectedAsPupa" "infectedAsAdult"
  ]
] ; if MergeWeakColonies = true  ...

; add pollen in spring
ask signs with [shape = "pollengrain"] [ hide-turtle ]
if AddPollen = true and day = 90 ; day 90: end of March
[
  ask signs with [shape = "pollengrain"] [ show-turtle ]
  set TotalPollenAdded TotalPollenAdded + addedPollen_kg
  output-type "Added pollen [kg]: "
  output-type addedPollen_kg
  output-type " total pollen added [kg]: "
  output-print TotalPollenAdded
  set PollenStore_g PollenStore_g + addedPollen_kg * 1000
]

; honey harvest
ask Signs with [shape = "honeyjar"] [ hide-turtle ]
if ((Day >=  HarvestingDay)
  and (Day <  HarvestingDay + HarvestingPeriod)
  and (HoneyHarvesting = true))
    ; honey can only be harvested within HarvestingPeriod
```

```
[
  if HoneyEnergyStore / ( ENERGY_HONEY_per_g * 1000 ) > HarvestingTH
  [
    set HarvestedHoney_kg (HoneyEnergyStore  / (ENERGY_HONEY_per_g * 1000)) - RemainingHoney_kg
    set HoneyEnergyStore HoneyEnergyStore - (HarvestedHoney_kg * ENERGY_HONEY_per_g * 1000)
    set TotalHoneyHarvested_kg TotalHoneyHarvested_kg + HarvestedHoney_kg
    output-type "Honey harvest on day "
    output-type ceiling (day mod 30.4374999)
    output-type "."
    output-type floor(day / (365.25 / 12)) + 1
    output-type "."
    output-type ceiling (ticks / 365)
    output-type ". Amount [kg]: "
    output-type precision HarvestedHoney_kg 1
    output-type " total honey harvested: "
    output-print precision TotalHoneyHarvested_kg 1
    ask Signs with [shape = "honeyjar"]
    [
      show-turtle
      set label precision HarvestedHoney_kg 1
    ]
  ]
]


; requeening
if QueenAgeing = true
[
  let requeening true ; true
  if requeening = true and Queenage >= 375
  [
    set Queenage 10
    output-print word "New queen inserted on day " day
```

```
   ] ; old queen is replaced by the beekeeper
  ]


 ; varroa treatment
 let treatmentDay 270   ; 270: 27.September
 let treatmentDuration 40  ; (28-40d) Fries et al. 1994
 let treatmentEfficiency 0.115
   ; (0.115) Fries et al. 1994 kills X*100% of phoretic mites each treatment Day

 ifelse ((varroaTreatment = true) and (Day >= treatmentDay)
   and (Day <=  treatmentDay + treatmentDuration )
   and (N_INITIAL_MITES_HEALTHY + N_INITIAL_MITES_INFECTED > 0))
   [
     set PhoreticMites round(PhoreticMites * (1 - treatmentEfficiency))
     ask signs with [shape = "x" or shape = "varroamite03"] [ show-turtle]
   ]
   [
     ask signs with [shape = "x" or shape = "varroamite03"] [ hide-turtle]
   ]
end

; ****************************************************************************
;.....................           PLOT PROCEDURES            ..................................................................
; ****************************************************************************


to DoPlotsProc

; CAUTION: choosing "age forager squadrons", "mileometer" or "mean total km per day" will affect
; the sequence of random numbers!
; with-local-randomness [  ; to run the procedure is run without affecting subsequent random events
 if showAllPlots = true [ DrawForagingMapProc ]
```

```
ask Signs with [ shape = "arrow" ]
[
  facexy (xcor + 1000000) (ycor + (HoneyEnergyStore - HoneyEnergyStoreYesterday)
     / ( ENERGY_HONEY_per_g / 1000))

  set label  word "H: " precision ((HoneyEnergyStore - HoneyEnergyStoreYesterday)
      / ( ENERGY_HONEY_per_g * 1000 )) 2

  ifelse (HoneyEnergyStore - HoneyEnergyStoreYesterday)
    / ( ENERGY_HONEY_per_g * 1000 ) >= 0
    [ set color green ]
    [ set color red ]
]

ask Signs with [ shape = "arrowpollen" ]
[
  facexy (xcor - 100) (ycor + (PollenStore_g - PollenStore_g_Yesterday))
  set label word "P: " precision ((PollenStore_g - PollenStore_g_Yesterday) / 1000) 2
  ifelse (PollenStore_g - PollenStore_g_Yesterday) > 0
    [ set color green ]
    [ set color red ]
]

ask Signs with [shape = "pete"]
[
  ifelse VarroaTreatment = true
         or FeedBees = true
         or HoneyHarvesting = true
         or AddPollen
         or MergeWeakColonies = TRUE
    [ show-turtle]
```

```
      [ hide-turtle ]
  ]

  ; calling GenericPlottingProc (8x) with plotname & plotChoice as input:
  GenericPlottingProc "Generic plot 1" GenericPlot1
  GenericPlottingProc "Generic plot 2" GenericPlot2
  GenericPlottingProc "Generic plot 3" GenericPlot3
  GenericPlottingProc "Generic plot 4" GenericPlot4
  GenericPlottingProc "Generic plot 5" GenericPlot5
  GenericPlottingProc "Generic plot 6" GenericPlot6
  GenericPlottingProc "Generic plot 7" GenericPlot7
  GenericPlottingProc "Generic plot 8" GenericPlot8
; ] ; end "with-local-randomness"
end

; ********************************************************************************************************************


to GenericPlotClearProc
  ; clear those plots, that only show output of 'today'

  let i 1
  while [ i <= N_GENERIC_PLOTS ]

  [
    let plotname (word "Generic plot " i)
      ; e.g. "Generic plot 1"
     if (i = 1 and (GenericPlot1 = "foragers today [%]" or GenericPlot1 = "active foragers today [%]"))
     or (i = 2 and (GenericPlot2 = "foragers today [%]" or GenericPlot2 = "active foragers today [%]"))
     or (i = 3 and (GenericPlot3 = "foragers today [%]" or GenericPlot3 = "active foragers today [%]"))
     or (i = 4 and (GenericPlot4 = "foragers today [%]" or GenericPlot4 = "active foragers today [%]"))
     or (i = 5 and (GenericPlot5 = "foragers today [%]" or GenericPlot5 = "active foragers today [%]"))
```

```
    or (i = 6 and (GenericPlot6 = "foragers today [%]" or GenericPlot6 = "active foragers today [%]"))
    or (i = 7 and (GenericPlot7 = "foragers today [%]" or GenericPlot7 = "active foragers today [%]"))
    or (i = 8 and (GenericPlot8 = "foragers today [%]" or GenericPlot8 = "active foragers today [%]"))
      [
        set-current-plot plotname
        clear-plot
      ]
    set i i + 1
  ]
end

; ****************************************************************************************************

to GenericPlottingProc [ plotname plotChoice ]
 set TotalEventsToday NectarFlightsToday + PollenFlightsToday + EmptyFlightsToday
 set-current-plot plotname

 set TotalWeightBees_kg
   ( TotalEggs * 0.0001     ; 0.0001g (wegg, HoPoMo)
     + TotalLarvae * 0.0457
         ; 0.0457g : average weight of a larva (using wlarva 1..5 from HoPoMo (p. 231)
     + TotalPupae * 0.16      ; 0.16g wpupa (HoPoMo)
     + (TotalIHbees + TotalForagers) * WEIGHT_WORKER_g      ; 0.1g wadult (HoPoMo)
     + TotalDroneEggs * 0.0001
     + TotalDrones * 0.22
         ; 0.22g (Rinderer, Collins, Pesante (1985), Apidologie)
     + TotalDroneLarvae *(0.1 * (0.22 / WEIGHT_WORKER_g))
        ; estimation of drone larva weight on basis of worker larva weight and
        ; adult worker:drone weight
        ; 0.10054 = 0.0457*2.2 = estimated drone larva weight
```

```
   + TotalDronePupae * (0.16 * (0.22 / WEIGHT_WORKER_g))
      ; estimation of drone pupa weight on basis of worker pupa weight and adult worker:drone weight
  ) / 1000 ; [g] -> [kg]

 if plotChoice = "colony weight [kg]" ; total weight of the colony without hive/supers etc.
 [
  create-temporary-plot-pen "weight"
  plot TotalWeightBees_kg ;
 ]

 if plotChoice = "foragingPeriod"
  [
   create-temporary-plot-pen "period"
   plotxy ticks DailyForagingPeriod / 3600
  ]
 if plotChoice = "# completed foraging trips (E-3)"
  [
    create-temporary-plot-pen "# trips"
    plotxy ticks totalEventsToday / 1000
  ]

if plotChoice = "trips per hour sunshine (E-3)"
  [
    create-temporary-plot-pen "trips/h"
    ifelse DailyForagingPeriod > 0
     [ plotxy ticks (TotalEventsToday / 1000) / (DailyForagingPeriod / 3600) ]
     [ plotxy ticks 0 ]
  ]

if plotChoice = "active foragers [%]"
  [
    create-temporary-plot-pen "active%"
```

```
    set-plot-y-range  0 100
    set-plot-pen-mode 1 ; 1: bars
    ifelse TotalForagers > 0
      [ plotxy ticks (100 * SQUADRON_SIZE
             * (count foragersquadrons with [km_today > 0])) / TotalForagers ]
      [ plotxy ticks 0 ]
  ]

if plotChoice = "mean trip duration"
  [
    create-temporary-plot-pen "trip [min]"
    set-plot-pen-mode 1 ; 1: bars
    ifelse ForagingRounds > 0
     [ plotxy ticks ( DailyForagingPeriod  / (ForagingRounds * 60)) ]
      ; mean Foraging trip duration [min] on this day
     [ plotxy ticks 0 ] ; if no foraging takes place
  ]

if plotChoice = "mean total km per day"
  [
    create-temporary-plot-pen "km/d"
    set-plot-pen-mode 0 ; 0: lines
    ifelse count foragerSquadrons > 0
      [ plotxy ticks mean [km_today] of foragerSquadrons ]
      [ plotxy ticks 0 ]
  ]

if plotChoice = "mileometer"
  [
    create-temporary-plot-pen "km"
    set-plot-x-range  0 850
    set-plot-y-range  0 40
```

```
    set-plot-pen-mode 1 ; 1: bars
    set-plot-pen-interval 25
    histogram [ mileometer ] of foragerSquadrons
  ]


if plotChoice = "loads returning foragers [%]"
  [
    set totalEventsToday NectarFlightsToday + PollenFlightsToday + EmptyFlightsToday
    ifelse totalEventsToday > 0
      [
        create-temporary-plot-pen "nectar"
        set-plot-pen-color yellow
        plotxy ticks (100 * NectarFlightsToday) / totalEventsToday
        create-temporary-plot-pen "pollen"
        set-plot-pen-color orange
        plotxy ticks (100 * PollenFlightsToday) / totalEventsToday
        create-temporary-plot-pen "empty"
        set-plot-pen-color cyan
        plotxy ticks (100 * EmptyFlightsToday) / totalEventsToday
      ]
      [
        create-temporary-plot-pen "nectar"
        set-plot-pen-color yellow
        plotxy ticks 0
        create-temporary-plot-pen "pollen"
        set-plot-pen-color orange
        plotxy ticks 0
        create-temporary-plot-pen "empty"
        set-plot-pen-color cyan
        plotxy ticks 0
      ]
```

```
  ]

 if plotChoice = "broodcare [%]"
  [
    set-plot-y-range 0 150
    create-temporary-plot-pen "Protein"
      set-plot-pen-color orange
      plot ( ProteinFactorNurses * 100 )  ; Proteinfactor of nurses [%]
    create-temporary-plot-pen "Workload"
     if ((TotalIHbees + TotalForagers * FORAGER_NURSING_CONTRIBUTION)
       * MAX_BROOD_NURSE_RATIO) > 0 ; avoids division by 0
     [
       plot ( 100 * (TotalWorkerAndDroneBrood / ((TotalIHbees + TotalForagers
            * FORAGER_NURSING_CONTRIBUTION) * MAX_BROOD_NURSE_RATIO)) )
     ]

    create-temporary-plot-pen "Pollen"
      set-plot-pen-color green
      plot (PollenStore_g / IdealPollenStore_g) * 100
  ]


if plotChoice = "consumption [g/day]"
  [
    create-temporary-plot-pen "honey"
      set-plot-pen-color yellow
      plot (DailyHoneyConsumption / 1000)  ;[g/day]

    create-temporary-plot-pen "pollen"
      set-plot-pen-color orange
      plot (DailyPollenConsumption_g)   ;[g/day]
  ]
```

```
if plotChoice = "drones"
  [
    create-temporary-plot-pen "Eggs"  ; DRONE eggs
      set-plot-pen-color blue
      plot (TotalDroneEggs)
    create-temporary-plot-pen "Larvae"  ;DRONE larvae
      set-plot-pen-color yellow
      plot (TotalDroneLarvae)
    create-temporary-plot-pen "Pupae"   ; DRONE pupae
      set-plot-pen-color brown
      plot (TotalDronePupae)
    create-temporary-plot-pen "Drones"
      plot (TotalDrones)
  ]


if plotChoice = "colony structure workers"
  [
    create-temporary-plot-pen "Eggs"
      set-plot-pen-color blue
      plot (TotalEggs)
    create-temporary-plot-pen "Larvae"
      set-plot-pen-color yellow
      plot (TotalLarvae)
    create-temporary-plot-pen "Pupae"
      set-plot-pen-color brown
      plot (TotalPupae)
    create-temporary-plot-pen "IHbees"
      set-plot-pen-color orange
```

```
      plot (TotalIHbees)
    create-temporary-plot-pen "Foragers"
      set-plot-pen-color green
      plot (TotalForagers)
    create-temporary-plot-pen "Adults"
      set-plot-pen-color black
      plot (TotalForagers + TotalIHbees)
    create-temporary-plot-pen "Brood"
      set-plot-pen-color violet
      plot (TotalEggs + TotalLarvae + TotalPupae)
  ]



 let totalNectarAvailableToDay 0
 let totalPollenAvailableToDay 0
 ask flowerPatches
 [
   set totalNectarAvailableToDay totalNectarAvailableToDay + quantityMyl
   set totalPollenAvailableToDay totalPollenAvailableToDay + amountPollen_g
 ]

if plotChoice = "nectar availability [l]"
 [
   ifelse readInfile = false
     [
       create-temporary-plot-pen "Patch 0"
         set-plot-pen-color red
         plot (([ quantityMyl ] of flowerPatch 0 ) / 1000000 )  ;[l] nectar
       create-temporary-plot-pen "Patch 1"
         set-plot-pen-color green
```

```
        plot (([ quantityMyl ] of flowerPatch 1 ) / 1000000 )  ;[l] nectar
      ]
      [
        create-temporary-plot-pen "all patches"
        set-plot-pen-color yellow ; black
        plot (totalNectarAvailableToDay / 1000000 )  ;[l] nectar
      ]
  ]


  if plotChoice =  "pollen availability [kg]"
  [
   ifelse readInfile = false
      [
        create-temporary-plot-pen "Patch 0"
          set-plot-pen-color red
          plot (([ amountPollen_g ] of flowerPatch 0 ) / 1000 )  ; [kg] pollen
        create-temporary-plot-pen "Patch 1"
          set-plot-pen-color green
          plot (([ amountPollen_g ] of flowerPatch 1 ) / 1000 )  ; [kg] pollen
      ]
      [
        create-temporary-plot-pen "all patches"
          set-plot-pen-color orange; black
          plot (totalPollenAvailableToDay / 1000 )  ; [kg] pollen
      ]
  ]

 if plotChoice = "egg laying"
 [
  create-temporary-plot-pen "new eggs"
  plot (NewWorkerEggs)
```

```
]

 if plotChoice = "honey gain [kg]"
   [
     set-plot-y-range -3 10
     create-temporary-plot-pen "gain"
     set-plot-pen-mode 1 ; 1: bars
     ifelse (HoneyEnergyStore - HoneyEnergyStoreYesterday) / ( ENERGY_HONEY_per_g * 1000 ) < 0
       [ set-plot-pen-color red ]
       [ set-plot-pen-color black ]
     plotxy ticks (HoneyEnergyStore - HoneyEnergyStoreYesterday) / ( ENERGY_HONEY_per_g * 1000 )
   ]

if plotChoice = "stores & hive [kg]"
[ create-temporary-plot-pen "honey"
    set-plot-pen-color yellow
    plot (HoneyEnergyStore / ( ENERGY_HONEY_per_g * 1000 ) )  ;[ml] honey
 ; create-temporary-plot-pen "decent honey"
 ;    set-plot-pen-color brown
 ;   plot (TotalIHbees + TotalForagers ) * 0.0015
    ;; 1.5g honey per bee = estimated honey necessary for the colony to survive the winter
  create-temporary-plot-pen "pollen x 20"
    set-plot-pen-color orange
    plot 20 * (PollenStore_g / 1000)  ;[kg * 10] pollen stored in the colony in kg
]

 if plotChoice = "mites"
 [
   create-temporary-plot-pen "totalMites"
     plot (TotalMites)  ; # all mites (phoretic & in cells)
   create-temporary-plot-pen "phoreticMites"
     set-plot-pen-color brown
```

```
    plot (PhoreticMites)  ; # phoretic mites
  create-temporary-plot-pen "phoreticMitesInfected"
    set-plot-pen-color red
    plot (PhoreticMites * (1 - PhoreticMitesHealthyRate))  ; # infected phoretic mites
  create-temporary-plot-pen "phoreticMitesHealthy"
    set-plot-pen-color green
    plot (PhoreticMites * PhoreticMitesHealthyRate)  ; # healthy phoretic mites
  create-temporary-plot-pen "miteDrop x 10"
    set-plot-pen-color violet
    plot (DailyMiteFall * 10)  ; # dropping mites
]

if plotChoice = "proportion infected mites"
[
  create-temporary-plot-pen "proportion"
  if TotalMites > 0 [ plotxy ticks (1 - PhoreticMitesHealthyRate) ]
]

if plotChoice = "aff & lifespan"
[
  create-temporary-plot-pen "aff"
    set-plot-y-range 0 200
    set-plot-pen-mode 1 ; 1: bars
    if count foragerSquadrons with [age = aff] > 0
       [ plotxy ticks (aff) ]
  create-temporary-plot-pen "lifespan"
    set-plot-pen-color green
    set-plot-pen-mode 2 ; 2: dots
    ifelse (DeathsAdultWorkers_t > 0)
      and ((SumLifeSpanAdultWorkers_t / deathsAdultWorkers_t) < MIN_AFF)
         [ plot-pen-down ]
         [ plot-pen-up  ]
```

```
    plot (SumLifeSpanAdultWorkers_t / (DeathsAdultWorkers_t + 0.0000001)) ; to avoid division by 0
  ]

  if plotChoice =  "age forager squadrons"
  [
   set-plot-y-range 0 10
   set-plot-x-range 0 300

   create-temporary-plot-pen "foragersHealthy"
    set-plot-pen-mode 1 ; 1: bars
    set-plot-pen-interval 1
    histogram [ age ] of foragerSquadrons
      with [ infectionState = "healthy" ]

   create-temporary-plot-pen "foragersDiseased"
    set-plot-pen-mode 1 ; 1: bars
    set-plot-pen-interval 1
    set-plot-pen-color red
    histogram [ age ] of foragerSquadrons
      with [ infectionState = "infectedAsPupa" ]
      ; infectedAsPupa = true or infectedAsAdult = true ]

   create-temporary-plot-pen "foragersCarrier"
    set-plot-pen-mode 1 ; 1: bars
    set-plot-pen-interval 1
    set-plot-pen-color blue
    histogram [ age ] of foragerSquadrons
      with [ infectionState = "infectedAsAdult" ]
  ]

end
```

```
; *******************************************************************************************************************

to DrawForagingMapProc
; CAUTION: choice of ForagingMap and DotDensity affects the sequence of random numbers!
; with-local-randomness [  ; procedure is run without affecting subsequent random events
  set-current-plot "foraging map"
  set-current-plot-pen "default"
  clear-plot
  let xplot 0
  let yplot 0
  ask flowerPatches
  [
    if ForagingMap = "Nectar foraging"
    [
      repeat nectarVisitsToday * DotDensity
      [
        let radius sqrt(size_sqm / pi)
          ; the (hypothetical) radius of the patch (assumed to be circular)

        set xplot (xcorMap - radius) + (random-float (2 * radius))
          ; x coordinate randomly chosen from centre +- radius

        let yRange sqrt((radius ^ 2) - ((xplot - xcorMap) ^ 2))
          ; calculate the range of possible y-coordinates for chosen x-coordinate,

        set yplot (ycorMap - yRange) + (random-float (2 * yRange))
          ; y coordinate randomly chosen from the range of possible values

        set-plot-pen-color yellow
        plotxy xplot yplot
```

```
  ]
]

if ForagingMap = "Pollen foraging"
[
  repeat pollenVisitsToday * DotDensity
  [
    let radius sqrt(size_sqm / pi)
    ; the (hypothetical) radius of the patch (assumed to be circular)

    set xplot (xcorMap - radius) + (random-float (2 * radius))
      ; x coordinate randomly chosen from centre +- radius

    let yRange sqrt((radius ^ 2) - ((xplot - xcorMap) ^ 2))
      ; calculate the range of possible y-coordinates for chosen x-coordinate,

    set yplot (ycorMap - yRange) + (random-float (2 * yRange))
      ; y coordinate randomly chosen from the range of possible values )

    set-plot-pen-color orange
    plotxy xplot yplot
  ]
]

if ForagingMap = "All visits"
[
  repeat (nectarVisitsToday + pollenVisitsToday) * DotDensity
  [
    let radius sqrt(size_sqm / pi)
      ; the (hypothetical) radius of the patch (assumed to be circular)

    set xplot (xcorMap - radius) + (random-float (2 * radius))
```

```
      ; x coordinate randomly chosen from centre +- radius

    let yRange sqrt((radius ^ 2) - ((xplot - xcorMap) ^ 2))
      ; calculate the range of possible y-coordinates for chosen x-coordinate,

    set yplot (ycorMap - yRange) + (random-float (2 * yRange))
      ; y coordinate randomly chosen from the range of possible values

    set-plot-pen-color black
    plotxy xplot yplot
  ]
]

 if ForagingMap = "All patches"
 [
  repeat 10000 * DotDensity
  [
    let radius sqrt(size_sqm / pi)
      ; the (hypothetical) radius of the patch (assumed to be circular)

    set xplot (xcorMap - radius) + (random-float (2 * radius))
      ; x coordinate randomly chosen from centre +- radius

    let yRange sqrt((radius ^ 2) - ((xplot - xcorMap) ^ 2))
      ; calculate the range of possible y-coordinates for chosen x-coordinate,

    set yplot (ycorMap - yRange) + (random-float (2 * yRange))
      ; y coordinate randomly chosen from the range of possible values

    if patchType = "YellowField"
       or patchType = "OilSeedRape"
    [
```

```
      set-plot-pen-color yellow
    ]
    if patchType = "RedField" [ set-plot-pen-color red ]
    if patchType = "BlueField" [ set-plot-pen-color blue ]
    if patchType = "GreenField" [ set-plot-pen-color green ]
    plotxy xplot yplot
  ]
]

if ForagingMap = "Available patches"
[
  let proportionPollen 0
  let pollenAvailable amountPollen_g / POLLENLOAD
    ; # pollen loads available

  let nectarAvailable quantityMyl / CROPVOLUME
    ; # crop loads available

  if pollenAvailable + nectarAvailable > 0
  [
    set proportionPollen pollenAvailable / (pollenAvailable + nectarAvailable)
  ]

  repeat round sqrt((pollenAvailable + nectarAvailable) * DotDensity)
    ; sqrt to avoid too many repeats
  [
    let radius sqrt(size_sqm / pi)
      ; the (hypothetical) radius of the patch (assumed to be circular)

    set xplot (xcorMap - radius) + (random-float (2 * radius))
      ; x coordinate randomly chosen from centre +- radius
```

```
    let yRange sqrt((radius ^ 2) - ((xplot - xcorMap) ^ 2))
      ; calculate the range of possible y-coordinates for chosen x-coordinate,

    set yplot (ycorMap - yRange) + (random-float (2 * yRange))
      ; y coordinate randomly chosen from the range of possible values

    ifelse random-float 1 < proportionPollen
      [ set-plot-pen-color orange ]
      [ set-plot-pen-color yellow ]
    plotxy xplot yplot
  ]
]

if ForagingMap = "Nectar and Pollen"
[
  let proportionPollen 0
  if pollenVisitsToday + nectarVisitsToday > 0
  [
    set proportionPollen pollenVisitsToday
     / ( pollenVisitsToday
       + nectarVisitsToday )
  ]

  repeat (pollenVisitsToday + nectarVisitsToday) * DotDensity
  [
    let radius sqrt(size_sqm / pi)
      ; the (hypothetical) radius of the patch (assumed to be circular)

    set xplot (xcorMap - radius) + (random-float (2 * radius))
      ; x coordinate randomly chosen from centre +- radius

    let yRange sqrt((radius ^ 2) - ((xplot - xcorMap) ^ 2))
```

```
        set yplot (ycorMap - yRange) + (random-float (2 * yRange))
        ifelse random-float 1 < proportionPollen
          [ set-plot-pen-color orange ]
          [ set-plot-pen-color yellow ]
        plotxy xplot yplot
      ]
    ]
   ]  ; end of: "Ask flowerpatches"

  set-plot-pen-color brown  ; draw the colony:
  repeat 10000
  [
    plotxy (-50 + random 100) (-50 + random 100)
  ]
; ]   ; end "local randomness"
end

; ****************************************************************************************************************************************

to WriteToFileProc
  ; writes data in file, copied from: Netlogo: Library:
  ; Code Examples: "File Output Example"

  let year ceiling (ticks / 365)
  foreach sort flowerPatches
  [
    ask ?
    [
      file-print
        ( word year " " word ticks " " ForagingRounds " " word self
          " distance: " distanceToColony
```

```
        " concentration: " nectarConcFlowerPatch
        " EEF: " EEF
        " quantity: " quantityMyl)
  ]
 ]

 foreach sort foragerSquadrons
 [
  ask ?
  [
   file-print
     (word year " " word ticks " " ForagingRounds " "  word self
      " age: " age
      " km: " mileometer)
  ]
 ]

end

; ****************************************************************************************************************

to ReadFileProc
 ; reads data in from file, copied from: Netlogo: Library:
 ; Code Examples: "File Input Example"

 ifelse ( file-exists? INPUT_FILE )
  ; We check to make sure the file exists first
  [
   set AllDaysAllPatchesList []
    ; IF: data are saved in a list (list still empty)
```

```
    file-open INPUT_FILE
    let dustbin file-read-line
      ; first line of input file with headings is read - but not used for anything

    while [ not file-at-end? ]
    [
      set AllDaysAllPatchesList sentence AllDaysAllPatchesList
         (list (list file-read file-read file-read file-read file-read
                     file-read file-read file-read file-read file-read
                     file-read file-read file-read file-read file-read))]
         ; 15 data colums are read in
      file-close  ; closes file
      set N_FLOWERPATCHES ((length AllDaysAllPatchesList) / 365)
      if (N_FLOWERPATCHES mod 1) != 0
      [
        user-message "Error in Infile - wrong number of lines"
        set BugAlarm true
      ]
   ] ; end "ifelse"
   [
    user-message "There is no such file in current directory!"
   ]
end

;***************************************************************************************************************
; ***   END   *********   END   *********   END   *********   END   *********   END      *************************************
;***************************************************************************************************************
```

9. GLOBAL VARIABLES

VARIABLE (CONSTANTS are shown in capitals)	Unit	Default or initial value	References & comments	Explanation
ABANDON_POLLEN_PATCH_PROB_PER_S	1/s	0.00002		probability of experienced (but not necessarily active) pollen foragers to abandon their current pollen patch
AddPollen		FALSE		to improve colony growth in spring
Aff	d	AFF_BASE		current age of first foraging
AFF_BASE	d	21	deGrandi-Hoffman et al. (1989): 21d	default value of Aff
AllDaysAllPatchesList		-		data of all flower patches for all days, read in from text file
AlwaysDance		FALSE		always 2 dance followers, irrespective of flower patch quality
BugAlarm		FALSE		used in assertions to stop run if set true
ColonyDied		FALSE		set true as result of winter mortality or if no bees left, stops run if true
ColonyTripDurationSum	s	-		sums up the duration of all nectar, pollen and empty foraging trips to calculate the duration of a foraging round
ColonyTripForagersSum	s	-		sums up the number of all nectar, pollen and empty foraging trips during a foraging round
CONC_G	mol/l	1.5	e.g. Seeley (1986) Fig. 2: sugar concentration of collected nectar: 0.5-2.5 mol/l	sucrose concentration in nectar of "green" patch
CONC_R	mol/l	1.5	e.g. Seeley (1986) Fig. 2: sugar concentration of collected nectar: 0.5-2.5 mol/l	sucrose concentration in nectar of "red" patch
ConstantHandlingTime		FALSE		if true, handling time does not increase with depletion of flower patch
CRITICAL_COLONY_SIZE_WINTER	bees	4000	Martin (2001): 4000 adult workers during winter (from Free & Spencer-Booth 1958, Harbo 1983)	threshold colony size for winter survival on julian day 365

CROPVOLUME	µl	50	Winston (1987), Nuñez (1966, 1970), Schmid-Hempel et al. (1985)	volume of a forager's crop, is completely filled at flower patch
CumulativeHoneyConsumption	mg	-		total honey consumption of colony since start of the simulation
DailyForagingPeriod	s	-		time per day, bees are allowed to forage
DailyHoneyConsumption	mg	-		total amount of honey consumed on current day
DailyMiteFall	mites	0		# mites falling from comb and dying on the day of their emergence from cells
DailyPollenConsumption_g	g	-		total amount of pollen consumed on current day
DANCE_INTERCEPT	dance circuits	0	(Seeley (1994): min. 0.6 (Tab.2))	to calculate # circuits a bee dances for a patch, depending o the patch quality (energetic efficiency)
DANCE_SLOPE	dance circuits	1.16	Seeley (1994): max. 1.16 (Tab. 2)	to calculate # circuits a bee dances for a patch, depending o the patch quality (energetic efficiency)
Day	julian day	-		current time step as Julian day (ordinal date)
DeathsAdultWorkers_t	bees	-		sums up all deaths of all adult bees during a day
DeathsForagingToday	bees	-		sums up all deaths of foragers during a day
DecentHoneyEnergyStore	kJ	N_INITIAL_BEES * 1.5 * ENERGY_HONEY_per_g		amount of honey a colony should have stored to overwinter
Details		TRUE		if true: results are recorded in output file after each foraging round
DETECT_PROB_G		0.2		probability, a searching foragerSquadron finds the "green" patch
DETECT_PROB_R		0.2		probability, a searching foragerSquadron finds the "red" patch
DISTANCE_G	m	500	e.g. Steffan-Dewenter & Kuhn (2003): foraging distance ca. 60 - 10000m, mean: 1500m	distance of the "green" patch to the colony
DISTANCE_R	m	1500	e.g. Steffan-Dewenter & Kuhn (2003): foraging distance ca. 60 - 10000m, mean: 1500m	distance of the "red" patch to the colony
DotDensity		0.01		affects # of visits shown in "foraging map" plot
DRONE_EGGLAYING_START	d	115	April 25 (Allen 1963: late April ..late August)	beginning of drone production

DRONE_EGGLAYING_STOP	d	240	August 28 (Allen 1963: late April ..late August)	end of drone production
DRONE_EGGS_PROPORTION		0.04	Wilkinson & Smith (2002): 0.04 (from Allen 1963, 1965)	proportion of drone eggs
DRONE_EMERGING_AGE	d	24	Winston (1987)	age at which adult drones emerge from pupae
DRONE_HATCHING_AGE	d	3	Jay (1963), Hrassnig & Crailsheim (2005)	age at which drone larvae hatch from eggs
DRONE_LIFESPAN	d	37	Fukuda, Ohtani (1977): 14d in summer; 32-42d in autumn, 37 = average autumn life span	maximum lifespan of an adult drone
DRONE_PUPATION_AGE	d	10	Winston (1987)	age of pupation for drone
EggLaying_IH		TRUE		if true: egg laying is affected by available nurse bees
EMERGING_AGE	d	21	Winston (1987) p.50	age at which adult workers emerge from pupae
EmptyFlightsToday	flights	-		sums up all empty foraging flights during a day
ENERGY_HONEY_per_g	kJ/g	12.78	USDA: 304kcal/100g (http://www.nal.usda.gov/fnic/foodcomp/search/)	energy content of 1g honey (also used for fondant or syrup)
ENERGY_SUCROSE	kJ/µmol	0.00582	wikipedia.org: sucrose: 342.3g/mol; 17kJ/g => 0.005819kJ/µmol	sucrose energy per molar concentration
ExcessBrood	bees	-		amount of brood that dies due to lack of nurses or pollen
Experiment		"none"		sets up colony to match specific empirical experiments
FeedBees		FALSE		if true: beekeeper may increase the colonies' honey store
FIND_DANCED_PATCH_PROB		0.5	ca. average of reported values: Seeley 1983: 0.21; Judd 1995: 0.25; references in Biesmeijer, deVries 2001: 0.95 (Oettingen-Spielberg 1949), 0.73 (Lindauer 1952)	probability for a recruit to find the advertised patch
FLIGHT_VELOCITY	m/s	6.5	derived from Seeley 1994, mean velocity during foraging flight see also Ribbands p127: 12.5-14.9mph (= 5.58-6.66m/s)	flight speed of a forager

FLIGHTCOSTS_PER_m	kJ/m	0.000006	Goller, Esch (1990): 0.000006531 kJ/m; Schmid-Hempl et al. (1985): 0.0334W	energy consumption on flight per meter
FORAGER_NURSING_CONTRIBUTION		0.2		contribution of foragers on brood care (i.e. foragers are 80% less efficient than inhive bees)
FORAGING_STOP_PROB		0.3		probability per foraging round that an active forager switches to "resting"
ForagingMap		"Nectar and Pollen"		specifies data that is shown in the "foraging map" plot, e.g. # nectar and pollen visits at each patch
ForagingRounds		-		counts foraging rounds on current day
ForagingSpontaneousProb		-		probability per foraging round for a resting bee to start foraging
HarvestedHoney_kg	kg	-		amount of honey gathered during a single honey harvest
HarvestingDay	julian day	135	May 15	first day of the year on which harvesting honey is possible
HarvestingPeriod	d	80		period of time during which harvesting honey is possible, starting on harvestingDay
HarvestingTH	kg	20		minimum honey store to actually harvest honey
HATCHING_AGE	d	3	Winston (1987) p.50	age at which worker larvae hatch from eggs
HONEY_STORE_INIT	g	0.5 * MAX_HONEY_STORE_kg * 1000		initial honey store
HoneyEnergyStore	kJ	(HONEY_STORE_INIT * ENERGY_HONEY_per_g)		honey store of the colony (recorded in energy not in weight)
HoneyEnergyStoreYesterday		-		saves yesterdays' energy (stored in the honey) to determine changes in the honey store
HoneyHarvesting		FALSE		if true: beekeeper harvests honey, if possible
HoneyIdeal		FALSE		if true: honey store is set to maximum every day
HoPoMo_seasont		-		seasonal factor from Schmickl&Crailsheim 2007, which affects egg-laying and food availability
IdealPollenStore_g	g	POLLEN_STORE_INIT		amount of pollen foragers are trying to store
InhivebeesDiedToday	bees	-		sums up all deaths of inhive bees during a day
INPUT_FILE		"Input_2-1_FoodFlow.txt"		contains data on food availability etc. of all flower patches for 365d

INVADING_DRONE_CELLS_AGE	d	DRONE_PUPATION_AGE - 2		age of drone larvae suitable for mite invasion
INVADING_WORKER_CELLS_AGE	d	PUPATION_AGE - 1		age of worker larvae suitable for mite invasion
InvadingMitesDroneCellsReal	mites	-		# mites that actually enter drone brood cells on that day
InvadingMitesDroneCellsTheo	mites	-		# mites that try to enter drone brood cells on that day
InvadingMitesWorkerCellsReal	mites	-		# mites that actually enter worker brood cells on that day
InvadingMitesWorkerCellsTheo	mites	-		# mites that try to enter worker brood cells on that day
LIFESPAN	d	290	Sakagami, Fukuda (1968): max. 290d	maximum lifespan of a worker bee
LostBroodToday	bees	0		counts amount of brood died in total due to lack of protein or nurse bees
LostBroodTotal	bees	0		counts amount of brood died today due to lack of protein or nurse bees
MAX_AFF	d	50	Winston (1987) p.92 (minimum AFF between 3 and 20 days, maximum AFF between 27 and 65 days)	maximum possible value age of first foraging (Aff)
MAX_BROOD_NURSE_RATIO		3	Free & Racey (1968): 3; Becher et al. (2010): 2.65	maximum amount of brood, nurse bees can care for, with nurse bees = totalIHbees + totalForagers * FORAGER_NURSING_CONTRIBUTION
MAX_BROODCELLS	cells	2000099	i.e. not limiting; alternative value: e.g. 20000	maximum brood space
MAX_DANCE_CIRCUITS	dance circuits	117	Seeley & Towne (1992): 117	maximum circuits a bee can perform per dance
MAX_EGG_LAYING	eggs / day	1600	Schmickl & Crailsheim (2007)	maximum egg laying rate per day
MAX_HONEY_ENERGY_STORE	kJ	MAX_HONEY_STORE_kg * ENERGY_HONEY_per_g * 1000		maximum amount honey that can be stores defined as energy not as weight)
MAX_HONEY_STORE_kg	kg	50		maximum amount honey that can be stored (defined as weight)
MAX_INVADED_MITES_DRONECELL	mites	4 ("Martin")	Martin (2001)	maximum number of mites allowed to invade a drone brood cell, depends on MiteReproductionModel
MAX_INVADED_MITES_WORKERCELL	mites	4 ("Martin")	Martin (2001)	maximum number of mites allowed to invade a worker brood cell, on MiteReproductionModel
MAX_km_PER_DAY	km	7299	i.e. not limiting; alternative: e.g. 72km (Neukirch (1982): mean flight distance per day: 59+-13.7 km)	maximum total distance a forager can fly on a single day (default value not limiting), if limiting: e.g. 72 km (= 2 * 36 km: Lindauer 1948, referring to Gontarski)

MAX_PROPORTION_POLLEN_FORAGERS		0.8	Lindauer (1952): 0.8	maximum proportion of pollen foragers
MAX_TOTAL_KM	km	800	Neukirch (1982): 838km max. flight performance in a foragers life (set to 800km in the model, as mortality acts only at end of time step)	maximum total distance a forager can fly during lifetime
MergeColoniesTH	bees	5000		beekeeper adds in autumn foragers to colonies smaller than this threshold, if MergeWeakColonies = true
MergeWeakColonies		FALSE		allows beekeeper to add forager to weak colonies in autumn
MIN_AFF	d	7	Winston (1987) p.92 (minimum AFF between 3 and 20 days, maximum AFF between 27 and 65 days)	minimum possible value age of first foraging (Aff)
MIN_IDEAL_POLLEN_STORE	g	250		minimum amount of pollen foragers are trying to store
MITE_FALL_DRONECELL	1/emergence	0.2	Martin (1998): 20%	probability that a mite emerging from a drone cell will fall from the comb and die
MITE_FALL_WORKERCELL	1/emergence	0.3	Martin (1998): 30%	probability that a mite emerging from a worker cell will fall from the comb and die
MITE_MORTALITY_BROODPERIOD	mortality/d	0.006	Martin (1998): 0.006; Fries et al. (1994) (Tab. 6): 0.006	mite mortality rate per day during brood period
MITE_MORTALITY_WINTER	mortality/d	0.002	Martin (1998): 0.002; Fries et al. (1994) (Tab. 6): 0.004	mite mortality rate per day if no brood is present
MiteReproductionModel		"Martin"		choice between different parameterisations for mite invasion and reproduction
ModelledInsteadCalcDetectProb		FALSE		choice between detection probabilities of flower patches either modelled in "BEEHAVE_Landscape" or calculated in "BEEHAVE_FoodFlow"
MORTALITY_DRONE_EGGS	mortality/d	0.064	Fukuda, Ohtani (1977): 100 eggs, 82 unsealed brood, 60 sealed brood and 56 adults	daily mortality rate of drone eggs
MORTALITY_DRONE_LARVAE	mortality/d	0.044	Fukuda, Ohtani (1977): 100 eggs, 82 unsealed brood, 60 sealed brood and 56 adults	daily mortality rate of drone larvae
MORTALITY_DRONE_PUPAE	mortality/d	0.005	Fukuda, Ohtani (1977): 100 eggs, 82 unsealed brood, 60 sealed brood and 56 adults	daily mortality rate of drone pupae
MORTALITY_DRONES	mortality/d	0.05	Fukuda Ohati (1977): Fig. 3, "summer", mean lifespan: 14d	daily mortality rate of healthy adult drones

MORTALITY_DRONES_INFECTED_AS_PUPAE	mortality/d	MORTALITY_INHIVE_INFECTED_AS_PUPA * (MORTALITY_DRONES / MORTALITY_INHIVE) (DWV, APV)		daily mortality rate of adult drones, infected as pupae (no infection of adult drones possible)
MORTALITY_EGGS	mortality/d	0.03	Schmickl & Crailsheim (2007): 0.03	daily mortality rate of worker eggs
MORTALITY_FOR_PER_SEC	mortality/s	0.00001	Visscher & Dukas (1997): 0.036 per hour foraging	mortality rate of foragers per second foraging
MORTALITY_INHIVE	mortality/d	0.004	derived from Martin (2001), Fig. 2b (non-infected, winter)	daily mortality rate of healthy in-hive bees and foragers
MORTALITY_INHIVE_INFECTED_AS_ADULT	mortality/d	MORTALITY_INHIVE (DVW); 0.2 (APV)		daily mortality rate of in-hive bees and foragers, infected as adults
MORTALITY_INHIVE_INFECTED_AS_PUPA	mortality/d	0.012 (DWV); 1 (APV)	derived from Martin (2001), Fig. 2b (infected, winter)	daily mortality rate of in-hive bees and foragers, infected as pupae
MORTALITY_LARVAE	mortality/d	0.01	Schmickl & Crailsheim (2007): 0.01	daily mortality rate of worker larvae
MORTALITY_PUPAE	mortality/d	0.001	Schmickl & Crailsheim (2007): 0.001	daily mortality rate of worker pupae
N_FLOWERPATCHES		(2)		# of all flower patches
N_GENERIC_PLOTS		8		# of "generic" plots shown on the interface
N_INITIAL_BEES	bees	10000		initial colony size (all foragers)
N_INITIAL_MITES_HEALTHY	mites	0		initial number of healthy mites
N_INITIAL_MITES_INFECTED	mites	0		initial number of infected mites
NectarFlightsToday	flights	-		sums up all nectar foraging flights during a day
NewDroneEggs	bees	-		number of drone eggs laid today
NewDroneLarvae	bees	-		number of drone eggs that developed into larvae today
NewDronePupae	bees	-		number of drone larvae that developed into pupae today
NewDrones	bees	-		number of drone pupae that developed into adult drones today
NewDrones_healthy	bees	-		number of drone pupae that developed into healthy drones today
NewForagerSquadronsHealthy	foragerSquadrons	(N_INITIAL_BEES / SQUADRON_SIZE)		new healthy foragers at the age of first foraging

NewForagerSquadronsInfectedAsAdults	squadrons	-		number of new forager squadrons that were infected as in-hive bees
NewForagerSquadronsInfectedAsPupae	squadrons	-		number of new forager squadrons that were infected as pupae
NewIHbees	bees	-		number of worker pupae that developed into in-hive bees today
NewIHbees_healthy	bees	-		number of worker pupae that developed into healthy in-hive bees today
NewReleasedMitesToday	mites	0		number of mites released from brood cells today
NewWorkerEggs	bees	-		number of worker eggs laid today
NewWorkerLarvae	bees	-		number of worker eggs that developed into larvae today
NewWorkerPupae	bees	-		number of worker larvae that developed into pupae today
PATCHCOLOR		38		background color of the Netlogo "world"
PhoreticMites	mites	N_INITIAL_MITES_HEALTHY + N_INITIAL_MITES_INFECTED		number of all (healthy and infected) phoretic mites
PhoreticMitesHealthyRate		N_INITIAL_MITES_HEALTHY / (N_INITIAL_MITES_HEALTHY + N_INITIAL_MITES_INFECTED)		proportion of not infected mites
POLLEN_DANCE_FOLLOWERS	bees	2		number of dance followers of a successful pollen forager
POLLEN_G_kg	kg	1		amount of pollen available at "green" patch
POLLEN_R_kg	kg	1		amount of pollen available at "red" patch
POLLEN_STORE_INIT	g	100		initial pollen store
PollenFlightsToday	flights	-		sums up all pollen foraging flights during a day
PollenIdeal		FALSE		if true: pollen store is set to "IdealPollenStore_g" every day
POLLENLOAD	g	0.015	Schmickl & Crailsheim (2007): 0.015g (from Seeley 1995)	amount of pollen collected during a single, successful pollen foraging trip, equals two pollen pellets
PollenStore_g	g	POLLEN_STORE_INIT		amount of pollen stored in the colony
PollenStore_g_Yesterday	g	-		saves yesterdays' pollen store

POST_SWARMING_PERIOD	d	0		defines period after swarming until egglaying etc. normalises
PRE_SWARMING_PERIOD	d	3	Schmickl & Crailsheim (2007): 3d, Winston (1987) p. 184: "until the week before swarming"	defines period during which colony prepares for swarming
ProbLazyWinterbees		0		probability of foragers with age > ageLaziness (100d) to not take part in foraging on that day
ProbPollenCollection		0		probability of a forager to collect pollen (if active), all other (active) foragers collect nectar
PropNewToAllPhorMites		-		proportion of mites that have become phoretic on that day
PROTEIN_STORE_NURSES_d	d	7	Crailsheim (1990): 7d	if no pollen is present and the brood to nurses ratio is at its maximum, then the protein stores of the nurse bees lasts for PROTEIN_STORE_NURSES_d days; if less brood is present, then the protein stores last proportionally longer
ProteinFactorNurses		1		protein content of brood food produced by nurse bees
Pupae_W&D_KilledByVirusToday	bees	-		counts number of worker and drone pupae that were killed due to virus infection on current day
PUPATION_AGE	d	9	Winston (1987) p.50	age of worker larvae pupation
QUANTITY_G_l	l	20		amount of nectar available at "green" patch
QUANTITY_R_l	l	20		amount of nectar available at "red" patch
Queenage	d	230	queen emerged on May 15	age of the queen, only relevant if QueenAgeing = true
QueenAgeing		FALSE		if true: egg laying rate decreases with queen age (following BEEPOP) and the queen is replaced every year
random-seed		RAND_SEED		seed of the pseudo-random number generator
ReadInfile		FALSE		if true: flower patch data are read from INPUT_FILE
RecruitedFlightsToday	flights	-		sums up all flights of recruits during a day
RemainingHoney_kg	kg	5		amount of honey the beekeeper leaves in the colony at honey harvest
SaveInvadedMODroneLarvaeToPupae	ID	-		saves ID of the relevant mite organiser when drone larvae develop into pupae
SaveInvadedMOWorkerLarvaeToPupae	ID	-		saves ID of the relevant mite organiser when worker larvae develop into pupae

SaveWhoDroneLarvaeToPupae	ID	-		saves ID of the drone larvae cohort that develops into pupae
SaveWhoWorkerLarvaeToPupae	ID	-		saves ID of the worker larvae cohort that develops into pupae
SEARCH_LENGTH_M	m	17*60*FLIGHT_VELOCITY (= 6630m)	Seeley (1983): mean search trip: 17min	trip length of unsuccessful scout or recruit
SearchingFlightsToday	flights	-		sums up all flights of searching scouts during a day
SEASON_START	julian day	1	January 1	first day of foraging season
SEASON_STOP	julian day	365	December 31	last day of foraging season
SeasonalFoodFlow		TRUE		if true: seasonal pattern of food availability at flower patches, otherwise constant food flow
SHIFT_G		-40		shifts the seasonal food flow of the "green" flower patch to earlier (positive) or later (negative) in the year
SHIFT_R		30		shifts the seasonal food flow of the "red" flower patch to earlier (positive) or later (negative) in the year
ShowAllPlots		TRUE		if true: all plots are in use, otherwise: "foraging map", "foragers today [%]" and "active foragers today [%]" are switched off
SimpleDancing		FALSE		if true: two dance followers for valuable flower patches, none for non valuable
SQUADRON_SIZE	bees	100		number of foragers in the super-individuals "foragerSquadron"
STEPWIDTH		50		to scale size of coloured bars in the colony histogram (GUI, "world") **of worker brood and adult workers**
STEPWIDTHdrones		5		to scale size of coloured bars in the colony histogram (GUI, "world") of drone brood and adult drones
stopDead		TRUE		if true: run stops, if colony is dead
SumLifeSpanAdultWorkers_t	d	-		sums up the age of all adult worker died today
SummedForagerSquadronsOverTime	squadrons	-		total number of forager squadrons ever produced
Swarming		"No swarming"		Swarming options: swarming and continue of parental colony or continue with prime swarm or no swarming at all
SwarmingDate	julian day	0		julian day when swarming takes place; calculated in SwarmingProc to its acual value and reset to 0 at the end of a year

Testing		"SIMULATION - NO TEST"		allows to add special parameterisations or changes to code to test the model with the option to easily remove them or switch them on or off
TIME_NECTAR_GATHERING	s	1200	Winston (1987) p. 172: average: 30-80min; Note: modelled handling time can increase with depletion of the patch	time to fill crop with nectar if nectar quantity is not yet reduced in the flower patch
TIME_POLLEN_GATHERING	s	600	Winston (1987) p. 172: 10 min; Note: modelled handling time can increase with depletion of the patch	time to collect a pollen load if pollen quantity is not yet reduced in the flower patch
TIME_UNLOADING	s	116	Seeley (1994): 116s	time to unload nectar in the colony
TIME_UNLOADING_POLLEN	s	210	Ribbands (1953) p.131: 3.5 minutes (from Park 1922,1928b)	time to unload pollen in the colony
TodaysAllPatchesList		-		data of all flower patches for current day only
TodaysSinglePatchList		-		data of a single flower patch for current day only
TotalBeesAdded	bees	-		total number of bees ever added by the beekeeper
TotalDroneEggs	bees	-		current number of drone eggs present in the colony
TotalDroneLarvae	bees	-		current number of drone larvae present in the colony
TotalDronePupae	bees	-		current number of drone pupae present in the colony
TotalDrones	bees	-		current number of drones present in the colony
TotalEggs	bees	-		current number of worker eggs present in the colony
TotalEventsToday		-		total number of all foraging events on the current day
TotalForagers	bees	newForagerSquadronsHealthy * SQUADRON_SIZE		number of all foragers of the colony
TotalFPdetectionProb		-1		probability to find any flower patch during a single search trip
TotalHoneyFed_kg	kg	-		total amount of honey ever added by the beekeeper
TotalHoneyHarvested_kg	kg	-		total amount of honey ever harvested by the beekeeper

TotalIHbees	bees	-		current number of in-hive bees present in the colony
TotalLarvae	bees	-		current number of worker larvae present in the colony
TotalMites	mites	phoreticMites		all varroa mites present in the colony (phoretic and in brood cells)
TotalPollenAdded	kg	-		total amount of pollen ever added by the beekeeper
TotalPupae	bees	-		current number of worker pupae present in the colony
TotalWeightBees_kg	kg	-		total weight of worker and drone brood and all adults
TotalWorkerAndDroneBrood	bees	-		current number of all worker and drone eggs, larvae and pupae
VarroaTreatment		FALSE		if true: colony is treated against varroa once a year
Virus		"DWV"		choice which virus is transmitted by infected mites (DWV, APV)
VIRUS_KILLS_PUPA_PROB	1/pupal development	0.2 (DWV); 1 (APV)	Martin (2001)	probability that an infected pupa will die
VIRUS_TRANSMISSION_RATE_MITE_TO_PUPA	1/pupal development	0.89 (Virus = "DWV"); 1 (Virus = "APV")	Martin (2001)	probability that an infected mite will infect a healthy pupa
VIRUS_TRANSMISSION_RATE_PUPA_TO_MITES	1/pupal development	1 (DWV); 0 (APV)	Martin (2001)	probability that an infected pupa will infect a healthy mite
Weather		"Rothamsted (2009)"		choice between several weather scenarios and datasets
WEIGHT_WORKER_g	g	0.1	Schmickl & Crailsheim (2007): 0.1g, Martin (1998): 1kg adults = 9000 bees; Calis et al. (1999): 0.125g; higher weight => less mites!	weight of a worker bee
WriteFile		FALSE		if true: some model results are written in an output file
X_Days	d	7		defines "X" for the "run X days"-button

10. REFERENCES

Allen, M.D. (1960) The honeybee queen and her attendants. *Animal Behaviour*, **8**, 201–208.

Allen, M.D. (1963) Drone production in honey-bee colonies (*Apis mellifera* L.). *Nature*, **199**, 789-790.

Anderson, D.L. & Trueman, J.W.H. (2000) *Varroa jacobsoni* (Acari: Varroidae) is more than one species. *Experimental and Applied Acarology*, **24**, 165–189.

Amdam, G.A. & Omholt, W. (2003) The hive bee to forager transition in honeybee colonies: the double repressor hypothesis. *Journal of Theoretical Biology*, **223**, 451–464.

Becher, M.A., Scharpenberg, H. & Moritz, R.F.A. (2009) Pupal developmental temperature and behavioral specialization of honeybee workers (*Apis mellifera* L.). *Journal of Comparative Physiology A*, **195**, 673-679.

Becher, M.A., Hildenbrandt, H., Hemelrijk, C.K. & Moritz, R.F.A. (2010) Brood temperature, task division and colony survival in honeybees: A model. *Ecological Modelling*, **221**, 769-776.

Biesmeijer, J.C. & de Vries, H. (2001) Exploration and exploitation of food sources by social insect colonies: a revision of the scout-recruit concept. *Behavioral Ecology and Sociobiology*, **49**, 89-99.

Boot, W.J., Calis, J.N.M. & Beetsma, J. (1992) Differential periods of *Varroa* mite invasion into worker and drone cells of honey bees. *Experimental and Applied Acarology*, **16**, 295–301.

Boot, W.J., Schoenmaker, J., Calis, J.N.M. & Beetsma, J. (1995). Invasion of *Varroa jacobsoni* into drone brood cells of the honey bee, *Apis mellifera*. *Apidologie*, **26**, 109–118.

Bowen-Walker, P.L., Martin, S.J. & Gunn, A. (1999) The transmission of deformed wing virus between honeybees (*Apis mellifera*) by the ectoparasitic mite *Varroa jacobsoni* Oud. *Journal of Invertebrate Pathology*, **73**, 101–106.

Brodschneider, R. & Crailsheim, K. (2010) Nutrition and health in honey bees, *Apidologie*, **41**, 278–294.

Calderone, N.W. & Johnson, B.R. (2002) The within-nest behaviour of honeybee pollen foragers in colonies with a high or low need for pollen. *Animal Behavior*, **63**, 749–758.

Camazine, S. (1993) The regulation of pollen foraging by honey bees - how foragers assess the colony need for pollen. *Behavioral Ecology and Sociobiology*, **32**, 265–272.

Camazine, S. & Sneyd, J. (1991) A model of collective nectar source selection by honey bees: Self-organization through simple rules. *Journal of Theoretical Biology*, **149**, 547-571.

Carreck, N.L., Ball, B.V., Martin, S.J. (2010) Honey bee colony collapse and changes in viral prevalence associated with *Varroa destructor*. *Journal of Apicultural Research*, **49**, 93–94.

Crailsheim, K. (1990) The protein balance of the honey bee worker. *Apidologie*, **21**, 417-429.

Crailsheim, K. (1991). Interadult feeding of jelly in honeybee (*Apis mellifera* L.) colonies. *Journal of Comparative Physiology B*, **161**, 55-60.

Crailsheim, K. (1998) Trophallactic interactions in the adult honeybee (*Apis mellifera* L.), *Apidologie*, **29**, 97-112.

De Vries, H. & Biesmeijer, J.C. (2002) Self-organization in collective honeybee foraging: emergence of symmetry breaking, cross inhibition and equal harvest-rate distribution. *Behavioral Ecology and Sociobiology*, **51**, 557-569.

DeGrandi-Hoffman, G., Roth, S.A., Loper, G.L. & Erickson, E.H. Jr (1989) Beepop: a honeybee population dynamics simulation model. *Ecological Modelling*, **45**, 133–150.

Deseyn, J. & Billen, J. (2005) Age-dependent morphology and ultrastructure of the hypopharyngeal gland of *Apis mellifera* workers (Hymenoptera, Apidae), *Apidologie*, **36**, 49-57.

Donzé, G. & Guerin, P.M. (1994) Behavioral attributes and parental care of *Varroa* mites parasitizing honeybee brood. *Behavioral Ecology and Sociobiology*, **34**, 305-319.

Donzé, G., Herrmann, M., Bachofen, B. & Guerin, P.M. (1996) Effect of mating frequency and brood cell infestation rate on the reproductive success of the honeybee parasite *Varroa jacobsoni*. *Ecological Entomology*, **21**, 17–26.

Dornhaus, A., Klügl, F., Oechslein, C., Puppe, F. & Chittka, L. (2006) Benefits of recruitment in honey bees: effects of ecology and colony size in an individual-based model. *Behavioral Ecology*, **17**, 336-344.

Dreller, C., Page, R.E. Jr. & Fondrk, M.K. (1999) Regulation of pollen foraging in honeybee colonies: effects of young brood, stored pollen, and empty space. *Behavioral Ecology and Sociobiology*, **45**, 227-233.

Dreller, C. & Tarpy, D.R. (2000) Perception of the pollen need by foragers in a honeybee colony. *Animal Behaviour*, **59**, 91-96.

Dyer, F.C. (2002) The biology of the dance language. *Annual Review of Entomology*, **47**, 917-949.

Eckert, C.D., Winston, M.L. & Ydenberg, R.C. (1994) The relationship between population size, amount of brood, and individual foraging behaviour in the honey bee, *Apis mellifera* L. *Oecologia*, **97**, 248-255.

Farina, W.M. (1996) Food-exchange by foragers in the hive – a means of communication among honey bees? *Behavioral Ecology and Sociobiology*, **38** : 59–64

Farina, W.M. (2000) The interplay between dancing and trophallactic behavior in the honey bee *Apis mellifera*. *Journal of Comparative Physiology A*, **186**, 239-245.

Fefferman, N.H. & Starks, P.T. (2006) A modeling approach to swarming in honey bees (*Apis mellifera*). *Insectes sociaux*, **53**, 37–45

Fewell, J.H. & Winston, M.L. (1992) Colony state and regulation of pollen foraging in the honey bee *Apis mellifera* L. *Behavioral Ecology and Sociobiology*, **30**, 387-393.

Free, J.B. & Racey, P.A. (1968) The effect of the size of honeybee colonies on food consumption, brood rearing and the longevity of the bees during winter. *Entomologia Experimentalis et Applicata*, **11**, 241-249.

Free, J.B. & Williams, I.H. (1972) The influence of a honeybee (*Apis mellifera*) colony on egg-laying by its queen. *Entomologia Experimentalis et Applicata*, **15**, 224-228.

Fries, I., Camazine, S. & Sneyd, J. (1994) Population dynamics of *Varroa jacobsoni*: A model and a review. *Bee World*, **75**, 5–28.

Fukuda, H. & Ohtani, T. (1977) Survival and life span of drone honeybees. *Researches on Population Ecology*, **19**, 51-68.

Fukuda, H. & Sakagami, S.F., (1968) Worker brood survival in honey bees. *Researches on Population Ecology*, **10**, 31–39.

Goller, F. & Esch, H.E. (1990) Waggle Dances of Honey Bees. *Naturwissenschaften*, **77**, 594-595.

Groh, C., Tautz, J. & Rössler, W. (2004) Synaptic organization in the adult honey bee brain is influenced by brood-temperature control during pupal development. *Proceedings of the National Academy of Sciences*, **10**, 4268-4273

Grimm, V., Railsback, S.F., 2005. Individual-Based Modeling and Ecology. Princeton University Press, Princeton, NJ.

Grimm, V., Berger, U., Bastiansen, F., Eliassen, S., Ginot, V., Giske, J., Goss-Custard, J., Grand, T., Heinz, S.K., Huse, G., Huth, A., Jepsen, J.U., Jørgensen, C., Mooij, W.M., Müller, B., Pe'er, G., Piou, C., Railsback, S.F., Robbins, A.M., Robbins, M.M., Rossmanith, E., Rüger, N., Strand, E., Souissi, S., Stillman, R.A., Vabø, R., Visser, U. & DeAngelis DL (2006) A standard protocol for describing individual-based and agent-based models. *Ecological Modelling*, **198**, 115-126.

Grimm, V., Berger, U., DeAngelis, D.L., Polhill, G., Giske, J. & Railsback, S. F. 2010 The ODD protocol: a review and first update. *Ecological Modelling*, **221**, 2760-2768.

Guzmán-Novoa, E., Page, R.E. Jr., Gary, N.E. (1994) Behavioral and life-history components of division of labor in honey bees (*Apis mellifera* L.). *Behavioral Ecology and Sociobiology*, **34**, 409-417.

Harbo, J.R. (1986) Effect of population size on brood production, worker survival and honey gain in colonies of honeybees. *Journal of Apicultural Research*, **25**, 22-29.

Himmer, A. (1927) Ein Beitrag zur Kenntnis des Wärmehaushaltes im Nestbau sozialer Hautflügler. *Zeitschrift für Vergleichende Physiologie*, **5**, 375-389.

Hrassnigg, N. & Crailsheim, K. (1998) The influence of brood on the pollen consumption of worker bees (Apis mellifera L.), *Journal of Insect Physiology* **44**, 393–404.

Hrassnigg, N. & Crailsheim, K. (2005) Differences in drone and worker physiology in honeybees (Apis mellifera). *Apidologie*, **36**, 255-277.

Huang, Z.Y. & Robinson, G.E. (1992) Honeybee colony integration: Worker-worker interactions mediate hormonally regulated plasticity in division of labor. *Proceedings of the National Academy of Sciences*, **89**, 11726-11729.

Huang, Z.Y., Robinson, G.E. (1996) Regulation of honey bee division of labor by colony age demography. *Behavioral Ecology and Sociobiology*, **39**, 147–158.

Jay, S.C. (1963) The development of honeybees in their cells, *Journal of Apicultural Research*, **2**, 117–134.

Kacelnik, A., Houston, A. I. & Schmid-Hempel, P. (1986) Central-place foraging in honey bees: the effect of travel time and nectar flow on crop filling. *Behavioral Ecology and Sociobiology*, **19**, 19-24.

Kanbar, G. & Engels, W., (2003) Ultrastructure and bacterial infection of wounds in honey bee (Apis mellifera) pupae punctured by *Varroa* mites. *Parasitology Research*, **90**, 349–354.

Khoury, D.S., Myerscough, M.R. & Barron, A.B. (2011) A quantitative model of honey bee colony population dynamics. *PLoS ONE* **6**, e18491.

Khoury, D.S., Barron, A.B. & Myerscough, M.R. (2013) Modelling food and population dynamics in honey bee colonies. *PLoS ONE* **8**, e59084.

Kirchner, W.H. & Lindauer, M. (1994) The causes of the tremble dance of the honeybee, *Apis mellifera*. *Behavioral Ecology and Sociobiology*, **35**, 303-308.

Kuenen, L.P.S. & Calderone, N.W. (1997) Transfers of Varroa mites from newly emerged bees: preferences for age- and function-specific adult bees. *Journal of Insect Behavior*, **10**, 213–228.

Le Conte, Y., Mohammedi, A. & Robinson, G.E. (2001) Primer effects of a brood pheromone on honeybee behavioural development. *Proceedings of the Royal Society B*, **268**, 163-168.

Leoncini I, LeConte Y, Costagliola G, Plettner E, Toth AL, Wang M, Huang Z, Bécard J-M, Crauser D, Slessor KN, Robinson GE (2004) Regulation of behavioral maturation by a primer pheromone produced by adult worker honey bees. *Proceedings of the National Academy of Sciences*, **101**, 17559-17564.

Lindauer, M. (1947) Über die Einwirkung von Duft- und Geschmacksstoffen sowie anderer Faktoren auf die Tänze der Bienen. *Zeitschrift für Vergleichende Physiologie*, **31**, 348-412.

Lindauer, M. (1952) Ein Beitrag zur Frage der Arbeitsteilung im Bienenstaat. *Zeitschrift für Vergleichende Physiologie*, **34**, 299-345.

Martin, S. (1998) A population model for the ectoparasitic mite *Varroa jacobsoni* in honey bee (*Apis mellifera*) colonies. *Ecological Modelling*, **109**, 267–281.

Martin, S.J. (2001) The role of Varroa and viral pathogens in the collapse of honeybee colonies: a modelling approach. *Journal of Applied Ecology*, 38, 1082–1093.

Martin, S.J. (1994) Ontogenesis of the mite *Varroa jacobsoni* Oud. in worker brood of the honeybee *Apis mellifera* L. under natural conditions. *Experimental and Applied Acarology*, **18**, 87–100.

Martin, S.J. (1995) Ontogenesis of the mite *Varroa jacobsoni* Oud. in drone brood of the honeybee *Apis mellifera* L. under natural conditions. *Experimental and Applied Acarology*, **19**, 199–210.

Martin, S.J. & Kemp, D., (1997) Average number of reproductive cycles performed by *Varroa jacobsoni* in honey bee (*Apis mellifera*) colonies. *Journal of Apicultural Research*, **36**, 113–123.

Martin, S.J., Ball, B.V., Carreck, N.L. (2010) Prevalence and persistence of deformed wing virus (DWV) in untreated or acaricide-treated *Varroa destructor* infested honey bee (*Apis mellifera*) colonies. *Journal of Apicultural Research*, **49**, 72-79.

Münch, D. & Amdam, G.V. (2010) The curious case of aging plasticity in honey bees. *FEBS Letters*, **584**, 2496 –2503.

Neukirch, A. (1982) Dependence of the life span of the honeybee (*Apis mellifica*) upon flight performance and energy consumption. *Journal of Comparative Physiology*, **146**, 35-40.

Núñez, J.A. (1966) Quantitative Beziehungen zwischen den Eigenschaften von Futterquellen und dem Verhalten von Sammelbienen. *Zeitschrift für vergleichende Physiologie*, **53**, 142-164.

Núñez, J. (1970) The relationship between sugar flow and foraging and recruiting behaviour of honey bees (*Apis mellifera* L.). *Animal Behaviour*, **18**, 527-538.

Oldroyd, B.P. (1999) Coevolution while you wait: Varroa jacobsoni, a new parasite of western honeybees. *Trends in Ecology and Evolution*, **14**, 312–315.

Pankiw, T., Huang, Z.Y., Winston, M.L. & Robinson, G.E. (1998) Queen mandibular gland pheromone influences worker honey bee (*Apis mellifera* L.) foraging ontogeny and juvenile hormone titers. *Journal of Insect Physiology*, **44**, 685-692.

Pernal, S.F. & Currie, R.W. (2000) Pollen quality of fresh and 1-year-old single pollen diets for worker honey bees (*Apis mellifera* L.). *Apidologie*, **31**, 387-409.

Ribbands, C.R. (1953) The behaviour and social life of honeybees. Bee Research Association, London.

Riley, J.R., Greggers, U., Smith, A.D., Reynolds, D.R. & Menzel, R. (2005) The flight paths of honeybees recruited by the waggle dance. *Nature*, **435**, 205–207.

Rinderer, T.E. & Baxter, J.R. (1978) Effect of empty comb on hoarding behavior and honey production of the honey bee. *Journal of Economic Entomology*, **71**, 757-759.

Robinson, G.E. (1992) Regulation of division of labor in insect societies. *Annual Review of Entomology*, **37**, 637–665

Robinson, G.E. (2002) Genomics and integrative analyses of division of labor in honeybee colonies. The American Naturalist. **160**, S160–S172.

Robinson, G.E. & Huang, Z.-Y. (1998) Colony integration in honey bees: genetic, endocrine and social control of division of labor. *Apidologie*, **29**,159-170.

Rortais, A., Arnold, G., Halm, M. P., & Touffet-Briens, F. (2005). Modes of honeybees exposure to systemic insecticides: estimated amounts of contaminated pollen and nectar consumed by different categories of bees. *Apidologie,* **36**, 71-83.

Rösch, G.A. (1925) Untersuchungen über die Arbeitsteilung im Bienenstaat. *Zeitschrift für Vergleichende Physiologie*, **6**, 264-298.

Rösch, G.A. (1930) Untersuchungen über die Arbeitsteilung im Bienenstaat. *Zeitschrift für Vergleichende Physiologie*, **12**, 1-71.

Rose, K.A., Christensen, S.W. & DeAngelis, D.L. (1993) Individual-based modeling of populations with high mortality: a new method based on following a fixed number of model individuals. *Ecological modelling,* **68**, 273-292.

Rosenkranz, P., Aumeier, P. & Ziegelmann, B. (2010) Biology and control of *Varroa destructor*. *Journal of Invertebrate Pathology,* **103**, S96-S119.

Sagili, R.R. & Pankiw, T. (2007) Effects of protein-constrained brood food on honey bee (*Apis mellifera* L.) pollen foraging and colony growth. *Behavioral Ecology and Sociobiology,* **61**, 1471-1478.

Sakagami, S.F. & Fukuda, H. (1968) Life tables for worker honeybees. *Researches on Population Ecology,* **10**, 127-139.

Scheffer, M., Baveco, J.M., DeAngelis, D.L., Rose, K.A. & Van Nes, E.H. (1995) Super-individuals a simple solution for modelling large populations on an individual basis. *Ecological modelling,* **80**, 161-170.

Schmickl, T. & Crailsheim, K. (2004) Costs of environmental fluctuations and benefits of dynamic decentralized foraging decisions in honey bees. *Adaptive Behavior,* **12**, 263-277.

Schmickl, T. & Crailsheim, K. (2007) HoPoMo: A model of honeybee intracolonial population dynamics and resource management. *Ecological modelling,* **204**, 219-245.

Schmickl, T., Thenius, R. & Crailsheim, K. (2012) Swarm-intelligent foraging in honeybees: benefits and costs of task-partitioning and environmental fluctuations. *Neural Computing and Applications,* **21**, 251-268.

Schmid-Hempel, P., Kacelnik, A. & Houston, A.I. (1985) Honeybees maximize efficiency by not filling their crop. *Behavioral Ecology and Sociobiology*, **17**, 61-66.

Schulz, D.J., Huang, Z.-Y. & Robinson, G.E. (1998) Effects of colony food shortage on behavioral development in honey bees. *Behavioral Ecology and Sociobiology,* **42**, 295-303.

Seeley, T.D. (1983) Division of labor between scouts and recruits in honeybee foraging. *Behavioral Ecology and Sociobiology*, **12**, 253-259.

Seeley, T.D. (1986). Social foraging by honeybees: how colonies allocate foragers among patches of flowers. *Behavioral Ecology and Sociobiology,* **19**, 343-354.

Seeley, T.D. (1989) Social foraging in honey bees: how nectar foragers assess their colony's nutritional status. *Behavioral Ecology and Sociobiology,* **24***,* 181-199.

Seeley, T.D. (1992). The tremble dance of the honey bee: message and meanings. *Behavioral Ecology and Sociobiology*, **31**, 375-383.

Seeley, T.D. (1994) Honey bee foragers as sensory units of their colonies. *Behavioral Ecology and Sociobiology*, **34**, 51-62.

Seeley, T.D. (2009) The wisdom of the hive: the social physiology of honey bee colonies. Harvard University Press.

Seeley, T.D. & Towne, W.F. (1992). Tactics of dance choice in honey bees: do foragers compare dances? *Behavioral Ecology and Sociobiology,* **30**, 59-69.

Seeley, T.D., Reich, A.M. & Tautz, J. (2005) Does plastic comb foundation hinder waggle dance communication? *Apidologie*, **36**, 513-521.

Stabentheiner, A. (2001) Thermoregulation of dancing bees: thoracic temperature of pollen and nectar foragers in relation to profitability of foraging and colony need. *Journal of Insect Physiology*, **47**, 385-392.

Stabentheiner, A. & Hagmüller, K. (1991) Sweet food means “hot dancing” in honeybees. *Naturwissenschaften*, **78**, 471-473.

Steffan-Dewenter, I. & Kuhn, A. (2003) Honeybee foraging in differentially structured landscapes. *Proceedings of the Royal Society B*, **270**, 569-575.

Sumpter, D. & Pratt, S. (2003) A modelling framework for understanding social insect foraging. *Behavioral Ecology and Sociobiology*, **53**, 131-144.

Toth, A.L., Kantarovich, S., Meisel, A.F. & Robinson, G.E. (2005) Nutritional status influences socially regulated foraging ontogeny in honey bees. *Journal of Experimental Biology*, **208**, 4641-4649.

Vaughan, D.M. & Calderone, N.W. (2002) Assessment of pollen stores by foragers in colonies of the honey bee, *Apis mellifera* L. *Insectes sociaux*, **49**, 23-27.

Visscher, P. K., & Dukas, R. (1997). Survivorship of foraging honey bees. *Insectes sociaux*, **44**, 1-5.

Visscher, P.K. & Seeley, T.D. (1982) Foraging strategy of honeybee colonies in a temperate deciduous forest. *Ecology*, **63**, 1790-1801.

von Frisch, K. (1967) The dance language and orientation of bees. Harvard University Press, Cambridge, MA.

Weidenmüller, A., & Tautz, J. (2002). In-hive behavior of pollen foragers (*Apis mellifera*) in honey bee colonies under conditions of high and low pollen need. *Ethology*, **108**, 205-221.

Wilkinson, D. & Smith, G.C. (2002) A model of the mite parasite, *Varroa destructor*, on honeybees (*Apis mellifera*) to investigate parameters important to mite population growth. *Ecological Modelling*, **148**, 263–275.

Winston, M.L. (1987) The biology of the honey bee. Harvard University Press, Cambridge, MA.

Woyciechowski, M. & Moron, D. (2009) Life expectancy and onset of foraging in the honeybee (*Apis mellifera*). *Insectes Sociaux*, **56**:193-201.
