# Supplementary material for: BEEHAVE: a systems model of honeybee colony dynamics and foraging to explore multifactorial causes of colony failure
Source: J Appl Ecol. 2014 Mar 4;51(2):470–82. doi: 10.1111/1365-2664.12222 (PMC4283046; doi:10.1111/1365-2664.12222)
Supplement: Supplementary file 2 — Appendix S2. (JPEbecherSA2_Manual.pdf): User Manual and Guided Tour of the BEEHAVE implementation. [file JPE-51-470-s002.pdf]

Appendix S2

The BEEHAVE user manual

Contents

1 BEEHAVE-COLONY: An interface overview……………………………….. 2

1.1 Setup & Running options………………………………………………….. 3

1.2 Input options………………………………………………………………. 8

1.2.1 ‘Input field’……………………………………………………….. 8

1.2.2 ‘Switch’……………………………………………………………. 11

1.2.3 ‘Chooser’………………………………………………………….. 13

1.3 Output options……………………………………………………………... 16

1.3.1 ‘Output plot’………………………………………………………. 16

1.3.2 ‘Output monitors’…………………………………………………. 23

1.4 World of BEEHAVE……………………………………………………… 25

2 Guided Tour……………………………………………………………………… 28

2.1 The Default scenario………………………………………………………. 28

2.2 The *Varroa* Scenario………………………………………………………. 48

3 BEEHAVE version control and documentation of simulation experiment…… 66

3.1 BEEHAVE version control………………………………………………... 66

3.2 Documentation of BEEHAVE simulation experiments…………………… 67

References……………………………………………………………………….... 68

List of Tables…………………………………………………………………….. 69

1 BEEHAVE_COLONY: An Interface Overview

After you have started Beehave2013.nlogo, you will discover an interface tab containing an enormous number of diverse Netlogo elements. In the face of this, here we get started clarifying the basic interface elements of BEEHAVE and offering a guided tour in order to make sure that you understand how BEEHAVE works and how you can use BEEHAVE to study the effects of various stressors on the performance of managed honeybee colonies.

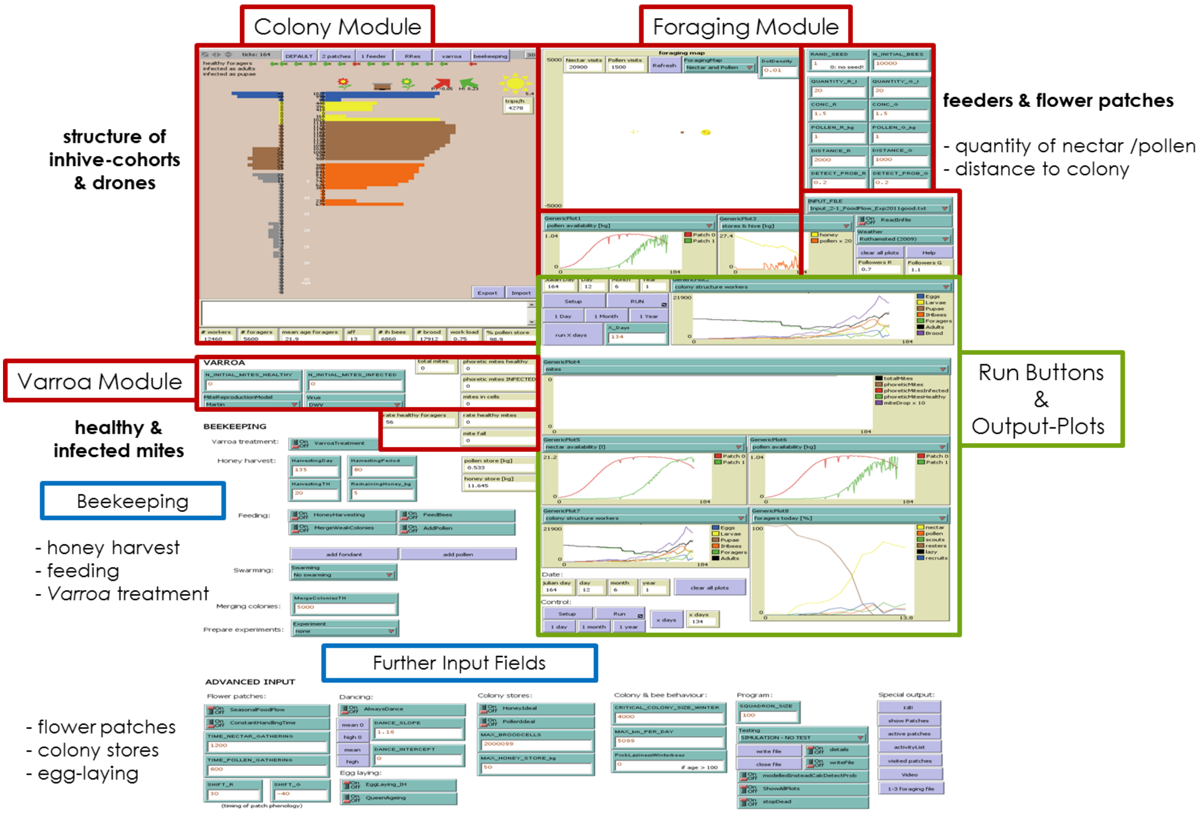

Figure 1.1: Overview of the BEEHAVE interface tab.

When started, BEEHAVE opens an interface tab that comprises several initialization and output options for the *Colony*, *Foraging* and *Varroa* modules which are described and explained in the following sections of this user manual:

◊ Setup & Running options

◊ Input options

◊ Output options

◊ World of BEEHAVE

1.1 Setup & Running options

The interface tab of BEEHAVE provides several setup and running buttons that initiate and start the simulation run as well as offering the option to choose pre-defined scenarios.

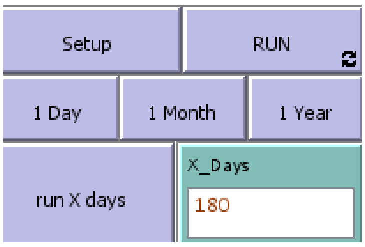

The **Setup** button initiates the model settings called by the parameterization procedure whereas the **Run** button starts the model run over an indefinite period (or until the death of the honeybee colony). Alternatively you can run the model for defined time intervals from one day to many years by using the **1 Day**, **1 Month**, **1 Year** and **run X days** buttons.

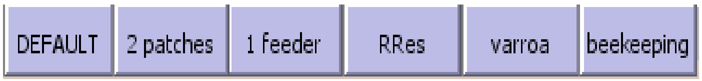

Furthermore, you can start pre-defined scenarios by the setup options of the BEEHAVE interface shown above. You may choose between the **Default**, **2 patches**, **1 feeder**, **RRes**, **varroa** and the **beekeeping** scenario.

When the **Default** scenario is initialized, all input variables will be reset to their default values, except for RAND_SEED, DotDensity, and INPUT_FILE. These default settings include 2 food patches located at distances of 1500 and 500 m from the hive, seasonal food flow as well as local weather data of Rothamsted from 2009, but do not include any beekeeping options or infestation by DWV-transmitting (deformed wing virus) *Varroa* mites.

The other pre-defined scenarios change *some* input variables. Thus, the **beekeeping** scenario implements a virtual beekeeper that feeds bees, harvests honey, merges weak colonies and performs *Varroa* treatments.

In addition, **1 feeder** offers the opportunity to implement only one active feeder, constant food flow and a short, constant handling time for nectar and pollen collection.

The **varroa** scenario adds 20 *Varroa* mites, 50 percent of which are infected with the deformed wing virus (DWV), to the default settings.

The **RRes** scenario integrates the locations and sizes of real patches of forage into the model run from a landscape map of the area around Rothamsted, and includes seasonal food flow and varying flower handling times for nectar and pollen foraging. This gives a more realistic foraging landscape for the bees. These data are read in from an input file.

Additionally, the pre-defined scenario **2 patches** is similar to the default settings, but if you change some input variables during succeeding simulation runs not patch related variables as *Weather* or *HoneyHarvesting* are not reset at restart of this pre-defined scenario.

You can choose several advanced model initializations and outputs by clicking defined buttons (Table 1.1) during the ongoing model run.

Table 1.1: Functions of advanced initialization and output buttons

**Button**	**Function**
Export Import	export and import the BEEHAVE world data as a csv-file that contains the actual values of all agent variables (global, turtle, patch and link variables) as coordinates, identity number, size, label, pen-mode, and specific defined features (e.g. ploidy, number_healthy, activity, age, EEF) of in-hive cohorts, forager squadrons, signs, outputs and links (thumbshot of BEEHAVE data)
Kill!	kills all in-hive bees. Right-click the button and add/remove semicolon to allow other modifications
show Patches	print information about the existing forage patches to the 'Command Center': patch ID, patch type, distance to colony, x, y, patch size (size_sqm), current amount of nectar [l] and pollen [kg], nectar concentration, current energetic efficiency (EEF), number of potential recruits (followers), detection probability, handling time of nectar and pollen, total visitors
active patches	prints information about active patches on that day to the 'Command Center': patch ID, distance to colony, patch size (size_sqm), amount of nectar [l] and pollen [kg], nectar concentration, detection probability, handling time of nectar and pollen, total visitors
activityList	records all activities of a forager squadron during the day and writes the activity list to the Command Center (for definitions of activities - see Table 1.2)
visited patches	prints information about the visited patches on that day to the 'Command Center': patch ID, distance to colony, patch size (size_sqm), amount of nectar [l] and pollen [kg], nectar concentration, detection probability, handling time of nectar and pollen, number of nectar and pollen visits on that day, total visitors
1-3 foraging file	creates a foraging file containing the number of visits per flower patch and day, which can be used as input file for the auxiliary "BEEHAVE_Landscape" tool.
write file<br>close file	writes and closes a file for simulation testing during a simulation run

mean 0<br>high 0	The dance slope of a dancing bee can be set to the mean (0.51) or maximum value (1.16) in order to calculate the number of circuits a bee dances for a patch depending on the patch quality (energetic efficiency) Default: 1.16
mean<br>high	The dance slope and intercept of a dancing bee can be set to the mean (0.51 and -11.1) or maximum value (1.16 and -17.7) in order to calculate the number of circuits a bee dances for a patch depending on its energetic efficiency: e.g. distance to the hive Default: dance slope (1.16) and intercept (0)
add pollen<br>add fondant	add 1 kg pollen or 1 kg fondant to the colony's pollen store at any time during the model run
Version Test	runs the model for 1825 days to detect, if the official BEEHAVE code was changed

The advanced button **activityList** offers the option to write all foraging activities of all active forager squadrons on that day to the 'Command Center'. So, the ID of the respective forager squadron, its flight distance travelled as well as its foraging activities per foraging round on that day are shown. Table 1.2 gives an overview of all possible foraging activities.

Table 1.2: Foraging activities of forager squadrons

**Activities**	**Definition**
NF	potential nectar forager, who would search or collect nectar, if it would not be resting
PF	potential pollen forager, who would search or collect pollen, if it would not be resting
R	resting bee that can be recruited to an advertised flower patch
L	lazy bee that cannot be recruited to an advertised flower patch won`t start foraging spontaneously
Xp/ Xn	experienced pollen / nectar forager
Sp/ Sn	scout searching a new pollen / nectar patch
fN/ fP	a scout finds a nectar and pollen patch and becomes a successful nectar / pollen forager
feN/ feP	find an empty nectar / pollen patch
bN/ bP	bringing nectar / pollen to the colony store, become experienced forager
E	unsuccessful scout returning empty
frN/ frP	a recruit finds a nectar / pollen patch and becomes a successful nectar / pollen forager
eSn/ eSp	a recruit found a patch that provides no nectar / pollen, and becomes a scout
mSn/ mSp	a recruit didn't find the advertised nectar/ pollen patch and searches a new patch
Dn/ Dp	a successful nectar / pollen forager dances and communicates EEF and ID of the patch
rFnNF/ rFpPF	a resting and unexperienced forager is recruited to an advertised flower patch by a dancing forager and becomes a nectar / pollen forager
rFnxNF/ rFpxPF	if the advertised nectar patch has higher EFF or the advertised pollen patch has a shorter trip duration than the known patch, the dance follower becomes a nectar / pollen forager and searches for the advertised patch
Xnr/ Xpr	an experienced forager recruited by following a dance, but start foraging at a known nectar / pollen patch with the same or higher EEF / trip duration than the advertised patch
An/ Ap	resting foragers that abandoned their nectar / pollen patch still rest
AnSn/ ApSp	an active forager that abandoned its nectar / pollen patch searches for a new one
AfR	an active forager that was not recruited in the foraging round before, abandons foraging and becomes a resting bee
Rx	a pollen or nectar forager that switches to resting
N/ P	collect nectar / pollen at a flower patch
uS	an unsuccessful searching bee finds no flower patch
End	end of the today’s foraging period of the respective forager squadron

Keep in mind that the Netlogo toolbar allows you to control the speed of simulation runs and the viewing of updates. Use the speed slider to control the speed of the model run, and use the chooser to adjust the frequency of viewing updates (from continuous to tick-based). Thus, continuous updates means that the Netlogo view will be updated many times a second, whereas the tick-based updates results in a Netlogo view only updated when the tick counter proceeds.

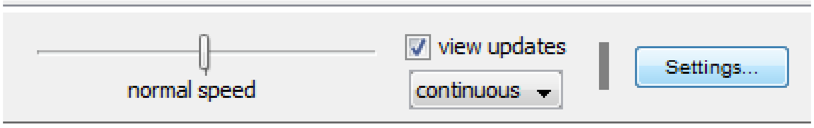

The ticks count daily as the model run proceeds. If you click the **Settings** button on the toolbar, then you will see the 'Model Settings' window (below) with a box to choose whether to 'Show tick counter' or not.

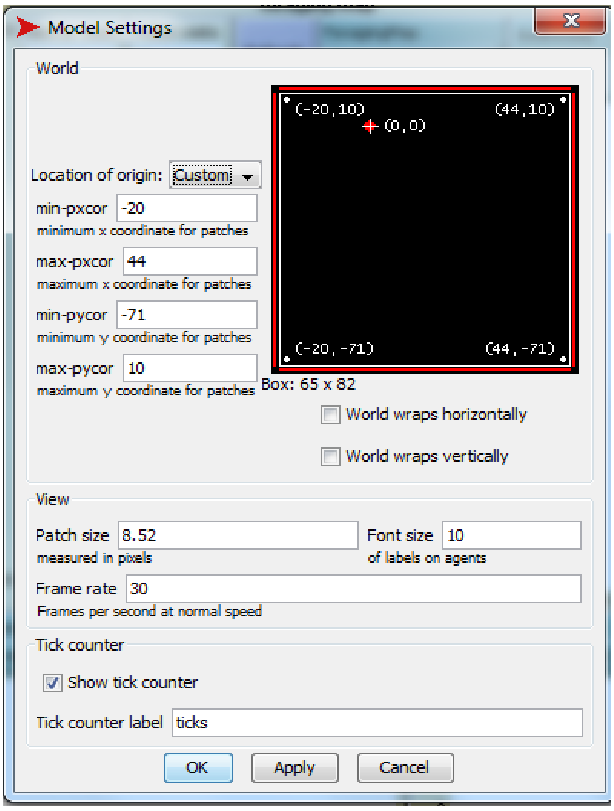

1.2 Input options

The BEEHAVE interface tab offers a large number of input options using ‘Input fields’, ‘Switch’ and ‘Chooser’ buttons (blue). These define the parameter settings, input data and a variety of adjustment options of colony and foraging processes, *Varroa* infestation and beekeeping practices for simulation experiments in order to investigate the effects of combined stressors on the performance of honey bee colonies. Thus, the following tables in this section give a review of the various input options in order to clarify these complex model initialization settings. Moreover, initial settings (default values) of all input variables are demonstrated.

1.2.1 ‘Input field’

‘Input fields’ allow you to initialize and vary the most important parameter settings of colony size and colony stores variables (CRITICAL_COLONY_SIZE_WINTER, MAX_BROODCELLS, MAX_HONEY_STORE_kg), quantities and qualities of flower patches crucial for foraging (QUANTITY_G_l, CONC_G, DISTANCE_G, DETECT_PROB_G), infestation by deformed wing virus transmitting *Varroa* mites (N_INITIAL_MITES_HEALTHY, N_INITIAL_MITES_INFECTED) and last but not least to implement beekeeping treatments (HarvestingDay HarvestingPeriod) as presented in table 1.3.

Table 1.3: Definition and initial settings (default value) of ‘Input field’ variables belonging to the following module *Colony*, *Foraging - Flower patch*, *Varroa* and optional *Colony - Beekeeping* are given. Optional initial settings are put in square brackets.

**Variable**	**Definition**	**Initial setting**	**Module**
RAND_SEED	initial value of the pseudo-random number generator; undefined if set to 0	8	-
N_INITIAL_BEES	initial colony size	10,000	Colony
QUANTITY_R_l	amount of nectar available at “red” patch [l]	20	Foraging – Flower patch
QUANTITY_G_l	amount of nectar available at “green” patch [l]	20	Foraging – Flower patch
CONC_R	sucrose concentration in nectar of “red patch” [mol/l]	1.5	Foraging – Flower patch
CONC_G	sucrose concentration in nectar of “green” patch [mol/l]	1.5	Foraging – Flower patch
POLLEN_R_kg	amount of pollen available at “red” patch [kg]	1	Foraging – Flower patch
POLLEN_G_kg	amount of pollen available at “green” patch [kg]	1	Foraging – Flower patch
DISTANCE_R	distance of the “red” patch to the colony [m]	1500	Foraging – Flower patch
DISTANCE_G	distance of the “green” patch to the colony [m]	500	Foraging – Flower patch
DETECT_PROB_R	probability that a searching forager squadron finds the “red” patch	0.2	Foraging – Flower patch
DETECT_PROB_G	probability that a searching forager squadron finds the “green” patch	0.2	Foraging – Flower patch
X_Days	defines the number of days simulated by the "run X days" button	-	-
N_INITIAL_MITES_HEALTHY	initial number of healthy mites	0	Varroa
N_INITIAL_MITES_INFECTED	initial number of infected mites	0	Varroa
HarvestingDay	first day of the year on which harvesting honey is possible	- [on day 135]	Colony – Beekeeping
HarvestingPeriod	period of time during which harvest of honey is possible, starting on harvesting day	- [80d]	Colony – Beekeeping
HarvestingTH	minimum honey store of the colony that allows to harvest honey	- [30kg]	Colony – Beekeeping
RemainingHoney_kg	amount of honey that the beekeeper	-	Colony –

	leaves in the colony when harvesting	[5kg]	Beekeeping
MergeColoniesTH	beekeeper adds foragers to colonies with a colony size below the threshold value of 5000 bees in autumn	- [5000]	Colony – Beekeeping
TIME_NECTAR_GATHERING	time to fill crop with nectar if nectar quantity is not yet reduced in the flower patch [s]	1200	Foraging
TIME_POLLEN_GATHERING	time to collect a pollen load if pollen quantity is not yet reduced in the flower patch [s]	600	Foraging
SHIFT_R	shifts the seasonal food flow of the “red” flower patch to earlier (positive) or later (negative) in the year	30	Foraging – Flower patch
SHIFT_G	shifts the seasonal food flow of the “green” flower patch to earlier (positive) or later (negative) in the year	-40	Foraging – Flower patch
DANCE_SLOPE	to calculate the number of circuits that a bee dances for a patch depending on the energetic efficiency of the flower patch	1.16	Foraging
DANCE_INTERCEPT	to calculate the number of circuits that a bee dances for a patch depending on the energetic efficiency of the flower patch	0	Foraging
MAX_BROODCELLS	maximum available brood space of the colony	2000099	Colony
MAX_HONEY_STORE_kg	maximum amount of honey that can be stored [kg] in the hive	50	Colony
CRITICAL_COLONY_ SIZE_WINTER	threshold of colony size for winter survival on Julian day 365, below this critical threshold the colony dies	4000	Colony
MAX_km_PER_DAY	maximum total distance a forager can fly during one day	5099	Foraging
ProbLazinessWinterbees	probability of foragers with an age of > 100 days to not take part in foraging at all on that day	0	Foraging
SQUADRON_SIZE	number of foragers in the super-individuals “foragerSquadron”	100	Foraging

1.2.2 'Switch'

The BEEHAVE interface tab includes a large number of 'Switches' for boolean variables. Here, important input data and settings are specified. You can define specific feedback loops in dependence of actual colony composition and stores, food flow conditions and foraging properties for simulation experiments using "on" and "off" switches (table 1.4).

The **Default** scenario sets the constant handling time for nectar and pollen collection (*ConstantHandlingTime*) "off" and sets a season-dependent pattern in food availability (*SeasonalFoodFlow*) "on". Hence, the maximum amount of either nectar or pollen available at the "red" and "green" patches depends on the season (if *ReadInFile* = false). If *ConstantHandlingTime* is off, then higher depletion of pollen and nectar at the flower patch during the day results in longer handling times to collect nectar or pollen loads.

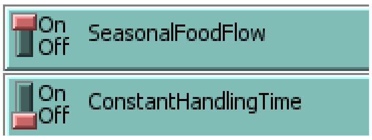

Moreover, the effect of queen's age on queen's egg-laying rate (*QueenAgeing*) is turned off. This implies that the seasonal egg-laying is not reduced with increasing age of the queen. But the egg-laying rate of the queen is affected by the number of available in-hive bees that feed the queen as well as prepare and nurse the brood (*EggLaying_IH*).

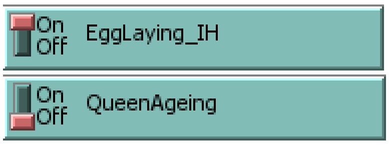

All beekeeping options such as feeding bees (*FeedBees*) and harvesting honey (*HoneyHarvesting*) are switched off.

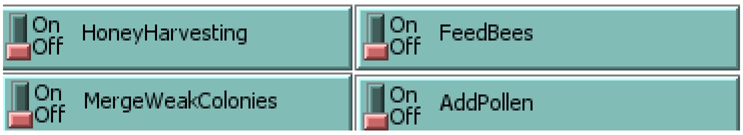

Table 1.4: Definition and initial setting (default value) of 'Switch' state variables belonging to the following modules*: Colony, Foraging, Varroa,* optional *Colony – Beekeeping* as well as involved producers *DoPlotsProc*, *Start_IBM_ForagingProc* (Foraging *) and *WriteToFileProc*

"Switch" - variable	Definition	Default	Module
ReadInfile	read in parameters of flower patches with their daily food availability during season from input files	off	Foraging
VarroaTreatment	beekeeping option to expose all phoretic mites to an additional daily mortality during a defined period	off	Colony - Beekeeping
HoneyHarvesting	beekeeping option to potentially harvest honey starting on day 135 over a period of 80 days	off	Colony - Beekeeping
FeedBees	beekeeping option to stock up the honey stores of the colony if these are below a certain threshold	off	Colony - Beekeeping
MergeWeakColonies	beekeeping option to add bees to a weak colony (colony size < 5000) during autumn	off	Colony - Beekeeping
AddPollen	beekeeping option to add additional pollen to the colony stores in spring in order to increase the colony growth	off	Colony - Beekeeping
SeasonalFoodFlow	choose a bell shaped seasonal flow curve of maximum food availability (on) or a constant food flow during season (off)	on	Foraging
ConstantHandlingTime	adjust the handling time of nectar or pollen collection by a honeybee to the depletion of nectar or pollen at a patch (off) or to a constant handling time during a day (on)	off	Foraging
AlwaysDance	dance circuits of foragers are dependent (off) or independent (on) of the energetic efficiency of a patch	off	Foraging
EggLaying_IH	integrate the feedback loop between the number of available nurse bees and the egg-laying rate of the queen	on	Colony
HoneyIdeal	set the honey store to the maximum every day	off	Colony
PollenIdeal	set the pollen store to the IdealPollenStore_g every day, no pollen foraging takes place	off	Colony
details	record the results in an output file after each foraging round	on	Foraging *
writeFile	generate an output file	off	WriteToFile Proc
modelledInstead CalcDetectProb	option to choose between modeled and calculated detection probabilities of flower patches	off	Foraging
ShowAllPlots	switch off the 'foraging map', 'foragers today [%]' and 'active foragers today [%]' (off); (on) all plots are in use	on	DoPlotsProc
StopDead	stop the simulation run if colony is dead	on	Colony
QueenAgeing	adjust the queen's daily egg-laying rate to queen's age; egg-laying rate is reduced with increasing queen's age (on)	off	Colony

1.2.3 ‘Chooser’

Additionally, the BEEHAVE interface tab offers several ‘Chooser’ input options (table 1.5). For example you can choose a model for mite reproduction (*MiteReproductionModel*) which determines how many mites can enter a brood cell as well as their reproduction rates. The mite reproduction rate is reduced if more than one *Varroa* mite invades a brood cell. However, the mite reproduction model according to ‘Martin’ (2001) allows a maximum number of four *Varroa* mites entering one brood cell whereas referring to ‘Fuchs & Langenbach’ (1989) up to 16 mites can invade one brood cell. Thus, different mite reproduction models may discriminatively affect honeybee colony dynamics.

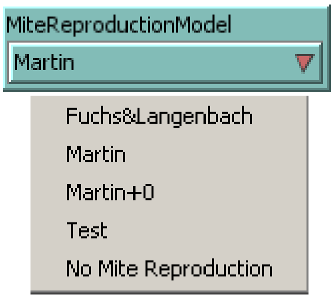

Moreover, specific weather scenarios can be chosen on the interface tab by using the chooser *Weather*. These scenarios are based either on real local weather data that define the daily available foraging period for each day of one year e.g. ‘Rothamsted (2009)’ or several years e.g. ‘Berlin (2000-2006)’ or on theoretical data e.g. ‘HoPoMo_Season’ (bell shapes curve of seasonal foraging period) or depict a constant foraging period of eight hours per day ‘Constant’.

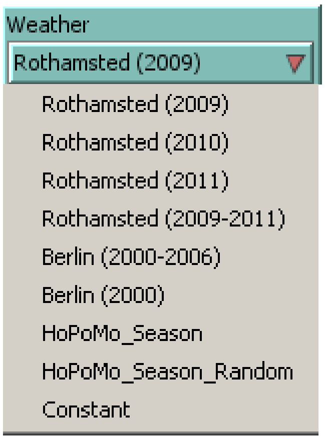

Finally specific seasonal food flow data of flower patches in different landscapes, affecting foraging activity in different ways can be selected (*INPUT_FILE*).

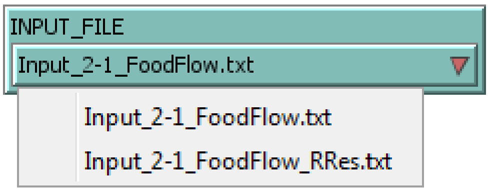

Table 1.5: Definition of integrated ‘Chooser’ variables and their several options important for the following BEEHAVE modules *Colony*, *Foraging* and *Varroa* are listed. The procedures *GenericPlottingProc* and *GenericPlotClearProc* (GenericPlot) display the variables which can be selected from the specific chooser ‘Generic Plots’.

**‘Chooser’ variable**	**Definition**	**Options**	**Module**
ForagingMap	simple representation of available patches offering nectar and pollen in the landscape and current foraging activities of the forager squadrons (see section 1.3.1)	show nothing<br>Nectar foraging<br>Pollen foraging<br>Nectar and pollen<br>All visits<br>All patches<br>Available patches	Foraging
INPUT_FILE	read in food flow data of available flower patches for 365 days	different food flow txt-files	Foraging
Weather	choose a specific weather scenario based on real local weather data or theoretical data that define the daily foraging period of each day of one or several years; hours of sunshine on days with a temperature above 15° C serve as an approximation of the daily foraging period; "Constant" allows eight hours of foraging every day.	Rothamsted (2009)<br>Rothamsted (2010)<br>Rothamsted (2011)<br>Rothamsted (2009-2011)<br>Berlin (2000-2006)<br>Berlin (2000)<br>HoPoMo_Season<br>HoPoMo_Season_Random<br>Constant	Foraging
MiteReproductionModel	choose a model for *Varroa* mite reproduction; models differ in their maximum number of infested mites per brood cell and degree of decrease in mite reproduction rate	Fuchs & Langenbach<br>Martin<br>Martin + 0<br>Test<br>No Mite Reproduction	Varroa
Virus	select one virus type transmitted by *Varroa* mites	DWV<br>APV<br>benignDWV<br>modifiedAPV	Varroa

		TestVirus	
Swarming	options to allow swarming of the honeybee colony<br>Swarm control:<br>The colony does not swarm but the day of the prevented swarming is recorded.<br>Swarming (parental colony):<br>A swarm is released, simulation continues with the colony left in the hive and a new queen.<br>Swarming (prime swarm):<br>A swarm is released, simulation continues with the swarm and the old queen.	No swarming<br>Swarm control<br>Swarming (parental colony)<br>Swarming (prime swarm)	Colony-beekeeping
Experiment	to create specific colony conditions, defined in GoTreatmentProc	none<br>Experiment A<br>Experiment B	Colony
Testing	add special parameterizations or parameter changes to the code in order to test the model including the option to easily remove them	SIMULATION – NO TEST	Currently not in use
Generic Plots	create a variety of output plots	see below (section 1.3.1)	GenericPlot

1.3 Output options

The BEEHAVE interface tab also offers a variety of output plots and these can be monitored in order to inspect what is going on inside the model run.

Note: The range of output monitors and plots that display important variables of colony status as well as foraging and *Varroa* dynamics can be extended as you like, by programming just a few additional code lines and adding advanced Netlogo output plots and monitors to the interface tab.

1.3.1 'Output plot'

The output plots show either histograms or line charts of the colony, foraging and mite infestation dynamics that the model is generating. For instance, the development of all age-based cohorts (*colony structure workers* & *drones*), *brood care [%]*, *consumption [g/day]*, mite infestation (*mites*), proportion of active foragers (*active foragers [%]*), number of foraging trips per hour sunshine (*trips per hour sunshine (E-3)* as well as *nectar availability [l]* over the whole time of model run are plotted. However, plots that show detailed foraging activities during a foraging round on the day e.g. *foragers today [%]* and *active foragers today [%]* are newly generated and cleared once a day. Moreover, patches that offer pollen and nectar (*Available patches*) as well as foraging activities of honeybee forager squadrons at the advertised patches (*Nectar foraging*, *Pollen foraging*, *All visits*) on that day can be illustrated on a two dimensional landscape representation ('foraging map'). All these output variables can be selected by the chooser 'Generic Plots' (see above). The following table 1.6 and plots give a review of all actually integrated output variables.

Table 1.6: Description of important plotted output variables

Variable	Description	Module
colony structure workers	number of worker eggs, larvae, pupae, in-hive bees and foragers as well as the total number of adults (in-hive worker bees + foragers) and the total amount of brood (worker eggs + worker larvae + worker pupae) over time	Colony
drones	number of drone eggs, larvae, pupae and adults over time	Colony
egg laying	number of newly laid eggs by the honeybee queen over time	Colony
broodcare [%]	relative amount of brood to the maximum nursing force (Workload), the relative pollen stores compared to ideal pollen stores (Pollen) and the protein content of the "jelly" produced by nurse bees (Protein) over time	Colony
age forager squadrons	histogram of forager squadrons: number of healthy (black), diseased (red: infected as pupae) and virus-carrying (blue: infected as adults) forager squadrons by age	Colony
aff & lifespan	age of first foraging and the lifespan of workers over time	Colony
mileometer	histogram of total flight distances of forager squadrons (> 800 km per squadron? = death)	Foraging
stores & hive [kg]	weight of honey and pollen stores of the colony and weight of the hive over time	Colony
colony weight [kg]	weight of all bee cohorts without the weight of the hive and stores over time	Colony
consumption [g/day]	daily consumption of honey and pollen [g/day] by the colony over time	Colony
honey gain [kg]	daily change in honey stores [kg] over time	Colony
mites	number of phoretic mites, phoretic mites infected, phoretic mites healthy as well as the total number of mites and the number of mites dropped from the comb to colony floor (increased by factor 10) over time	Varroa
proportion infected mites	proportion of virus infected phoretic mites	Varroa
active foragers [%]	percentage of foragers that have been active during the day over time	Foraging
active foragers today [%]	percentage of active and dead foragers of all foragers over all foraging rounds on that day	Foraging
Foraging Period	daily amount of time available for foraging trips [h] over time defined by the weather (hours of sunshine on days with a maximum temperature > 15 ° C)	Foraging
foraging probability	probability that resting bee will start foraging spontaneously depending on the demand for pollen and nectar over time	Foraging
foragers today [%]	percentage of nectar and pollen foragers, scouting, resting, lazy and recruited bees of all foragers over all foraging rounds on that day	Foraging
loads returning foragers [%]	percentage of returning foragers loaded with pollen and nectar as well as foragers returning empty over time	Foraging

mean trip duration	mean duration [min] of a foraging trip on that day over time	Foraging
mean total km per day	mean total flight distances [km] of foragers on that day over time	Foraging
# completed foraging trips (E-3)	number of completed foraging trips over time, divided by 10^3	Foraging
trips per hour sunshine (E-3)	number of foraging trips of foragers per hour sunshine, divided by 10^3	Foraging
nectar availability [l]	summed nectar availability at all patches over time	Foraging
pollen availability [kg]	summed pollen availability at all patches over time	Foraging
ForagingMap	representation of all, or only currently available patches, offering nectar and pollen in the landscape or of today's foraging: nectar and pollen foraging, or total visits to patches;<br>yellow dots refer to nectar, orange dots to pollen	Foraging

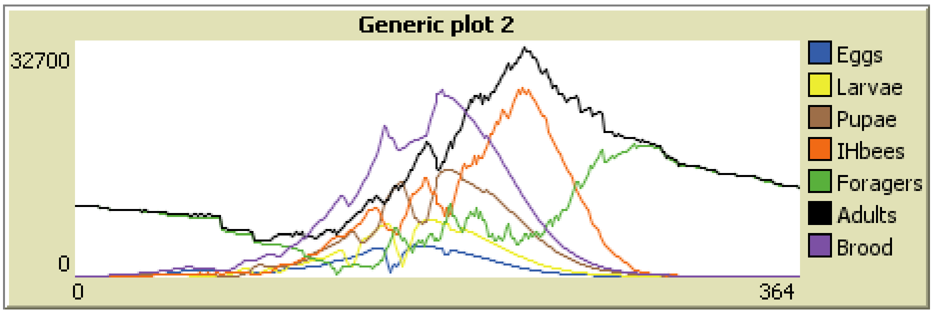

Figure 1.2: *Colony structure workers*. The total number of eggs, larvae, pupae, adult in-hive bees (IHbees) and foragers as well as the total number of worker brood cells (Brood) and adult working bees (Adults) over time

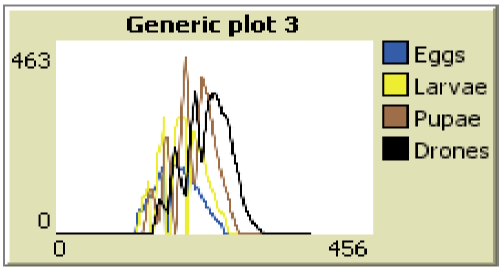

Figure 1.3: *Drones*. Total number eggs, larvae, pupae and adult drones over time

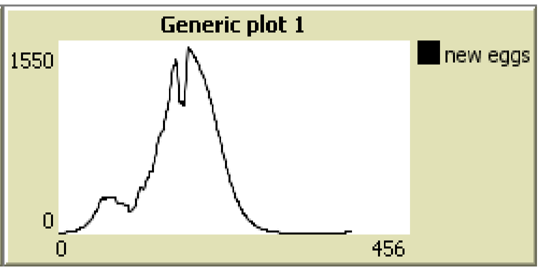

Figure 1.4: *Egg-laying*. The season-dependent pattern in the number of newly laid eggs by the honeybee queen

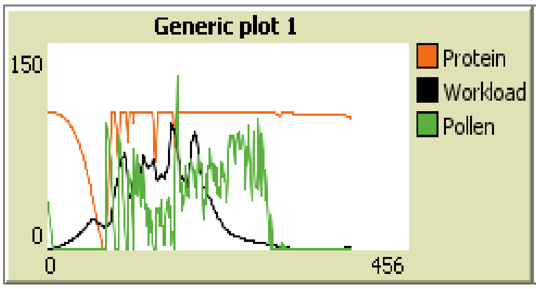

Figure 1.5: *Brood care [%]*. The relative amount of brood to the maximum nursing force (Workload), the relative pollen stores compared to ideal pollen stores (Pollen) and the protein content of "jelly" produced by nurse bees (Protein) over time

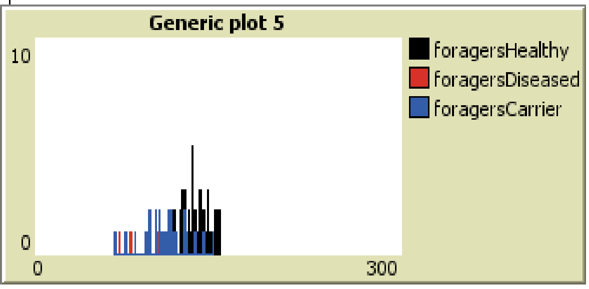

Figure 1.6: *Age forager squadrons*. Histogram of forager squadrons of healthy (black), diseased (red: infected as pupae) and virus-carrying (blue: infected as adults) foragers by age

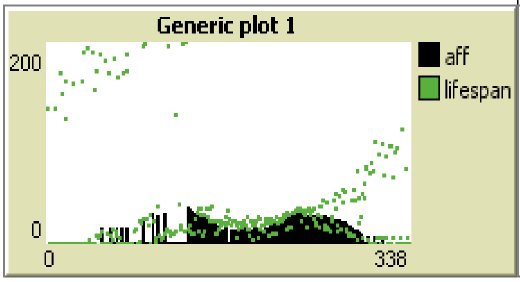

Figure 1.7: *Aff & lifespan*. Age of first foraging and the lifespan of workers over time.

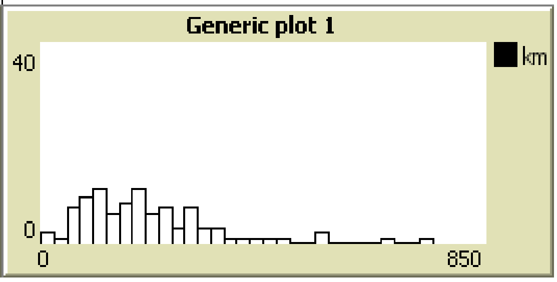

Figure 1.8: *Mileometer***.** Histogram of total flight distances [km] of forager squadrons.

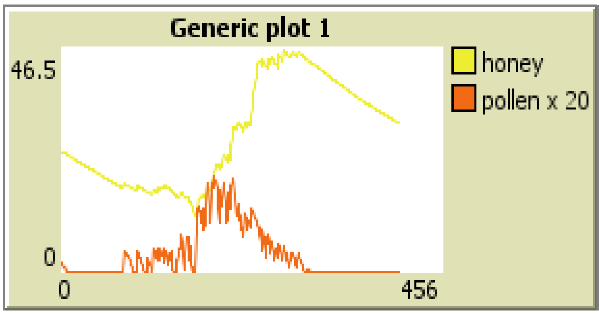

Figure 1.9: *Stores & hive [kg]*. The seasonal change of weight [kg] of stored honey and pollen (pollen x 20) as well as the complete hive.

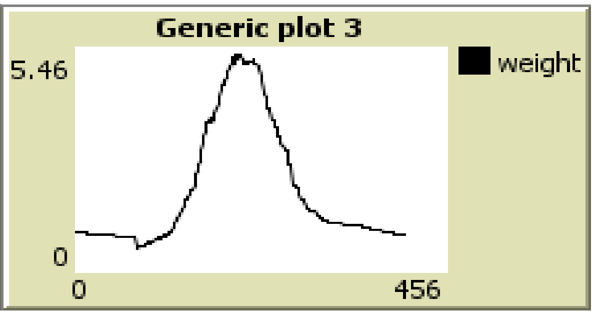

Figure 1.10: *Colony weight [kg]*. The seasonal change of weight [kg] of all bee cohorts.

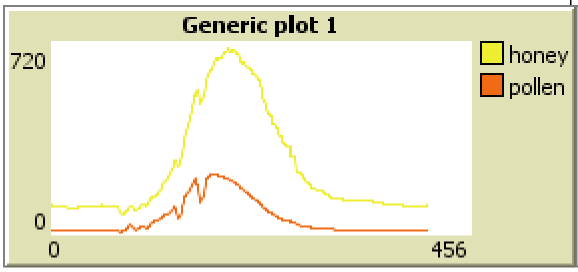

Figure 1.11: *Consumption [g/day*]. The daily consumption of honey and pollen [g/day] by the colony over time.

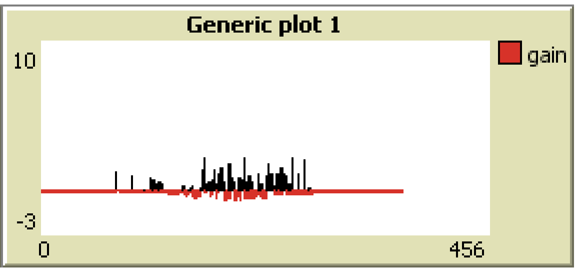

Figure 1.12: *Honey gain [kg]*. The daily change of honey stores [kg] over time.

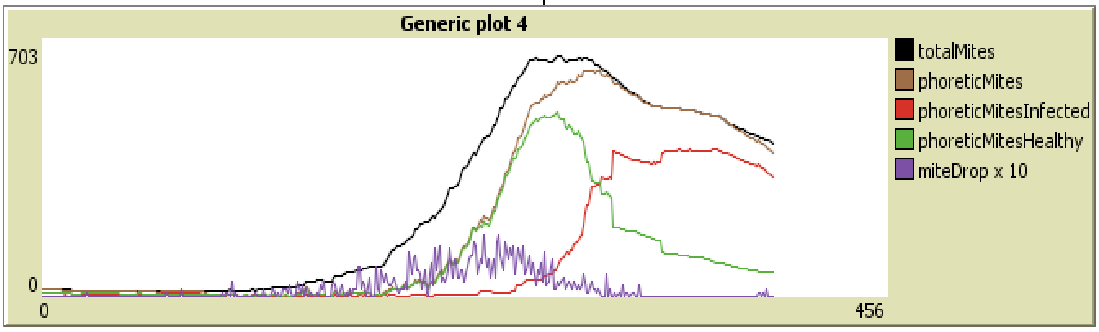

Figure 1.13: *Mites***.** The seasonal change of the number of phoretic mites, phoretic mites infected, phoretic mites healthy as well as the total number of mites and the number of mites dropped (miteDrop x 10).

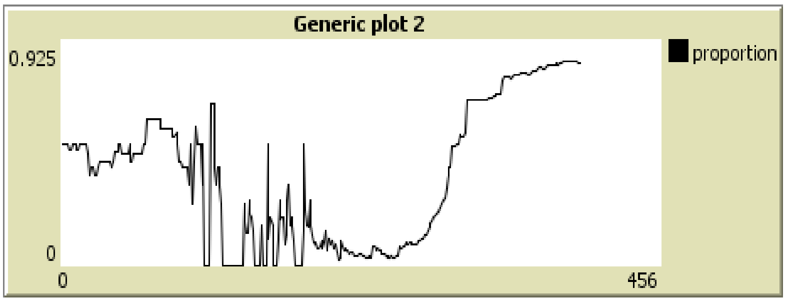

Figure 1.14: *Proportion infected mites*. The seasonal change in the proportion of phoretic mites infected by DWV (deformed wing virus) or APV (acute paralysis virus).

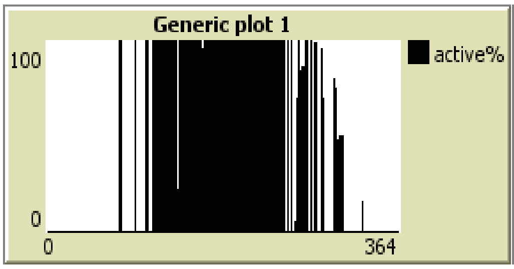

Figure 1.15: *Active foragers [%]*. The percentage of foragers active during a day over time.

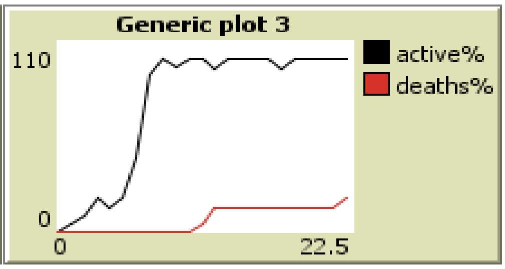

Figure 1.16: *Active foragers today [%]*. The percentage of active and dead foragers of all foragers over all foraging rounds on that day.

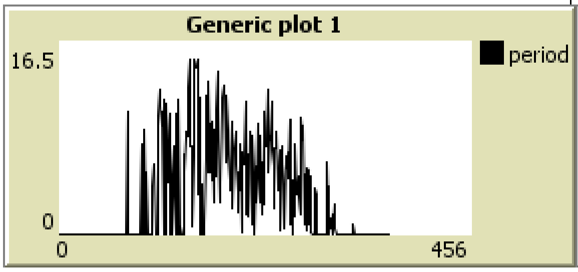

Figure 1.17: *Foraging period*. The season-dependent pattern in the daily amount of time available for foraging trips [h]. The daily available time for foraging trips is defined by the hours of sunshine on days with Tmax > 15 °C.

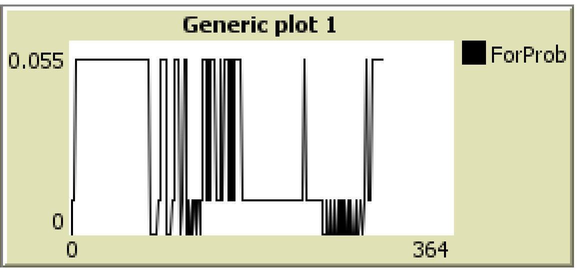

Figure 1.18: *Foraging probability*. The probability that a resting forager will start foraging spontaneously over time.

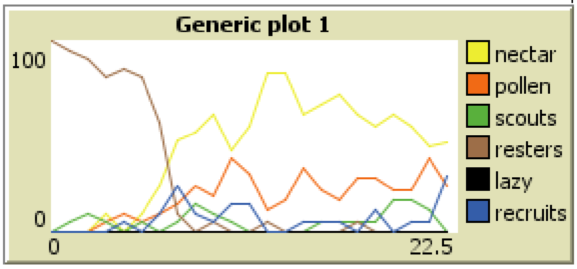

Figure 1.19: *Foragers today [%]***.** The percentage of nectar and pollen foragers, scouting, resting, lazy and recruited bees of all forager over all foraging rounds on that day.

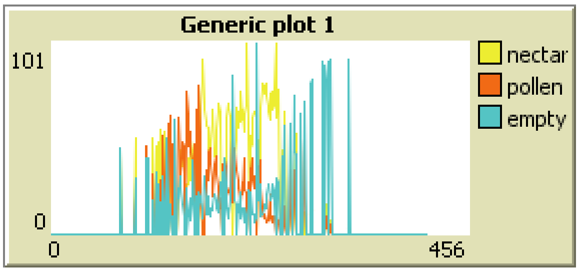

Figure 1.20: *Loads returning foragers [%]*. The percentage of foragers returning with pollen, nectar or empty over time.

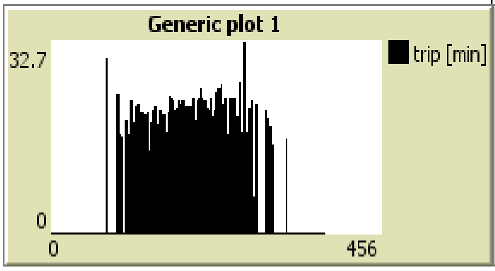

Figure 1.21: *Mean trip duration*. The daily mean duration [min] of all foraging trips over time.

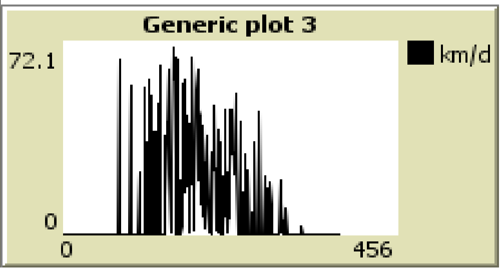

Figure 1.22***:*** *Mean total km per day*. The aily mean flight distances [km/d] of foragers over time.

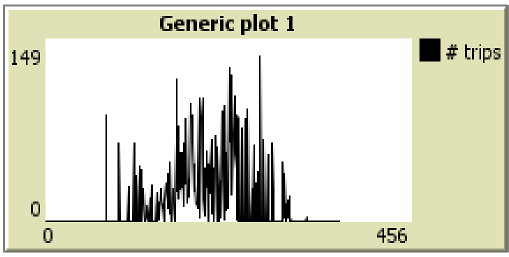

Figure 1.23: *Number of completed foraging trips* (E-3). The number of completed daily foraging trips divided by 10^3 over time.

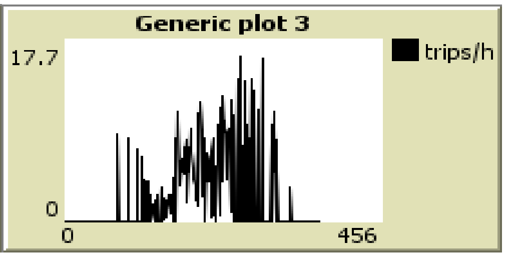

Figure 1.24: *Trips per hour sunshine (E-3)*. The daily number of foraging trips per hour sunshine divided by 10^3.

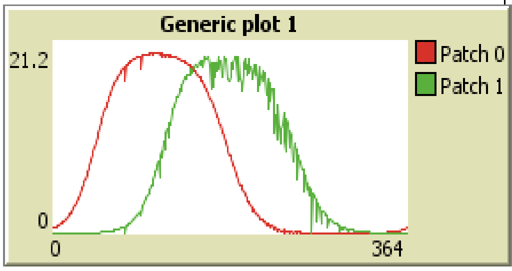

Figure 1.25: *Nectar availability [l]*. The availability of nectar [l] either at the two default patches or summed up for all patches over time.

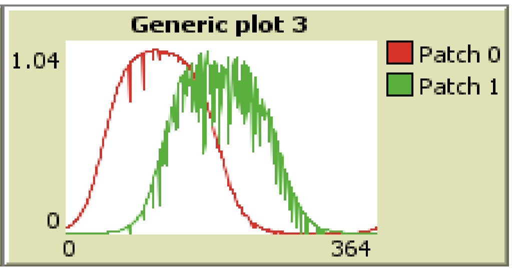

Figure 1.26: *Pollen availability [kg]*. The availability of pollen [kg] either at the two default patches or summed up for all patches over time.

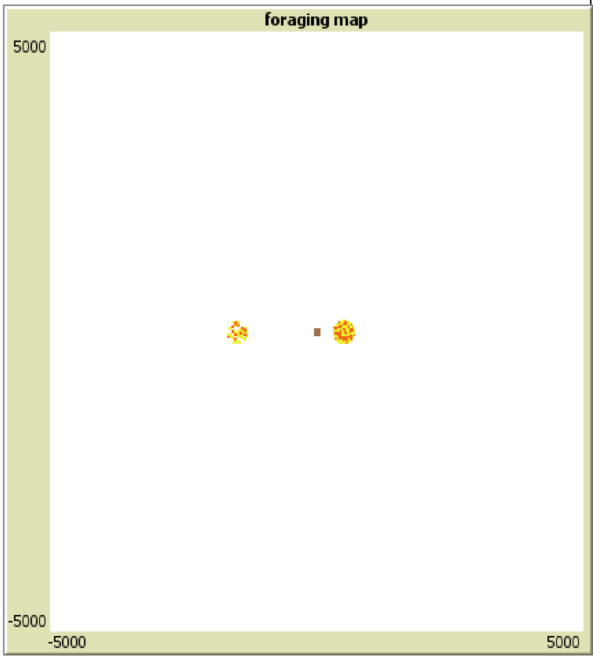

Figure 1.27: 'Foraging map' – *Nectar and Pollen.* The map shows the foraging of nectar and pollen at the available patches on that day. Yellow / orange dots refer to nectar / pollen visits of a forager squadron.

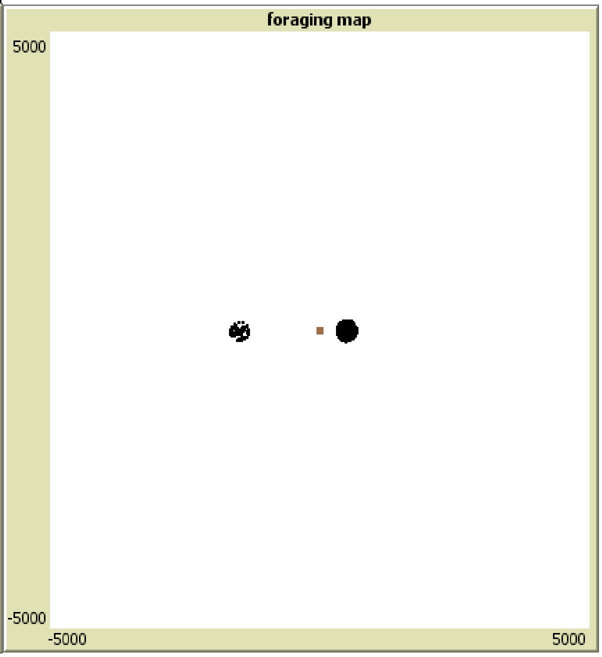

Figure 1.28: 'Foraging map' – *All visits.* All visits of forager squadrons at available patches providing pollen and nectar on the current day are shown.

1.3.2 'Output monitor'

The integrated monitors of the BEEHAVE user interface display the most important variables of the actual colony composition and stores, foraging trips and infestation by *Varroa* mites on that day (table 1.7). At the beginning of the model run starting on the 1st January with an initial colony size of 10,000 worker bees, all these bees are defined as foragers with an average age of 130 days. Hence, the total number of workers (*# workers*) is identical to *# foragers*, until the first cohort of in-hive bees emerges (fig. 1.30).

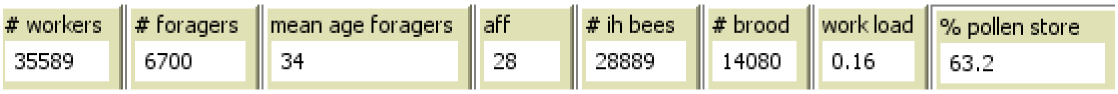

Figure 1.30: Output monitors display the actual colony composition and stores on day 31 (above) and 221 of model run.

Note: The abrupt, distinct decrease in the average age (*mean age foragers*) illustrates the extinction of the long-living foragers (surviving winter bees) and thus the emergence of newly foragers attaining the age of first foraging (*aff*).

Table 1.7: Definition of important output variables of the BEEHAVE modules *Colony*, *Foraging* and *Varroa* displayed by a monitor on the current day

**Output variable**	**Definition**	**Module**
# workers	total number of in-hive and foraging worker bees	Colony
# foragers	number of foragers	Colony
mean age foragers	mean age of foraging bees [d]	Colony
aff	current age of first foraging [d]	Colony
# ih bees	number of in-hive workers on that day	Colony
# brood	total number of brood cells (eggs, larvae and pupae of drones and worker bees)	Colony
work load	relative amount of brood to maximum nursing force	Colony
pollen store [%]	current pollen store relative to the ideal amount of pollen [%]	Colony
nectar visits	number of nectar foraging trips	Foraging
pollen visits	number of pollen foraging trips	Foraging
Julian Day	current day of the year	-
Day	approximate current date of model simulation run	-
Month		
Year		
Followers R	mean number of dance followers for the red patch	Foraging
Followers G	mean number of dance followers for the green patch	Foraging
total mites	number of healthy and infected mites (phoretic and in cells)	Varroa
phoretic mites healthy	number of healthy phoretic mites	Varroa
phoretic mites INFECTED	number of virus-infected phoretic mites	Varroa
mites in cells	number of mites in brood cells	Varroa
rate healthy foragers	proportion of foragers that are not virus infected	Varroa
rate healthy mites	proportion of uninfected phoretic mites	Varroa
mite fall	number of dropped mites	Varroa
pollen store [kg]	store of pollen [kg]	Colony
honey store [kg]	store of honey [kg]	Colony
trips/h	number of foraging trips per hour	Foraging

1.4 World of BEEHAVE

The world panel of BEEHAVE shows colour-contrasting age-based cohorts of drones and worker bees representing the colony structure on that day. Moreover, forager squadrons highlighted in colour and many additional symbols illustrate the foraging activities as well as the current status of the brood and colony stores.

After three days of the egg stage (blue), worker and drone larvae hatch. The larval stage (yellow) lasts 6 days for worker and 7 days for drone larvae until the cells are sealed. And finally, after finishing the pupal stage (brown) of 12 days for worker bees and 14 days for drones the adult workers (orange) and drones (grey) emerge from the capped cells requiring a total developmental time of 21 days for the immature worker stages and 24 days for the drones. The total number of individuals in each daily age-cohort is displayed.

Also, the virus infection is represented by colour nuances of the adult bee cohorts of workers and drones. Hence, the world of BEEHAVE allows you to distinguish between healthy (worker: orange, drones: grey), workers infected as adults (dark orange) and infected as pupae (worker: red brown, drone: dark grey) bee cohorts (figure 1.31).

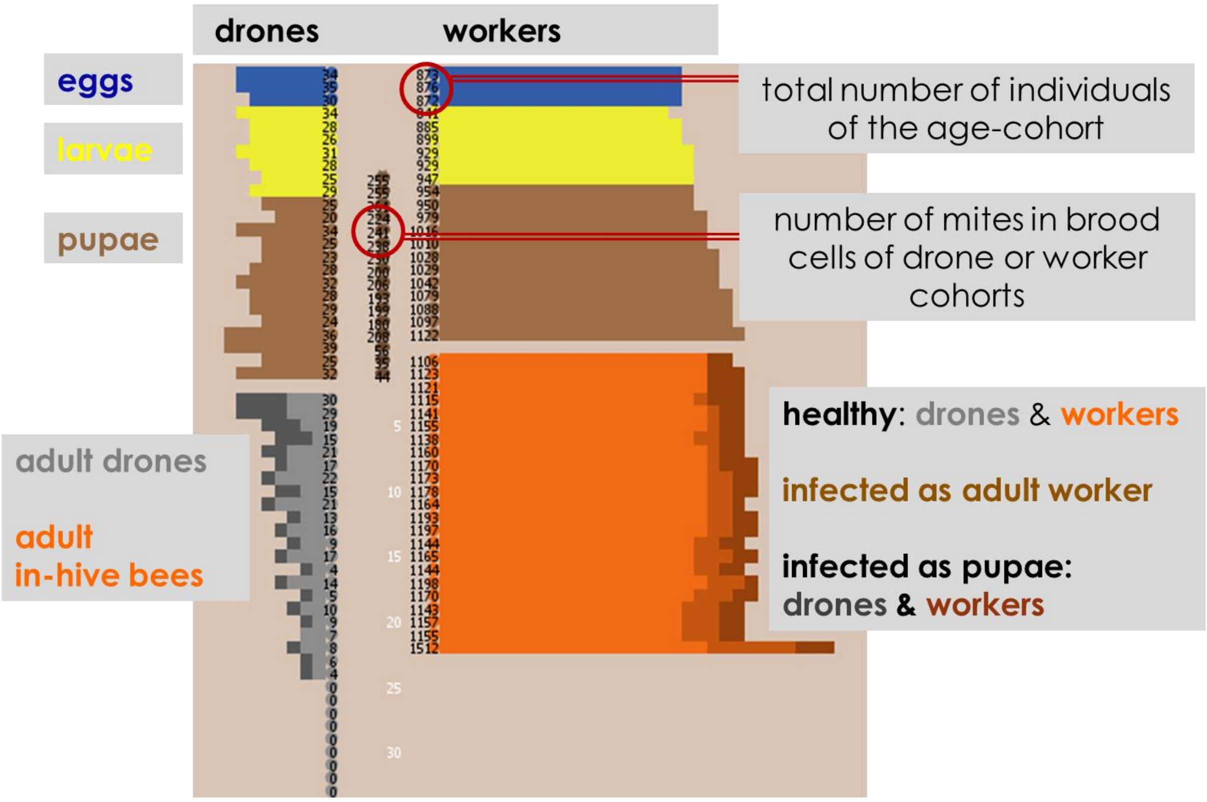

Figure 1.31: Overview of the age-based cohorts of the honeybee colony.

In addition, forager squadrons are divided into healthy, infected as adults and infected as pupae, whereby all these squadrons are moved to the right when they ageing. Moreover, successful foragers assume the colour of their last visited flower patch, while scouts and inactive bees are shown in grey.

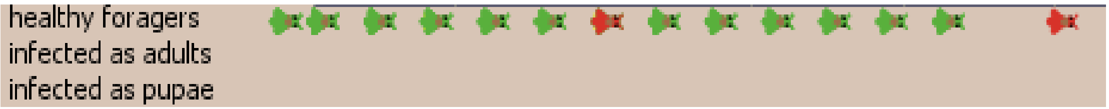

The current time step of the simulation run is shown by a tick counter. Hence, the model proceeds in daily time steps represented by ticks.

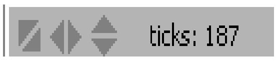

However, the BEEHAVE world provides several additional symbols that illustrate the foraging opportunities, the current state of the brood, the infestation of immature stages by mites and also the colony stores as well as applied beekeeping treatments.

Thus, the positioning of the '*red*' and '*green flower*' to the left and right of the hive represents the distances of the nectar- and pollen-providing flower patches to the colony. If the mapped flowers are depicted as withered no further nectar and pollen is supplied by these patches.

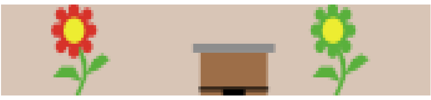

In addition, local weather conditions strongly influence flight activities of scouting and foraging bees on that day. Hence, the following weather symbols indicate the duration of the foraging period on that day.

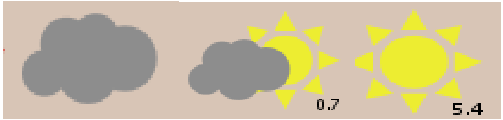

'Cloud' indicates that the local weather conditions do not support foraging trips on that day. Furthermore, *'Sun & Cloud'* indicate a possible foraging duration of up to 4 hours, and *'Sun'* symbolizes a possible daily foraging duration of more than 4 hours.

Besides, changes in the colony's pollen (left) and honey (right) stores in comparison to the day before are represented on that day using *'arrows'* which indicate either a reduction (red) or stocking up (green) of the colony stores, with numbers representing the amount of change [kg].

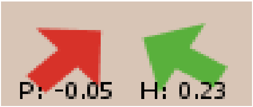

The *'exclamation mark'* is shown on days, when then foraging probability is set to zero, which may happen even if the weather is suitable for foraging.

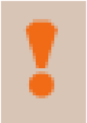

Death of the brood due to lack of pollen or nurse bees is a further important indicator of the current colony state and is displayed as a symbol.

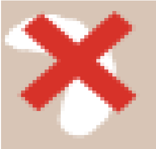

Moreover, beekeeping options like honey harvesting, feeding bees, stocking up colony stores, merging weak colonies and treatment of invaded *Varroa* mites (not included in the **Default** setting) may also be very important drivers of colony dynamics.

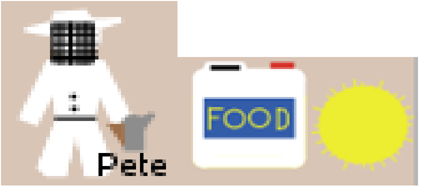

Thus, a '*Beekeeper*' represents the onset of at least one of the following beekeeping options. Now, honey or pollen stores are added to the colony store ('*food*' or '*pollen grain*').

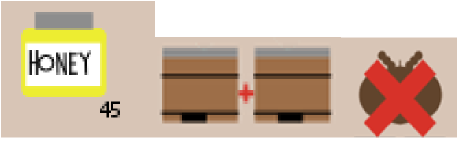

Additionally, the beekeeping symbol '*Honey*' indicates the harvest of honey during the pre-defined harvesting period regarding the defined threshold of honey stores and the amount of honey [kg] harvested.

Bees can be added to a weak colony in autumn if the current colony size is below a defined threshold value ('*2 hives*'). Furthermore, treatments of *Varroa* mites take place in autumn ('*Varroa*').

In the end, if the colony size falls below the threshold of the critical colony size during winter, the honey stores are below zero or all bees have died, then the death of the honeybee colony is displayed by the '*skull*'.

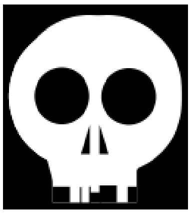

2 Guided Tour

In this section we will demonstrate your first pre-defined BEEHAVE scenarios with step-by-step instructions.

2.1 The Default scenario

1. Open the file “Beehave2013.nlogo”.
2. Type in the input field ‘RAND_SEED’ 8 and press the **Default** button.

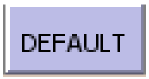

> **Note:** The choice of this pre-defined scenario resets all input variables to their default values (table 2.1) and requires 2 feeders (“red” and “green” patch), seasonal food flow as well as local weather data at Rothamsted from 2009. In the **Default** scenario beekeeping options and *Varroa* infestation are turned off.

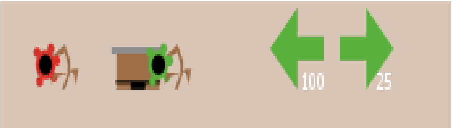

However, the food sources (‘*red*’ and ‘*green flower*’) providing nectar and pollen have different distances to the hive. The withered flowers indicate that the existing patches offer no nectar and pollen to foraging bees at the moment whereas the arrows display the initial stores of pollen (left: 100 g) and honey (right: 25 kg) of the colony. 10,000 long-living winter bees, here referred to as foragers, constitute the initial colony size (figure 2.1).

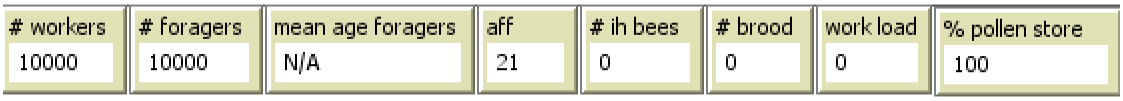

Figure 2.1: Initial colony size, age of first foraging (*aff*), and amount of brood and pollen stores of the implemented honeybee colony. Please note, the number of workers (*# workers*) sums up the number of in-hive bees and foragers, and thus the initial number of workers *(# workers*) is only composed of 10,000 long-living foragers (*# foragers*) (surviving winter bees).

Table 2.1: This table gives an overview of all default initial settings. The meaning of the input parameters is explained in the User Manual (see above) and the ODD model description.

**Input variable**	**Initial setting**	**Input variable**	**Initial setting**
'Input fields'		***Input 'Switch'***	
RAND_SEED	8	HoneyHarvesting	False
N_INITIAL_BEES	10000	FeedBees	False
QUANTITY_R_l	20	MergeWeakColonies	False
QUANTITY_G_l	20	AddPollen	False
CONC_R	1.5	VarroaTreatment	False
CONC_G	1.5	SeasonalFoodFlow	True
POLLEN_R_kg	1	ConstantHandlingTime	False
POLLEN_G_kg	1	AlwaysDance	False
DISTANCE_R	1500	EggLaying_IH	True
DISTANCE_G	500	QueenAgeing	False
DETECT_PROB_R	0.2	HoneyIdeal	False
DETECT_PROB_G	0.2	PollenIdeal	False
N_INITIAL_MITES_HEALTHY	0	ReadInfile	False
N_INITIAL_MITES_INFECTED	0	ShowAllPlots	True
TIME_NECTAR_GATHERING	1200	stopDead	True
TIME_POLLEN_GATHERING	600	modelledInsteadCalcDetectProb	False
SHIFT_R	30	Details	True
SHIFT_G	-40	writeFile	False
DANCE_SLOPE	1.16		
DANCE_INTERCEPT	0	***Input 'Chooser'***	
MAX_BROODCELLS	2000099	Weather	Rothamsted (2009)
MAX_HONEY_STORE_kg	50	Swarming	No swarming
CRITICAL_COLONY_SIZE_WINTER	4000		
MAX_km_PER_DAY	5099		
ProbLazinessWinterbees	0		
SQUADRON_SIZE	100		

Note: Further pre-defined scenarios can be chosen using the buttons **2 patches**, **1 feeder**, **RRes**, **varroa** or **beekeeping** that only change a few input variables. Subsequently, we will perform the **varroa** scenario as the second pre-defined example.

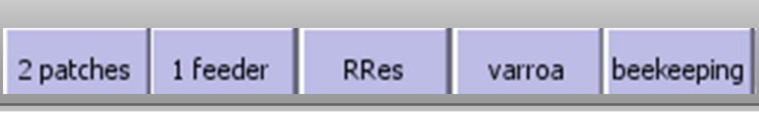

3. There are eight "generic plots" where, via choosers, you can select what you want to plot. Please select the following eight outputs by selecting the corresponding choosers, in the given sequence, for the generic plots 1-8: *egg-laying*, *Colony structure workers*, *brood care [%]*, *foragers today [%]*, *aff & lifespan*, *pollen availability [kg]*, *nectar availability [l]*, and *loads returning foragers [%]*.
   Furthermore, choose the option 'Nectar and Pollen' from the 'Foraging map' chooser.

4. Press the **Setup** button. 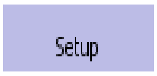

5. Now, let's take a closer look at those 'output monitors' that offer an easy way to see how the most important properties of the system change as the model runs. Firstly, in order to recall the definition of the several displayed variables **click the right mouse button** on any 'output monitor' and then click **Edit**. The dialog window of the monitor appears; here, the monitors of four outputs, referred to their input field "Display Name", are shown:

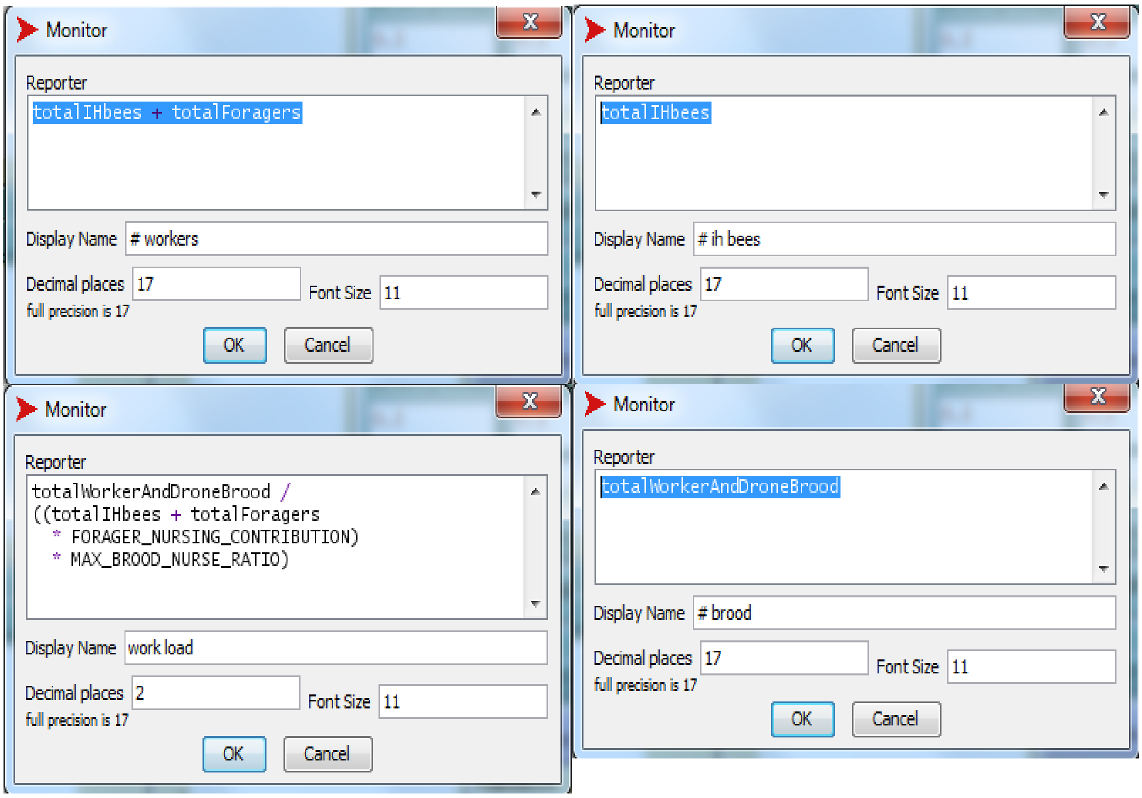

The monitor windows show the displayed name of the output variable and the instruction for computing their daily value (called 'Reporter' in NetLogo). Thus, the total daily number of workers (*# workers*) sums up the total number of in-hive bees and foragers (*totalIHbees* + *totalForagers*) on that day. The total amount of brood (*# brood*) is composed of the total number of worker and drone brood cells (*totalWorkerAndDroneBrood*) on the current day.

The daily workload of the colony is a function of the total amount of brood cells and the total number of working bees in association with the contribution of foraging to nursing bees and the ratio of brood cells to nursing bees (*work load*).

6. Now, start the model run by clicking the **1 Day** button. On the top left of the interface the in-hive cohorts and forager squadrons, and several ‘output monitors’ illustrating the current colony status are displayed.

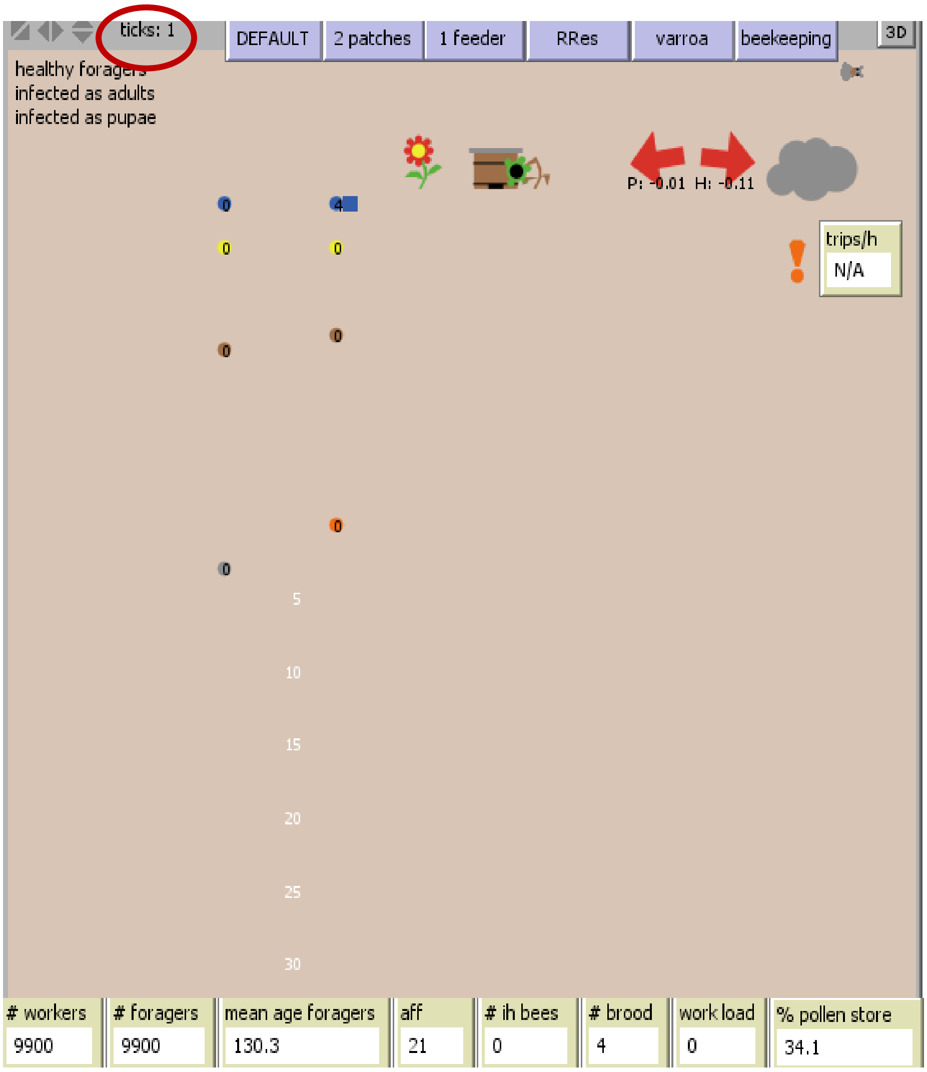

Figure 2.2: Colony composition, honey and pollen stores and also weather conditions on day 1 of simulation run.

At this initial state of the simulation the actual total number of working bees of the colony is only composed of the number of long-living foragers (winter bees) with an average age of 130.3 days (*mean age foragers*); remember, we are on day one of model run (see tick counter ‘ticks’ on the top left). The ‘output monitor’ *# ih bees* and the orange-colored age-cohorts (orange bars) on the left panel illustrate that no new in-hive bees emerge on that day.

Moreover, four worker eggs are laid by the queen (blue bar), but no age-cohorts of larvae (yellow bar) and pupae (brown bar) are developed during the current day. Thus, the daily total colony's amount of immature stages is only composed of four brood cells (*# brood*). The actual relative amount of brood to maximum nursing force (*work load*) amounts to zero on that day. Compared to the day before the stores of honey and pollen decreases (*red arrows*: left: pollen; right: honey). The current pollen store only exceeds 34.1 % of the ideal pollen store (*% pollen store*). In addition, the actual weather conditions allow no foraging trips as represented by the '*Cloud'* (figure 2.2).

Nevertheless, one scouting forager squadron leaves the hive (grey bee). But only one patch '*red flower*' provides nectar on that day. In contrast, the withered '*green flower*' symbolizes no pollen and nectar provision by the green patch.
Thus, we will check the food availability of the existing patches and also the current visits at the flower patches by clicking the advanced inputs (bottom right of interface):

visited patches and active patches.

Now, click 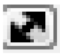 of the 'Command Center' to enlarge its window. The following information of the available patches is printed out by the 'Command Center':

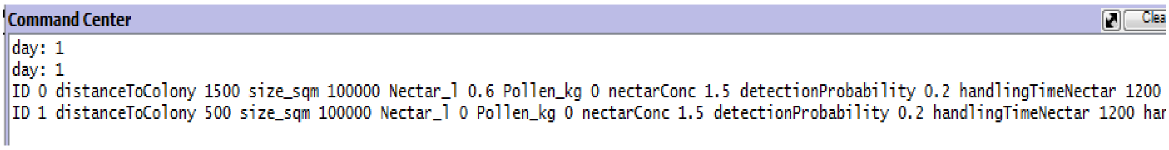

Visited patches (first two lines): No visits at flower patches on that day
Active patches (third and fourth line): Provided nectar & pollen of patches on that day

Hence, only the '*red flower*' patch offers very low amounts of nectar. Moreover, neither flower patch is visited by any forager squadron.

Note: Minor deviations of several output values may result from the application of different operating systems, different NETLOGO versions or the selection of different random seeds.

7. Click the **1 Day** button again.

Compared to the day before, five new worker eggs are laid and the four eggs laid on the previous day have been shifted to the second age-cohort of the egg stage (blue bars). But still no larvae (yellow bars), pupae (brown bars) and newly emerged in-hive bee (orange bars, *# ih bees*) cohorts exist on the current day. Furthermore, the total number of working bees is still composed of long-living foragers (*# foragers*: 9800, *mean age foragers*: 131.2 days). Only one flower patch offers low quantities of nectar today and local weather conditions prevent foraging trips on that day (*'Cloud'*). Therefore, no nectar and pollen visits take place (*Nectar visits*, *Pollen visits*) as shown in figure 2.3.

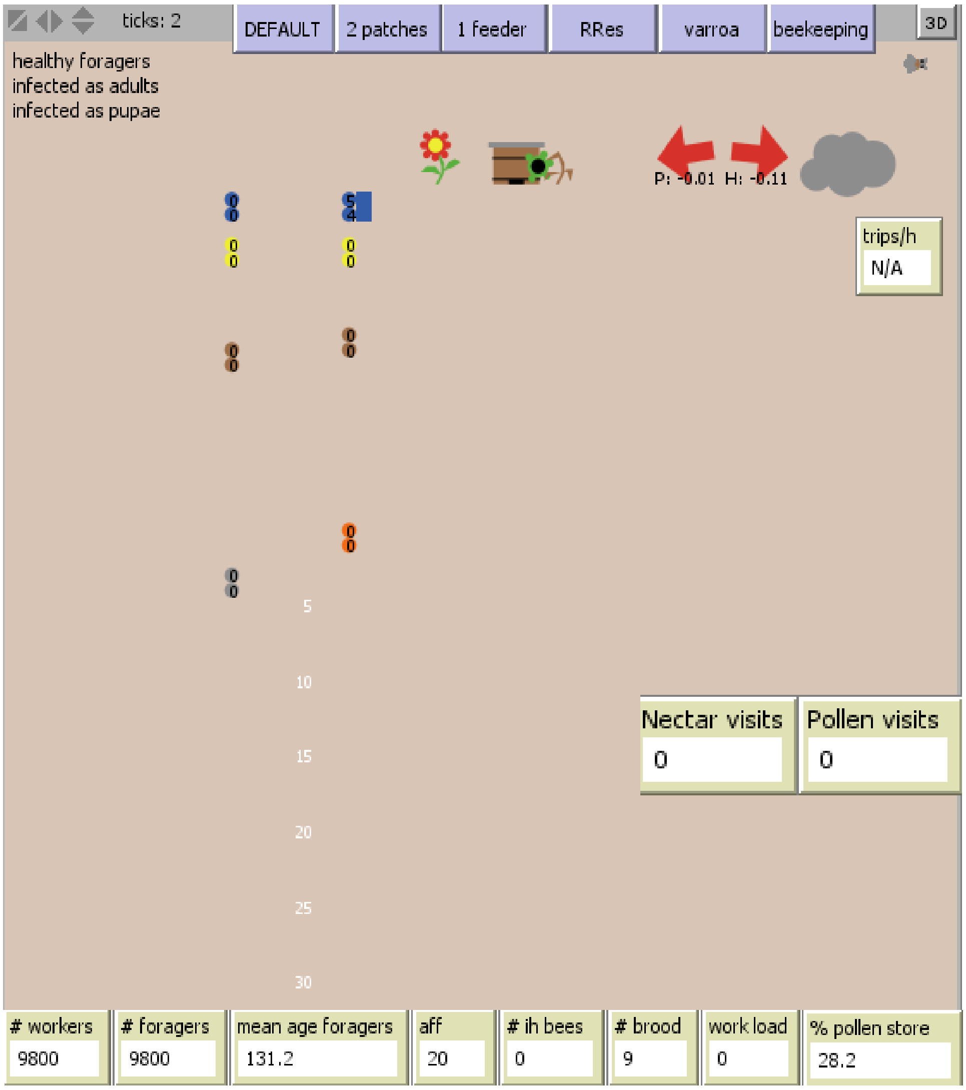

Figure 2.3: The colony structure and stores as well as conditions for foraging and performed nectar and pollen visits by foragers at the 2nd day of simulation run are represented. Note that here the output monitors Nectar and Pollen visits have been replaced; in the program, they are attached to the foraging map.

8. Click the **1 Day** button several times on the trot until the 'output monitor' *Julian Day* displays day '7' (tick counter). Now, press the advanced button **active patches**.

The *egg-laying* rate ('Generic plot 1') and also the number of brood cells (*# brood*) increases. Furthermore, the first age-cohort of the larval developmental stage (yellow bars) hatches, but no pupae (brown bars) and in-hive bee cohorts (orange bars) emerge. At this time point no drone brood cells have developed (bars of age-cohorts on the left side of the panel). The 'output monitors' display the increase in the mean age of foragers, the decrease in the age of first foraging (*aff*) and a lack of pollen stores *(% pollen store*). In addition, the amount of nectar provided by the '*red flower*' patch increases, but the '*green flower*' patch still does not offer nectar rewards ('Command Center', 'Generic plot 7'). Insufficiently good weather conditions symbolized by the '*Cloud*' inhibit foraging trips on that day. Therefore, honey and pollen stores decrease compared to the day before (*red arrows*).

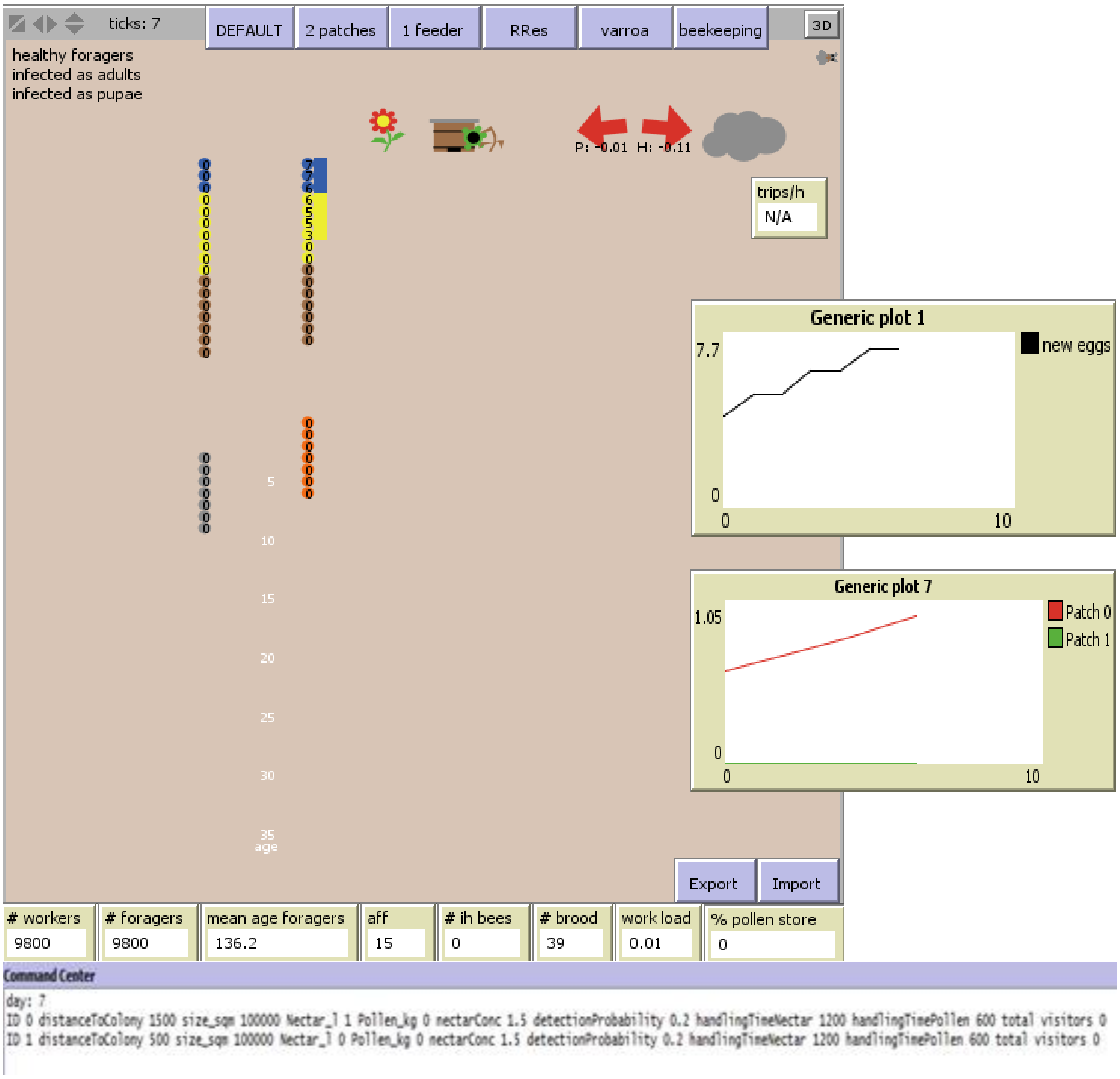

Figure 2.4: Age-cohorts of immature stages and adult bees, colony stores, actual forager squadrons and foraging conditions on day 7 are illustrated by the world panel of BEEHAVE. In addition available nectar rewards ('Generic plot 7') and egg-laying rate of the honeybee queen ('Generic plot 1') until day 7 are represented.

9. Next, click the **1 Month** button twice. The *Julian Day* output monitor displays day '67' of model run.

Now, take a look at the nectar and pollen amounts provided as well as the energetic efficiency and visitors of the available patches by clicking **show Patches** and **active patches.**

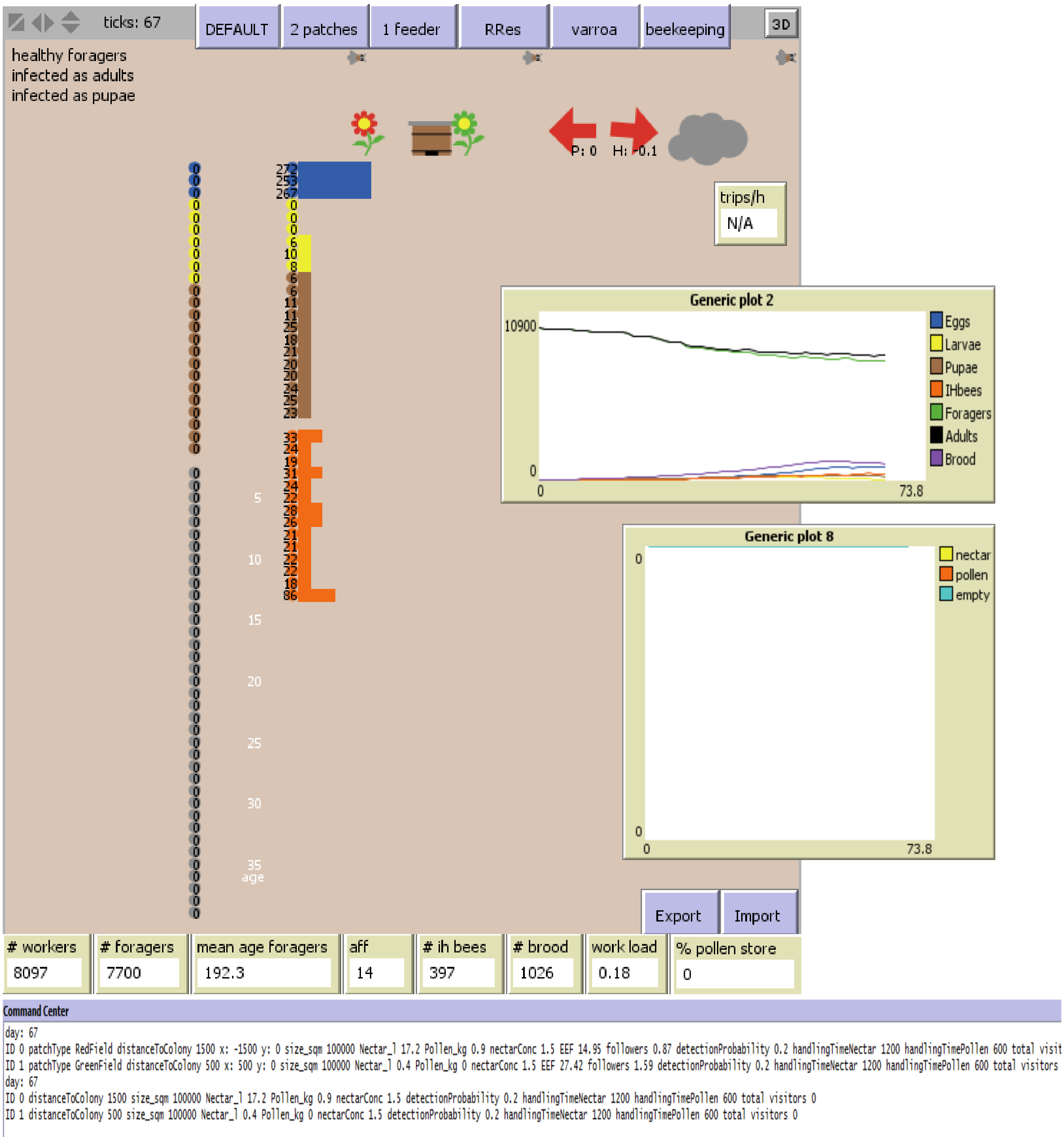

Figure 2.5: Composition, stores, and foraging conditions on day 67. The development of immature and adult stages of worker and drones as well as nectar and pollen loads of returning foragers from starting day until day 67 of simulation run is shown by the 'Generic plots' and prints of the 'Command center'.

As illustrated in figure 2.5, on the current day 272 new worker eggs are laid (first egg cohort on the right), but no drone eggs emerge (first egg cohort on the left). In total 397 in-hive bees hatched from the last immature stages (*# ih bees*) within 60 days starting from day seven. At

this point of time the total number of workers is now composed of long-living foragers and newly emerged in-hive bees. The number of foragers (*# foragers*) decreases ('Generic plot 2'), at the same time the *mean age of foragers* increases. Moreover, with increasing amount of brood cells the colony's *work load* increases. However, until day 67 of the model run no forager squadrons performed foraging trips, only a few scouting squadrons are active on the current day. Hence, no forager squadrons visit any of the available patches ('Command center') or returned to the hive loaded with pollen and nectar ('Generic plot 8'); both patches provide nectar rewards but only the '*red flower*' patch offers small amounts of pollen on that day ('Command Center'). So, the pollen stores of the hive are empty and the honey stores further decrease compared to the day before (*red arrows*).

10. Now, we want to inspect several age-cohorts of the developmental stages of the in-hive bees by using the agent monitors of Netlogo. **Click the right mouse button on the first egg age-cohort and pupae age-cohort** (the circle with a label on it; it is next to the bar indicating the size of this cohort). Next, choose **egg-cohort 649** (and **pupae-cohort 653**) and click **inspect egg-cohort 649** (and **inspect pupae-cohort 653**).

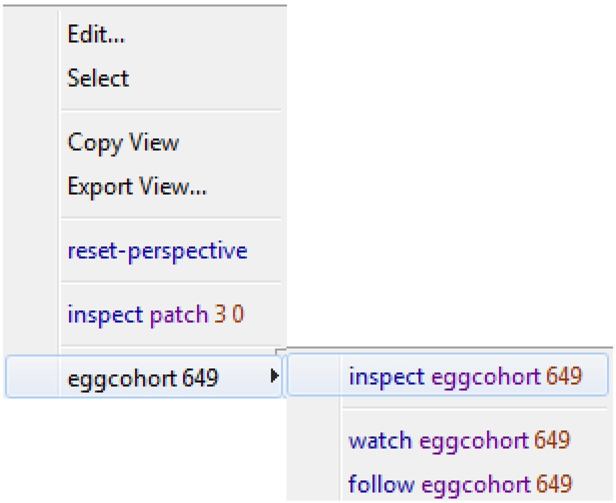

The window of the egg-cohort appears and displays the cohort ID (*who*), the *ploidy* level and *age* of the egg-cohort as well as the number of individuals in this age-cohort (*number*). Now close these monitors.

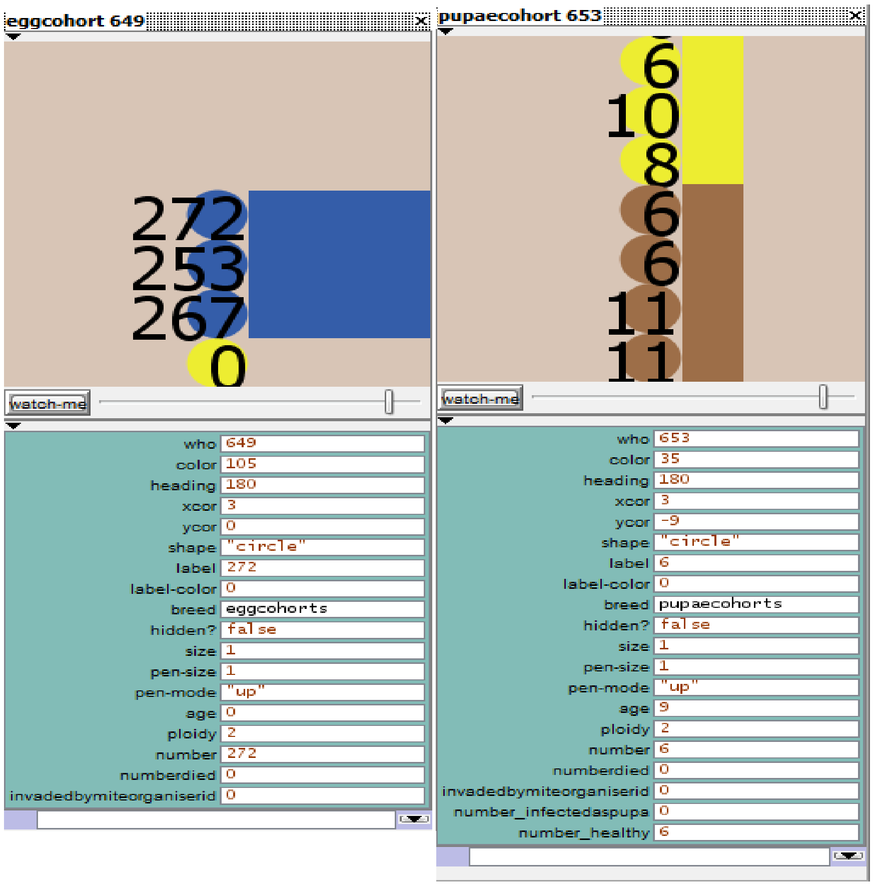

11. Click the **1 Month** button twice, again. Now, the model run should attain day '127'. Moreover, we want to check the foraging activities of all forager squadrons on that day, so click the advanced buttons **ActivityList** (see button on bottom right of the interface) and **visited patches**.

You can consider many changes in the colony dynamics from day 67 to day 127 (figure 2.6, 'Generic plot 2'). The total number of adult bees *(# workers*) decreases due to the distinct decline of long-living foragers (*# foragers*) attaining a *mean age foragers* of 231.7 days. Moreover, the total number of brood cells (*# brood*) including developmental stages not only of workers but also drones, the workload ('Generic plot 3') and also the number of emerged in-hive workers and their age of first foraging increases during this period of time ('Generic plot 5'). Both available patches offer pollen and nectar on that day ('Command Center' of fig. 2.7). But forager squadrons mainly perform pollen and nectar visits at the near-distance '*green flower*' patch (look at the values for visitors of patch 0 and 1 displayed by the

'Command Center'). Weather conditions allow foraging trips within a time interval of 3.2 hours on that day ('*Sun & Cloud'*). Moreover, pollen loads (5000) are mainly cropped by the forager squadrons and only few nectar rewards (1300) are collected on that day (fig. 2.6: output monitors *Nectar visits* and *Pollen visits* attached to the 'Foraging map', 'Generic plot 4 & 8'; 'Command Center' in fig. 2.7). Nevertheless, 'Generic plot 8' also illustrates the loads of returning foragers from day 1 to day 127 of simulation run; thereby crops of pollen and nectar collected by active forager squadrons were brought to the hive within the last days.

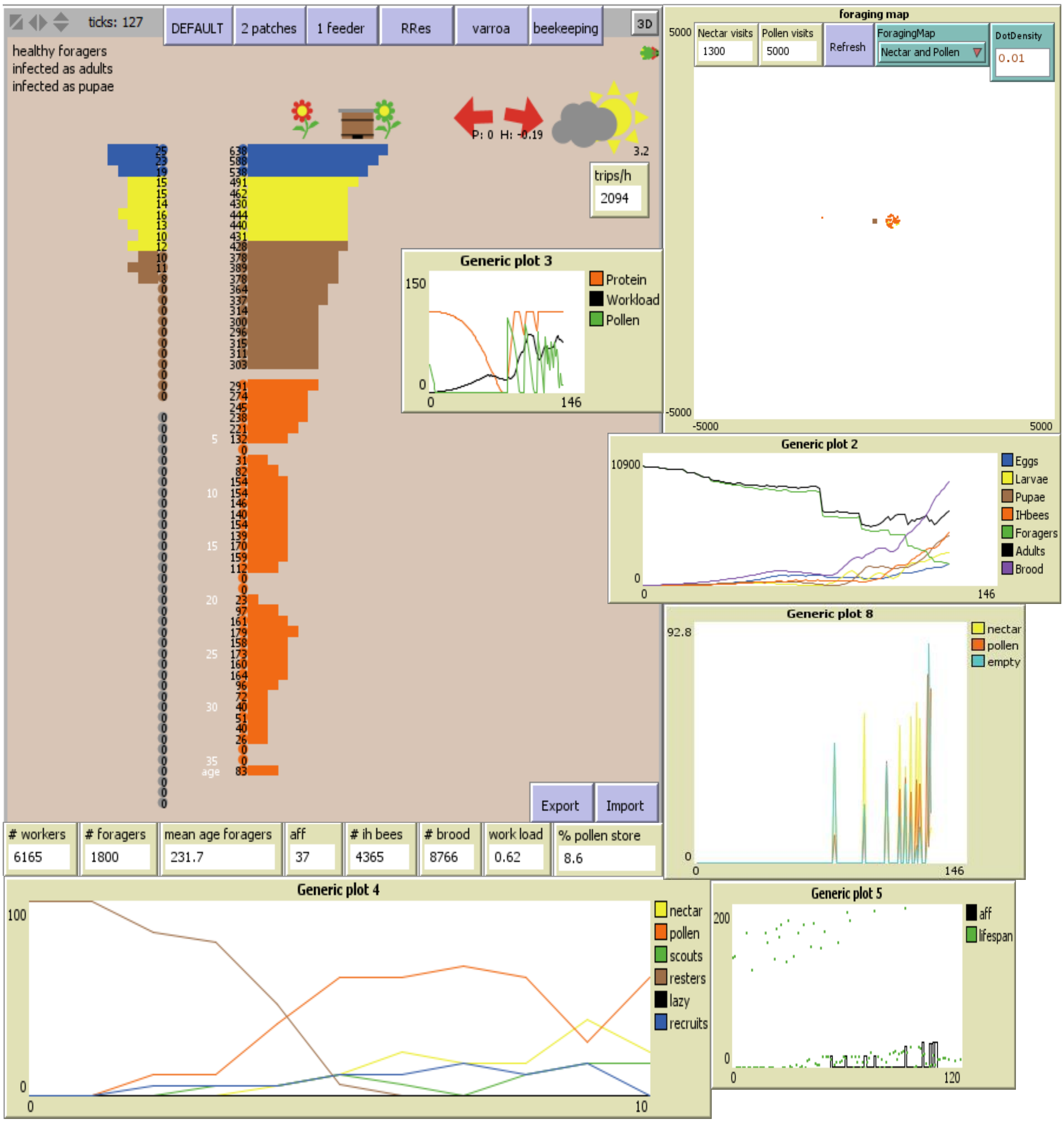

Figure 2.6: Colony structure and hive stores, brood care and foraging trips at the current day 127.

Now, try to examine all activities of at least one forager squadron over all foraging rounds during the day (hint: see table 1.2).

The activity list of forager squadrons during the day shows the diurnal foraging activities of the 18 active forager squadrons (fig. 2.7: ‘Command Center’). The first two columns of one line, which represents one forager squadron, indicate the squadron ID and the daily distance travelled by the respective forager squadron. The actual activities of the forager squadron per foraging round are put in brackets. Here, the foraging activities of one forager squadron during all foraging rounds on the day may include resting (R), recruitment to an advertised flower patches by dancing bees (rFnNF / rFpPF), successful returning to the hive loaded with nectar and pollen (bN/ bP), scouting (Sn / Sp) for a nectar or a pollen patch, dancing for a sufficient nectar and pollen source (Dn / Dp) or abandonment of a patch (An / Ap).

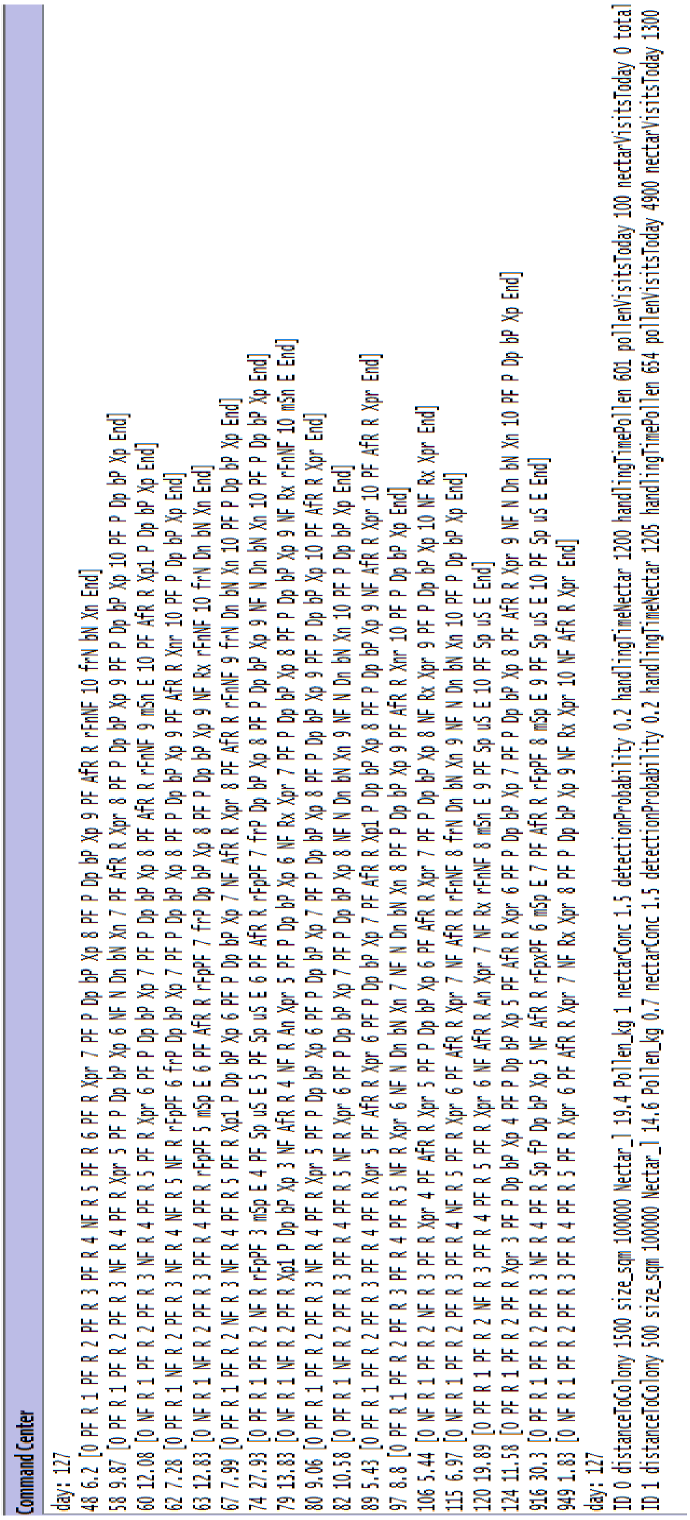
Command Center
day: 127
48 6.2 [0 PF R 1 PF R 2 PF R 3 PF R 4 NF R 5 PF R 6 PF R Xpr 7 PF P Dp bP Xp 8 PF P Dp bP Xp 9 PF AfR R rFnNF 10 frN bN Xn End]
58 9.87 [0 PF R 1 PF R 2 PF R 3 NF R 4 PF R Xpr 5 PF P Dp bP Xp 6 NF N Dn bN Xn 7 PF AfR R Xpr 8 PF P Dp bP Xp 9 PF P Dp bP Xp 10 PF P Dp bP Xp End]
60 12.08 [0 NF R 1 PF R 2 PF R 3 NF R 4 PF R 5 PF R Xpr 6 PF P Dp bP Xp 7 PF P Dp bP Xp 8 PF AfR R rFnNF 9 mSn E 10 PF AfR R Xp1 P Dp bP Xp End]
62 7.28 [0 PF R 1 NF R 2 PF R 3 NF R 4 NF R 5 NF R rFpPF 6 frP Dp bP Xp 7 PF P Dp bP Xp 8 PF P Dp bP Xp 9 PF AfR R Xnr 10 PF P Dp bP Xp End]
63 12.83 [0 NF R 1 NF R 2 PF R 3 PF R 4 PF R rFpPF 5 mSp E 6 PF AfR R rFpPF 7 frP Dp bP Xp 8 PF P Dp bP Xp 9 NF Rx rFnNF 10 frN Dn bN Xn End]
67 7.99 [0 PF R 1 PF R 2 NF R 3 NF R 4 PF R 5 PF R Xp1 P Dp bP Xp 6 PF P Dp bP Xp 7 NF AfR R Xpr 8 PF AfR R rFnNF 9 frN Dn bN Xn 10 PF P Dp bP Xp End]
74 27.93 [0 PF R 1 PF R 2 NF R rFpPF 3 mSp E 4 PF Sp uS E 5 PF Sp uS E 6 PF AfR R rFpPF 7 frP Dp bP Xp 8 PF P Dp bP Xp 9 NF N Dn bN Xn 10 PF P Dp bP Xp End]
79 13.83 [0 NF R 1 NF R 2 PF R Xp1 P Dp bP Xp 3 NF AfR R 4 NF R An Xpr 5 PF P Dp bP Xp 6 NF Rx Xpr 7 PF P Dp bP Xp 8 PF P Dp bP Xp 9 NF Rx rFnNF 10 mSn E End]
80 9.06 [0 PF R 1 PF R 2 PF R 3 NF R 4 PF R Xpr 5 PF P Dp bP Xp 6 PF P Dp bP Xp 7 PF P Dp bP Xp 8 PF P Dp bP Xp 9 PF P Dp bP Xp 10 PF AfR R Xpr End]
82 10.58 [0 PF R 1 NF R 2 PF R 3 PF R 4 PF R 5 NF R Xpr 6 PF P Dp bP Xp 7 PF P Dp bP Xp 8 NF N Dn bN Xn 9 NF N Dn bN Xn 10 PF P Dp bP Xp End]
89 5.43 [0 PF R 1 PF R 2 PF R 3 PF R 4 PF R Xpr 5 PF AfR R Xpr 6 PF P Dp bP Xp 7 PF AfR R Xp1 P Dp bP Xp 8 PF P Dp bP Xp 9 NF AfR R Xpr 10 PF AfR R Xpr End]
97 8.8 [0 PF R 1 PF R 2 PF R 3 PF R 4 PF R 5 NF R Xpr 6 NF N Dn bN Xn 7 NF N Dn bN Xn 8 PF P Dp bP Xp 9 PF AfR R Xnr 10 PF P Dp bP Xp End]
106 5.44 [0 NF R 1 PF R 2 NF R 3 PF R Xpr 4 PF AfR R Xpr 5 PF P Dp bP Xp 6 PF AfR R Xpr 7 PF P Dp bP Xp 8 NF Rx Xpr 9 PF P Dp bP Xp 10 NF Rx Xpr End]
115 6.97 [0 NF R 1 PF R 2 PF R 3 PF R 4 NF R 5 PF R Xpr 6 PF AfR R Xpr 7 NF AfR R rFnNF 8 frN Dn bN Xn 9 NF N Dn bN Xn 10 PF P Dp bP Xp End]
120 19.89 [0 PF R 1 PF R 2 NF R 3 PF R 4 PF R 5 PF R Xpr 6 NF AfR R An Xpr 7 NF Rx rFnNF 8 mSn E 9 PF Sp uS E 10 PF Sp uS E End]
124 11.58 [0 PF R 1 PF R 2 PF R Xpr 3 PF P Dp bP Xp 4 PF P Dp bP Xp 5 PF AfR R Xpr 6 PF P Dp bP Xp 7 PF P Dp bP Xp 8 PF AfR R Xpr 9 NF N Dn bN Xn 10 PF P Dp bP Xp End]
916 30.3 [0 PF R 1 PF R 2 PF R 3 NF R 4 PF R Sp fP Dp bP Xp 5 NF AfR R rFpxPF 6 mSp E 7 PF AfR R rFpPF 8 mSp E 9 PF Sp uS E 10 PF Sp uS E End]
949 1.83 [0 NF R 1 PF R 2 PF R 3 PF R 4 PF R 5 PF R Xpr 6 PF AfR R Xpr 7 NF Rx Xpr 8 PF P Dp bP Xp 9 NF Rx Xpr 10 NF AfR R Xpr End]
day: 127
ID 0 distanceToColony 1500 size_sqm 100000 Nectar_l 19.4 Pollen_kg 1 nectarConc 1.5 detectionProbability 0.2 handlingTimeNectar 1200 handlingTimePollen 601 pollenVisitsToday 100 nectarVisitsToday 0 total
ID 1 distanceToColony 500 size_sqm 100000 Nectar_l 14.6 Pollen_kg 0.7 nectarConc 1.5 detectionProbability 0.2 handlingTimeNectar 1205 handlingTimePollen 654 pollenVisitsToday 4900 nectarVisitsToday 1300

Figure 2.7: Activities of all active forager squadrons over all foraging rounds and nectar and pollen visits at the both flower patches on that day 127.

12. Click **1 Month**. Day '157' of simulation run should be displayed by the tick counter. Check the colony dynamics (panel, 'Generic plot 2') and stores as well as foraging activities.

Note, the number of foragers' (# foragers) increases, but at the same time the *mean age of foragers* decreases. The current foragers emerge from the newly developed in-hive bees attaining their age of first foraging (*aff*). Thus, forager squadrons are not mainly constituted of long-living foragers (winter bees) any more. Moreover, no foraging activities and thus no nectar and pollen visits are performed on that day due to bad weather conditions as displayed by the '*Could*' and 'output monitor' *trips/ h* (fig. 2.8).

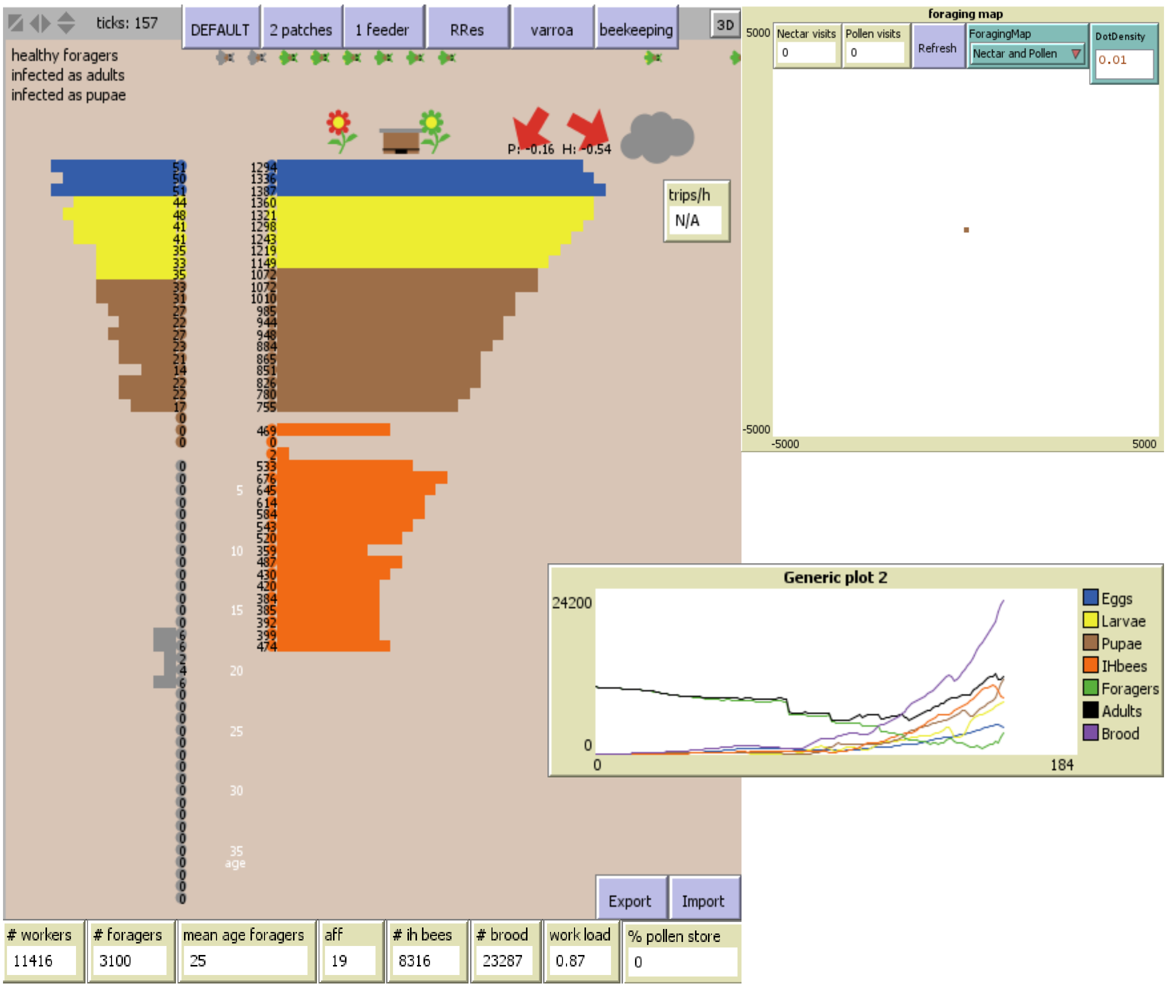

Figure 2.8: Composition and stores of the colony as well as foraging opportunities on day 157 are shown.

13. Before continuing our simulation example, we select the option '*All visits*' using the 'Foraging map' chooser in order to visualize all foraging trips of all active forager squadrons at the available patches.
    Now, click the **1 Month** button.
    Also, we want to take a look at the offered pollen and nectar amounts as well as the number of visits at the available flower patches on that day. Thus, click **visited patches**.
    Check the development of age-based cohorts and the current stores of the colony. Furthermore, compare nectar and pollen availability, and also the number of visits at the existing patches (fig. 2.9).

Foragers mainly visit the near-distanced 'green' patch and perform especially nectar visits on that day as displayed in the output monitor *Nectar visits*, 'Generic plot 4' (*foragers today [%]*, yellow line), 'Command Center' and the forager squadrons (*green bees*) of the world panel of figure 2.9. Furthermore the amount of brood *(# brood* = 27096) and the number of workers *(# workers* = 18611) and in-hive bees (*# ih bees* = 12611) increases compared to the month before (day 157), also illustrated by 'Generic plot 2'.

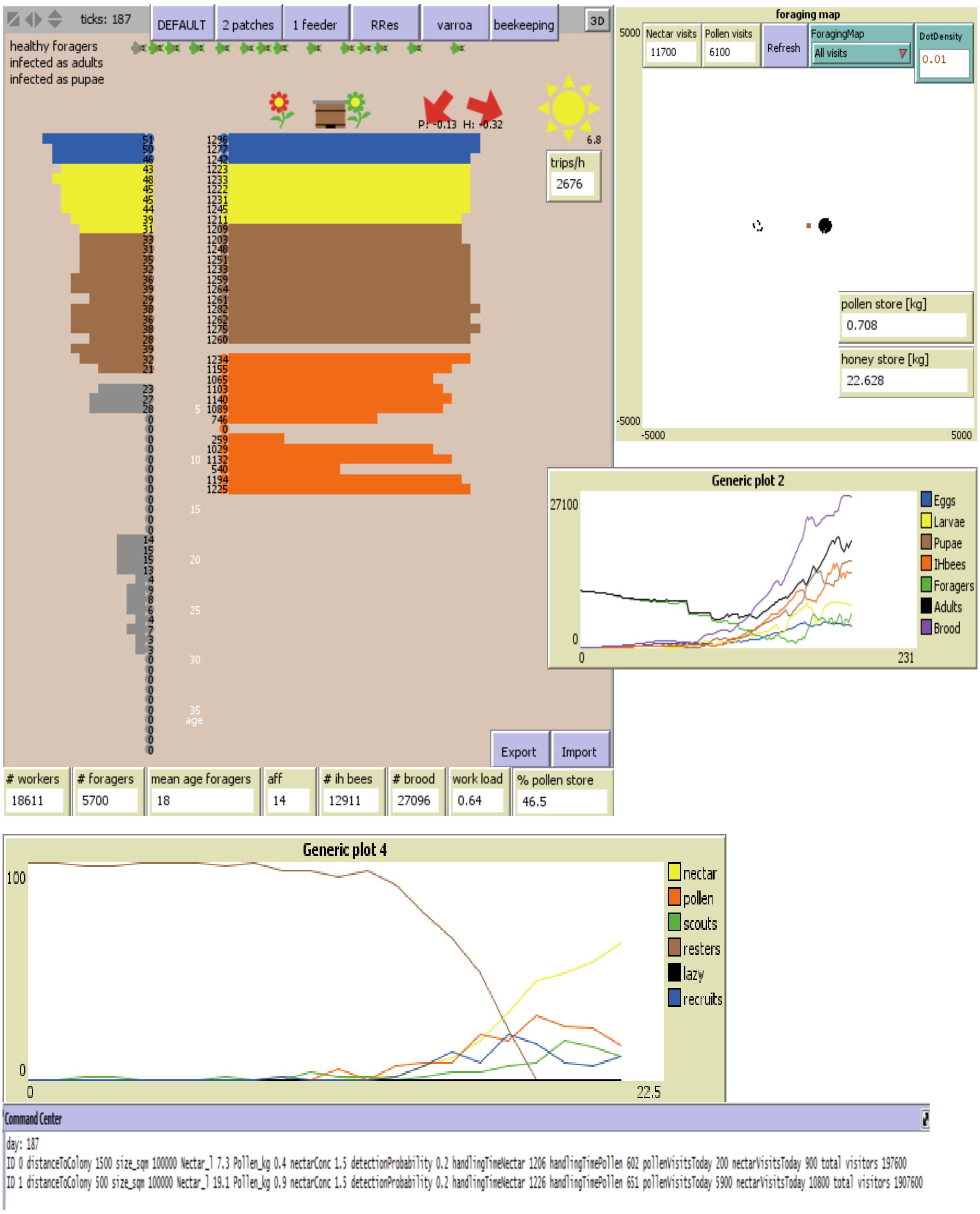

Figure 2.9: The colony structure and foraging trips on day 187 are illustrated.

14. Click **1 Month**. Now, the ‘output monitor’ *Julian Day* shows day ‘217`of model run. Again, consider the dynamics of the age-cohorts and the seasonal progress of egg-laying (fig. 2.10).

You will observe that the actual intra-colonial dynamics are characterized by a distinct decrease in the queen’s egg-laying activity (‘Generic plot 1’) as well as in the amount of brood (‘Generic plot 2’), and a considerably increase in the number of in-hive bees emerging from the previous immature stages (‘Generic plot 2’). Therefore, the workload resulting from the relative amount of brood to the maximum nursing force decreases as displayed by ‘Generic plot 3’ of figure 2.10. As illustrated by ‘Generic plot 5’ the age of first foraging increases and attains an actual value of 24 days (*aff*). Moreover, the pollen (‘Generic plot 6’) and nectar (‘Generic plot 7’) availability of the *‘red’* patch providing nectar and pollen early in the season strongly decreases, while the ‘*green*’ patch offers large amounts of rewards. In total 6746 foraging trips per hour (*trips/h*) due to suitable weather conditions, that allow a foraging duration of 5.9 hours (‘*Sun*’), are performed on that day.

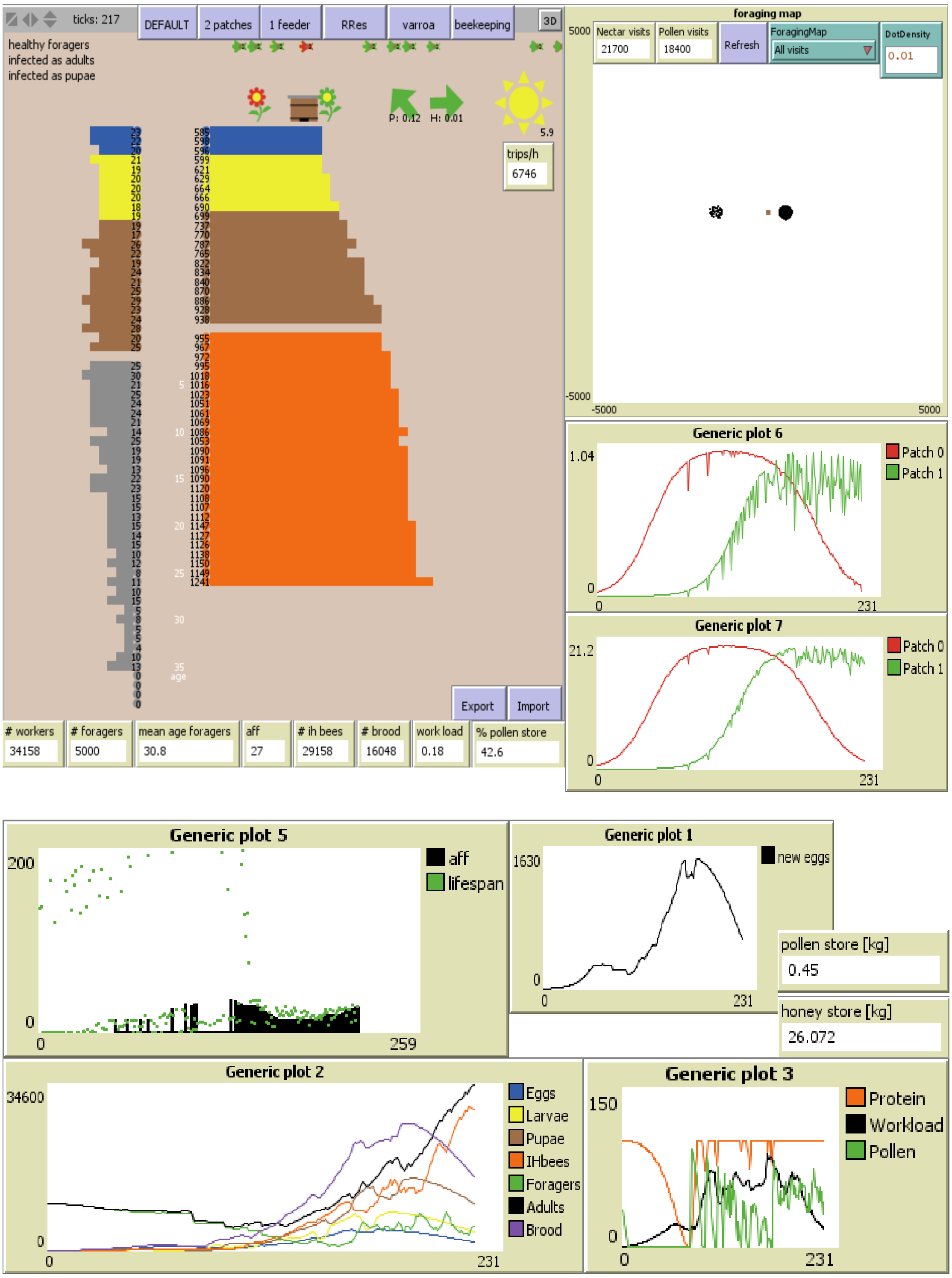

Figure 2.10: The colony structure, season-dependent pattern in queen's egg-laying and food availability as well as *brood care*, age of first foraging (*aff*) and number of performed foraging trips on day 217 are represented.

15. Next, click the **1 Month** button twice. Well, we should attain day '277' of our simulation run. Apply the advanced buttons **show Patches** and **visited patches** in order to recheck the provided nectar rewards, energetic efficiency (EEF) and forager visits of the available patches at the current day (fig. 2.11).

When you take a look at the intra-colonial dynamics you see a distinct decrease in the individual number of all age-cohorts as illustrated by 'Generic Plot 2' of figure 2.11. Although the current weather conditions are suitable for a foraging period of 4.9 hours on that day ('*Sun*'), no foraging trips are performed (monitors: *Nectar visits*, *Pollen visits* and *trips/h*, 'Command Center'), because the foraging probability is set to zero as illustrated by the '*exclamation mark*'. Furthermore, only the '*green*' patch flowering later in the season actually offers nectar and pollen rewards ('Command Center').

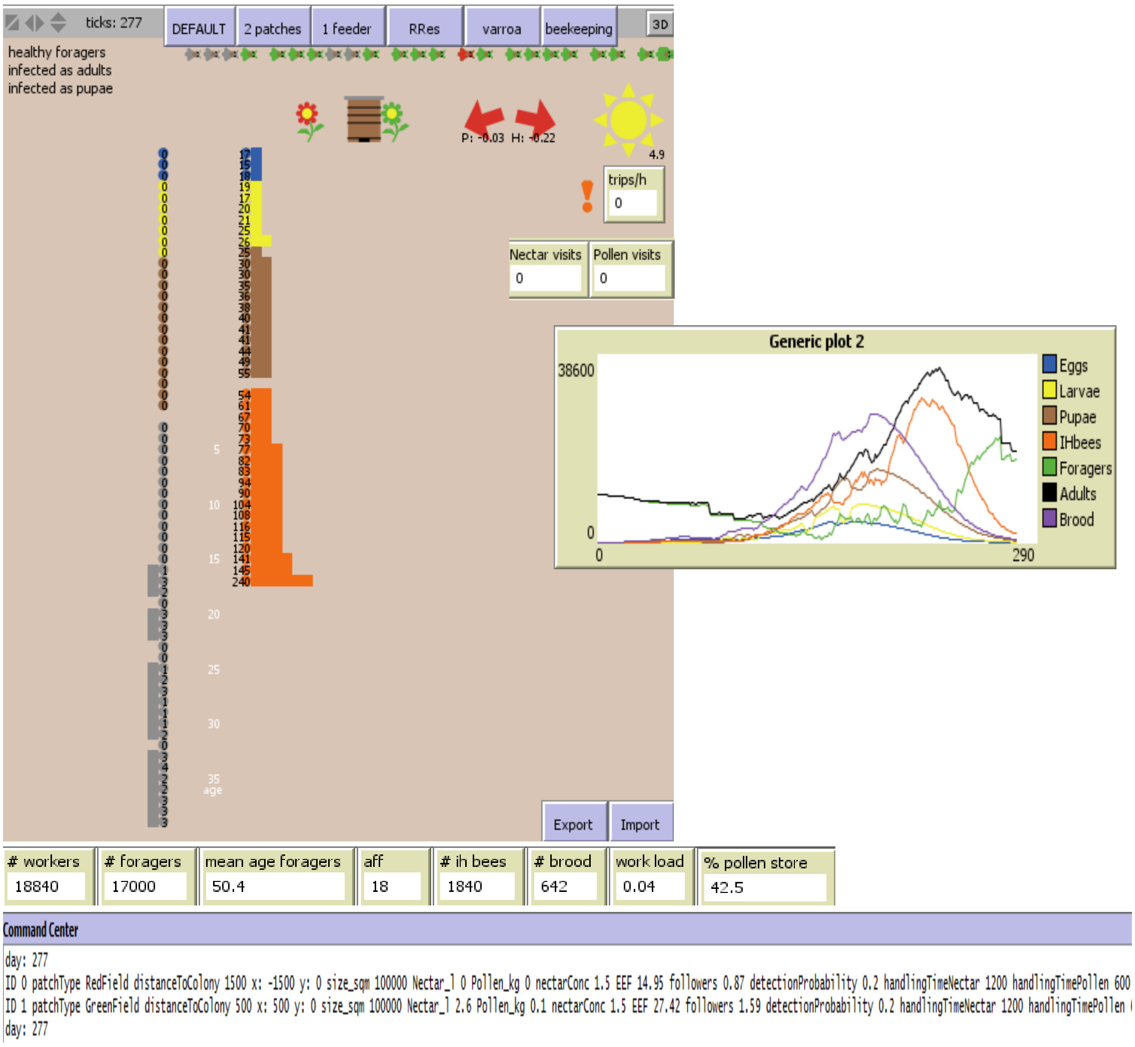

Figure 2.11: The colony composition, provided amount and energetic efficiency of nectar and pollen of both patches and the number of performed foraging trips on day 277.

16. In the end, click **1 Month** three times. Now, we attain tick '367' (day 2 of year 2).

The output field underneath the cohort visualization panel displays the size of the remaining (over-wintering bees) colony (*COLONY SIZE*) and the current store of honey (*HONEY STORE*) at 31st December (fig. 2.12).

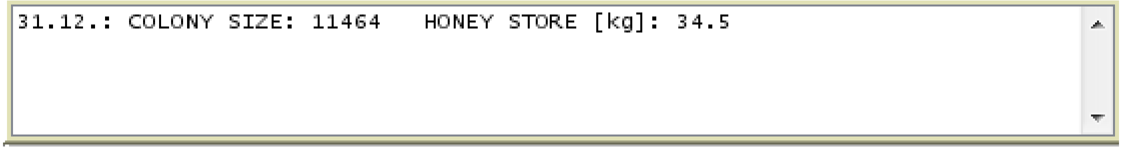

Figure 2.12: Colony size of overwintering bees and actual honey store at the 31st December.

17. Finally, let's take a look at the colony and foraging dynamics of several years. Please select the following eight output plots by selecting the corresponding choosers, in the given sequence, for the generic plots 1-8: *drones*, *colony structure workers*, *colony weight [kg]*, *active foragers [%]*, and *trips per hour sunshine*, *loads returning foragers [%]*, *nectar availability [l]* and *pollen availability [kg]*.
For example, click the chooser element of 'Generic Plot 1' and select the output *drones* as illustrated by figure 2.13.

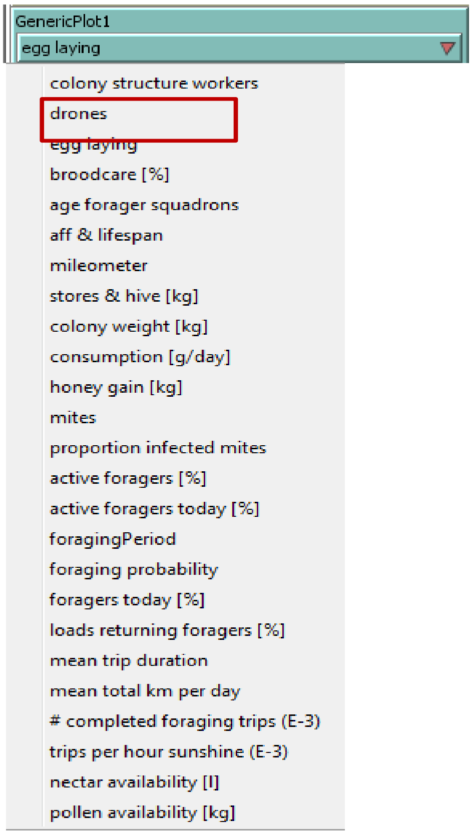

Figure 2.13: Selection of the output drones by the chooser element ‚Generic Plot'.

Press the **Setup** button and click the **1 Year** button three times in a row. Consider the following 'Generic plots' (fig. 2.14).

> **Note:** You can also write 1095 in the 'input field' **X_Days** and click **run X days**.

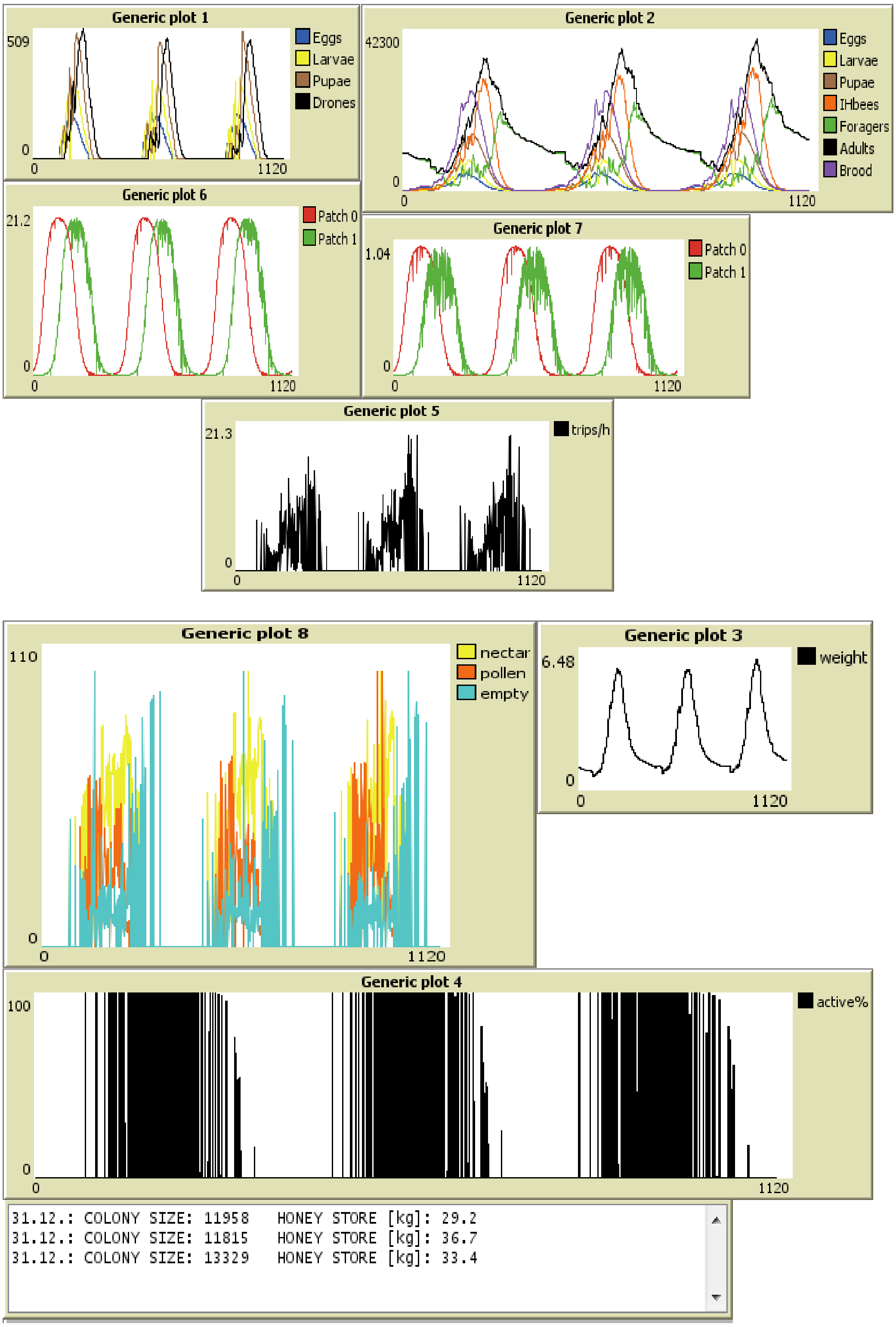

Figure 2.14: Colony and foraging dynamics of three consecutive years.

2.2 The *Varroa* Scenario

1. Type in the input field 'RAND_SEED' 8 and press the **varroa** button in order to initialize the pre-defined **varroa** scenario.

Table 2.2: Initial settings of the *Varroa*-specific parameters for the pre-defined **varroa** scenario are given.

Variable	varroa setting
N_INITIAL_MITES_HEALTHY	10
N_INITIAL_MITES_INFECTED	10
Virus	DWV
MiteReproductionModel	Martin
RAND_SEED	8
DISTANCE_R	1500
DISTANCE_G	500

2. Select the following plot options by the 'Generic plot' chooser: *egg-laying*, *colony structure workers*, *aff & lifespan*, *mites*, *age forager squadrons*, *drones*, *proportion infected mites*, *stores & hive*. Furthermore, choose the option 'All visits' of the chooser 'Foraging map'.

3. Click the **Setup** button.

4. When the pre-defined **varroa** scenario has been initialized, several 'output monitors' display specific variables of initial mite infestation (fig. 2.15).

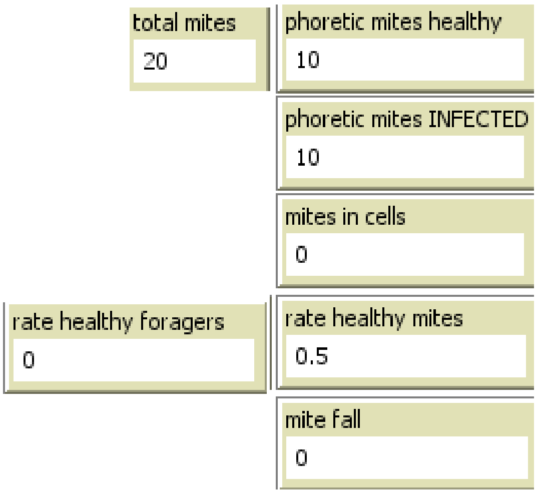

Figure 2.15: Initial variables of mite infestation and virus transmission as implemented in the pre-defined **varroa** scenario.

Thus, in total 20 phoretic mites (*total mites*) initially infest the honeybee colony, 50 percent of these are infected with the deformed wing virus (*rate healthy mites*).

The definition of the displayed mite variables can be called by the following steps. **Click the right mouse button** on any mite-specific 'output monitor' and then click on **Edit**. The dialog window of the monitor appears. Here, the monitors of five outputs, referred to by the input field "Display Name", are shown. Now, take a closer look at the reporters by which the values of mite-specific variables are daily computed:

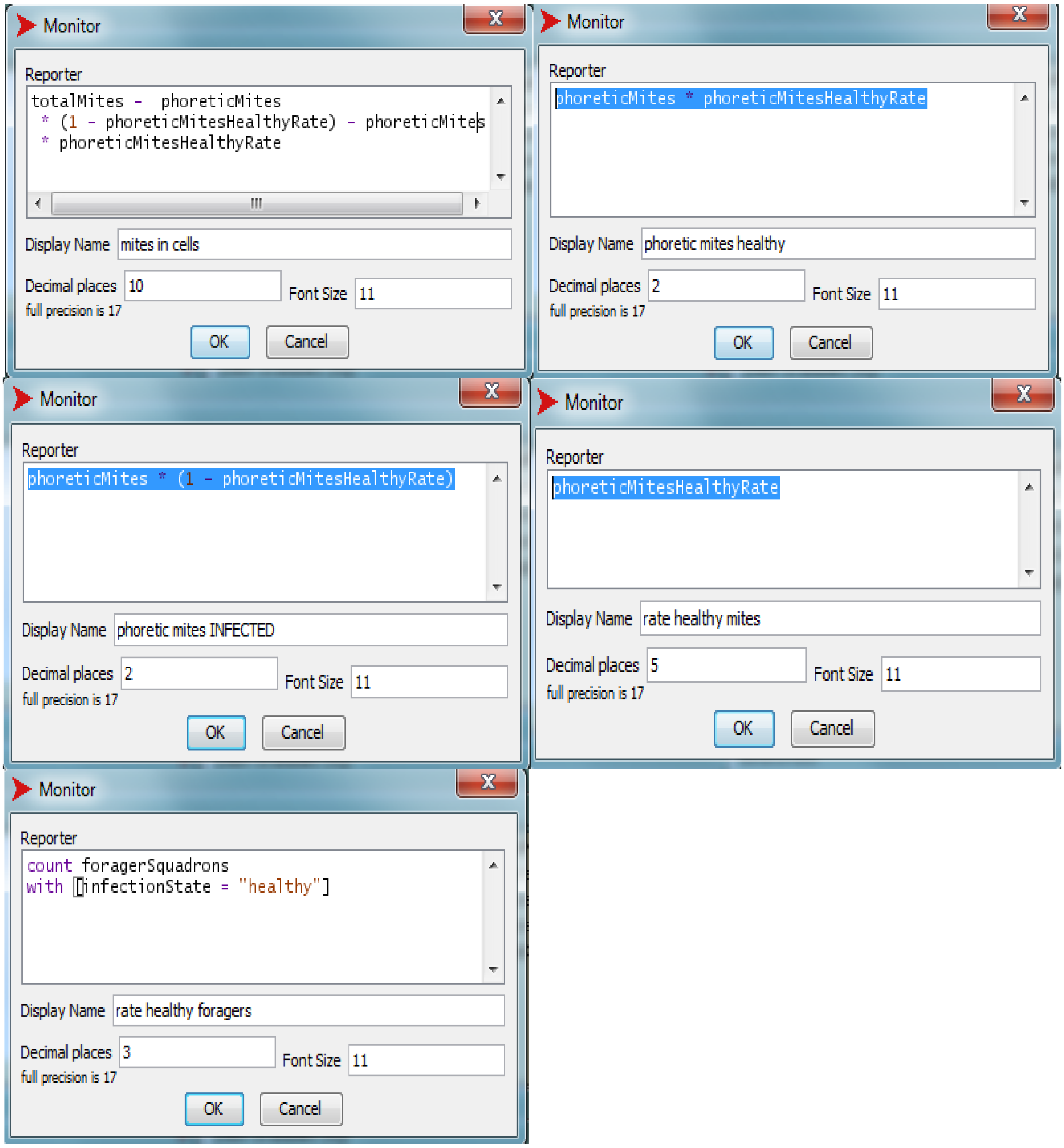

5. Next, click **Run**.

Now, this pre-defined simulation runs until the death of the colony illustrated by the '*skull*'. Subsequently, the day of colony death, colony sizes and honey stores of the several years are written to the 'output box'. Furthermore, for the day of colony's death the number of healthy and infected phoretic mites (*phoretic mites healthy*, *phoretic mites INFECTED*) is printed out (figure 2.16).

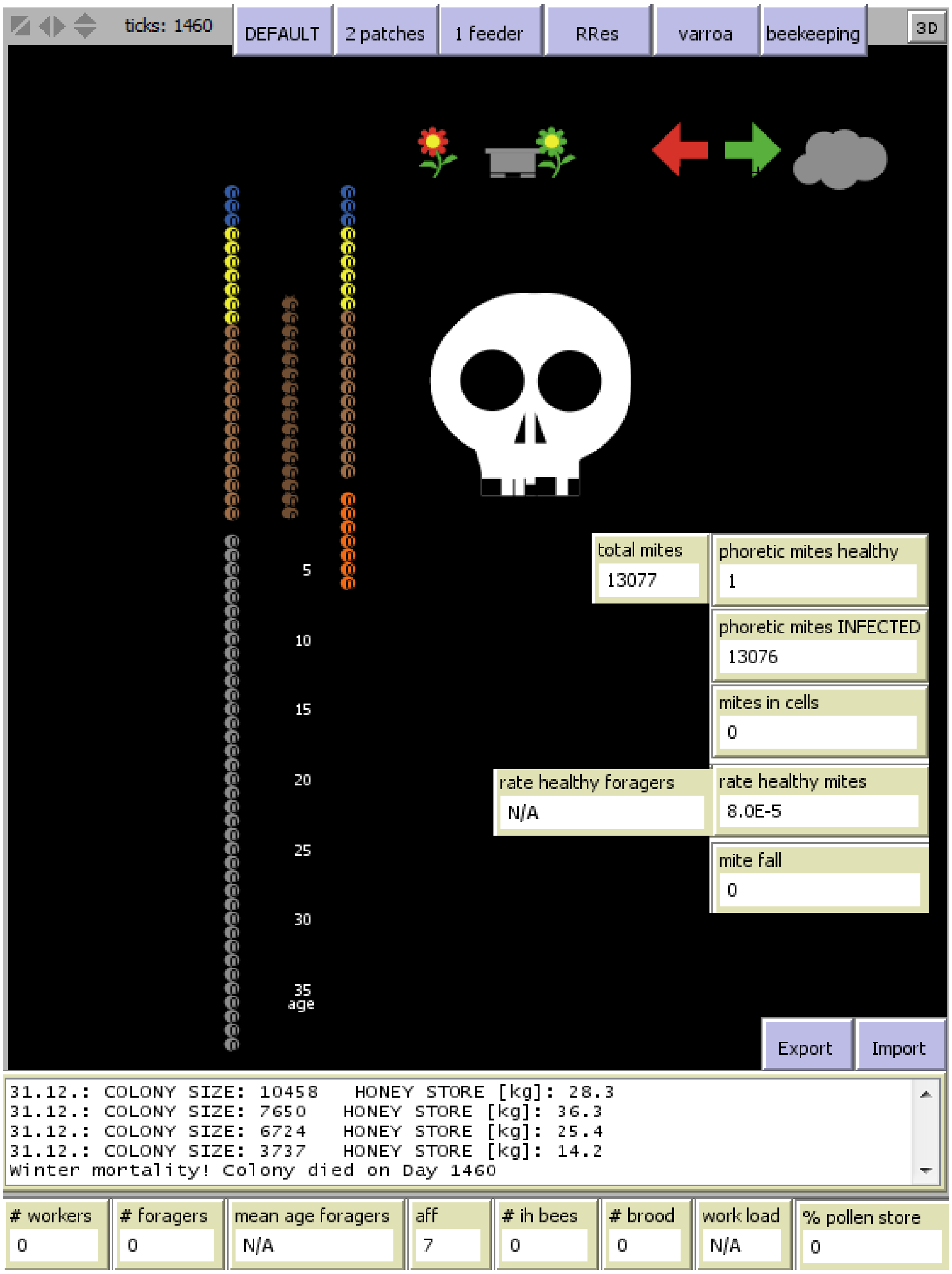

Figure 2.16: Death of the honeybee colony infested by DWV-transmitting *Varroa* mites on day 1460 of simulation run. The colony sizes and honey stores per year are printed out until the colony size falls below the critical threshold of 4000 surviving winter bees. The current number of healthy and infected phoretic mites on the day of colony's death is given.

Consider, over the simulation period of 1460 day until the colony died, the queen's egg-laying rate, the age-cohorts of workers and drones as well as the honey and pollen stores of the colony distinctly decreases as illustrated by 'Generic plot 1, 2, 6 and 8' of figure 2.17. Furthermore, the number of mites invading the honeybee colony and proportion of infected mites increases over time, but you can also see that the mite dynamics strongly depend on the honeybee colony dynamics ('Generic plot 4 & 7').

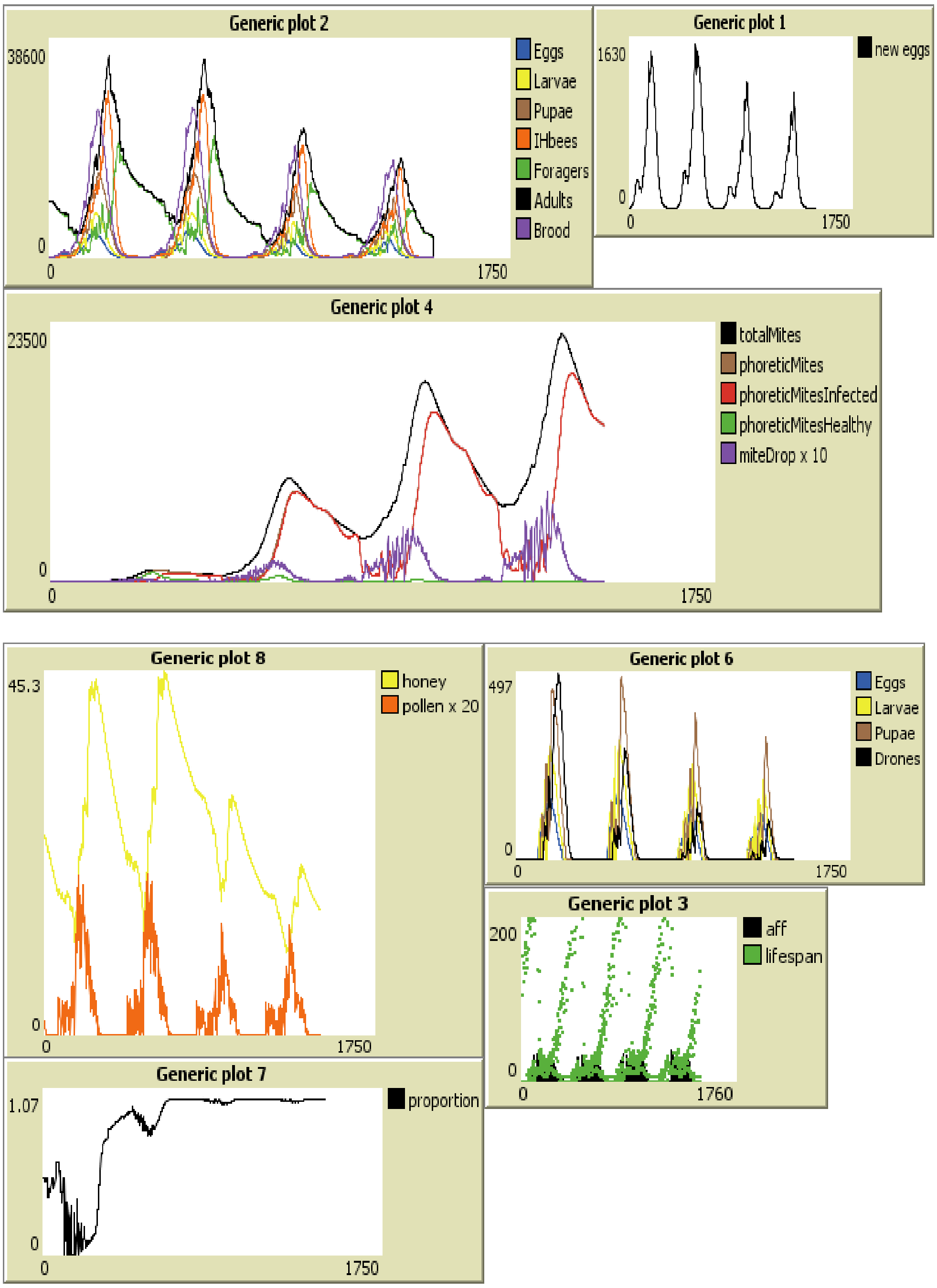

Figure 2.17: The temporal progress of egg-laying, structure of immature and adult stages of worker bees and drones, healthy and DWV-transmitting mites and mite drops, honey and pollen stores of the hive as well as the age of first foraging and lifespan of worker bees is presented over several years until the death of the colony.

Now, we conduct the pre-defined **varroa** scenario once more, but short step-by-step instructions offer the possibility to explore colony and mite dynamics as well as foraging activities at different time steps of simulation run.

6. Thus, enter ‘127’ into the input field ‘X_days’ and start the simulation run by clicking **run X days**.

Take a look at the age-cohorts of the colony, the actual number of healthy and DWV infected phoretic mites invading the colony and the current proportions of healthy and diseased foragers.

In total 26 *Varroa* mites actually infest the honeybee colony. However, 25 *Varroa* mites invade unsealed cells of the last larval stage on that day (output monitor: *mites in cells*). Moreover, one phoretic mites is healthy (*phoretic mites healthy*) and no mites are infected by the DWV-virus (*phoretic mites INFECTED*). Hence, the proportion of healthy mites is 1 (*rate healthy mites*). No mites fall from the comb on that day (*mite fall*). DWV-transferring *Varroa* mites invade the last and the pre-last larval stage of workers and drones. Few individuals of the in-hive bees were infected as pupae (red brown nuances of adult age-cohorts) and adults (dark orange nuances of adult age-cohorts) as illustrated by figure 2.18. Moreover, all actual foragers were neither infected by DWV as immature individuals nor adult bees as displayed by the *rate of healthy foragers* and ‘Generic Plot 5’.

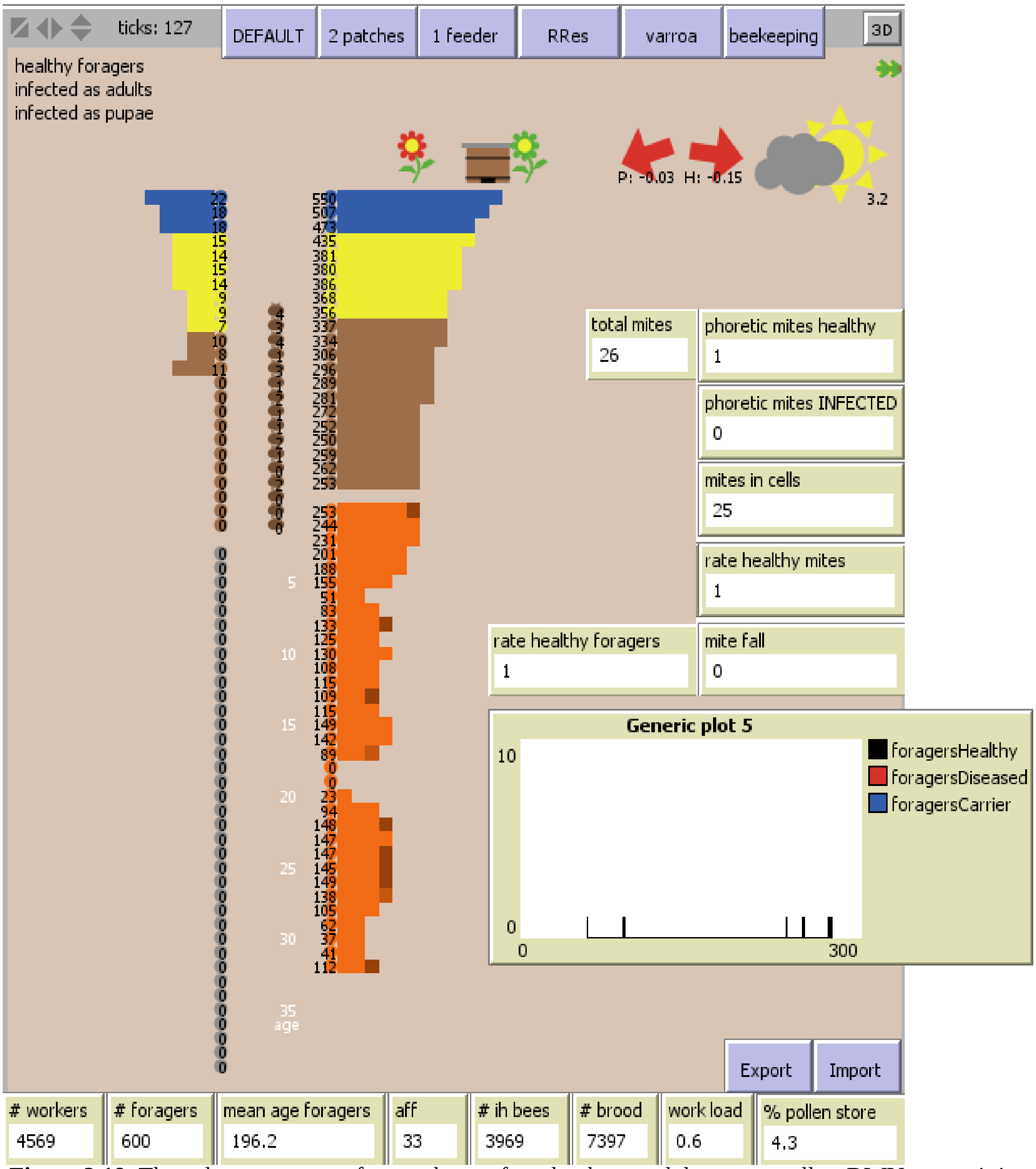

Figure 2.18: The colony structure of age-cohorts of worker bees and drones as well as DWV-transmitting *Varroa* invasion on day 127 of the pre-defined **varroa** scenario.

Now, we take a closer look at the mite infestation of drone and worker brood cells as well as adult in-hive workers.

7. Click the right mouse button on the pen-ultimate larval cohort of drone cells in the following way:

   Choose **drone larvae cohort 1209** and click **inspect drone larvae cohort 1209**. Repeat this application for the first and last pupal stage of workers, and the first age-cohort of in-hive worker bees.

The following windows appear:

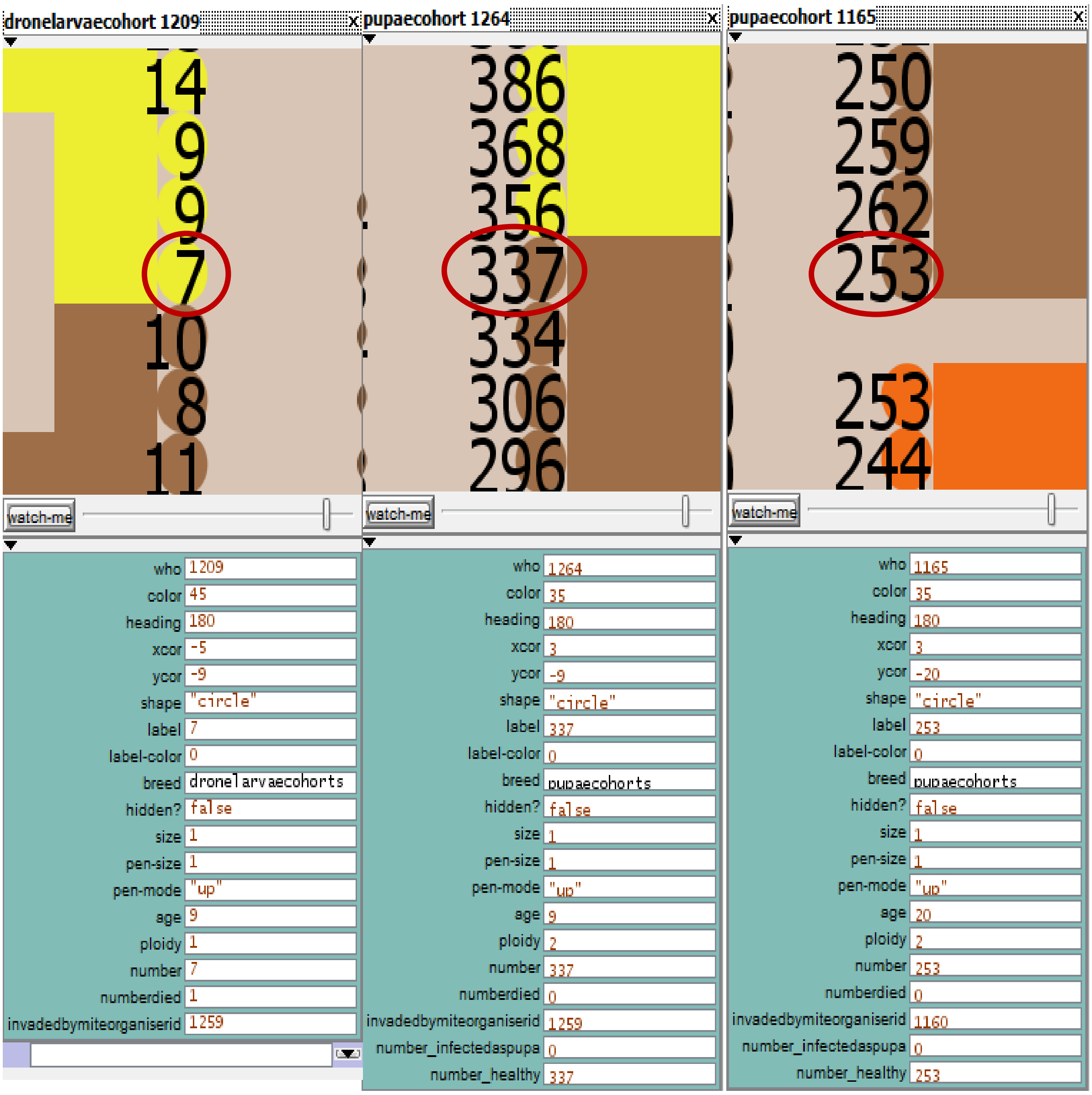

The windows of the larvae and pupae cohort display the ID (*who*), *age* and *ploidy* (1: drone, 2: worker) of the actual immature cohort. In addition, the ID of the mite organizer which invaded this actual brood cohort is displayed (*invaded by mite organizer id*). No mite infestation takes place if the mite organizer's ID is set to zero. So, the total larval or pupal cell number of the cohort (*number*) is composed of healthy (*number_healthy*) and dead (*number died*) brood cells. Moreover, drones and worker bees that hatched from immature stages may also be infested by DWV-transmitting mites as pupae (*number_ infected as pupae*) or attached by phoretic mites as adult bees (*number_infected as adult*). Here, the *age* of the actual in-hive cohort refers to the hatching date from the last immature stage to the adult stage.

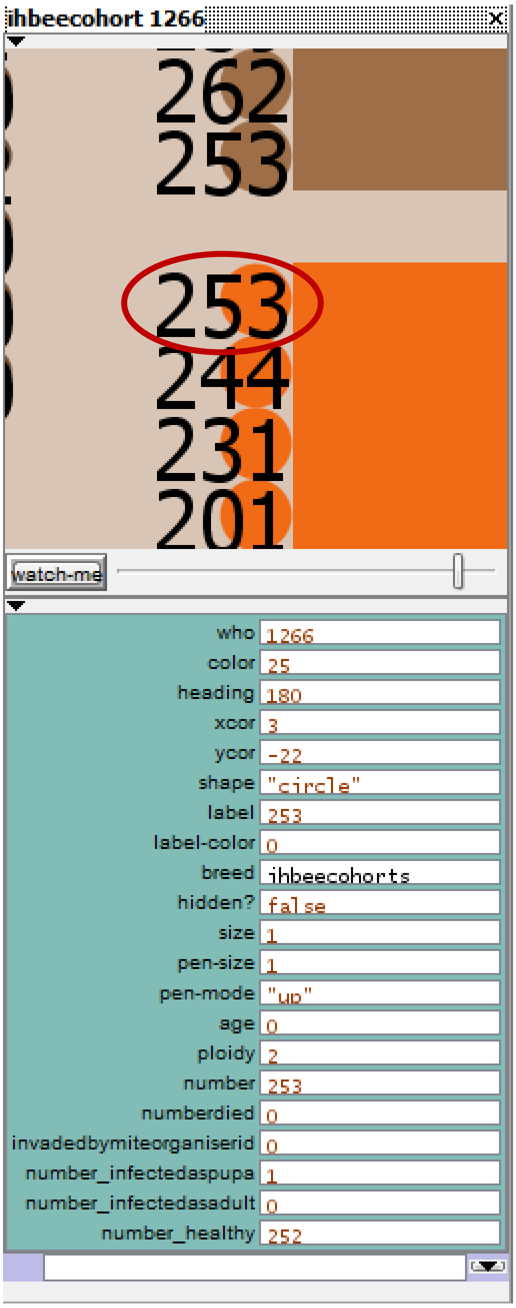

8. Click the right mouse button on the first *Varroa* sign in the following way in order to inspect the first mite organizer:

   Choose **miteorganiser1268** and click **inspect miteorganiser1268**.

   The following window appears:

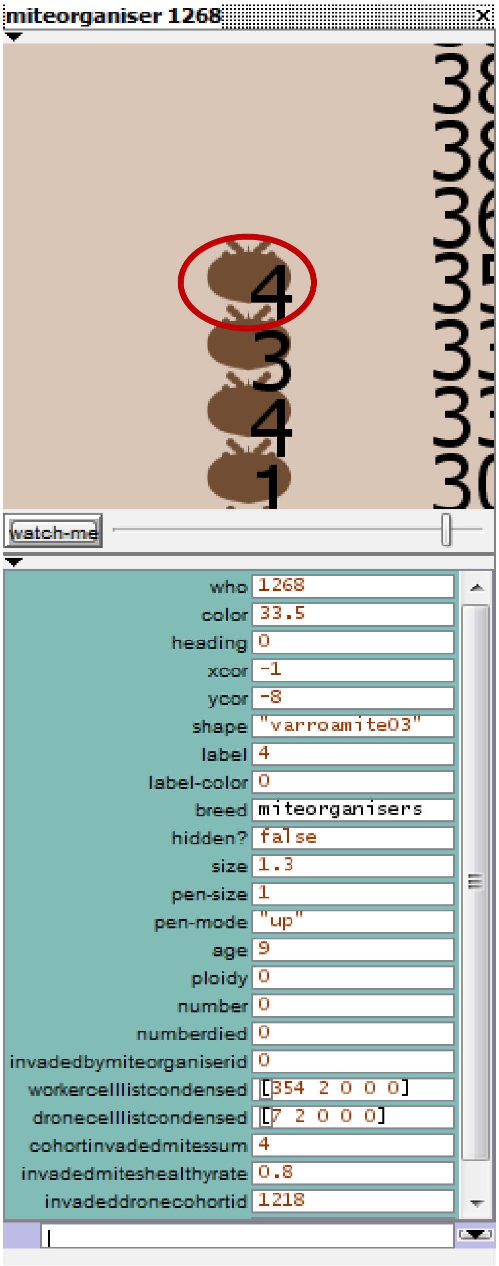

The window of the mite organizer shows how many brood cells of one worker or drone brood cohort are infested with zero, one, two or more mites whereas the maximum number of mites invading one brood cell depends on the chosen model of mite reproduction (e.g. ‘Martin’: four mites). However, the ID (*who*) of the mite organizer (1268) represents the entered worker larvae cohort including its *age* of infestation by the actual mite organizer. But the ID of the invaded drone brood cohort is separately shown (*invaded drone cohort id*). Moreover, the cell lists of the mite organizer extract the number of worker (*worker cell list condensed*) and drone cells (*drone cell list condensed*) invaded by zero, one, two or more mites respectively. Thus, the following worker cell list [364 3 0 0 0] means that 364 brood cells of this larval age-cohort are not infested by mites; three cells are invaded by a single mite, and no cells are invaded by two or more mites on this day. . In addition the rate of healthy invaded mites

(*invaded mites healthy rate*) and the total number of mites invading cells (*cohort invaded mites sum*), resulting from the product of number of infested cells and the number of mites within these cells, is given.

9. Enter '90' into the input field 'X_Days' and click **run X days**, again.
   The tick counter should now display day 217 (ticks) of model run.

Consider the structure of worker and drone age-cohorts and the infestation of adult drones and workers by DWV-transmitting mites as pupae or adults. Compared with day '127' of the simulation run the number of mites invading the honeybee colony increases with the season-dependent increase in the number of brood cells and in-hive bees (panel and 'Generic plot 4' of fig. 2.19). Now, in total 245 mites (*total mites*) actually infest the honeybee colony including 182 mites invading worker and drone brood cells (*mites in cells*) and 59 healthy phoretic mites attached on adult bees (*phoretic mites healthy*). On that day only 4 phoretic mites are infected (*phoretic mites INFECTED*) by the DW-virus, and thus the proportion of healthy mites actually amounts to 0.93651 (*rate healthy mites*). Few individual in-hive bees and adult drones were infected by DWV-transferring mites as pupae or adults (fig. 2.19). Thus, all foragers are healthy as illustrated by 'Generic plot 5' at the current day and they perform 30600 *Nectar visits* and 16200 *Pollen visits* ('ForagingMap'). Also, keep in mind how to explore the foraging activities using the following hints:

- Compare the amount of nectar and pollen rewards provided as well as the energetic efficiency of both patches!
  Press the advanced button: **show Patches**!
- How many nectar and pollen visits at the two patches are performed on that day?
  Press the advanced button: **visited patches** and **active patches!**
- Look at the activities of forager squadrons on that day!
  Press the advanced button: **activityList**!

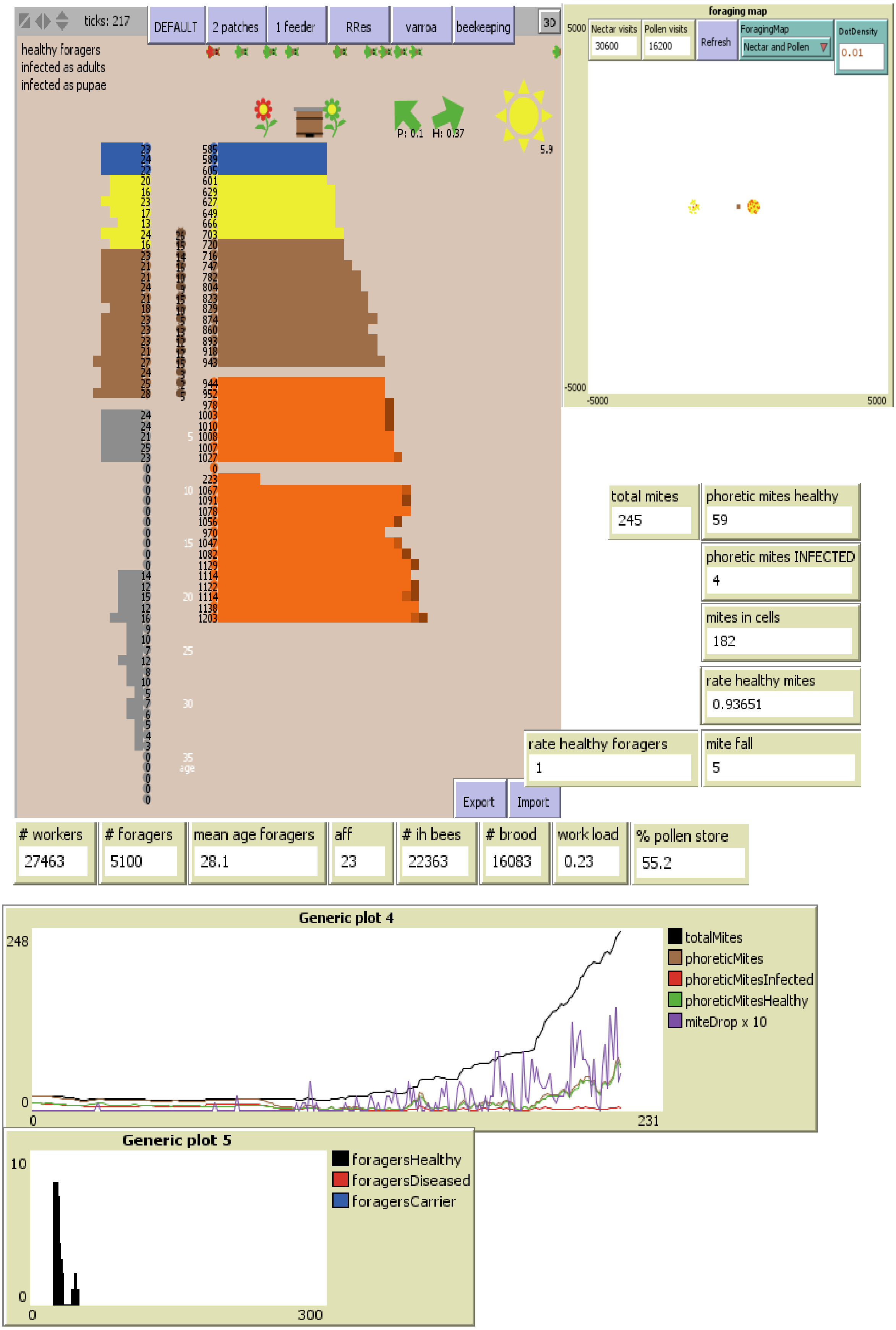

Figure 2.19: Structure of the age-cohorts of immature and adult worker bees and drones as well as *Varroa* invasion and in-hive stores are illustrated. The foraging map displays the nectar and pollen visits at the two available patches on day 217 of simulation. In addition, the current infection of forager squadrons is presented (day 217).

10. Next, enter ‘275’ into ‘X_Days’ and click **run X days**. Now, the simulation run attains tick 492 (day 127 of year 2). The colony size and honey store of the first year is printed underneath the panel of the age-cohorts.

On that day in total 526 *Varroa* mites invade the honeybee colony. 465 mites have entered brood cells just prior to capping and reproduce within these sealed drone and worker cells. The remaining mites live attached to adult bees (fig. 2.20). Moreover, the proportion of infected mites increases as illustrated by ‘Generic plot 7’, and no foragers were infected by DWV-transferring mites as adults (*rate healthy foragers*; ‘Generic plot 5’).

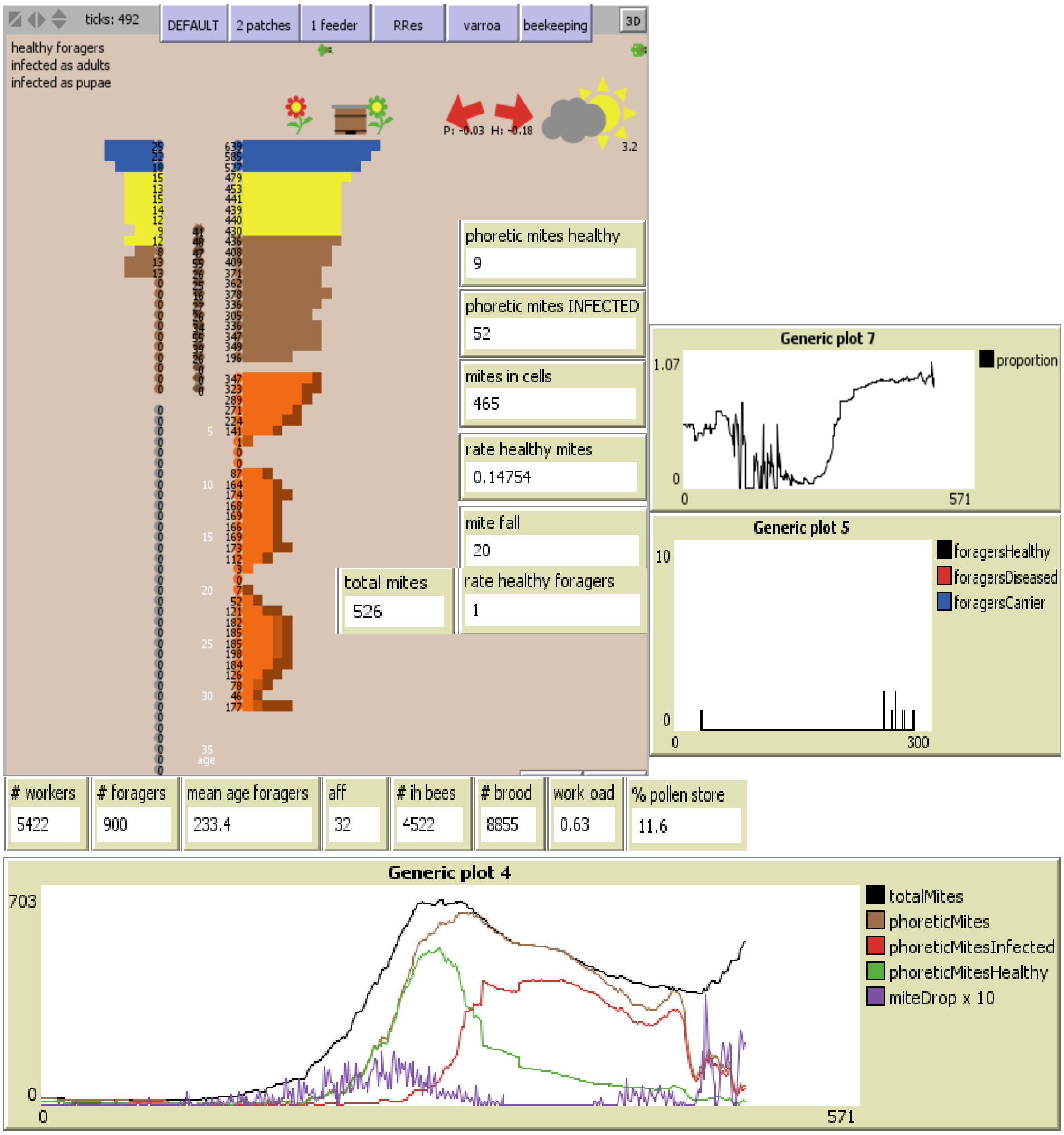

Figure 2.20: The colony’s structure, the infestation by *Varroa* mites, the proportion of infected mites and the infection of forager squadrons on the current day 492 are plotted.

11. In the input field 'X days' enter '90' and click **run X days**.

The tick counter should display 582.

The panel of age-cohorts shows that worker and drone brood cells are infested by mites. Moreover, adult drones and workers have been infected as pupae as well as adult bees. Thus, the number of mites invading the colony increases to 4004 on that day. The majority of foragers are healthy (*rate healthy foragers*: 0.855), but some foragers have been infected as pupae (diseased) or adults (DWV-carriers) as illustrated by 'Generic plot 5'.

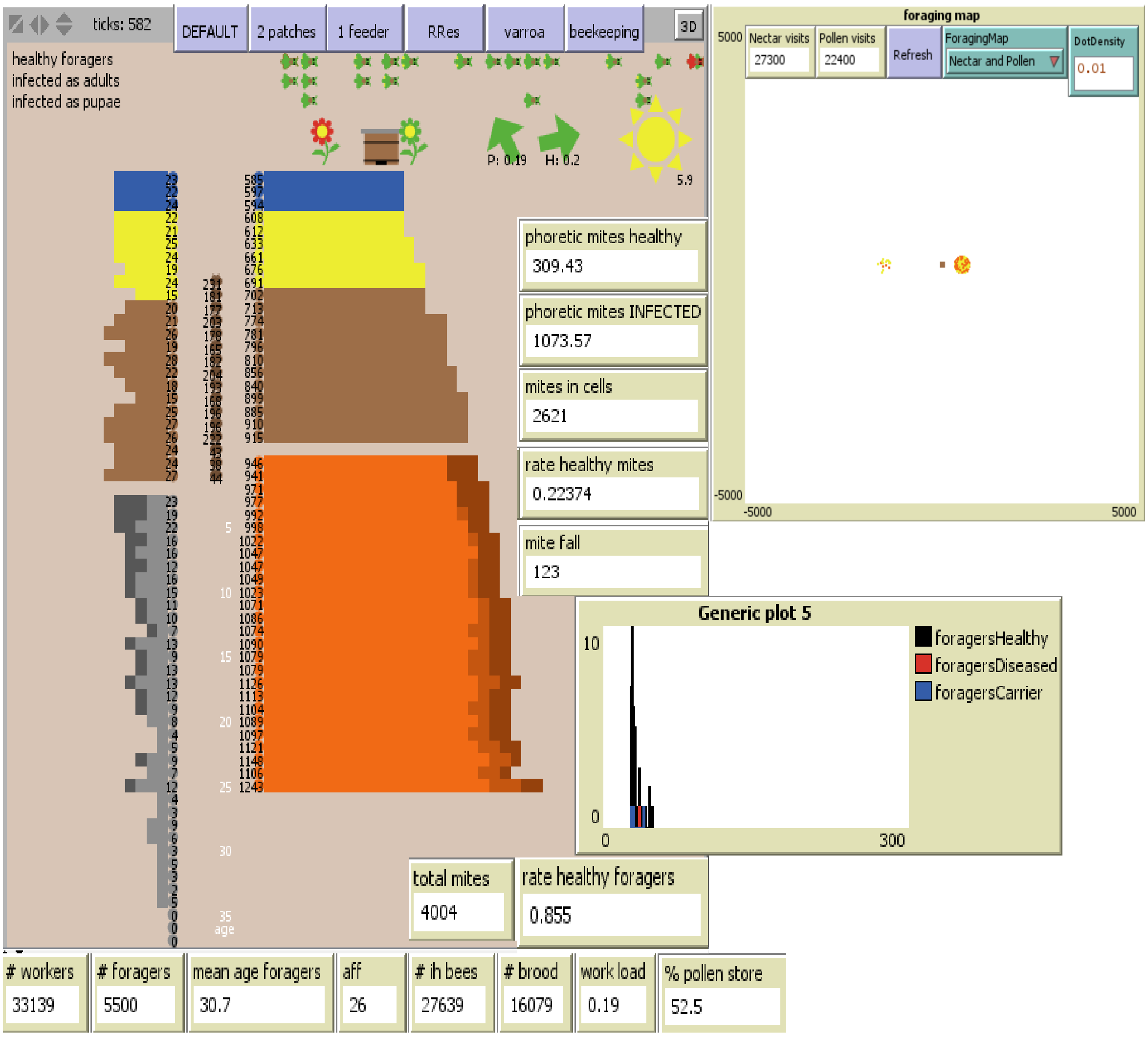

Figure 2.21: The panel illustrates the colony composition and the infestation of forager squadrons by DWV-infected *Varroa* mites on day 582.

12. Enter '275' into the box and click **run X days**, again. At this time of point, the tick counter displays that the current day of model run is the day 857 (day 127 of the third year).

    Inspect one mite organizer and some age-cohorts of larvae, pupae and in-hive workers by **clicking the right mouse button on** any age-cohort or mite organizer. Then select the marked mite organizer or age-cohort and click **inspect … cohort/ mite organizer … (ID)**.

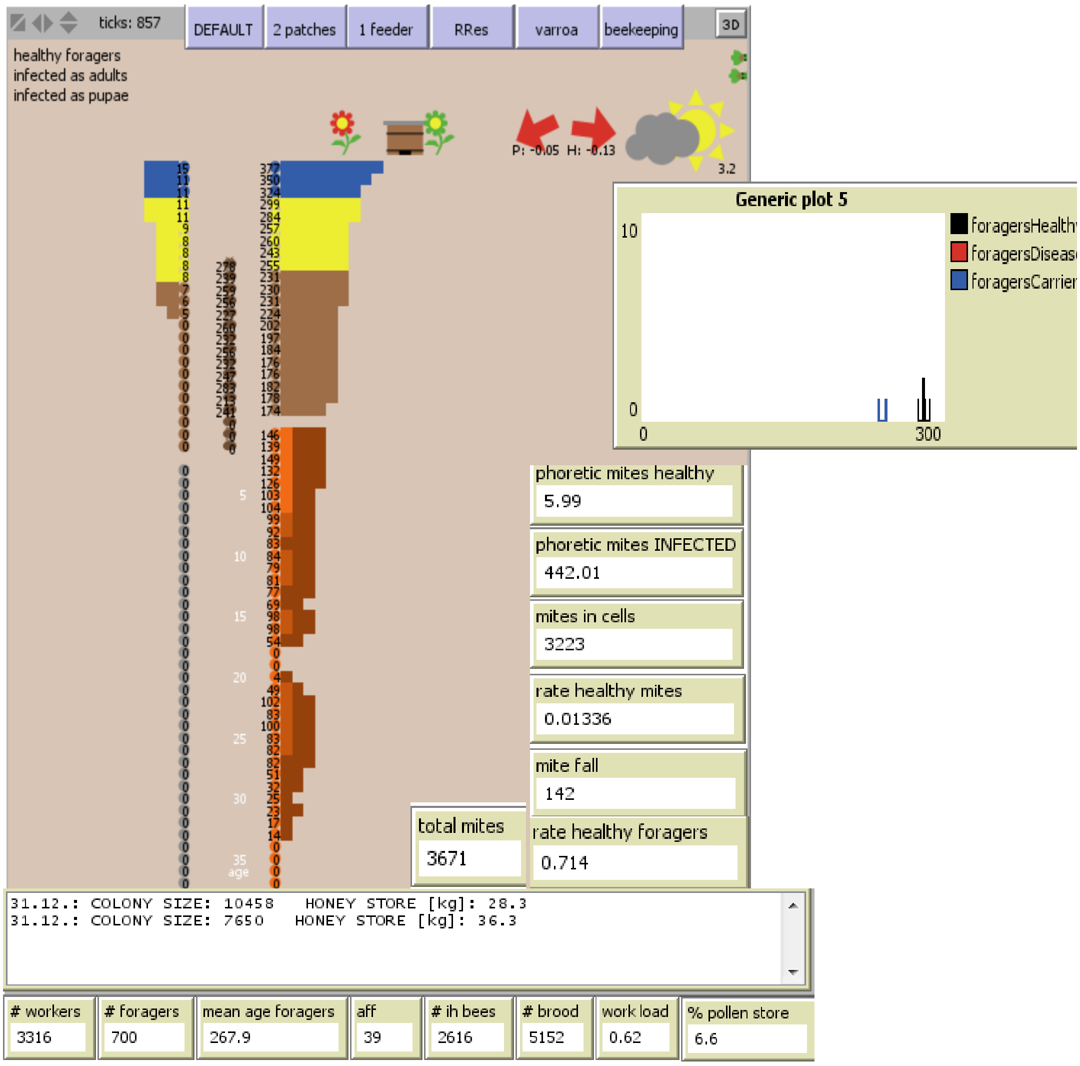

Figure 2.22: The panel illustrates the structure of the colony and the infection state of forager squadrons on day 857.

Now, the *rate* of *healthy mites* is very low (0.01336) and 142 mites actually fall from the combs (*mite fall*). Furthermore, the majority of in-hive workers have been infected as adults as illustrated by figure 2.22. For instance, the in-hive bee cohort 9528 consists of 92 in-hive workers including 69 workers infected as pupae, 7 workers attacked by mites as adults and only 16 healthy workers (fig. 2.23 left). Moreover, the window of the miteorganiser 9557

shows that 56 worker brood cells of the entered worker larva-cohort 9557 are not infested by any mites, but 65, 55, 19 and 7 brood cells have been infested by one, two, three and four *Varroa* mites (*worker cell list condensed*). No drone brood cell of the drone-cohort 9572 is infested by mites (*invaded drone cohort id, drone cell list condensed*). In total 260 mites actually invade brood cells of this age-cohort (*cohort invaded mites sum*) (fig. 2.23 right window).

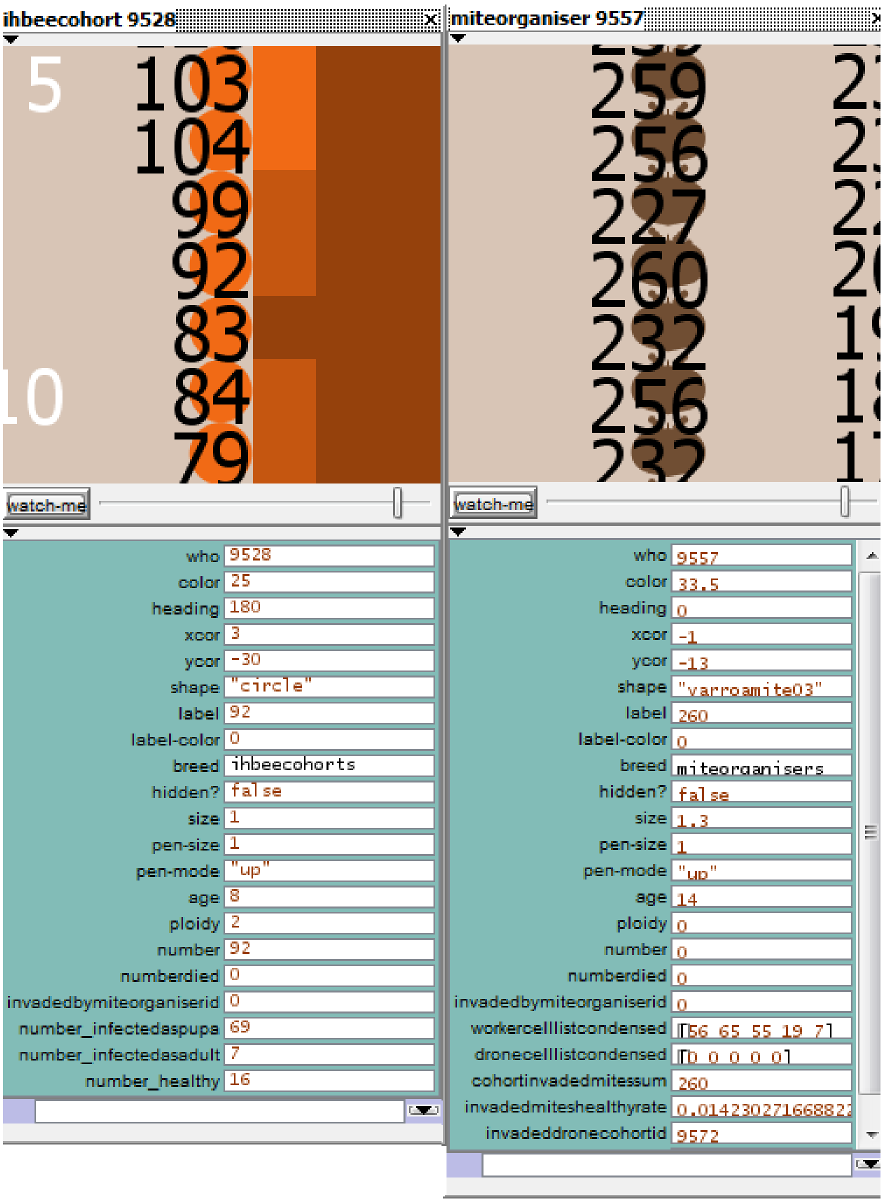

Figure 2.23: Display of the in-hive bee cohort 9528and the miteorganiser 9557.

13. Now enter ‘150’ into ‘X_days’ and click **run X days**.

The simulation run attains day 1007 and the majority of active foragers are infected by the deformed wing virus transmitted by infected mites. Thus, foragers of the colony are either carriers of the virus or already diseased (‘Generic plot 5’). Furthermore, the queens’s egg-laying rate, the amount of worker and drone brood and the total number of adult workers and drones decreases over the simulation period (‘Generic plot 1, 2 & 6’), whereas the mite infestation distinctly increases (‘Generic plot 4’). Also, take a look at the current number of healthy and infected phoretic mites, the number of mites infesting brood cells of the colony and the mite fall (fig. 2.24).

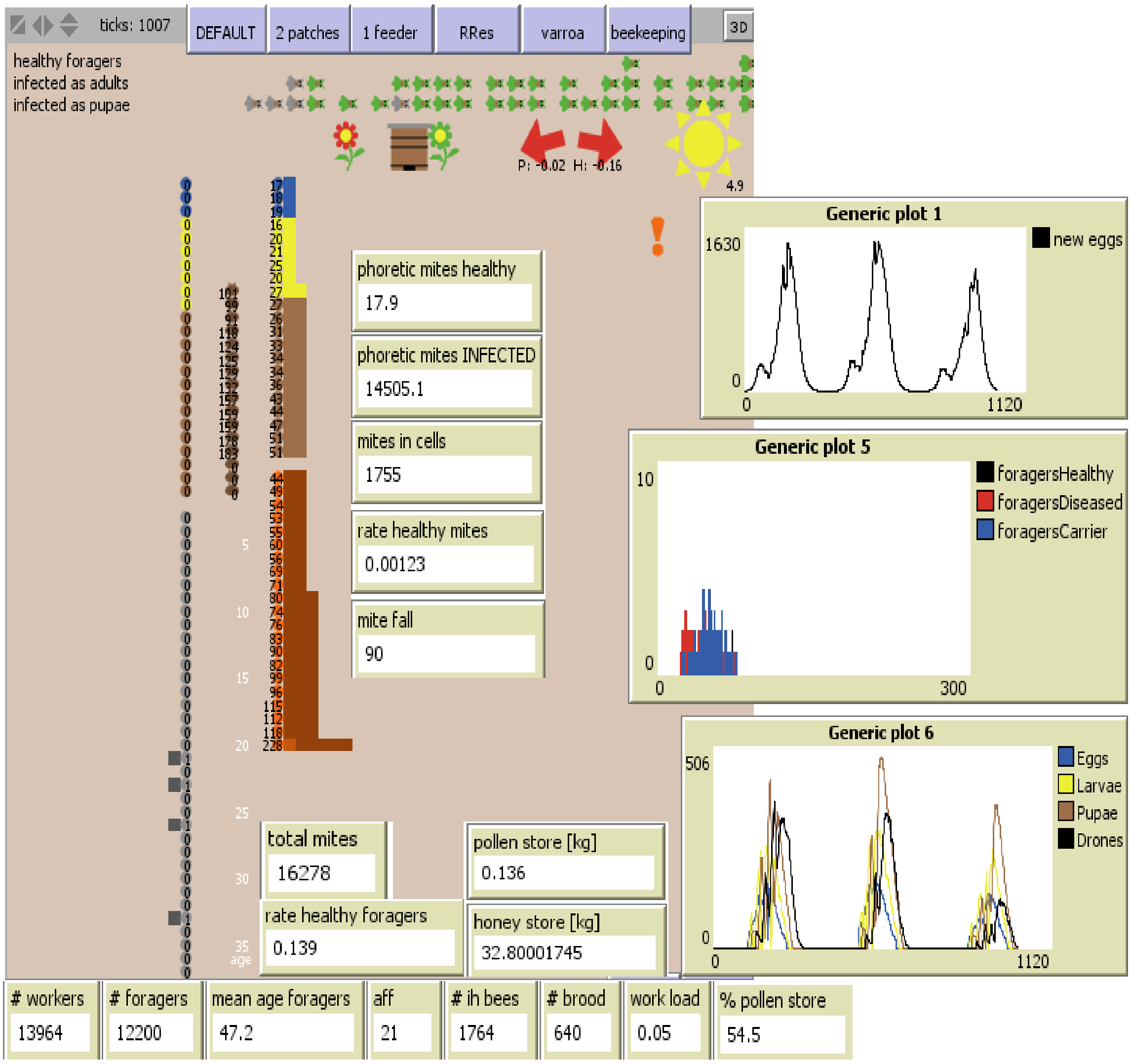

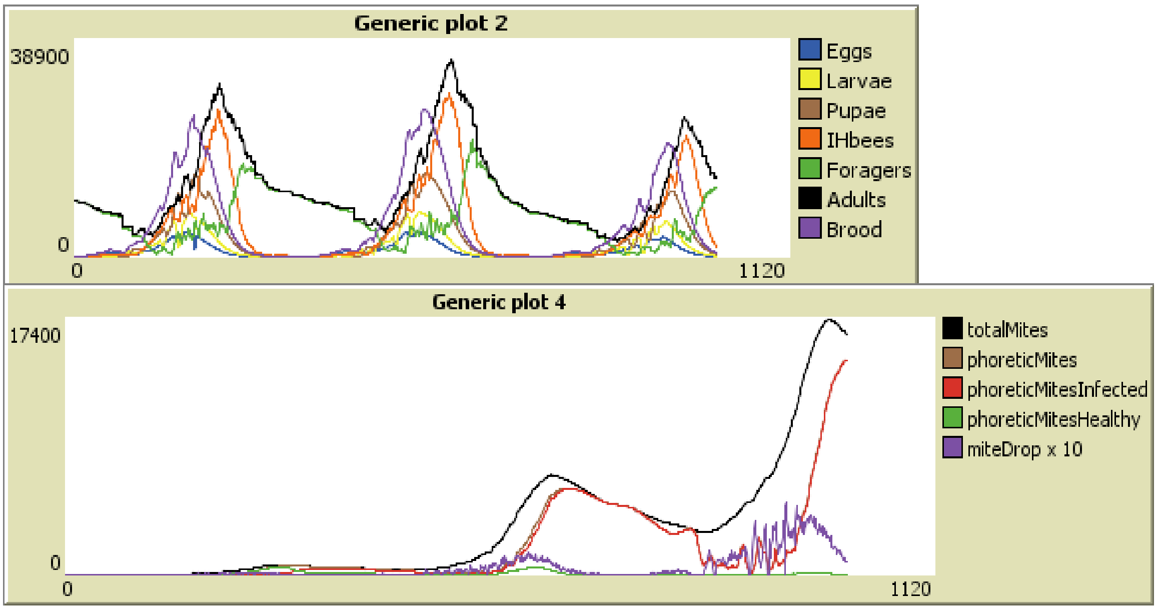

Figure 2.24: The panel illustrates the intra-colonial structure, the egg-laying rate, the infestation by infected *Varroa* mites and the infection of forager squadrons on day 1007.

14. Finally, click **Run** to run the simulation until the colony has died.

On day 1460 the colony size falls below the critical threshold of 4000 surviving winter bees. Look at the output of the colony sizes and honey stores of the several years until the death of the colony. Compare the development of the colony's structure, the mite dynamics and the infection of forager squadrons by DWV between the several years (see fig. 2.16 & 2.17).

15. Subsequently, perform a second *Varroa* simulation experiment by reducing the distances of the foraging sources to the hive. For this purpose, enter the following distances of patches to the hive: '1000' and '300' metres into the input boxes **DISTANCE_R** and **DISTANCE_G**.

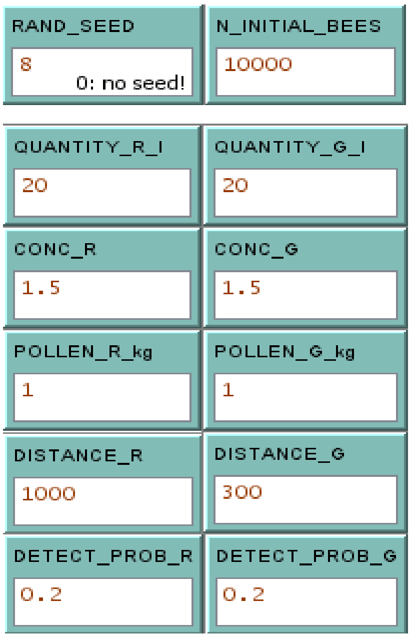

Next, click **Setup** in order to initialize these new parameter values. Now, click **Run** and wait until the colony died. Inspect the outputs of the model run. What has happened?

Note: You can also change the virus type of infected phoretic mites, the number of initial healthy and virus-infected mites as well as the mite reproduction model in order to examine the effect of different *Varroa* infestation conditions on colony survival. Furthermore, you may perform simulation experiments combining *Varroa* infestation and different foraging conditions.

3 BEEHAVE version control and documentation of simulation experiment

Last but not least we want to give some advices to model users that do not apply modeling approaches in their everyday work, how to ensure control of modified BEEHAVE versions and how to document your performed simulation experiments. Careful version control and documentation of changed parameters and procedures from the start may save you from unneeded trouble and wasted time.

3.1 BEEHAVE version control

Before you change anything in the actual model version make a new copy of the program and save it with a descriptive name regarding the following instructions:

1. Click the mouse button on **File**!

2. Choose **Save As…**!

3. Choose the directory and give a descriptive name and click **Save**!

   e.g. experiment name_changes (parameter, procedures)_version number_date

In addition, if you make changes in program code (e.g. edit input files, perform iterating operations for each item of a list within a procedure) or edit parameters (e.g. global, patch, local variables) or procedures use detailed comments in the procedures tab to document your modifications:

4. Click with the mouse button on **Code**!

5. Add your comments to modified parameters or procedures at the specific position in the BEEHAVE code by using the punctuation mark ‘**;**’ at the beginning!

Finally, create a table in order to document your changes made in each version of the BEEHAVE model. Probably, you like to use the exemplary box head presented below (table 3.1).

Table 3.1: Exemplary box head to document modified BEEHAVE versions. * In the NetLogo world consist of agent variables: global, turtle, patch, link and local variables. Changes of agents, procedures and parameter values include their modifications as well as deletion and editing.

version number	date	file directory	changed agents*	changed procedures	changed parameter values	planned experiments

3.2 Documentation of BEEHAVE simulation experiments

Much literature of documentation methods already exists (e.g. TRACE: Campolongo *et al*. 2007; Schmolke *et al*. 2010b, Railsback & Grimm 2011), thus we want to briefly clarify only the most important issues that should help to document your simulation experiments for investigations of e.g. factors affecting performance and foraging behaviour of honeybee colonies briefly and quickly. As or real experiments, the 'design of an experiment' have to be specified. Thus, we suggest that you give a complete and self-explanatory title and description of your simulation experiments performed with BEEHAVE by answering the following questions and briefly document your assumptions and chosen parameters:

- What is the purpose of the simulation experiment?
- Specify the settings of the simulation experiment:
  Which model versions, parameter values, initial values of state variables were used?
  Which assumptions were applied?
  Define the ranges and values over those specific parameters were varied!
  Which references (literature, field data, and expert opinion) for used parameters were turned into account?
- What currencies (output variables) did you measured?
- What is the time horizon for each simulation?
- How many repetitions were run for each simulation?
- What processes in the model are stochastic?

Probably, you like to create a short overview of your simulation experiments and may use the following suggested box head (table 3.2).

Table 3.2: Exemplary box head for brief documentation of simulation experiments performed with BEEHAVE.

**Scenario ID**	**description of experiment**	**time horizon**	**important currencies**	**repetitions of simulation run**	**parameter values**	**references**

References

Campolongo, F., Cariboni, A. & Satelli, A. (2007). An effective screening design for sensitivity analysis of large models. *Environmental Modeling & Software* **22**: 1509 – 1518.

Railsback, S.F. & Grimm, V. (2011). Agent-based and Individual-based Modeling: A Practical Introduction. Princeton University Press, Princeton, New Jersey.

Schmolke, A., Thorbek, P., DeAngelis, D.L. & Grimm, V. (2010b). Ecological modeling supporting environmental decision making: a strategy for the future. *Trends in Ecology and Evolution* **25**: 479-486.

List of Tables

Table 1.1	Functions of advanced initialization and output buttons	4
Table 1.2	Foraging activities of forager squadrons	6
Table 1.3	Definition and initial setting (default) of ‘Input field’ variables	9
Table 1.4	Definition and initial setting (default) of ‘Switch’ variables	12
Table 1.5	Definition of integrated ‘Chooser’ variables and their options	14
Table 1.6	Description of important plotted output variables	17
Table 1.7	Definitions of important output monitor variables	24
Table 2.1	Overview of initial settings of the **Default** scenario	29
Table 2.2	Initial settings of the *Varroa*-specific parameters of the **Varroa** scenario	48
Table 3.1	Exemplary box head to document modified BEEHAVE versions	67
Table 3.2	Exemplary box head for brief documentation of simulation experiments performed with BEEHAVE	67
