# Supplementary material for: BEEHAVE: a systems model of honeybee colony dynamics and foraging to explore multifactorial causes of colony failure
Source: J Appl Ecol. 2014 Mar 4;51(2):470–82. doi: 10.1111/1365-2664.12222 (PMC4283046; doi:10.1111/1365-2664.12222)
Supplement: Supplementary file 4 — Appendix S4. (JPEbecherSA4_Sensitivity.pdf): Complete sensitivity analyses. [file JPE-51-470-s004.pdf]

Appendix S4: SENSITIVITY ANALYSES

Sensitivity analyses were done for the default setting and when varroa was added (i.e. *N_Initial_Mites_Healthy*: 10, *N_Initial_Mites_Infected*: 10). Each single parameter was multiplied by a factor ranging from 0.1 to 4 (x-axis). Mean colony size after 3 years was used as output (y-axis) (N = 10). Default values (i.e. factor = 1) are shown as a white circles. The effect of *Squadron_Size*, which defines the number of foragers represented by a forager super-individual, was varied from 1 to 1000.

Without varroa | With varroa

(i) "ABANDON_POLLEN_PATCH_PROB_PER_S" (default probability = 0.00002 per second)

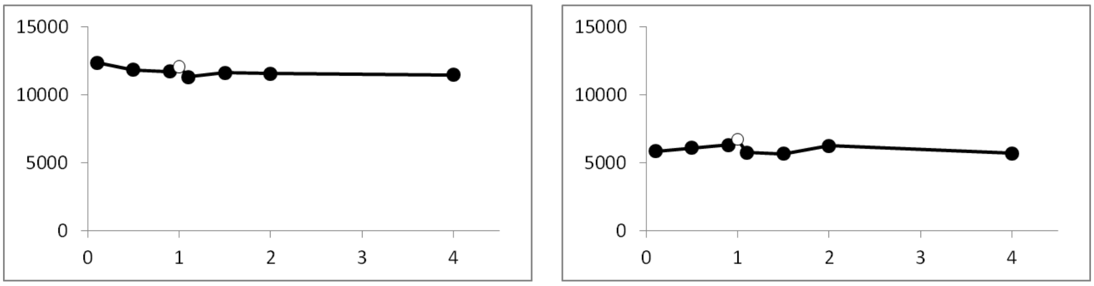

(ii) "AFF_BASE" (default = 21 days)

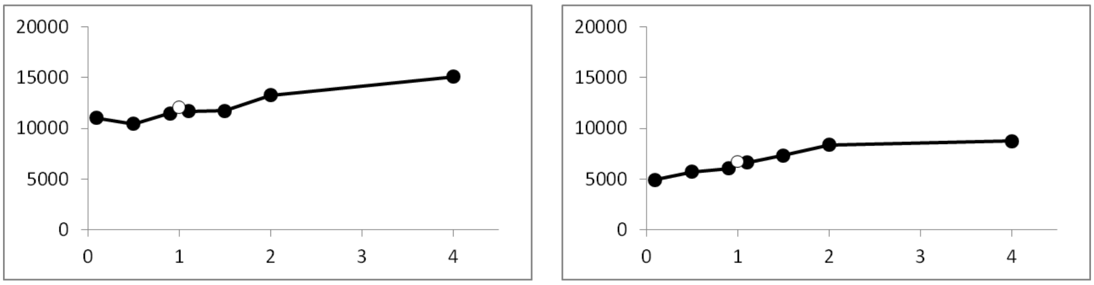

(iii) "CONC_G" (default concentration = 1.5 mol/l)

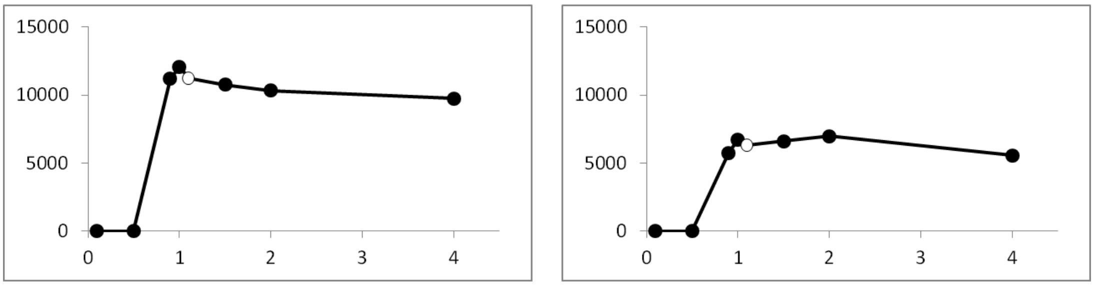

(iv) "CONC_R" (default concentration = 1.5 mol/l)

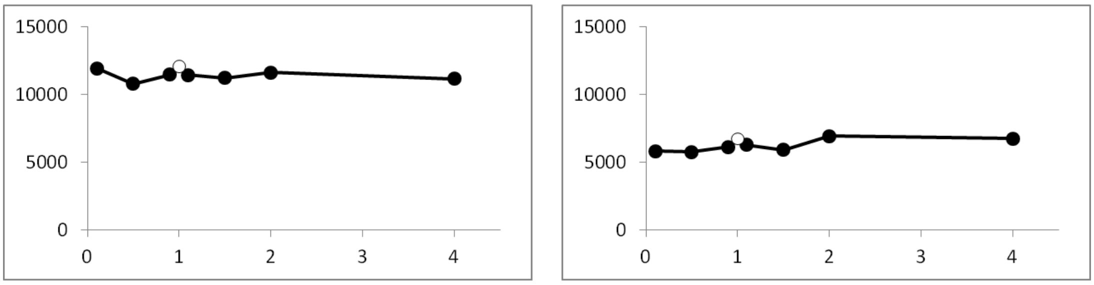

Appendix S4

Without varroa — With varroa

(v) "CRITICAL_COLONY_SIZE_WINTER" (default size = 4000 bees)

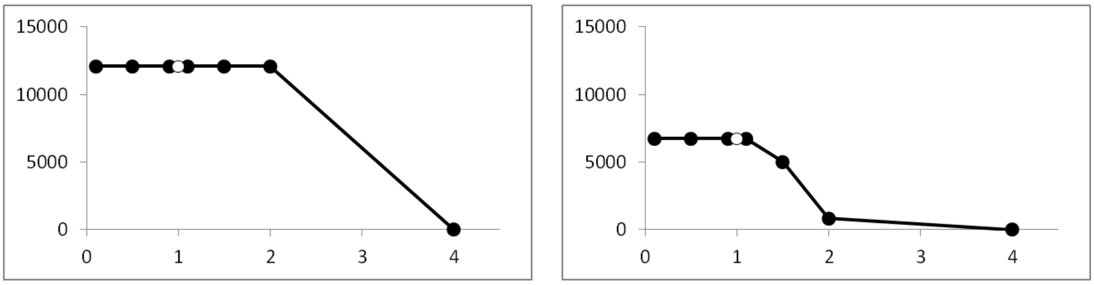

(vi) "CROPVOLUME" (default volume = 50 µl)

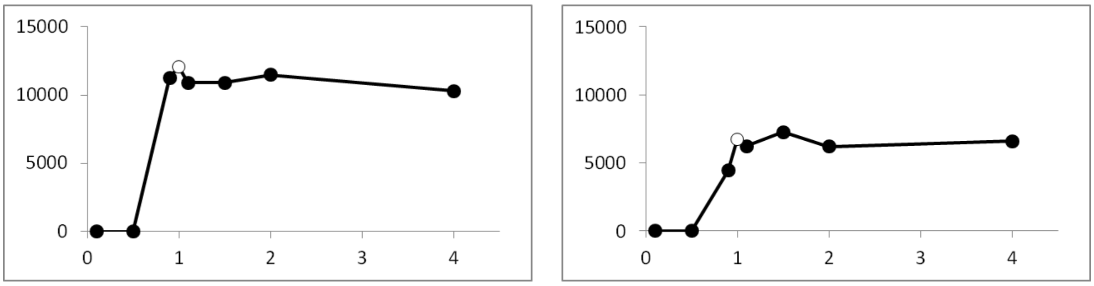

(vii) "DANCE_SLOPE" (default = 1.16 dance circuits)

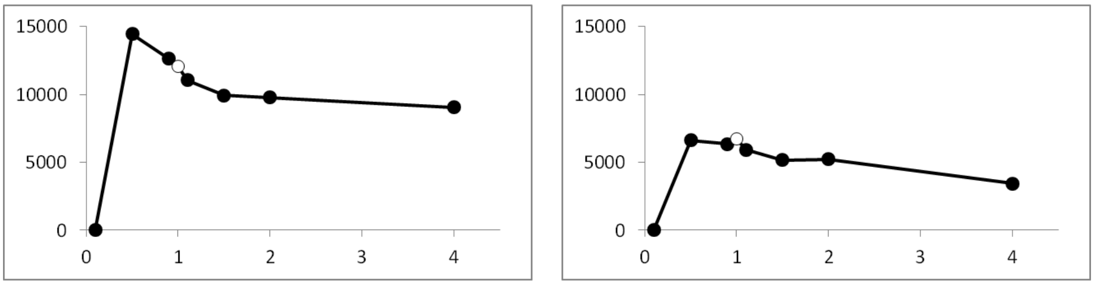

(viii) "DETECT_PROB_G" (default probability = 0.2)

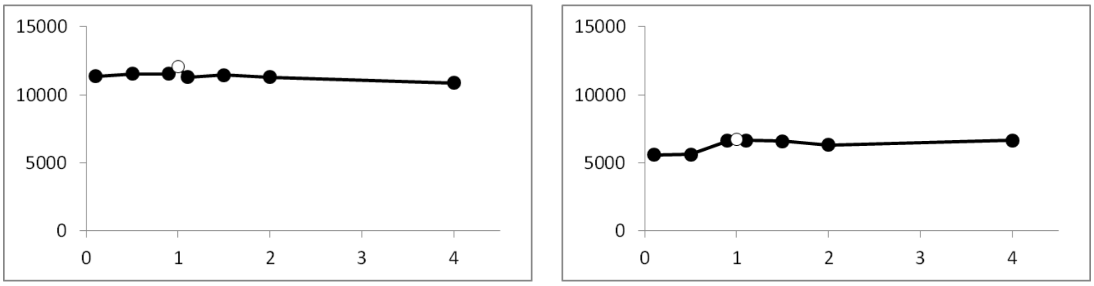

Appendix S4

Without varroa | With varroa

(ix) "DETECT_PROB_R" (default probability = 0.2)

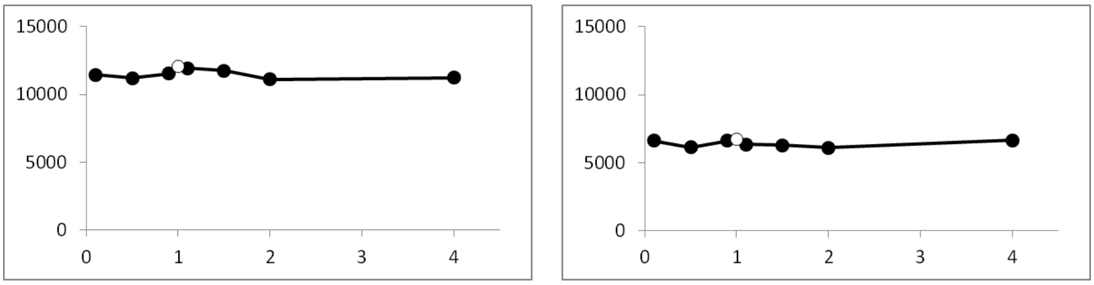

(x) "DISTANCE_G" (default distance = 500 m)

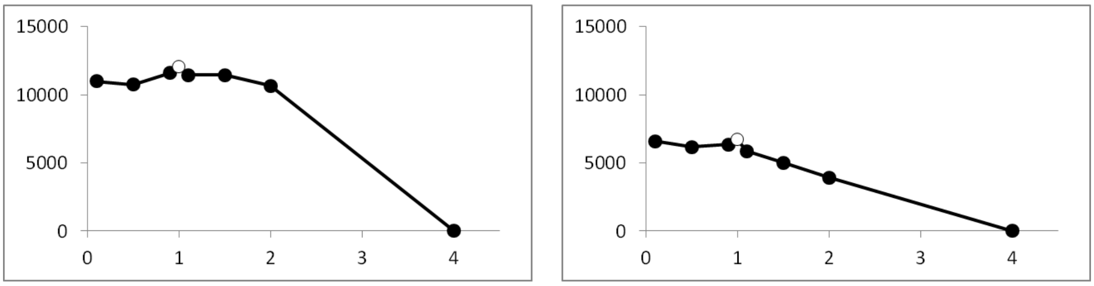

(xi) "DISTANCE_R" (default distance = 1500 m)

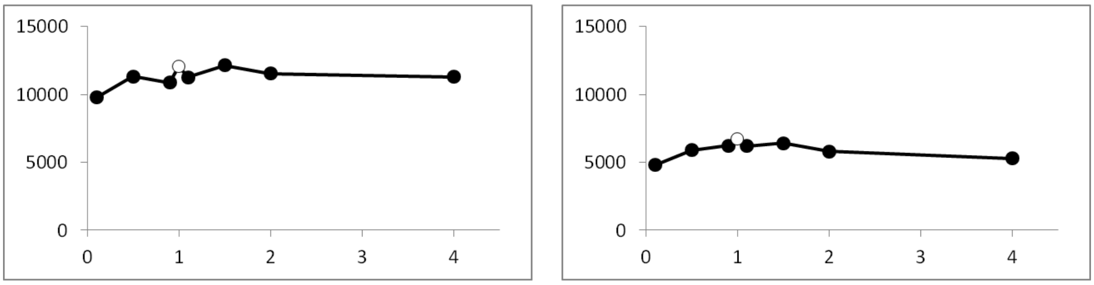

(xii) "DRONE_EGGLAYING_START" (default = day 115, i.e. 25th April)

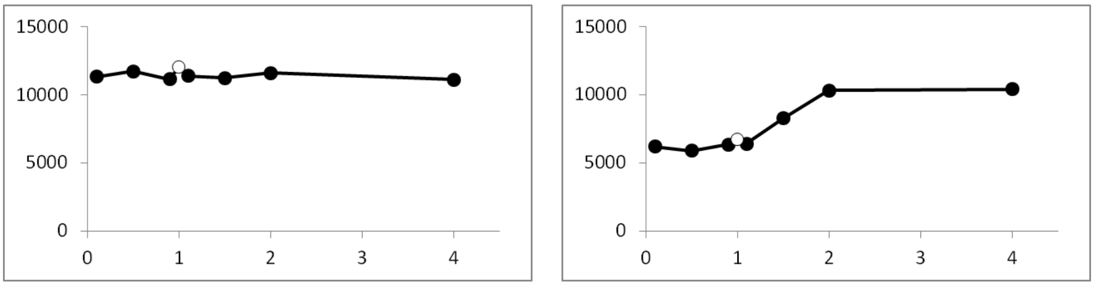

Appendix S4

Without varroa | With varroa

(xiii) "DRONE_EGGLAYING_STOP" (default = day 240, i.e. 28th August)

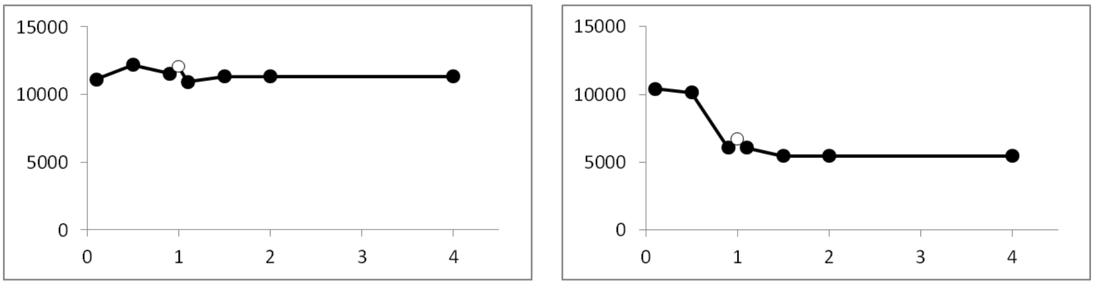

(xiv) "DRONE_EGGS_PROPORTION" (default proportion = 0.04)

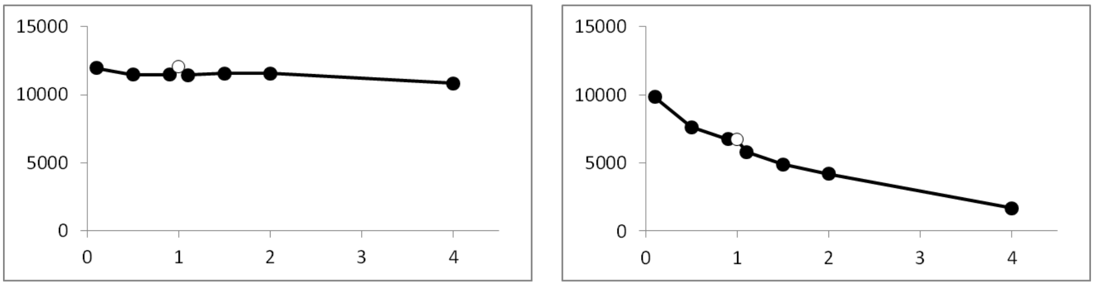

(xv) "DRONE_LIFESPAN" (default = 37 days)

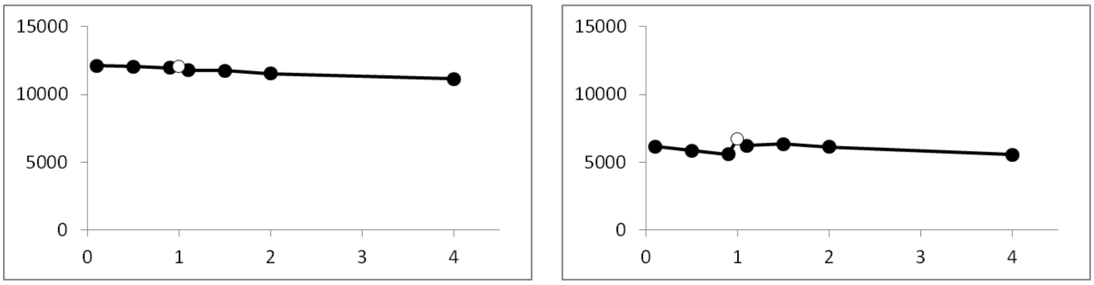

(xvi) "FIND_DANCED_PATCH_PROB" (default probability = 0.5)

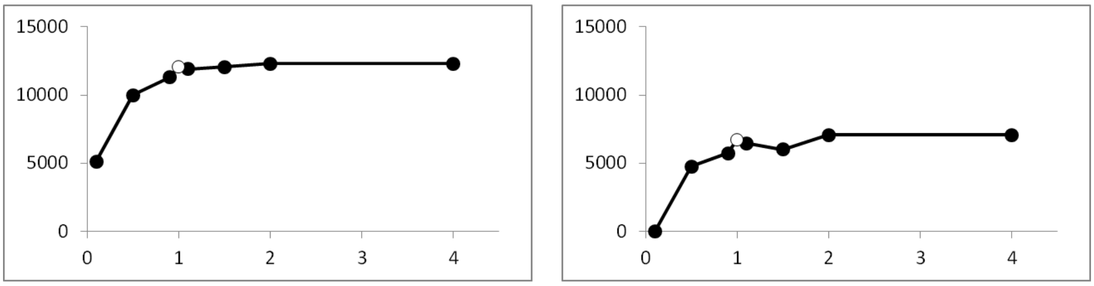

Appendix S4

	Without varroa	With varroa

(xvii) "FLIGHT_VELOCITY" (default velocity = 6.5 m/s)

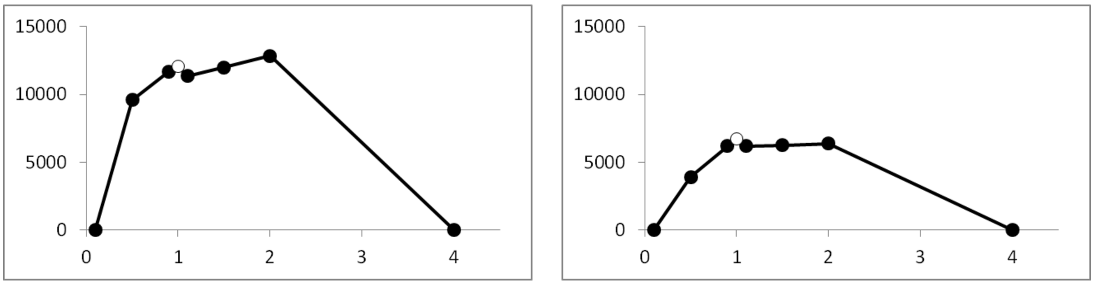

(xviii) "FLIGHTCOSTS_PER_m" (default = 0.000006 KJ/m)

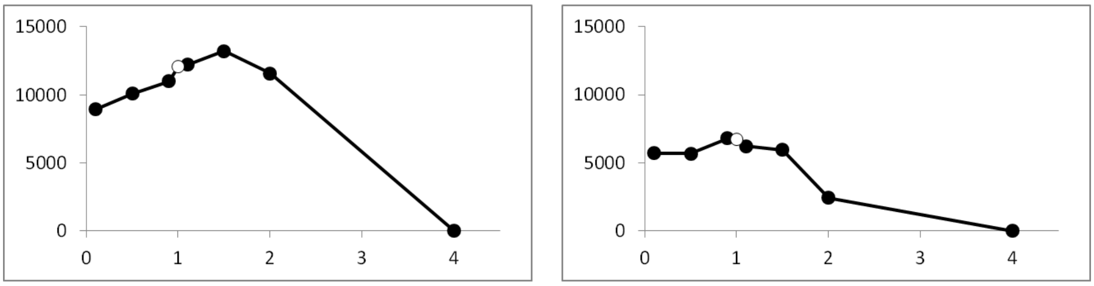

(xix) "FORAGER_NURSING_CONTRIBUTION" (default contribution = 0.2, relative to in-hive bees)

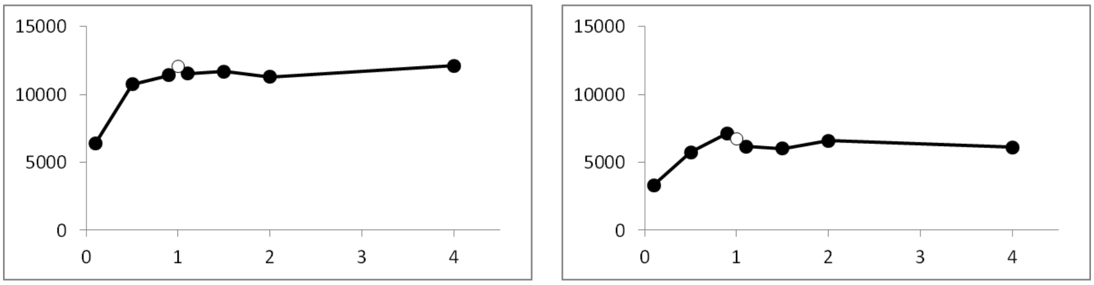

(xx) "FORAGING_STOP_PROB" (default probability = 0.3)

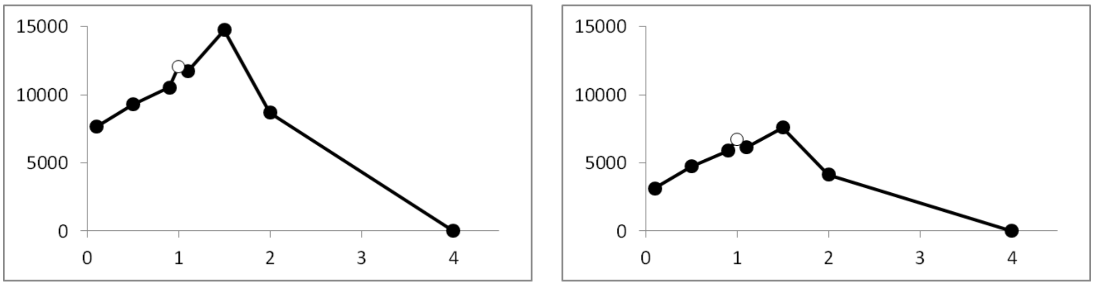

Appendix S4

Without varroa | With varroa

(xxi) "LIFESPAN" (default = 290 days)

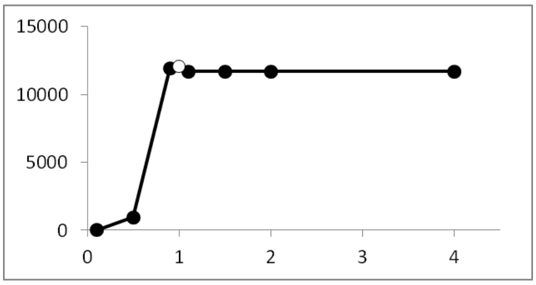

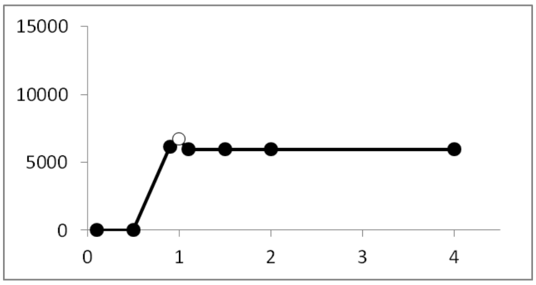

(xxii) "MAX_BROOD_NURSE_RATIO" (default probability = 0.3 per emergence)

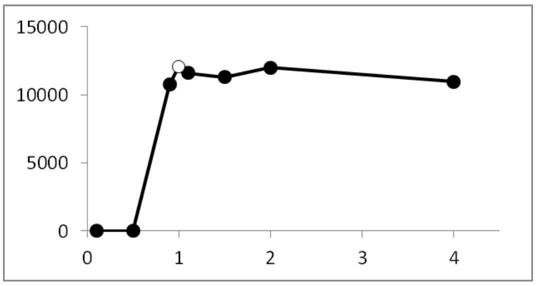

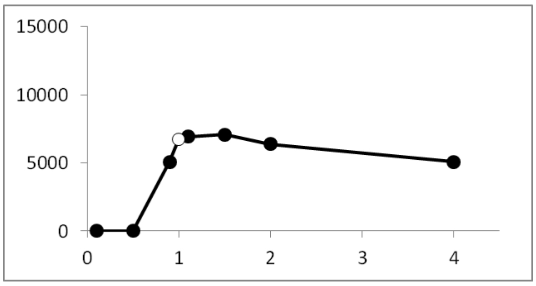

(xxiii) "MAX_DANCE_CIRCUITS" (default = 117 circuits)

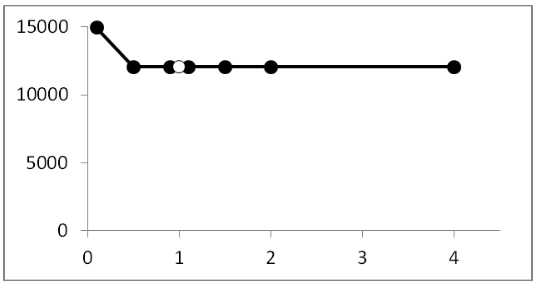

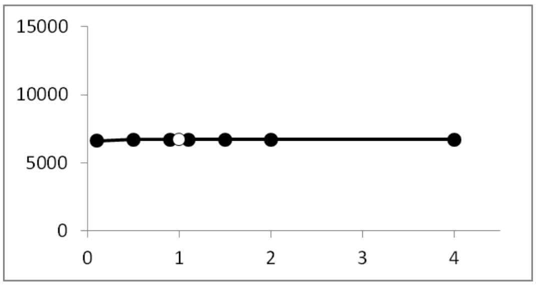

(xxiv) "MAX_EGG_LAYING" (default = 1600 eggs per day)

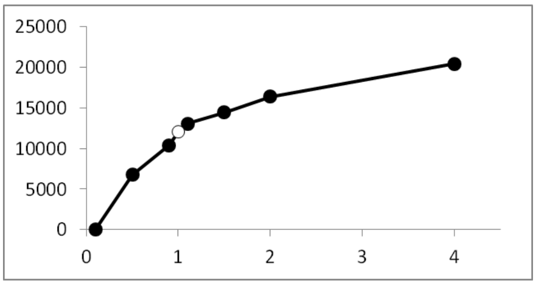

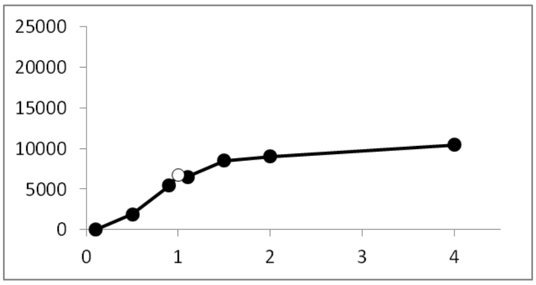

Appendix S4

Without varroa | With varroa

(xxv) "MAX_HONEY_STORE_kg" (default weight = 50 kg)

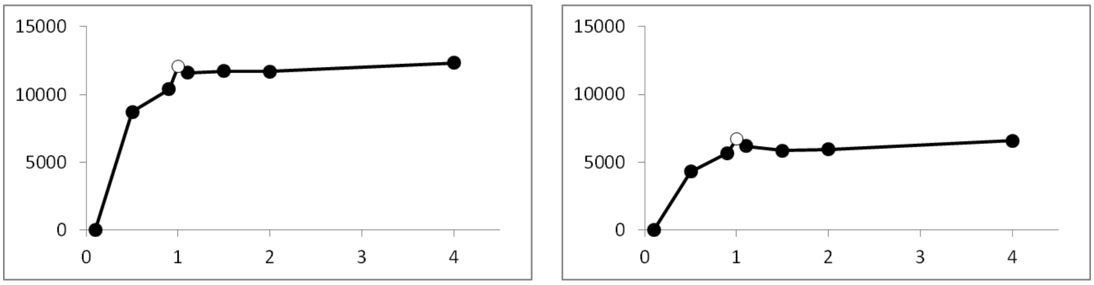

(xxvi) "MAX_PROPORTION_POLLEN_FORAGERS" (default proportion = 0.8)

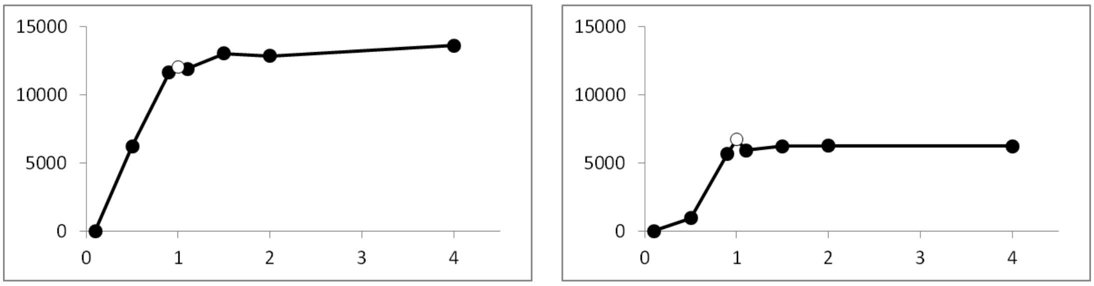

(xxvii) "MAX_TOTAL_KM" (default distance = 800 km)

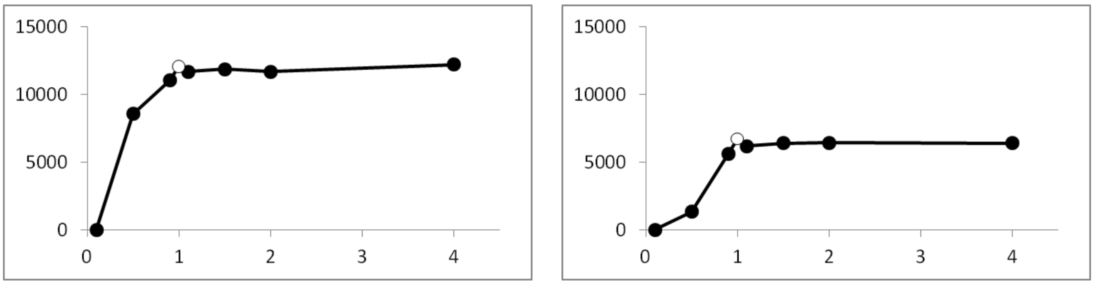

(xxviii) "MIN_IDEAL_POLLEN_STORE" (default weight = 250 g)

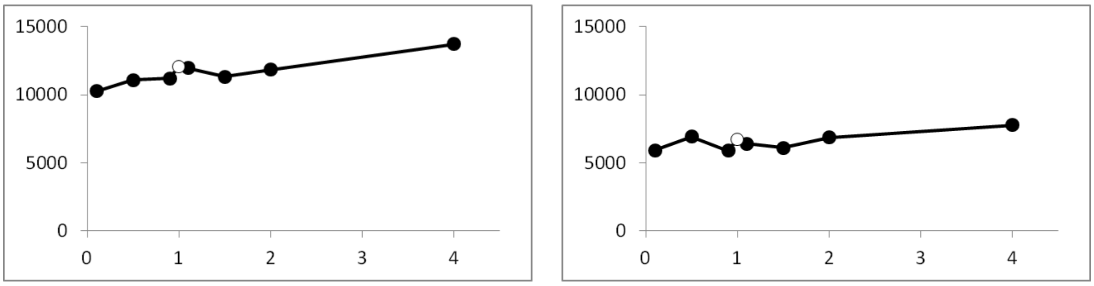

Appendix S4

Without varroa With varroa

(xxix) "MITE_FALL_DRONECELL" (default proportion = 0.2 per emergence)

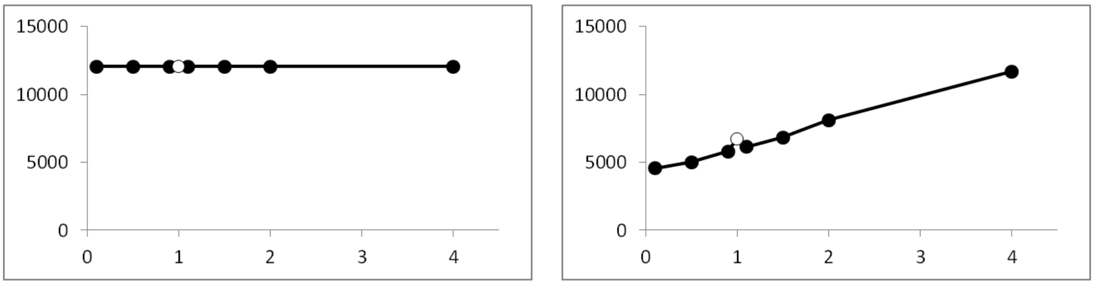

(xxx) "MITE_FALL_WORKERCELL" (default proportion = 0.3 per emergence)

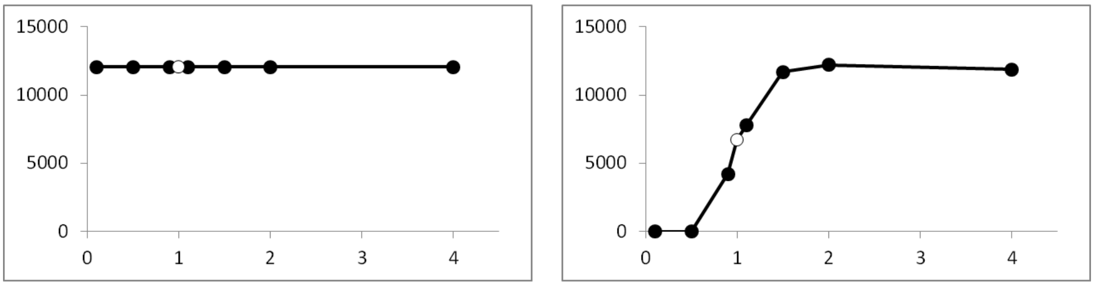

(xxxi) "MITE_MORTALITY_BROODPERIOD" (default mortality = 0.006 per day)

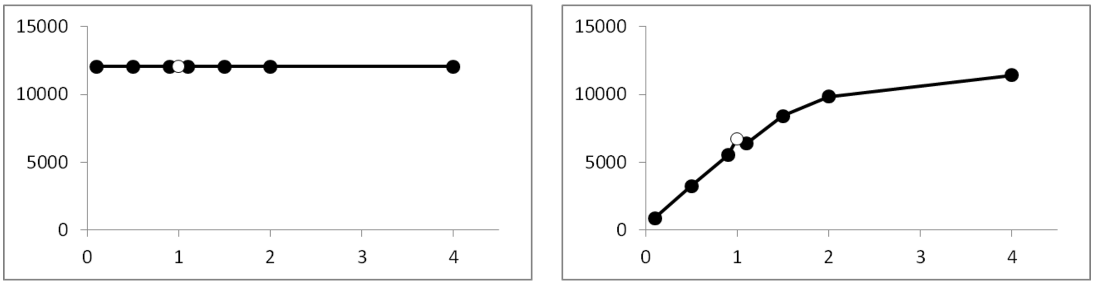

(xxxii) "MITE_MORTALITY_WINTER" (default mortality = 0.002 per day)

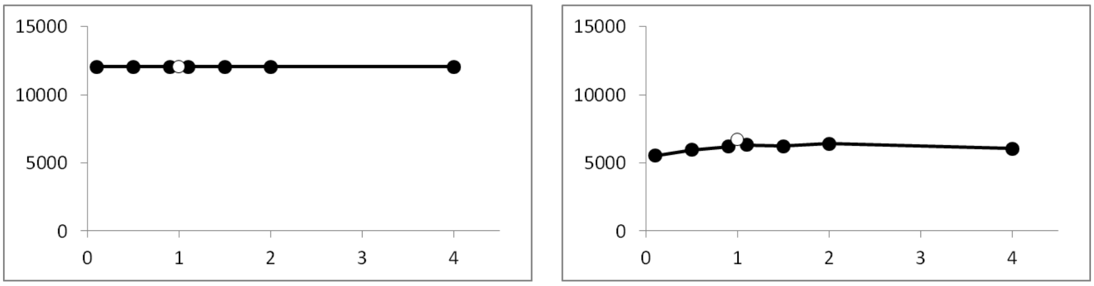

Appendix S4

Without varroa | With varroa

(xxxiii) "MORTALITY_DRONE_EGGS" (default mortality = 0.064 per day)

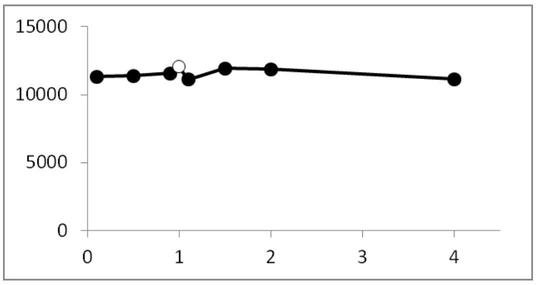

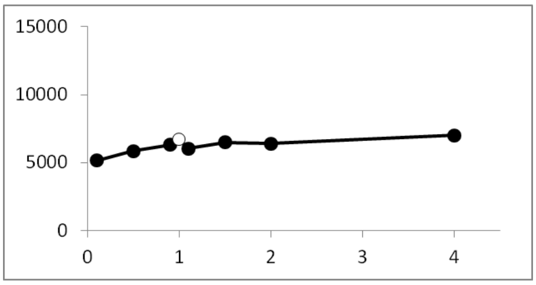

(xxxiv) "MORTALITY_DRONE_LARVAE" (default mortality = 0.044 per day)

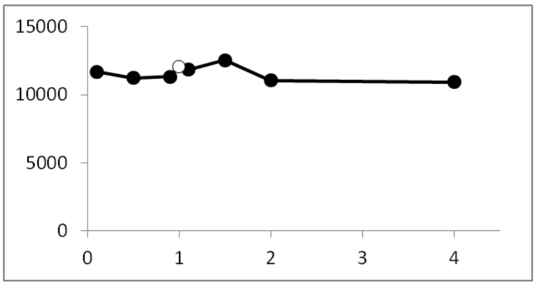

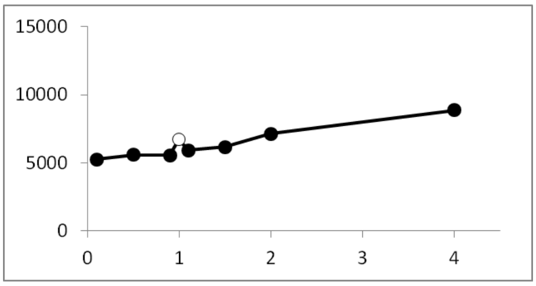

(xxxv) "MORTALITY_DRONE_PUPAE" (default mortality = 0.005 per day)

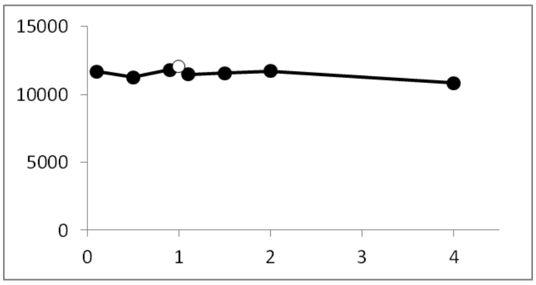

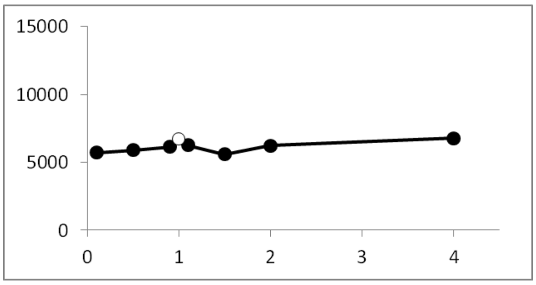

(xxxvi) "MORTALITY_DRONES" (default mortality = 0.05 per day)

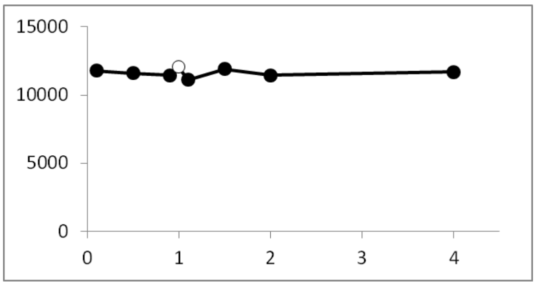

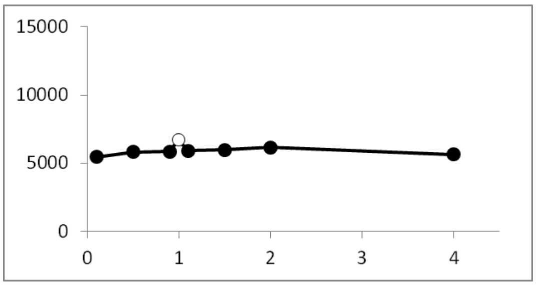

Appendix S4

Without varroa | With varroa

(xxxvii) "MORTALITY_EGGS" (default mortality = 0.03 per day)

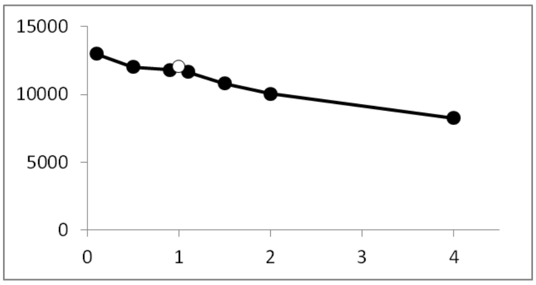

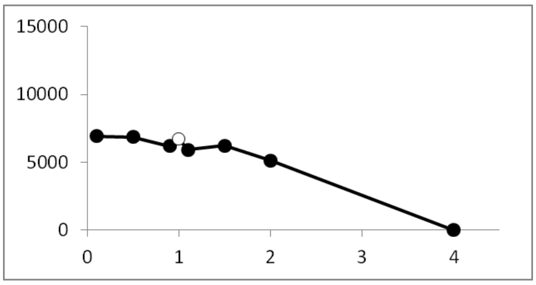

(xxxviii) "MORTALITY_FOR_PER_SEC" (default mortality = 0.00001 per second)

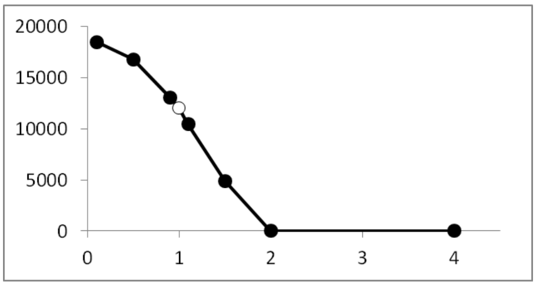

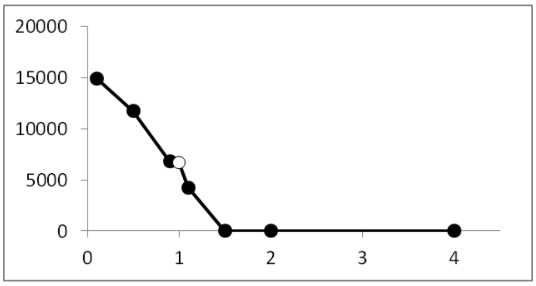

(xxxix) "MORTALITY_INHIVE" (default mortality = 0.004 per day)

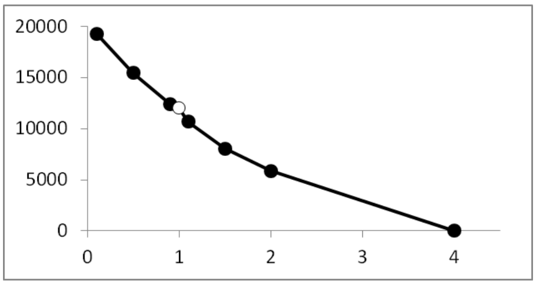

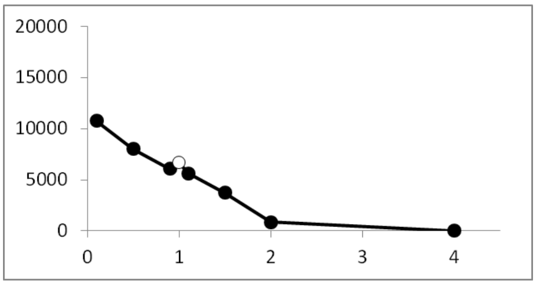

(xl) "MORTALITY_LARVAE" (default mortality = 0.01 per day)

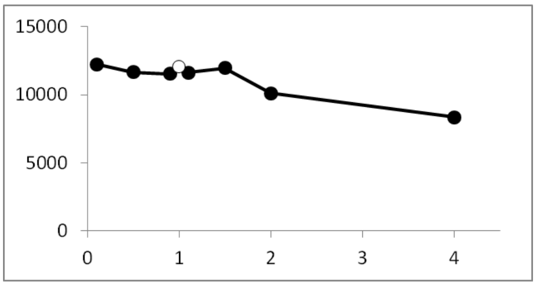

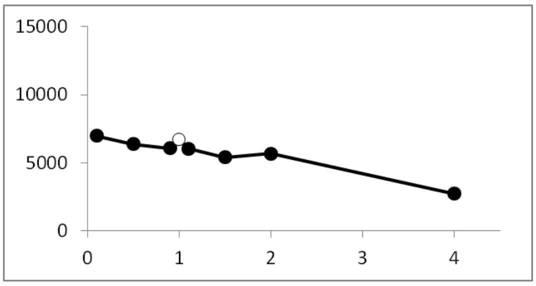

Appendix S4

Without varroa | With varroa

(xli) "MORTALITY_PUPAE" (default mortality = 0.001 per day)

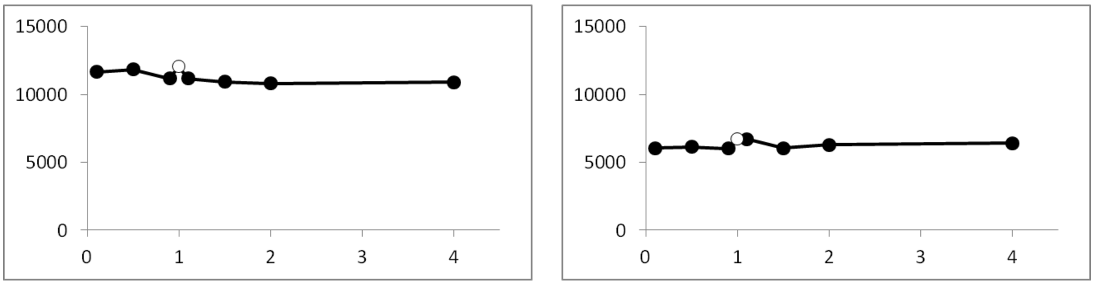

(xlii) "N_INITIAL_BEES" (default = 10000 bees)

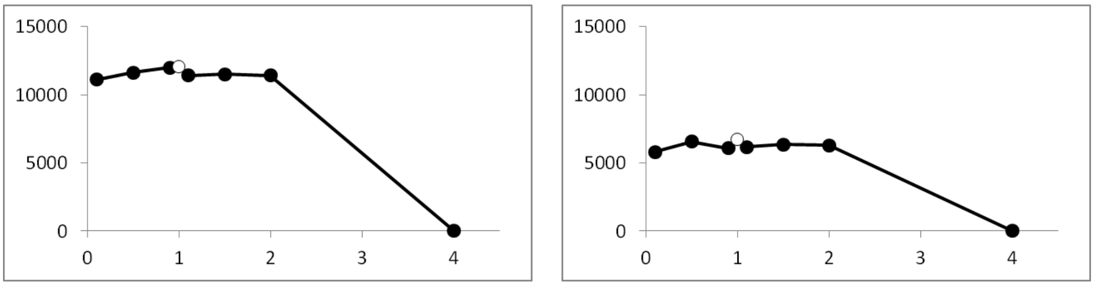

(xliii) "POLLEN_DANCE_FOLLOWERS" (default = 2 bees)

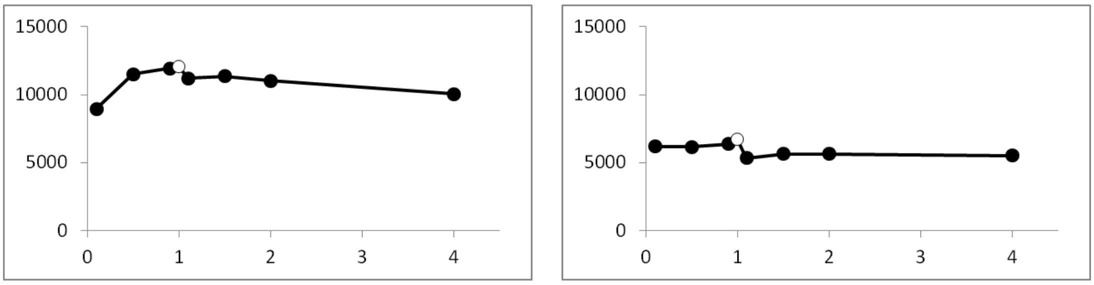

(xliv) "POLLEN_G_kg" (default weight = 1 kg)

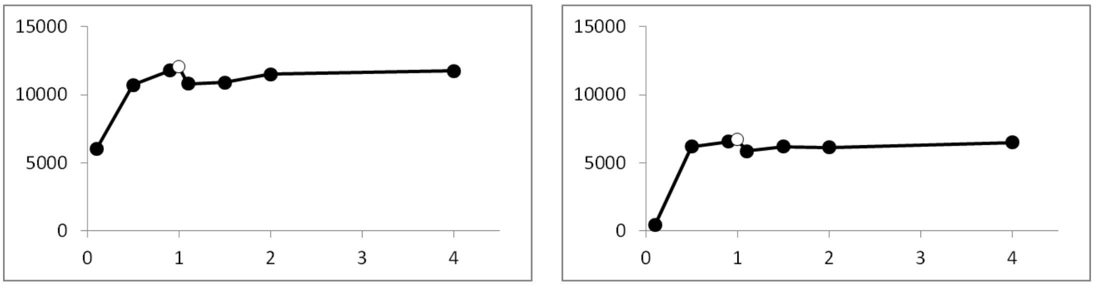

Appendix S4

Without varroa | With varroa

(xlv) "POLLEN_R_kg" (default weight = 1 kg)

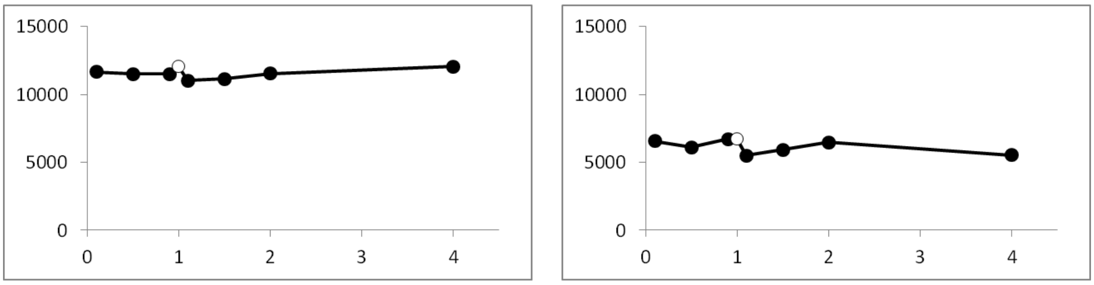

(xlvi) "POLLEN_STORE_INIT" (default weight = 100 g)

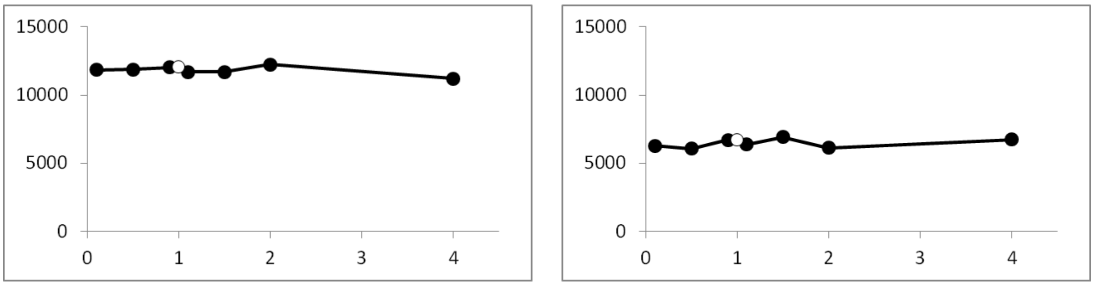

(xlvii) "POLLENLOAD" (default weight = 0.015 g)

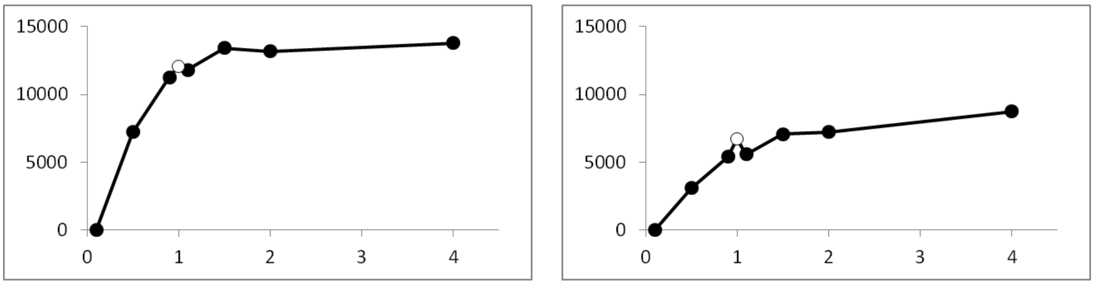

(xlviii) "PRE_SWARMING_PERIOD" (default = 3 days)

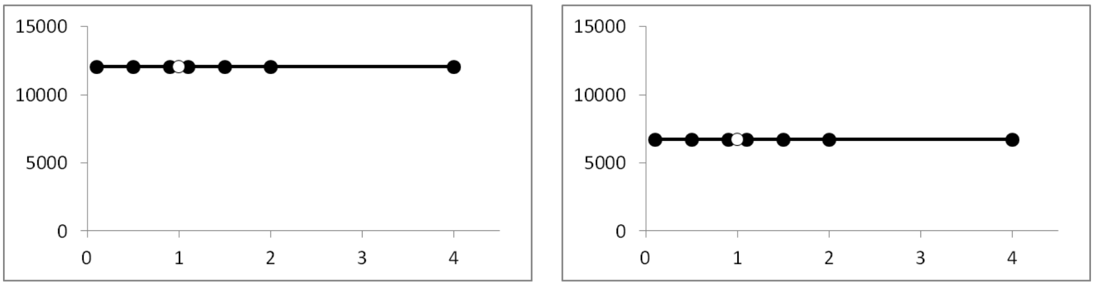

Appendix S4

Without varroa | With varroa

(xlix) "PROTEIN_STORE_NURSES_d" (default = 7 days)

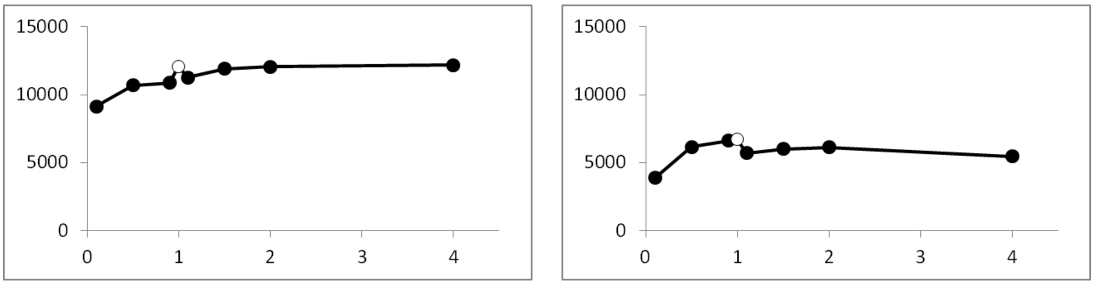

(l) "QUANTITY_G_l" (default amount = 20 litres)

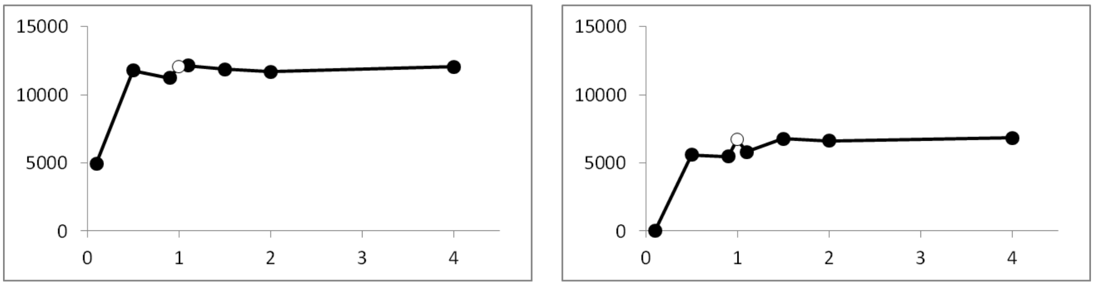

(li) "QUANTITY_R_l" (default amount = 20 litres)

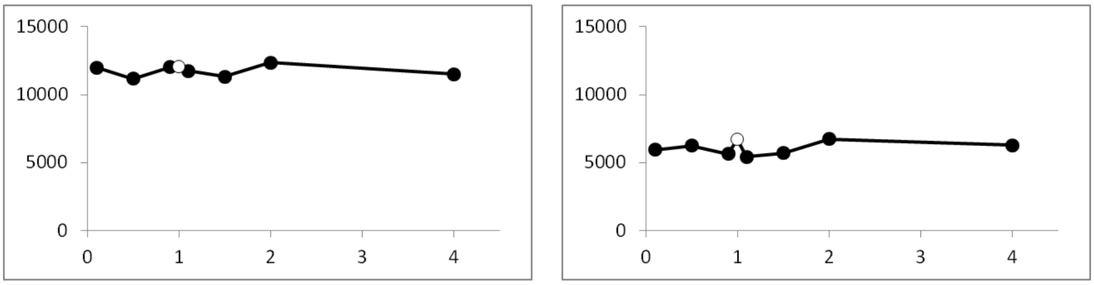

(lii) "SHIFT_G" (default = -40)

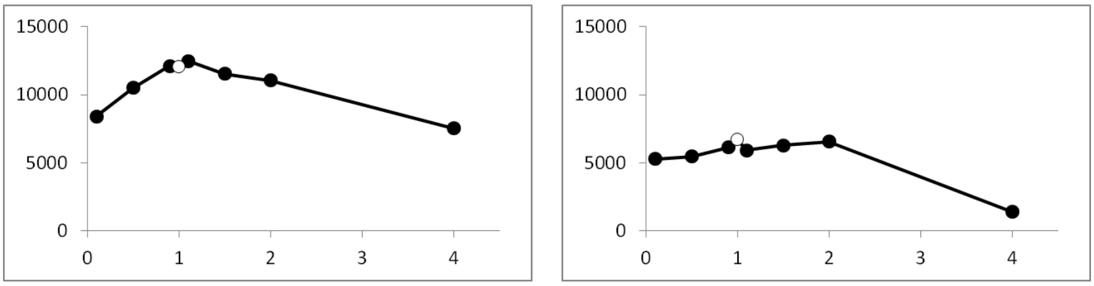

Appendix S4

Without varroa | With varroa

(liii) "SHIFT_R" (default = 30)

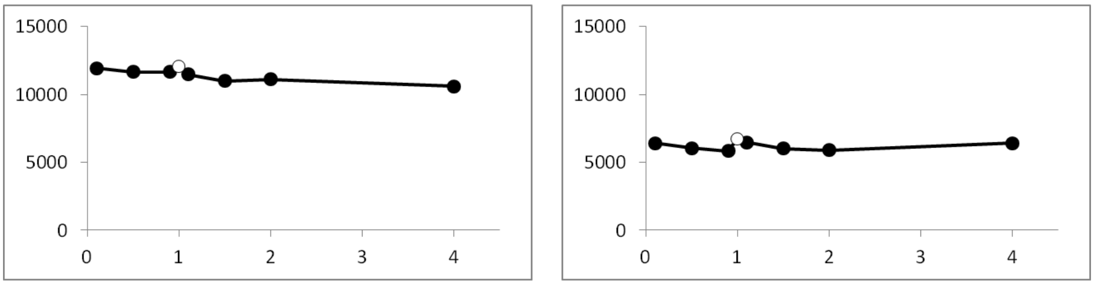

(liv) "TIME_NECTAR_GATHERING" (default time = 1200 seconds)

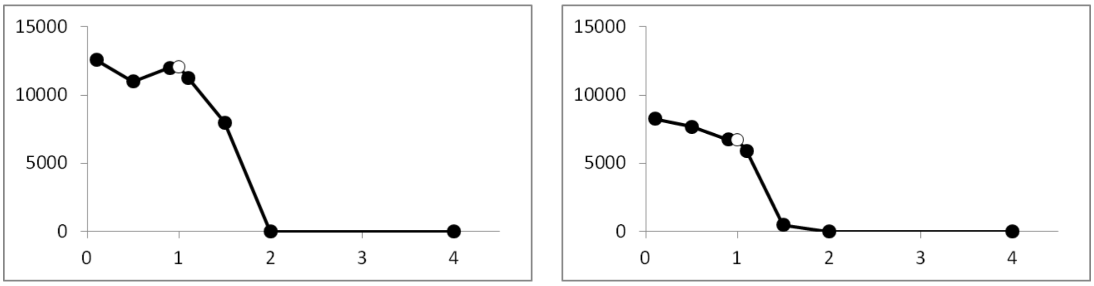

(lv) "TIME_POLLEN_GATHERING" (default time = 600 seconds)

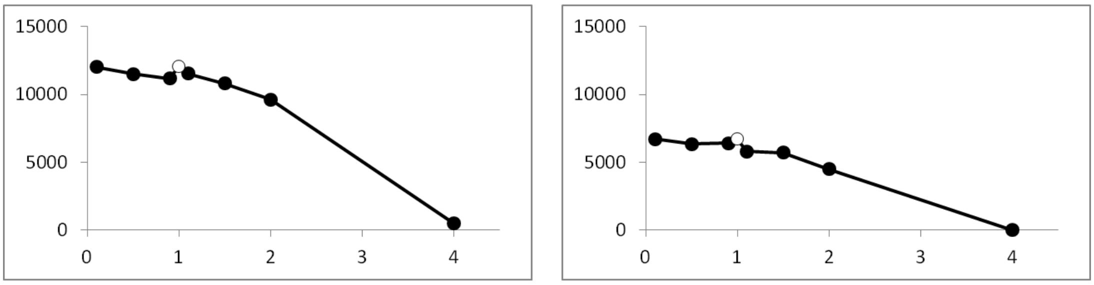

(lvi) "TIME_UNLOADING" (default time = 116 seconds)

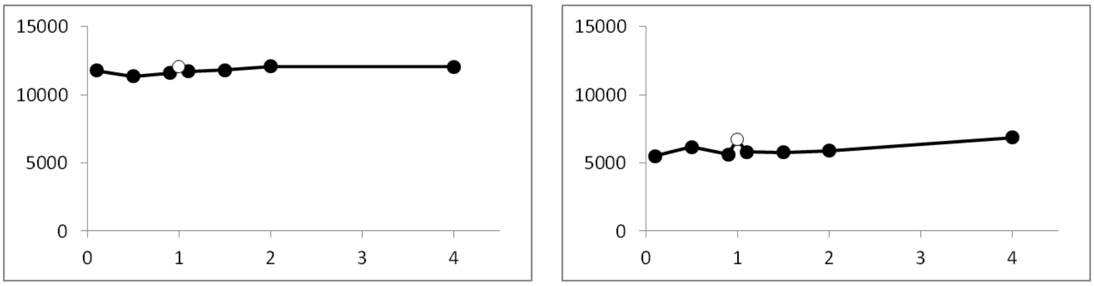

Appendix S4

Without varroa With varroa

(lvii) "TIME_UNLOADING_POLLEN" (default time = 210 seconds)

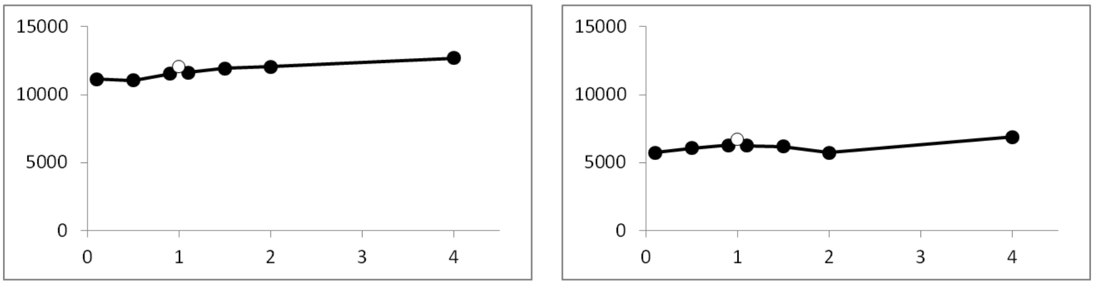

VIRUS SPECIFIC PARAMETERS (Deformed Wing Virus):

(lviii) "MORTALITY_INHIVE_INFECTED_AS_PUPA" (default mortality = 0.012 per day)

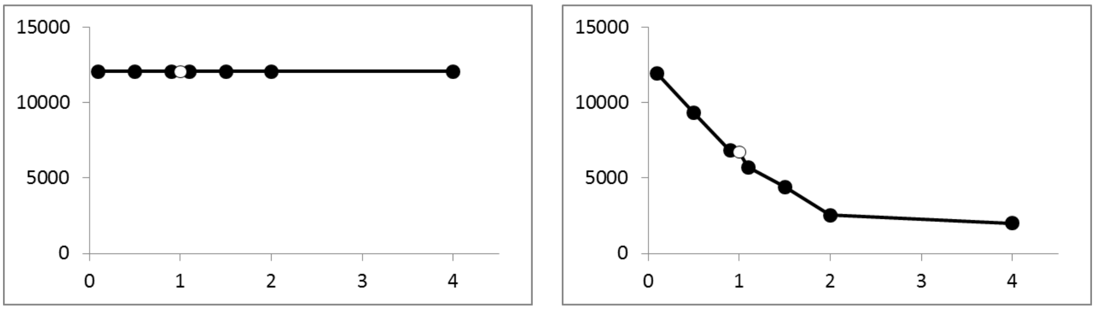

(lix) "VIRUS_KILLS_PUPA_PROB" (default probability = 0.2)

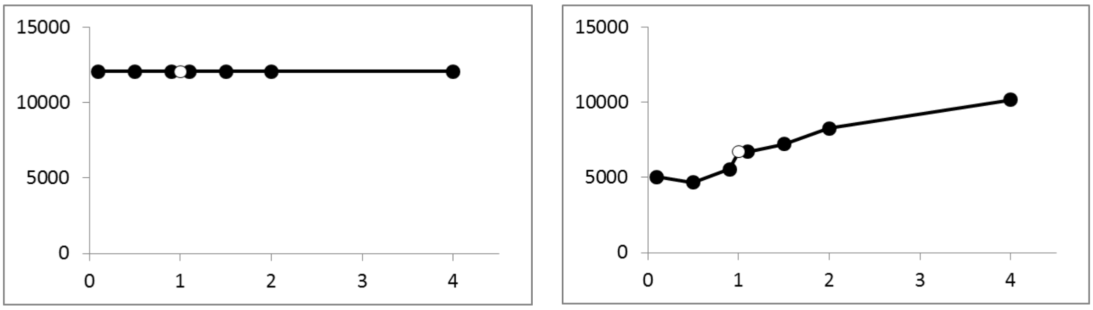

(lx) "VIRUS_TRANSMISSION_RATE_MITE_TO_PUPA" (default probability = 0.89)

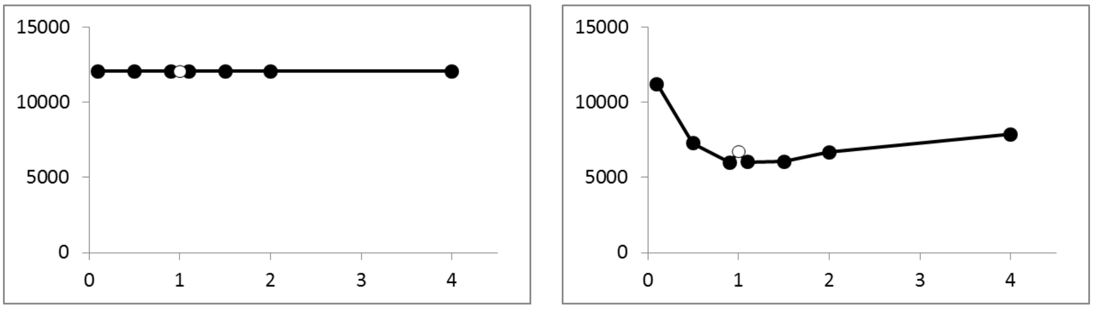

Appendix S4

Without varroa | With varroa

(lxi) "VIRUS_TRANSMISSION_RATE_PUPA_TO_MITES" (default probability = 1)

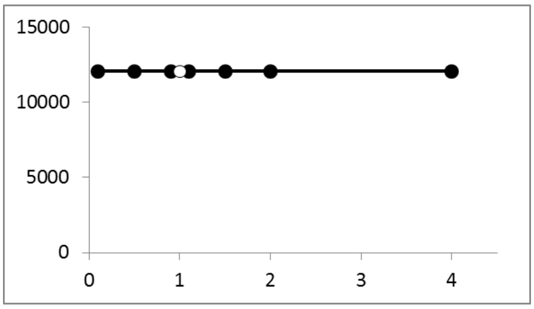

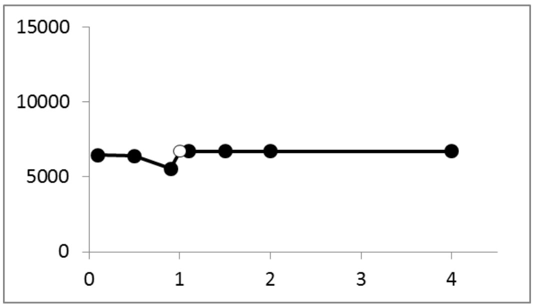

Impact of the number of foragers per super-individual:

(lxii) "SQUADRON_SIZE" (only without varroa) (default number of bees = 100)

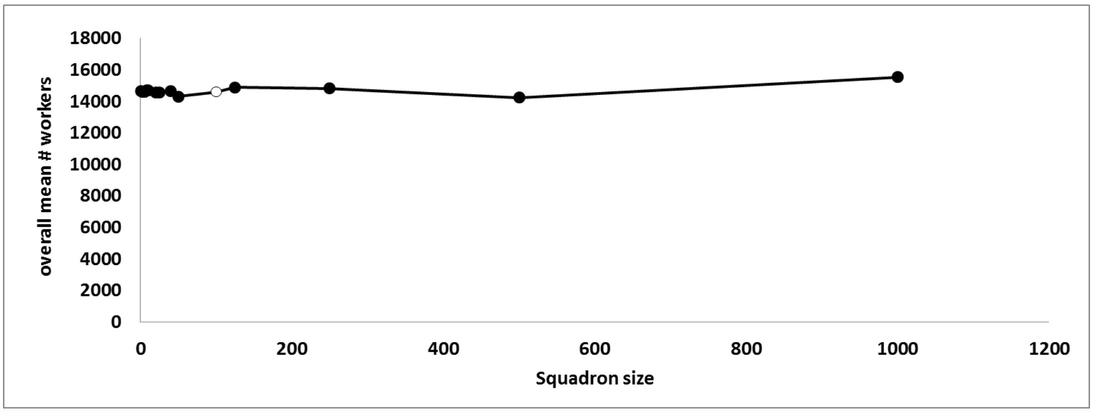

Parameters not included, as they require integer values:

"DRONE_EMERGING_AGE"
"DRONE_HATCHING_AGE"
"DRONE_PUPATION_AGE"
"EMERGING_AGE"
"HATCHING_AGE"
"MAX_AFF"
"MIN_AFF"
"PUPATION_AGE"

Parameters not included, as their default value is 0:

"DANCE_INTERCEPT"
("N_INITIAL_MITES_HEALTHY")
("N_INITIAL_MITES_INFECTED")
"POST_SWARMING_PERIOD"

Parameters not included for other reasons (e.g. because they are constants or do not affect the modelled processes):

"ENERGY_HONEY_per_g"
"ENERGY_SUCROSE"
"INPUT_FILE"
"MAX_BROODCELLS"
"MAX_km_PER_DAY"
"N_FLOWERPATCHES"
"N_GENERIC_PLOTS"
"PATCHCOLOR"
"SEASON_START"
"SEASON_STOP"
"STEPWIDTH"
"STEPWIDTHdrones"
"WEIGHT_WORKER_g"
